# Impact of Social Participation Types on Depression in the Elderly in China: An Analysis Based on Counterfactual Causal Inference

**DOI:** 10.3389/fpubh.2022.792765

**Published:** 2022-04-01

**Authors:** Xiaofeng Wang, Jiamin Guo, Huawei Liu, Tengteng Zhao, Hu Li, Tan Wang

**Affiliations:** Northeast Asian Research Center, Jilin University, Changchun, China

**Keywords:** the elderly, depression, social participation types, counterfactual framework, propensity score matching

## Abstract

**Objectives:**

Depression is the leading cause of ill health and disability worldwide, and has become one of the key issues in the public health sector. Social participation is one of the most important measures to support the rapidly aging population and can reduce the risk of depression in the elderly. This study aims to explore the causal relationship between different types of social participation and depression in the elderly, and reduce the level of depression in the elderly by promoting social participation.

**Methods:**

In order to accurately evaluate the causal relationship between participation in different types of social activities and depression in the elderly, this paper uses propensity score matching (PSM) for analysis based on counterfactual framework. The specific matching methods used are: k-nearest neighbor matching method, kernel matching method and radius matching method.

**Results:**

In this study, 31.4% of the elderly have depression, and the proportion of female elderly is significantly higher. Participation in different social activities has different effects on depression in the elderly. Taking into account sample selection bias, participation in friend-making, exercise, and recreational activities can effectively reduce the risk of depression in the elderly. Compared with other social activities, participation in exercise and recreational activities are more helpful to reduce the risk of depression in the elderly. Participating in exercise activities only reduces the risk of depression in 60–69-year-olds, while participating in economic activities increases the risk of depression in the elderly aged 70 and over. Compared with the male elderly, participation in friend-making, exercise, and recreational activities results in the female elderly having stronger resistance to the risk of depression.

**Conclusions:**

Depression was prevalent among the elderly. Through PSM analysis, different types of social participation have different effects on depression in the elderly. In order to maximize the positive effects of different types of social participation on depression in the elderly, it is necessary to provide differentiated social support environment for the elderly. Expanding the research on the relationship between social participation and depression of the elderly will help to formulate more reasonable public health policies and improve the mental health of the elderly.

## Introduction

In the 21st century, the world is facing an increasingly severe situation of population aging. In 2000, China joined the list of countries with an aging population. According to the data of China's sixth census in 2010, the number of people aged 60 and over was 177.65 million, accounting for 13.26% of the total population (1). By China's seventh census in 2020, the population aged 60 and over was 264.2 million, accounting for 18.70% of the total population (2). China has accelerated its aging process over the past two decades. Compared with other countries that have become old-age countries, China's population aging has two prominent characteristics: first, the fast speed of population aging and the absolute growth of the elderly population; second, the process of population aging is ahead of the level of economic development.

Within the context of population aging, the main diseases of the elderly, such as chronic diseases, disabilities, and mental disorders, have attracted widespread public attention. Depression is a common mental disorder. According to the latest statistics published by the World Health Organization (WHO) in 2019, there are more than 350 million people with depression worldwide (3). Prolonged depression has a significantly adverse impact on patients' work, life, and interpersonal communication. The report “Depression and other common mental disorders” published by the WHO in 2017 shows that depression is the single largest contributor to disability in the world and the main cause of death by suicide, and the elderly have a higher risk of morbidity than the general population (4). According to a national survey, 32.55% of the elderly in China suffer from depression (5). In 2016, the disease burden caused by depression among 60–69-year-old elderly people in China was 1.7 times higher than that in 2000, and there was an accelerated upward trend (3). After entering old age, the elderly face a decline in their physical function, transformation of family roles (such as changed family status, an empty nest, widowhood, etc.), and social roles (changed social status, retirement, changes in leisure activities, etc.). If the elderly cannot adapt to these changes, anxiety and depression are inevitable consequences, and even suicide. Studies have confirmed that the high incidence of suicide among the elderly is related to physical and psychological stress such as depression and helplessness (6). Senile depression has become a social public health problem. Strengthening the research on senile depression has important practical significance for actively addressing the aging of the population.

According to social capital theory, elderly people improve their social capital through social participation, while social capital improves individual health through emotional and material support among social members (7, 8). Socioemotional selectivity theory holds that everyone has different self-needs and resource capital, and social participation is the behavior of meeting self-needs through resource exchange (9). According to activity theory, older people should replace their roles lost due to widowhood or retirement with new ones, so as to adapt to society and obtain psychological satisfaction (10). Elderly people can establish new roles through social participation to alleviate the symptoms of depression caused by role changes. Population migration and the miniaturization of the family structure in China have caused marked changes in intergenerational relationships, increasing the number of elderly people living alone and the proportion of empty nesters. Therefore, the elderly should be encouraged to alleviate their depression through social participation. As an important measure to actively deal with population aging, social participation has attracted widespread public attention. Many studies have shown that active social participation can effectively reduce the risk of depression, improve life satisfaction, and protect the mental health of the elderly (11–15). Participating in social activities can slow down the cognitive decline of the elderly and help protect their mental health (7); the higher the social participation of the elderly, the lower the level of depression (16). However, the complexity of social participation means that different activities have different effects on depression in the elderly, as highlighted by previous studies; participation in religious organizations has the most obvious effect on their health (12). Different types of social participation may affect the welfare, health, and survival risk of the elderly through different mechanisms (17).

There is abundant current research on the influence of social participation on the depression of the elderly, but there are still areas of debate and limitations. First, a debated point is the correlation between different types of social activities and depression in the elderly. Studies have found that participation in mentally stimulating and exercise activities has a certain causal effect on senile depression (18), while participation in helping activities has no significant effect on senile depression (12). However, some studies have pointed out that by participating in voluntary service, the elderly can realize their own value and improve their quality of life (19). The withdrawal of the elderly from work can reduce their sense of control over their lives and lead to a higher incidence of depression (20). However, some studies have pointed out that the physical health and life satisfaction of the elderly increase after retirement, especially the performance of mental workers (21). It is evident that the relationship between different activity types and depression in the elderly is subject to debate. Therefore, it is necessary to classify social activities to study their causal relationship with depression in the elderly, so as to encourage the elderly to choose social activities that are beneficial to their mental health. Second, the existing research does not satisfactorily address the problem of sample selection bias. Whether or not the elderly participate in social activities, and their choice of activities, are not randomly assigned but determined by individual, family, and social characteristics. Thus, it is impossible to accurately judge whether the difference in the level of depression of the elderly is caused by participation in social activities or by differences in individual, family, and social characteristics. The existence of sample selection bias may lead to an underestimation of the impact of social participation on depression in the elderly.

In countries outside China, social participation is mainly defined based on social relations, the exchange of resources, and broader social activities, emphasizing contact and interaction with others in the process of social participation to obtain individual value (22–24). Chinese scholars have conducted a more detailed and localized examination of social participation, and contend that no matter how the elderly maintain contact with society, this is a form of social participation (10). Therefore, this study, based in China, divides the daily activities that Chinese elderly people mainly participate in into five types: friend-making, exercise, recreational, helping, and economic activities, so as to comprehensively compare the heterogeneous effects of various types of social participation on the depression of the elderly. Furthermore, counterfactual inference is a useful tool for comparing outcomes of interventions on complex systems and is widely used in the study of causality (25, 26). In order to more accurately evaluate the causal effects of social participation types and depression in the elderly, this study uses propensity score matching (PSM) based on the counterfactual framework of Rosenbaum and Rubin (27). This will enable more accurate identification of the types of social activities that can alleviate the depression of the elderly, and provides a theoretical basis for improving the mental health and quality of life of the elderly through social participation.

## Materials and Methods

### Data

This study uses the data from the China Health and Retirement Longitudinal Study (CHARLS), which was conducted in 2015. This is a large-scale interdisciplinary survey project based at the National School of Development at Peking University, and jointly implemented by the Institute of Social Science Survey and Youth League Committee of Peking University. CHARLS aims to obtain high-quality micro-data representing Chinese middle-aged and elderly people aged 45 and over, and their families, to analyze the aging problem of the Chinese population and thereby promote interdisciplinary research on this area. The CHARLS national baseline survey was carried out in 2011 and is tracked every 2 years. Its sample covers 150 counties and 450 communities (villages) in 28 provinces (autonomous regions and municipalities) across China. In the national follow-up in 2015, a total of 23,000 respondents from 12,400 households were interviewed. The survey data is publicly available and can be found on GITHUB. The publicly available datasets presented in this study are available from an online repository: https://github.com/Guo-jiamin/CHARLS.

This study analyzes the impact of social participation on the mental health of the elderly based on the 2015 data. According to the study research purpose and needs, we first merged the data set according to the individual IDs, and then cleaned up the data, re-assigned variables, removed missing values, and finally obtained a sample of 7,901 senior citizens aged 60 and over.

### Variables

The outcome variable was set as depression of the elderly; and the level of depression was partially reflected by the CES-D Depression Scale, which was constructed based on the depression-related questions in the 2015 questionnaire. The questionnaire asked the respondents about their feelings and behaviors over the past week, including 10 questions where respondents were presented with a series of negative or positive statements. Negative statements included “I am troubled by some trivial things,” “It is difficult to concentrate when doing things,” and “My sleep is not good.” Positive statements included “I am hopeful for the future” and “I am happy.” For each question, respondents were asked to select from four options to indicate the frequency with which each statement had applied to them in the past week: “Little or not at all (<1 day),” “Not too much (1–2 days),” “Sometimes or half the time (3–4 days),” “Most of the time (5–7 days).” Values of 0–3 were assigned to these options with reverse scoring for positive questions, and the scores of the 10 questions were added up to obtain each respondent's depression level. The total score was between 0 and 30: a higher total score signifies a higher depression level, while a lower total score signifies a lower depression level. A reliability test was conducted on the scale options, which showed that the Cronbach's Alpha was 0.795, and the reliability coefficient was high, meaning that the scale could be used to measure the depression level of the respondents.

In order to know the depression status of the elderly in the sample, we think that when the CES-D value is >10, the elderly have depression, while if the CES-D value is ≤10, the elderly do not have depression (28). As shown in Table 1, the proportion of depression in the elderly is 31.4%, which is similar at different ages. The proportion of depression in female and male is 42.43 and 23.74%, respectively, and female is more prone to depression.

**Table 1:** Depression in the elderly.

**Sub sample**		**No depression (CES-D** **≤10)**		**Depression (CES-D** **>** **10)**		**Total**	
		***N***	**%**	***N***	**%**	***N***	**%**
Age	60–69	3,583	68.68	1,634	31.32	5,217	100
	70 and above	1,836	68.41	848	31.59	2,684	100
Gender	Female	1,867	57.57	1,376	42.43	3,243	100
	Male	3,552	76.26	1,106	23.74	4,658	100
Total		5,419	68.59	2,482	31.41	7,901	100

Although much research has been conducted on the concept of social participation, there is still no uniform definition in academic circles. In this paper, social participation is mainly understood in terms of how the elderly maintain contact with their surrounding environment and maintain interaction with society by participating in social activities. Social participation is thus viewed from a broad perspective; however, it is difficult to include all social activities within the scope of the research. Therefore, the research objects are restricted to the activities that the elderly generally participate in daily. The CHARLS questionnaire asked respondents the following multiple-choice question: “Did you engage in the following social activities in the past month?” and whether they participated in work-related activities. As a result, in this study social participation activities are divided into the following five types: (1) Friend-making activities: interacted with friends; (2) Exercise activities: went to a sport, social, or other kind of club; (3) Recreational activity: played Ma-jong, played chess, played cards, or went to a community club; (4) Helping activities: provided help to family, friends, or neighbors who do not live with you; did voluntary or charity work; cared for a sick or disabled adult who does not live with you; (5) Economic activities: engaged in any work. In this study, these five types of social participation were set as the five treatment variables that affect depression in the elderly, and assigned, respectively. A value of 1 was assigned to a variable if the elderly respondents participated in any activity of a certain type; otherwise, a value of 0 was assigned.

Other covariates that may affect depression in the elderly were divided into four categories: demographic characteristics, health status, family support, and social characteristics. The demographic characteristics variables include age, gender, and education level. Health status variables include activities of daily living (ADL), instrumental activities of daily living (IADL), and self-rated health. Family support variables include marital status, number of children, frequency of meeting with their children, whether their children provide financial support, and whether they are satisfied with their relationship with their children. Social characteristics variables include whether the elderly respondents have medical insurance and whether they have pension insurance. Table 2 shows the specific variable definitions and sample descriptions. Among the 7901 elderly respondents, the average level of depression is 8.453, 31.97% participated in friend-making activities, 7.4% participated in exercise activities, 18.31% participated in recreational activities, 13.64% participated in helping activities, and 55.14% participated in economic activities. Compared to the other types of social participation, more elderly respondents participated in economic and friend-making activities.

**Table 2:** Variable definition and sample description.

**Variable**	**Variable meaning**	**Mean percentage (standard deviation)**
**Outcome variable**		
Depression level	Continuous variable, between 0 and 30	8.453 (6.540)
**Treatment variables**		
Participate in friend-making activities	Yes = 1; no = 0	31.97%
Participate in exercise activities	Yes = 1; no = 0	7.40%
Participate in recreational activities	Yes = 1; no = 0	18.31%
Participate in helping activities	Yes = 1; no = 0	13.64%
Participate in economic activities	Yes = 1; no = 0	55.14%
**Covariate**		
Age	Continuous variable, between 60 and 101	67.777 (6.381)
Male	Male = 1; female = 0	58.95%
Education level	No education = 1; elementary school = 2; junior high school = 3; high school and above = 4	1.987 (0.886)
Difficulty with ADL	Difficulty = 1; no difficulty = 0	26.48%
Difficulty with IADL	Difficulty = 1; no difficulty = 0	36.63%
Self-rated health	Healthy = 1; unhealthy = 0	22.76%
Married	With spouse = 1; without spouse = 0	81.57%
Number of children	Continuous variable, between 1 and 11	3.248 (1.474)
Meet with children often	Often = 1; not often = 0	79.00%
Children give financial support	Yes = 1; no = 0	86.27%
Good relationship with children	Satisfied = 1; dissatisfied = 0	95.00%
Have medical insurance	Yes = 1; no = 0	91.85%
Have pension insurance	Yes = 1; no = 0	61.70%

### Method

#### Counterfactual Framework

In order to accurately determine the causal relationship between participating in different types of social activities and the depression of the elderly, this study applied the PSM method to construct a counterfactual framework for the effects of participating in five social activities on the depression of the elderly (27). Counterfactual refers to a potential outcome or state of affairs that occurs when cause does not exist. Therefore, for the members in the treatment condition, the counterfactual is the potential outcome in the condition of control; for the members in the condition of control, counterfactual is the potential outcome in the treatment condition. The counterfactual framework emphasizes that individuals entering the treatment group and the control group have potential outcomes in both states. The counterfactual framework can be expressed as the following model:


(1) $$\begin{array}{r} Y_{i}=D_{i}Y_{1i}+\left(1-D_{i}\right)Y_{0i} \end{array}$$

In Equation (1), it is assumed that the individual *i* in the treatment group will have two outcomes (*Y*_1*i*_, *Y*_0*i*_), corresponding to the actual outcome and the counterfactual potential outcome, respectively. *D*_*i*_ = 1 means to accept the treatment, *D*_*i*_ = 0 means not to accepted the treatment, and *Y*_*i*_ means the measured outcome variable. Based on such a counterfactual framework, the causal analysis becomes the difference between *Y*_1*i*_ and *Y*_0*i*_. For friend-making activities in this article, the elderly who participated in such activities were used as the treatment group, and the non-participating elderly were used as the control group. To study the causal relationship between participating in the friend-making activities and the depression of the elderly, it was necessary to determine the depression level of the respondent sample in the two states of participating in and not participating in the activity. Since each individual can only be in one state in reality, it was necessary to construct a counterfactual state to study the net effect of participating in this activity on the depression of the elderly. This counterfactual framework was also used to analyze exercise, recreational, helping, and economic activities.

As shown in Table 3, *Y*^*T*^ and *Y*^*Z*^ represent the actual depression level and counterfactual potential depression level, respectively. If individual *i* participates in such activities, the depression level after participation can be obtained ($Y_{1i}^{T}$), but the potential depression level of non-participation cannot be known ($Y_{0i}^{Z}$). Similarly, if individual *i* does not participate in such activities, then the non-participated depression level can be obtained ($Y_{0i}^{T}$), but the potential depression level of participation cannot be known ($Y_{1i}^{Z}$). Therefore, it is necessary to construct the potential depression level $Y_{0i}^{Z}$ and $Y_{1i}^{Z}$, that is, the counterfactual potential depression level. Equation (2) represents average treatment effect for the treated (ATT), and Equation (2) represents average treatment effect for the untreated (ATU). The main purpose of this study is to examine the changes in the level of depression in the treatment group after they participated in five social activities, so only the ATT value is observed.

**Table 3:** Counterfactual inference of the impact of participation in social activities on depression in the elderly.

**Participation or non-participation in the activity**	**Depression level**		**Causal effect**
Treatment group: participation (*D*_*i*_ = 1)	Actual outcome $Y_{1i}^{T}$	Counterfactual potential outcome $Y_{0i}^{Z}$	$ATT=E\left(Y_{1i}^{T}-Y_{0i}^{Z}|D_{i}=1\right)$ (2)
Control group: non-participation (*D*_*i*_ = 0)	Actual outcome $Y_{0i}^{T}$	Counterfactual potential outcome $Y_{1i}^{Z}$	$ATU=E\left(Y_{0i}^{T}-Y_{1i}^{Z}|D_{i}=0\right)$ (3)

#### Propensity Score Matching

The above analysis shows that it is necessary to construct counterfactual potential outcome variables. It is not possible to know the counterfactual state of the same individual, but it is possible to construct a matching individual with the same or similar characteristics. These characteristics can be represented by the probability of entering the treatment state (propensity score *P*), that is, the characteristics of multiple dimensions are compressed into one dimension. According to the similar propensity score *P*, the treatment group and the control group are matched, and then ATT is obtained by comparing the differences in the outcome variables of the two groups. The propensity score *P* is affected by individual, family, social, and other factors. These factors also affect both the treatment variable and the outcome variable, which leads to sample selection bias. We defined these factors that lead to sample selection bias as covariates or confounding variables. Taking into account sample selection bias, Equation (2) can be rewritten as equation (4):


(4) $$\begin{array}{l} ATT=E[Y_{1}^{T}|D_{i}=1,P(D_{i}=1|X_{i})]-E[Y_{0}^{Z}|D_{i}\;\\ \;=1,P(D_{i}=1|X_{i})] \end{array}$$

*P*(*D*_*i*_ = 1|*X*_*i*_) is the probability of an individual entering the intervention state based on controlling the covariate *X*_*i*_, and *X*_*i*_ is the covariate that may affect the participation and depression of the elderly, including age, gender, education level, health status, and other variables.

**Figure 1:**
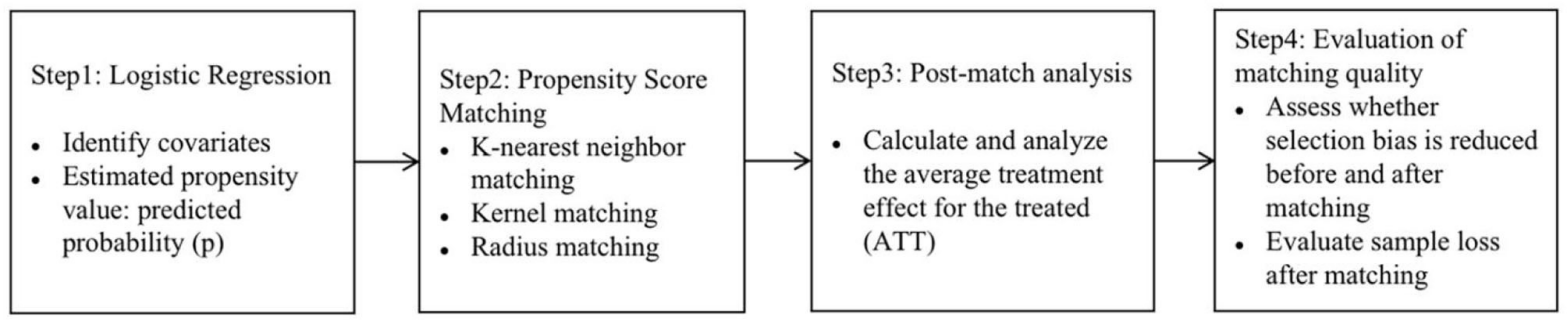
The steps of PSM.

Based on the above ideas, PSM was proposed by Rosenbaum and Rubin (27), and it is a method to evaluate causal effects using non-experimental data. This method has the advantage of reducing the multiple dimensions of covariates and compressing them into a one-dimensional score, and then using the one-dimensional score for matching, which can better solve the problem of sample selection bias. Therefore, based on the counterfactual framework, this study adopts PSM to analyze the causal relationship between the types social participation and depression in the elderly. *K*-nearest neighbor matching (29, 30), kernel matching (29, 31) and radius matching (29) are more commonly matching method used in PSM, and these three methods are also used in this study. The analysis steps of PSM in this study are shown in Figure 1.

## Results

### Logistic Estimation Results of Different Social Activities

According to the research steps of the PSM method, the first step involves estimating the respective probability of the elderly respondents participating in five social activities. These five social activities were set as the dependent variables, while age, gender, education level, self-rated health, marriage, and other characteristics were set as the independent variables. Logistic regression was then used to obtain the propensity scores of the elderly respondents participating in the five social activities.

Table 4 shows that the elderly respondents' participation in friend-making, exercise, recreational, helping, and economic activities is not random, but is affected by demographic characteristics, health status, family support, and social characteristics. With increasing age, the probability of the elderly participating in exercise, helping, and economic activities decreases. Female elderly have a higher probability of participating in friend-making and exercise activities, while male elderly have a higher probability of participating in recreational and economic activities. Respondents with a higher level of education have an increased probability of participating in friend-making, exercise, recreational, and helping activities, but a decreased probability of participating in economic activities. The elderly with good health have a higher probability of participating in exercise, recreational, and economic activities. The elderly without a spouse have a higher probability of participating in friend-making and exercise activities, while the elderly with a spouse and more children have a higher probability of participating in economic activities. The effects of medical insurance and pension insurance on the participation of the elderly in different social activities are also different.

**Table 4:** Logistic estimation results of five types of social activities.

**Variable**	**Friend-making activities**	**Exercise activities**	**Recreational activities**	**Helping activities**	**Economic activities**
	**Exp(B)**	**Exp(B)**	**Exp(B)**	**Exp(B)**	**Exp(B)**
Age	0.997 (0.004)	0.985^*^ (0.008)	0.993 (0.006)	0.964^***^ (0.006)	0.884^***^ (0.004)
Gender	0.717^***^ (0.041)	0.710^***^ (0.075)	1.615^***^ (0.118)	1.135 (0.089)	1.362^***^ (0.079)
Education level	1.312^***^ (0.042)	1.695^***^ (0.089)	1.300^***^ (0.048)	1.240^***^ (0.052)	0.690^***^ (0.023)
ADL	1.017 (0.065)	0.924 (0.118)	0.956 (0.080)	1.029 (0.092)	0.720^***^ (0.046)
IADL	0.929 (0.055)	0.713^***^ (0.085)	0.550^***^ (0.044)	0.881 (0.074)	0.751^***^ (0.045)
Self-rated health	0.885^***^ (0.035)	0.801^***^ (0.058)	1.006 (0.049)	0.841^***^ (0.046)	0.812^***^ (0.033)
Marital status	0.712^***^ (0.046)	0.653^***^ (0.077)	0.874 (0.074)	0.874 (0.082)	1.631^***^ (0.111)
Number of children	1.002 (0.019)	0.887^***^ (0.034)	0.911^***^ (0.022)	0.941^**^ (0.026)	1.130^***^ (0.022)
Frequency of meeting with children	0.912 (0.055)	1.307^**^ (0.151)	1.054 (0.078)	0.890 (0.071)	0.577^***^ (0.037)
Financial support	1.201^**^ (0.089)	1.299^**^ (0.168)	1.299^***^ (0.116)	1.384^***^ (0.143)	1.303^***^ (0.096)
Relationship with children	0.952 (0.108)	1.139 (0.267)	1.145 (0.176)	1.104 (0.184)	0.835 (0.097)
Medical insurance	1.234^**^ (0.116)	1.693^***^ (0.344)	1.380^**^ (0.178)	0.972 (0.125)	1.033 (0.097)
Pension insurance	0.995 (0.053)	0.648^***^ (0.061)	0.970 (0.062)	1.041 (0.075)	2.185^***^ (0.119)
Constant	0.540^*^ (0.191)	0.129^***^ (0.087)	0.142^***^ (0.064)	1.666 (0.842)	6618.136^***^ (2530.464)
Adjusted R2	0.014	0.069	0.053	0.025	0.142
Sample size	7901	7901	7901	7901	7901

*, **, *** *represent the significance levels of 10, 5, and 1%, respectively; standard errors are given in brackets*.

In general, the decision to participate in social activities is intervened in by covariates such as age, gender, and education level. This kind of intervention allocation leads to sample selection bias, and the use of OLS regression cannot solve this problem well. Therefore, in order to more accurately evaluate the causal relationship between the five social activities and depression in the elderly, it was essential to analyze the data using PSM.

### Propensity Score Matching Estimation Results

After obtaining propensity scores, these scores were used to match treatment group and control group members. The core idea of matching is to create a new sample after obtaining the propensity scores, so that the cases in it could be assigned to the treatment group with roughly the same possibility. In order to ensure the robustness of the results, k-nearest neighbor matching, radius matching, and kernel matching were used to investigate ATT of participating in five types of social activities on depression in the elderly. The matching results are shown in Table 5.

**Table 5:** ATT of participating in five types of social activities on depression in the elderly.

**Types of activities**	**Matching method**	**Depression level**		
		**ATT**	**Std. Err**.	**T-stat**
Friend-making activities	Before matching	−0.746^***^	0.158	−4.73
	K-nearest neighbor matching	−0.359^**^	0.173	−2.07
	Radius matching	−0.523^***^	0.157	−3.33
	Kernel matching	−0.549^***^	0.156	−3.52
Exercise activities	Before matching	−2.006^***^	0.280	−7.16
	K-nearest neighbor matching	−0.624^**^	0.290	−2.15
	Radius matching	−0.790^***^	0.262	−3.01
	Kernel matching	−0.978^***^	0.258	−3.78
Recreational activities	Before matching	−2.049^***^	0.189	−10.85
	K-nearest neighbor matching	−0.742^***^	0.196	−3.79
	Radius matching	−0.727^***^	0.176	−4.12
	Kernel matching	−0.813^***^	0.175	−4.64
Helping activities	Before matching	−0.668^***^	0.214	−3.12
	K-nearest neighbor matching	0.084	0.234	0.36
	Radius matching	0.079	0.211	0.38
	Kernel matching	0.080	0.234	0.34
Economic activities	Before matching	−0.122	0.148	−0.83
	K-nearest neighbor matching	0.292	0.207	1.41
	Radius matching	0.263	0.188	1.40
	Kernel matching	0.260	0.184	1.42

*k-nearest neighbor matching (k = 4); radius matching (caliper value = 0.01)*;

**, *** *represent the significance levels of 5% (1.96 ≤ |T| <2.58) and 1% (|T| ≥ 2.58), respectively*.

The results show significant differences in the level of depression between the treatment group and the control group. Before matching, there was a significant correlation between friend-making, exercise, recreational, and helping activities, and the level of depression. Compared with the elderly who did not participate in these activities, those who participated in friend-making, exercise, recreational, and helping activities could reduce their depression level in an effective manner. After matching using k-nearest neighbor matching, radius matching, and kernel matching, the ATT of friend-making activities, exercise activities, and recreational activities are still significant. The absolute value of ATT for participating in friend-making activities is between 0.3 and 0.6; the absolute value of ATT for participating in exercise activities is between 0.6 and 1.0; and the absolute value of ATT for participating in entertainment activities is between 0.7 and 0.9. The average treatment effects of the three activities are: −0.477, −0.797, and −0.760. It can also be said that the depression level of the elderly was reduced by 0.477, 0.797, and 0.760 units by participating in friend-making, exercise, and recreational activities, respectively. In general, participation in these activities can effectively reduce the risk of depression in the elderly, while participation in exercise and recreational activities are more helpful to reduce the risk of depression in the elderly.

### Evaluation of Matching Quality

After PSM, it was necessary to evaluate the quality of matching. The evaluation involves two aspects: evaluating whether sample selection bias was reduced before and after matching; and evaluating the loss of the sample after the matching.

Sample selection bias can be evaluated by testing the balance of covariates. The balance test mainly examines whether the matched covariates are still significantly different between the treatment group and the control group. The balance of data can be judged by observing the significance (*P* > chi2), the degree of variation of the standard bias (MeanBias, MedBias) and the size of the balance coefficient B and R of the covariates before and after matching. The joint significance test of covariates is significant before matching and no longer significant after matching, which shows that the covariate difference between the treatment group and the control group is small and the matching result is good. The absolute value threshold of the standard bias of PSM is generally selected as 5 or 10%. If the standard deviation is less than the threshold, the match can be considered to pass the balance test. At the same time, if the balance coefficient B is <25 and the *R* value is between 0.25 and 2, the matching effect can be considered good. Table 6 shows the test results. After matching, the covariates were no longer significant; the standard bias of the covariates of the five social activities decreased significantly, and all were <5%; the coefficients B are <25 and the R values are about 1. The test results show that the covariates between the treatment group and the control group are effectively balanced, indicating that sample matching effect is good and the sample selection bias is well-handled.

**Table 6:** Balance test results of covariates before and after propensity score matching.

**Types of activities**	**Matching method**	**Ps R2**	**P > chi2**	**MeanBias**	**MedBias**	**B**	**R**
Friend-making	Before matching	0.014	0.000	5.700	5.100	28.7^*^	1.020
activities	K-nearest neighbor matching	0.000	1.000	1.100	1.100	3.900	1.000
	Radius matching	0.000	1.000	0.500	0.400	2.300	1.030
	Kernel matching	0.001	0.994	1.000	0.500	5.400	1.090
Exercise	Before matching	0.068	0.000	20.900	19.700	72.4^*^	1.180
activities	K-nearest neighbor matching	0.002	0.998	2.100	2.000	9.900	1.080
	Radius matching	0.001	1.000	1.600	1.900	5.700	0.970
	Kernel matching	0.003	0.972	4.100	3.600	13.300	0.910
Recreational	Before matching	0.054	0.000	18.500	14.500	61.0^*^	0.740
activities	K-nearest neighbor matching	0.001	0.964	1.900	1.400	8.700	1.080
	Radius matching	0.000	1.000	0.700	0.600	2.800	1.010
	Kernel matching	0.001	0.997	1.500	1.000	6.700	1.020
Helping	Before matching	0.025	0.000	13.100	11.300	41.2^*^	0.940
activities	K-nearest neighbor matching	0.001	0.992	1.800	1.500	8.500	0.940
	Radius matching	0.000	1.000	0.400	0.300	1.800	1.030
	Kernel matching	0.001	0.986	2.700	2.100	9.100	1.140
Economic	Before matching	0.142	0.000	21.100	18.900	93.1^*^	0.690
activities	K-nearest neighbor matching	0.001	0.194	1.700	1.100	8.900	1.230
	Radius matching	0.002	0.119	2.300	2.100	10.900	1.160
	Kernel matching	0.002	0.122	2.300	2.100	10.700	1.140

* *represent the values if B > 25*.

**Figure 2:**
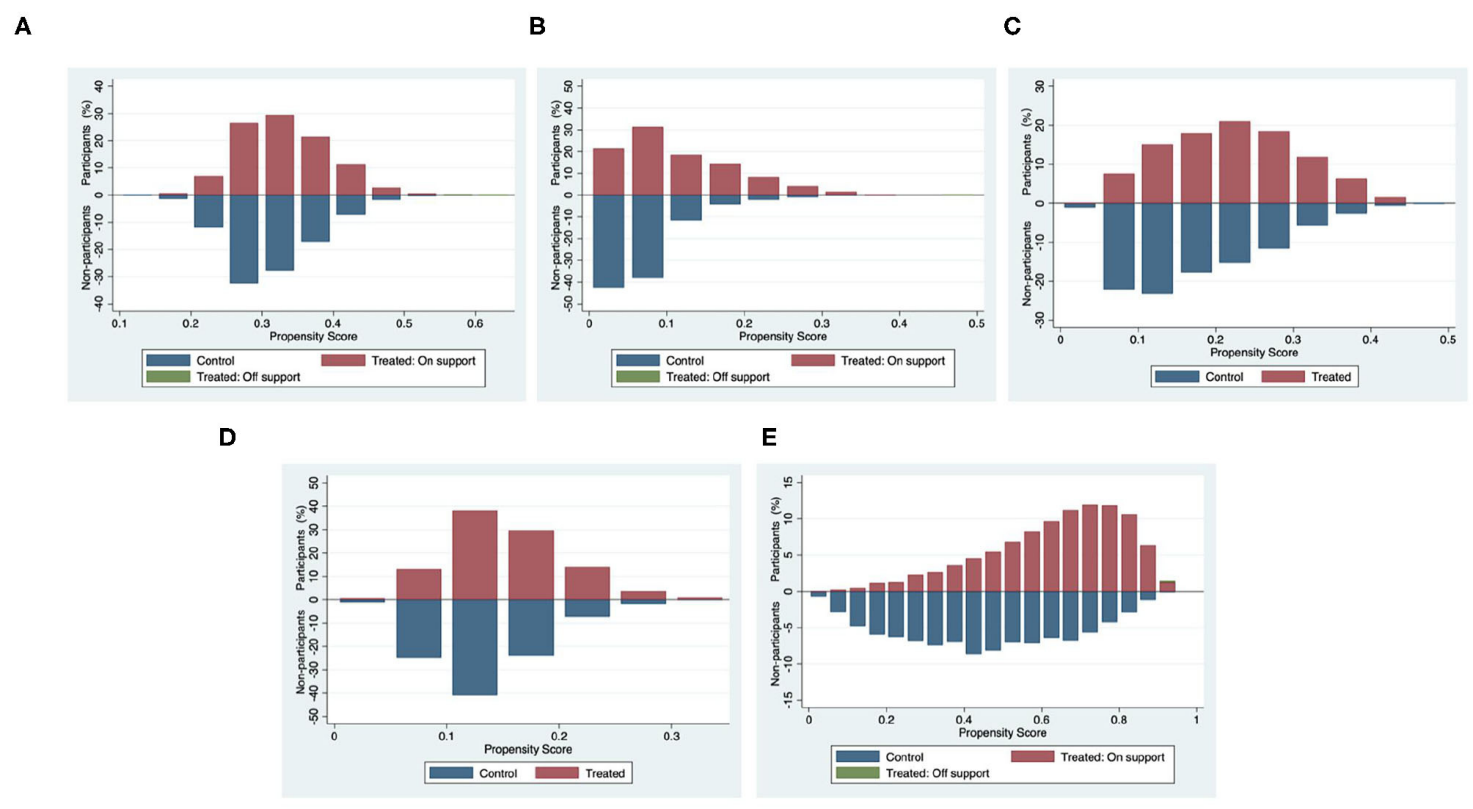
Common value range of propensity scores of participating in five types of social activities. The graphs show percentage shares of participants and non-participants in two states: the treatment group and the control group (the shares for each group add to 100%). The propensity scores of participating in each type of social activity are measured on the horizontal axis (the value range is in [0, 1]). **(A)** Friend-making activities. **(B)** Exercise activities. **(C)** Recreational activities. **(D)** Helping activities. **(E)** Economic activities.

The loss of the matched sample can be evaluated by finding the common value range of the propensity scores. The premise of matching is to ensure that the value range of the propensity scores of the treatment group and the control group have a common support field. Otherwise, the control group individuals that can match the treatment group individuals will not be found. In order to improve the matching quality, only individuals whose propensity scores are in the common value range are usually retained, and the samples outside the common value range will be lost. If the propensity scores of the control group and the treatment group have a large enough range, then the sample loss after matching will be less, which meets the matching requirements. Conversely, if the common value range is too small, a large number of samples will be lost, leading to bias. Each subfigure in Figure 2 graphs the propensity score histogram by treatment status, which shows that the propensity scores for both the treatment and control groups are mostly within common values (on support), and the number of samples lost after PSM is very small (off support), so it is suitable for matching.

### Bias-Corrected Matching Estimator

Due to the uncertainty in estimating propensity score in the first stage of PSM, imprecise matching generally has bias. Abadie and Imbens proposed a method of bias-corrected matching estimator and considering heteroscedasticity robust standard error (32). Based on the counterfactual framework, only two results exist in the research object: observed and unobserved results. The unobserved result is missing data, which is estimated according to the matching estimator of the sample data. That is, the missing value is filled with a “vector norm” similar to the propensity score, and the filled data can be directly estimated (33). In order to make the results more robust and reliable, this study explored the average treatment effect of participating in five types of social activities on depression in the elderly based on bias-corrected matching estimator. The results are shown in Table 7.

**Table 7:** Sample average treatment effect for the treated using the bias-corrected estimator estimates.

**Types of activities**	**SATT**	**Std. Err**.	**z**	**P>z**	**95%CI**
Friend-making activities	−0.532	0.161	−3.300	0.001	[−0.848, −0.216]
Exercise activities	−0.617	0.248	−2.480	0.013	[−1.103, −0.130]
Recreational activities	−0.746	0.170	−4.400	0.000	[−1.078, −0.414]
Helping activities	0.181	0.201	0.900	0.368	[−0.213, 0.574]
Economic activities	0.271	0.165	1.650	0.100	[−0.051, 0.594]

The results show that after correcting the matching bias, friend-making, exercise, and recreational activities still have a significant impact on the depression of the elderly (*P* < 0.1), and the average treatment effect of participating in helping and economic activities on the depression of the elderly is still not significant. Friend-making, exercise, and recreational activities reduce the depression level of the elderly by 0.532 units, 0.617 units, and 0.746 units, respectively. The analysis of Table 5 shows that the depression level of the elderly was reduced by 0.477, 0.797, and 0.760 units by participating in friend-making, exercise, and recreational activities, respectively. The results of the two methods are similar, indicating that the estimation results of ATT in PSM are robust.

### Matching Estimation Results by Age and Gender

As shown in Table 8, the matching results show that there are age and gender differences in the impact of five types of social activities on the depression of the elderly.

**Table 8:** Average intervention effect by age and gender matching.

**Types of activities**	**Matching method**	**Age (ATT)**		**Gender (ATT)**	
		**60-69**	**70 and above**	**Female**	**Male**
Friend-making	Before matching	−0.719^***^	−0.798^***^	−0.880^***^	−0.807^***^
activities	K-nearest neighbor matching	−0.540^**^	−0.546^*^	−0.612^**^	−0.252
	Radius matching	−0.490^***^	−0.511^*^	−0.747^***^	−0.382^**^
	Kernel matching	−0.550^***^	−0.499^*^	−0.800^***^	−0.428^**^
Exercise	Before matching	−1.919^***^	−2.195^***^	−2.294^***^	−1.543^***^
activities	K-nearest neighbor matching	−0.820^**^	−0.658	−1.658^***^	−0.499^*^
	Radius matching	−0.756^**^	−0.634	−1.363^***^	−0.511^*^
	Kernel matching	−0.926^***^	−0.915^*^	−1.790^***^	−0.487^*^
Recreational	Before matching	−2.034^***^	−2.065^***^	−2.662^***^	−1.104^***^
activities	K-nearest neighbor matching	−0.568^**^	−0.680^*^	−1.233^***^	−0.497^**^
	Radius matching	−0.782^***^	−0.672^**^	−1.606^***^	−0.441^**^
	Kernel matching	−0.850^***^	−0.723^**^	−1.763^***^	−0.474^**^
Helping	Before matching	−0.668^***^	−0.621	0.029	−0.726^***^
activities	K-nearest neighbor matching	0.368	0.204	0.082	−0.169
	Radius matching	0.075	−0.128	0.267	−0.182
	Kernel matching	−0.065	−0.104	0.152	−0.279
Economic	Before matching	−0.141	0.032	−0.455^*^	0.047
activities	K-nearest neighbor matching	0.223	0.877^***^	0.310	0.189
	Radius matching	0.073	0.749^***^	0.195	0.077
	Kernel matching	0.100	0.746^***^	0.183	0.056

*k-nearest neighbor matching (k = 4)*;

*, **, *** *represent the significance levels of 10, 5, and 1%, respectively*.

The matching results by age show that there is no age difference in the influence of participating in friend-making and recreational activities on depression in the elderly. Participation in friend-making activities can reduce the depression level of the elderly by about 0.5 units, and recreational activities can reduce the depression level of the elderly by 0.7 units. Participation in exercise activities only have a significant treatment effect on the depression level of the elderly aged 60–69, and can reduce the depression level of these elderly by 0.8 units.

The matching results by gender show that participation in friend-making, exercise, and recreational activities have significant treatment effects on the depression levels of male and female elderly, but there are large differences in the degree of treatment. Participation in friend-making activities can reduce the depression level of female elderly by about 0.7 units and male by about 0.35 units; participation in exercise activities can reduce the depression levels of female elderly by about 1.6 units and male by about 0.5 units; while participation in recreational activities can reduce the depression levels of female elderly by about 1.5 units and male by about 0.5 units. The treatment effect of friend-making, exercise, and recreational activities on female is stronger, indicating that social activities are more likely to reduce the risk of depression in female elderly, and effectively promote their mental health.

## Discussion

This study found that participation in friend-making, exercise, and recreational activities can significantly reduce the risk of depression in the elderly, which is consistent with previous research findings (12–14), indicating that active social participation can reduce the level of depression in the elderly. As China is a society of acquaintances, interaction with friends is the main form of social participation of the elderly. The interaction of the elderly with friends gives them their most important emotional sustenance besides care for their children, and their friendship network has a greater protective effect on the mental health of the elderly than their family network (34). However, participation in friend-making activities is mainly to communicate with friends, and its activity form is relatively single, so its positive effect is limited. Exercise is a powerful treatment method for the psychology and physiology of the elderly. Elderly people who participate in regular exercise tend to think that they are stronger, healthier, and more energetic, and so are more likely to adhere to continued participation in exercise. Physical strength and mental health form a virtuous circle. By participating in exercise activities, the elderly can also obtain more opportunities to interact with others, find like-minded friends, and obtain emotional support. Recreational activities based on the interests and hobbies of the elderly can enable them to release negative emotions to the greatest extent possible and enjoy a pleasant mood. At the same time, activities such as playing chess and cards often have the nature of a game, which can stimulate the desire of the elderly to win, give them an outlet for their wisdom and skills, and enable them to obtain happiness and a sense of achievement. In addition, compared with the male elderly, participation in friend-making, exercise, and recreational activities results in the female elderly having stronger resistance to the risk of depression, and some studies have found that positive associations of the frequency and autonomy of social participation with mental health are stronger in female elderly than male (35). The above results suggest that social participation has a more positive effect on mental health in female elderly than male. The reason may be that females tend to be better at expressing their emotions, so they are more likely to form intimate relationships and feel happy in the process of social participation. Therefore, in order to reduce the risk of depression in the elderly, it is essential to meet their need to participate in friend-making, exercise, recreational, and other interactive activities by putting in place settings and equipment suitable for the elderly.

Helping activities have no significant effect on the risk of depression in the elderly, which is in line with the previous study (12, 18). Some studies have found that there is a negative association between formal volunteer activity and depression among urban elderly, while there is a positive association between caring for a sick or disabled adult and depression among both urban and rural elderly (36). However, this is not a contradiction. In this study, participation in volunteer activities, free help, and providing care for others are all helping activities; free help and care for others account for more than 90% of helping activities, so it cannot be denied that participation in formal volunteer activities can reduce the risk of depression in the elderly. Chinese traditional ethics still attaches importance to the maintenance of the productivity of the elderly, and social morality encourages them to enhance their sense of value by taking care of their relatives (37). Therefore, in China, a country with close family relations, most elderly people engage in activities to take care of their relatives. Helping and caring for others requires the time, energy, and emotions of the elderly, but they can also obtain a sense of achievement and satisfaction through realizing their self-worth. Participation in economic activities has no effect on depression in the young elderly, but can increase the risk of depression in the elderly aged 70 and over, which is different from previous studies (20, 21). It shows that there are age differences in the impact of work on depression in the elderly. In this paper, 55% of the elderly respondents engage in a certain form of work in their old age. With an aging population, the working elderly will become an important part of the labor market, and the Chinese government is also intending to change policy to delay the retirement age. Since participation in economic activities in this study has no effect on depression in the young elderly, it also has a certain reference value for appropriately increasing the retirement age. At the same time, the elderly aged 70 and over may experience economic pressure and need to continue to work in their later years to maintain their livelihood, which is not conducive to their mental health. It is necessary to reduce the participation rate of economic activities of the elderly aged 70 and over by improving the level of social security. Helping others and working belong to the activities of creating social value, which is conducive to promoting social and economic development. Therefore, the elderly can be encouraged to realize their self-worth by creating social value, so as to improve their mental health.

In general, different types of social participation have different effects on depression in the elderly. This finding has strong guiding significance for active aging. The concept of “Active Aging” was officially put forward by the WHO at the Second World Assembly on Aging in 2002. It is an upgrade of the concept of healthy aging, emphasizing the health, social participation, and security of the elderly. At present, the health status and social security level of the elderly in China are still at a low level compared with developed countries, and cannot meet the participation needs of the elderly. For friend-making, exercise, and recreational activities, it is necessary to create a good participation environment for the elderly, provide diversified forms of participation, and encourage them to choose activities that are more conducive to their own health. For helping activities, the participation of Chinese elderly has no obvious positive effect on depression. The question arises as to whether this is related to the lack of health security and social support. The participation rate of the younger elderly in economic activities is relatively high, but their mental status cannot be guaranteed. A further question arises as to whether this is related to economic pressure, labor market restrictions, or discrimination. In order to enable the elderly to transform social value into self-worth through participation in helping and economic activities, improving the health and social security of the elderly is an important issue to be considered in China in the future.

This study had various strengths and limitations. The strength of the study is to explore the causal relationship between different types of social participation and depression in the elderly on the premise of eliminating sample selectivity bias. Compared with previous studies, the research results are more accurate, so as to provide a theoretical reference for improving the mental health of the elderly through social participation. Although we have achieved some research results through PSM, this paper still has some limitations. First, because there is no unified classification standard for social activities, this paper is more subjective in the classification of social participation, and the classification of social participation is not comprehensive enough due to data limitations. Second, different types of social participation have different effects on depression in the elderly, and there must be different influence mechanisms. This paper lacks in-depth analysis of the influence mechanism, which need to be further explored. Third, machine learning is widely used in the field of prediction and analysis (38–40), and this cutting-edge method has higher prediction accuracy. In this study, although logistic regression is applicable to PSM, it is more traditional. We will try to integrate cutting-edge methods such as machine learning into this research in the future.

## Conclusions

Most of the existing studies take social participation as a single index or only study a certain type of social participation to explore the causal relationship between social participation and depression in the elderly, and without controlling the sample selectivity bias, this causal relationship is controversial. Therefore, this paper classifies social participation, and based on the counterfactual framework, applied PSM to conduct an in-depth analysis of the causal relationship between participation in different types of social activities and depression in the elderly. First, in this study, the elderly have a higher probability of depression, and it is more common in female elderly. Future health system development in China should take targeted prevention and intervention measures for high-risk groups. Second, participation in friend-making, exercise and recreational activities can reduce the risk of depression in the elderly, and has a stronger resistance to depression in female elderly than male. It shows that it is effective to intervene the depression of the elderly, especially female, through social participation. In order to maximize the positive role of social participation, it is very necessary to further optimize public service facilities and create a good participation environment for the elderly. Third, participation in economic activities is only related to depression of the elderly aged 70 and over. On the one hand, this result can provide reference significance for appropriately extending the retirement age to meet the needs of the market labor force. On the other hand, it is necessary to reduce the economic participation rate of the elderly aged 70 and over by improving the level of social security.

In short, with the acceleration of the aging process, depression of the elderly has become an important topic in the field of public health, and social participation is an effective way to resist depression. This study aims to research the differences in the relationship between different types of social participation and depression in the elderly, which is of great significance to formulate a differentiated social support environment. In order to maximize the positive impact of different types of social participation on depression of the elderly, it is very necessary to optimize public service facilities, eliminate market employment restrictions and age discrimination, and improve the level of health and social security.

## Data Availability Statement

The datasets presented in this study can be found in online repositories. The names of the repository/repositories and accession number(s) can be found in the article/Supplementary Material.

## Ethics Statement

Ethical review and approval was not required for the study on human participants in accordance with the local legislation and institutional requirements. Written informed consent for participation was not required for this study in accordance with the national legislation and the institutional requirements.

## Author Contributions

XW and JG conceived the idea. JG drafted the manuscript. HLiu, HLi, and TZ participated in the discussion and put forward valuable opinions. HLiu, HLi, and TW revised and polished this article. All authors have contributed to the article and agree to submit this version.

## Conflict of Interest

The authors declare that the research was conducted in the absence of any commercial or financial relationships that could be construed as a potential conflict of interest.

## Publisher's Note

All claims expressed in this article are solely those of the authors and do not necessarily represent those of their affiliated organizations, or those of the publisher, the editors and the reviewers. Any product that may be evaluated in this article, or claim that may be made by its manufacturer, is not guaranteed or endorsed by the publisher.
