# Supplementary material for: Impact of Social Participation Types on Depression in the Elderly in China: An Analysis Based on Counterfactual Causal Inference
Source: Front Public Health. 2022 Apr 1;10:792765. doi: 10.3389/fpubh.2022.792765 (PMC9010741; doi:10.3389/fpubh.2022.792765)
Supplement: Supplementary file 1 [file Data_Sheet_1.ZIP › Supplementary Material/Research Data/CHARLS_2015_Questionnaire.pdf]

China Health and Retirement Longitudinal Study Followup Questionnaire 2015

中国健康与养老追踪调查 2015 年追访问卷

June 2015

China Center for Economic Research
Institute of Social Science Survey
Peking University

This page intentionally left blank

Contents 目 录

B DEMOGRAPHIC BACKGROUNDS 基本信息 . . . **1**

C FAMILY 家庭 . . . **13**

C1 RARENT, CHLIDREARING AND SIBLING INFORMATION 父母、子女、兄弟姐妹信息 . . . 13

CA PARENT INFORMATION 父母信息 . . . 13

CB CHILDREARING INFORMATION 子女信息 . . . 25

CC SIBLINGS 兄弟姐妹 . . . 31

C2 TIME TRANSFER AND TRANSFERS 家庭交往与经济帮助 . . . 37

CD TIME TRANSFER 与父母、子女间的交往 . . . 37

CE TRANSFERS 家庭得到及提供的经济帮助 . . . 40

CF TIME SPENT PROVIDING CARE 提供照料时间 . . . 46

A HOUSEHOLD MEMBER 家户成员 . . . 48

D HEALTH STATUS AND FUNCTIONING 健康状况和功能 . . . **51**

DA HEALTH STATUS 健康状况 . . . 52

PART I: GENERAL HEALTH STATUS AND DISEASE HISTORY 第一部分：一般健康状况和疾病史 . . . 52

PART II: LIFESTYLE AND HEALTH BEHAVIORS 第二部分：生活方式和健康行为 . . . 67

DB FUNCTIONAL LIMITATIONS AND HELPERS 身体功能障碍以及辅助者 74

DC COGNITION & DEPPRESSION 认知和抑郁 . . . 89

E HEALTH CARE AND INSURANCE 医疗保健与保险 . . . **97**

PART I MEDICAL INSURANCE 第一部分：医疗保险 . . . 97

PART II HEALTH CARE COSTS AND UTILIZATION 第二部分：医疗成本与使用情况 . . . 102

F WORK, RETIREMENT AND PENSION 工作、退休和养老金 . . . **121**

FA JOB STATUS 工作情况 . . . 123

FC AGRICULTURE WORK 农业工作 . . . 133

FARM EMPLOYED 农业打工 . . . 133

HOUSEHOLD AGRICULTURAL WORK 自家农业生产活动 . . . 133

FD EMPLOYED 受雇 . . . 136

FE QUESTIONS ABOUT LABOR SUPPLY 劳动力供给 142
FF QUESTIONS ABOUT WAGES 受雇工作的工资问题 142
FG Fringe Benefits 单位福利 145
FH NON-FARM SELF-EMPLOYED AND UNPAID FAMILY BUSINESS 非农自雇和为家庭经营活动帮工 145
FJ SIDE JOB (EMPLOYED OR SELF-EMPLOYED) 非主要职业（受雇或自雇） 149
FK UNEMPLOYMENT AND JOB SEARCH ACTIVITIES 失业，求职经历 . 149
FL LAST JOB 最近一份工作 151
FM RETIREMENT 退休与退职 156
FN PENSION INSURANCE 养老保险 166
Part 1 政府机关、事业单位养老保险（退休金）及企业职工基本养老保险 167
Part 2 政府机关、事业单位和企业职工基本养老保险（年金） 172
Part 3 新型农村养老保险、城乡居民养老保险及城镇居民养老保险 . . . 175
Part 4 征地养老保险（失地农民养老保险/被征地农民养老保险） 181
Part 5 高龄老人补贴 182
Part 6 人寿保险 183
Part 7 商业养老保险（人寿保险除外） 184
Part 8 其他养老保险 187

G&H INCOME, EXPENDITURES AND ASSETS 收入、支出与资产 191
G2 HOUSEHOLD INCOME AND EXPENDITURES 家户收入与支出 191
PART 1 Household Wage Income and Individual-based transfers 家户工资收入和个人获得的转移收入 191
PART 2 HOUSEHOLD AGRICULTURAL INCOME AND EXPENDITURE 家户农业收入与支出 195
PART 3 Self-employed Activities 个体经营或开办私营企业 198
PART 4 HOUSEHOLD PUBLIC TRANSFER INCOME 家户政府转移支付收入 199
PART 5 HOUSEHOLD LIVING EXPENDITURE 家户生活支出 199
HA HOUSEHOLD ASSETS 家户资产 202
PART 1 Current Residence 第一部分：现有住宅 202
PART 2 Other Residences 第二部分：其他房产 208
PART 3 Land 第三部分：土地 212

PART 4 Equipments, Consumption durables, and Valuables. 家用设备、耐用消费品和其他贵重物品 214
HB INDIVIDUAL ASSETS 个人资产 215
PART 1 Financial Assets 第一部分：金融资产 215
PART 2 DEBTS 第二部分：债务 220

I HOUSING CHARACTERISTICS 住房情况 **223**

This page intentionally left blank

B DEMOGRAPHIC BACKGROUNDS 基本信息

[**NOTE**: ONLY THOSE WHO WERE BORN BEFORE JULY 1, 1970 AND THEIR SPOUSES WILL BE INTERVIEWED **注意**：1970 年 7 月 1 日之前出生的人及其配偶需要做下列问卷]

Type of Interview R 受访者类型		
XRType = REIW	This is a reinterview R	受访者类型为回访受访者
XRType = NEWIW	This is a new interview R	受访者类型为新受访者

BA000_W2_1 We record your name is [name], is it right? 我们系统记录您的姓名是 [姓名]，对吗？

[**PROCEDURE**: Preload the name of R]

1. Yes 是 → Skip to BA000_W2_3 跳至 BA000_W2_3
2. No 否

BA000_W2_2 What's your name? 您的姓名是？ ___________

BA000_W2_3 Interviewer record R's gender. 访员记录受访者性别。

[**PROCEDURE**:Preload R's gender]

1. Male 男
2. Female 女

BA004_W3 What's your date of birth on ID card or Household register? 您身份证或户口本上登记的出生日期是？

1. _____ (**BA004_W3_1**) Year 年 _____ (**BA004_W3_2**) Month 月 _____ (**BA004_W3_3**) Day 日
2. Does not have Hukou or ID card 没有户口或者没有身份证

[IWER: The year must be a number in the range [1900 – 2015]. Mark the year using four digits. Take down the month as its actual number. For example, write January as "1" not "01", December as "12". If do not remember month and day, fill '0' 访员注意：年份填写范围 [1900 — 2015]。用 4 位数表示年，按照实际的月份填写月。例：1 月写作"1"，而不是"01"，12 月写作"12"。如果记不住月份和日期，请填入'0']

BA002 What's your actual date of birth? 您的真实的出生日期是？

1. The same as on Household or ID card 与户口本或身份证上的一样
2. _____ (**BA002_1**) Year 年 _____ (**BA002_2**) Month 月 _____ (**BA002_3**) Day 日

[IWER:The year must be a number in the range [1900 – 2015]. Mark the year using four digits. Take down the month as its actual number. For example, write January as "1" not "01", December as "12". If do not remember month and day, fill '0' 访员注意：年份填写范围 [1900 — 2015]。用 4 位数表示年，按照实际的月份填写月。例：1 月写作"1"，而不是"01"，12 月写作"12"。如果记不住月份和日期，请填入'0']

PROCEDURE 程序:
If the person does not know the date of birth, BA002 = .d or BA002 = .r, skip BA003.
如果受访者不知道出生日期即 BA002选择不知道或拒绝回答，跳过 BA003

BA003 Is your answer to BA002 based on the solar or the lunar calendar? 您刚才回答的是公历（阳历）还是农历（阴历）?
1. Solar calendar 公历（阳历）
2. Lunar calendar 农历（阴历）

[INTRO: Next are some questions about your birth place, some changes in your housing location, your Hukou and education 接下来我们将问到您的出生地、居住地的变化情况，以及您的户口和受教育程度]

PROCEDURE 程序:
If XRType = REIW, then preload the information of address in last wave, if missing, then ask BB000_W3. 如果这是一个回访受访者，加载上一期的地址信息, 如果上一期的地址信息缺失，回答 BB000_W3
If XRType = NEWIW, go to BB001_W3. 如果这是一个新的受访者，跳至 BB001_W3

BB000_W3 What's your address in [the time of last interview]? [加载上次访问时间] 时，您住在哪里?
1. [Address in last interview] 上期访问时的常住地址
2. Other 其他地方:
   _____ (**BB000_W3_a_1**) province/city/county 省/市/县
   _____ (**BB000_W3_a_2**) township 乡/镇/街道
   _____ (**BB000_W3_a_3**) village/neighborhood 村/社区
3. Abroad 国外

[IWER: Confirm the permanent address with respondents, and fill in the changed. If do not know the name, please fill in "other" 访员注意：请与受访者确认常住地址，并且记录变化的地址。如果不知道村、市、省的具体名称，请填入"其它"]

[IWER: county/city includes county-level administrative units/county-level city/district 访员注意：县/市包括县级行政单位/县级市/区]

BB000_W3_1 What is the type of the address? 居住地址的类型是?
1. Family housing 家庭住宅
2. Nursing home 养老院或其他养老机构
3. Hospital 医院
4. Other, please specify 其他，请注明 _____ （**BB000_W3_1_1**）

BB000_W3_2 Was it village or city/town? 是农村还是城市?
1. Main city zone 主城区

2. Combination zone between urban and rural areas 城乡结合区
3. The town center 镇中心区
4. ZhenXiang area 镇乡结合区
5. special area 特殊区域
6. Township central 乡中心区
7. Village 村庄

BB001_W3 What's your address now? 您现在一般住在哪里?

1. [Preload address] 加载上期访问时的常住地址
2. Other 其他地方:
   _____ (**BB001_W3_a_1**) province/city/county 省/市/县
   _____ (**BB001_W3_a_2**) township 乡/镇/街道
   _____ (**BB001_W3_a_3**) village/neighborhood 村/社区
3. Abroad 国外

[IWER: Confirm the permanent address with respondents, and fill in the changed. If do not know the name, please fill in "other" 访员注意：请与受访者确认常住地址，并且记录变化的地址。如果不知道村、市、省的具体名称，请填入"其它"]

[IWER: county/city includes county-level administrative units/county-level city/district 访员注意：县/市包括县级行政单位/县级市/区]

BB001_W3_1 What is the type of the address? 居住地址的类型是?

1. Family housing 家庭住宅
2. Nursing home 养老院或其他养老机构
3. Hospital 医院
4. Other, please specify 其他，请注明 _____ （**BB001_W3_1_1**）

BB001_W3_2 Was it village or city/town? 是农村还是城市?

1. Main city zone 主城区
2. Combination zone between urban and rural areas 城乡结合区
3. The town center 镇中心区
4. ZhenXiang area 镇乡结合区
5. special area 特殊区域
6. Township central 乡中心区
7. Village 村庄

PROCEDURE 程序:

If BB001_W3 ! = 1 or XRType = NEWIW, then skip to BB003. Otherwise, skip to BB005_W3_1. 如果 BB001_W3 ! = 1 或者 XRType = NEWIW, 跳至 BB003. 否则，跳至 BB005_W3_1

BB003 When did you first live in permanent address's county/city/district? 您第一次搬到常住地所在县/市/区是在什么时候? _____ 1900..2015 Year 年
[IWER: Mark the year using four digits 访员注意：请用 4 位数表示年]

BB004 When you first moved to permanent address, did you live in the same village/community as you currently do? 您第一次搬到常住地，就是住在当前村/社区吗?
1. Yes 是 → skip to BB005_W3_1 跳至 BB005_W3_1
2. No 否

BB005 In what year did you first live in permanent address's village/community? 您第一次搬来常住地所在村/社区居住是在什么时候? _____1900..2015 Year 年
[IWER: Mark the year using four digits 访员注意：请用 4 位数表示年]

BB005_W3_1 Would you change your address in next two years? 接下来的 2 年里, 您会搬家吗?
1. Yes 是
2. No 否 → skip to BB001 跳至 BB001

BB005_W3_2 Where would your new address? 您的新家地址会是?
1. I am not sure 我不知道
2. Another house in the same village/community 同一个村/社区, 不同的房子
3. Other 其他地方:
   _____ (**BB005_W3_2_1**) province/city/county 省/市/县
   _____ (**BB005_W3_2_2**) township 乡/镇/街道
   _____ (**BB005_W3_2_3**) village/neighborhood 村/社区

BB001 Where were you born? 您的出生地是哪里?
1. The same as permanent address 和常住地一样
2. Another village/neighborhood in permanent address' s county/city/district 常住地所在县/市/区的其他村/社区
   _____ (**BB001_1**) township 乡/镇/街道
   _____ (**BB001_2**) village/neighborhood 村/社区
3. Other 其它:
   _____ (**BB001_3**) province_city_county/city/district 省 _ 市 _ 县/市/区
   _____ (**BB001_4**) township 乡/镇/街道
   _____ (**BB001_5**) village/neighborhood 村/社区
4. Abroad 国外

[IWER: county/city includes county-level administrative units/county-level city/district 访员注意：县/市包括县级行政单位/县级市/区]

PROCEDURE 程序:
If ZBC001 = *nonresponse*, answer BC002_W3_1 & BC002_W3_4, then skip to BD001_W2_4. 如果 ZBC001 = *nonresponse*, 回答 BC002_W3_1 和 BC002_W3_4后, 跳至 BD001_W2_4

BC001_W3_1 We record your hukou type is [ZHukou Type], right? 我们记录您上一次访问时的户口类型是 [户口类型]，对吗？

1. Yes 是 → SKIP TO BC001_W3_3 跳至 BC001_W3_3
2. No 否

BC001_W3_2 What's your hukou type in last interview? 你上次的户口类型是什么？

1. Agricultural Hukou 农业
2. Non-agricultural Hukou 非农业
3. Unified Residence Hukou 统一居民户
4. Do not have Hukou 没有户口

BC001_W3_3 What's your Hukou location in last interview? 你上次的户口所在地是什么？[访员注意：如果不知道具体名称，请填入"其他"]

1. The same as permanent address 和常住地一样
2. Other: 其他：
   _____ (**BC001_W3_3_1**) province_city_county/city/district 省 _ 市 _ 县/市/区
   _____ (**BC001_W3_3_2**) township 乡/镇/街道
   _____ (**BC001_W3_3_3**) village/neighborhood 村/社区
3. 国外

BC002_W3 Since [ZIWTime], have you Hukou type and Hukou location changed? 自从 [ZIWTime] 以来，您的户口类型和户口所在地有没有发生变化？

1. Only Hukou type has changed 户口类型变了
2. Only Hukou place has changed 户口所在地变了
3. Both Hukou type and place have changed 户口类型和户口所在地都变了
4. Both haven't changed 都没变

PROCEDURE 程序:

If BC002_W3 = 1 or = 3, then answer BC002_W3_1 - BC002_W3_3. 如果 BC002_W3 = 1 或者 = 3，则回答 BC002_W3_1 至 BC002_W3_3

BC002_W3_1 What is your current Hukou status? 您这次的户口类型是？

1. Agricultural Hukou 农业
2. Non-agricultural Hukou 非农业
3. Unified Residence Hukou 统一居民户口
4. Do not have Hukou 没有户口

BC002_W3_2 What is the reason that you changed to the agricultural Hukou? 您的户口类型发生变化的原因是什么？

1. Go to school 上学
2. Marriage 结婚
3. Employment 就业
4. Land is acquired by the government 土地被征用

5. Migration of the whole village 整村搬迁
6. 其他: _____ (**BC002_W3_2_1**)

BC002_W3_3 When did your type of Hukou change? 您的户口类型发生变化的时间是?
_____ (**BC002_W3_3_1**) Year 年 _____ (**BC002_W3_3_2**) Month 月

PROCEDURE 程序:
If BC002_W3 = 2 or = 3, then answer BC002_W3_4 - BC002_W3_6. 如果 BC002_W3 = 2 或者 = 3, 则回答 BB005_w3_4 至 BC002_W3_6

BC002_W3_4 What's your current location of Hukou? 您现在的户口所在地是?
1. Permanent address 常住地
2. Other 其他:
   _____ (**BC002_W3_4_1**) province_city_county/city/district 省 _ 市 _ 县/市/区
   _____ (**BC002_W3_4_2**) township 乡/镇/街道
   _____ (**BC002_W3_4_3**) village/neighborhood 村/社区
3. Abroad 国外

BC002_W3_5 Why did your location of Hukou change? 您的户口所在地发生变化的原因是什么?
1. Go to school 上学
2. Marriage 结婚
3. Employment 就业
4. Land is acquired by the government 土地被征用
5. Migration of the whole village 整村搬迁

BC002_W3_6 When did your location of Hukou change? 您的户口所在地发生变化的时间是?
_____ (**BC002_W3_6_1**) Year 年 _____ (**BC002_W3_6_2**) Month 月

BD001_W2_4 Have your highest level of education changed from last wave? If so, what's the highest level of education your have attained now?(not including adult education) 您现在获得的最高教育水平是什么(不包括成人教育)?
1. No formal education (illiterate) 未受过教育(文盲)
2. Did not finish primary school 未读完小学
3. Sishu/home school 私塾毕业
4. Elementary school 小学毕业
5. Middle school 初中毕业
6. High school 高中毕业
7. Vocational school 中专(包括中等师范、职高)毕业
8. Two-/Three-Year College/Associate degree 大专毕业
9. Four-Year College/Bachelor's degree 本科毕业
10. Master's degree 硕士毕业
11. Doctoral degree/Ph.D. 博士毕业

12. No changing 没有变化

PROCEDURE 程序:

If BD001_W2_4 = 1/2/3 , ask BD001_W3_1, otherwise ask BD002_W3。如果 BD001_W2_4 = 1/2/3，询问 BD001_W3_1，否则询问 BD002_W3

BD001_W3_1 Are you literate? 您是否识字？

1. Yes 是
2. No 否

BD002_W3 How many years at school after your highest level of education changed from last wave? 毕业以后又读了几年书？（毕业后没再读书的填 0） _____

BD006 At what age did you finish schooling? 您几岁读完书？ _____1...120 years old 岁

[IWER: It asks age when R finishes schooling, not age when R finished elementary school 访员注意：此处询问的是停止上学的年龄，并非读完小学/私塾的年龄]

BD007_W2_1 Have you attended school for adult education? 您参加过成人教育（如电大，夜校，自考，函授，扫盲班，速成班）吗？（可多选）

1. None 没有
2. TV University 电大
3. Night School 夜校
4. Zikao (examinations for self-taught students) 自考
5. Hanshou/Correspondence course/Distance learning 函授
6. Literacy course 扫盲班
7. Accelerated education course 速成班
8. Other (explain:) 其他，请注明_____(**BD007_W2_1_1**)

BD008_W2_1 How many years did you spend in adult education? 您成人教育一共读了几年？ _____ year 年

BD009_W2_1 Did you get a diploma or degree from the adult education program you attended? 您有从成人教育中获得了学位或文凭吗？

1. Yes 是
2. No 否 → 跳至 BD012

BD010 When did you receive the diploma? 您什么时候获得该学位或者文凭？ _____ 1900...2015 year 年

BD011 What is the highest adult schooling degree? 您获得最高的成人教育学位或文凭是什么？

1. Vocational school 中专
2. Two/Three Year College / Associate degree 大专
3. Four Year College / Bachelor's degree 本科

4. Others 其他

BD012 Since [ZIWTime], have you participated in vocational and technical training? 自从 [ZIWTime] 以来，您参加过职业技术培训吗？

1. Yes 是
2. No 否 → to 跳至 BE001

BD012 How many times have your participated? 您接收过几次职业培训？ _____ Times 次

BD012 Since [ZIWTime], how many years have you participated? 自从 [ZIWTime] 以来，您职业技术培训一共参加了几年？ _____ Years 年

[Show Card 出示卡片 1]

BE001 RMaritalStatus: What is your marital status? 您目前的婚姻状态？
[IWER: common-law marriage is considered as married 即使没有领结婚证但自称结婚者也可视为已婚]

1. Married with spouse present 已婚与配偶一同居住
2. Married but not living with spouse temporarily for reasons such as work 已婚，但因为工作等原因暂时没有跟配偶在一起居住
3. Separated 分居（不再作为配偶共同生活）
4. Divorced 离异
5. Widowed 丧偶
6. Never married 从未结婚
7. Cohabitated 同居

PROCEDURE 程序:
If BE001 ! = 6 和 XRType = 2, ask BE003_W2_1 to BE009。如果 BE001 ! = 6 和 XRType = 2，询问 BE003_W2_1 至 BE009

[INTRO: Many people have more than one marriage through whole life. Please bear me a few more questions on this 很多人一生中不止结过一次婚，所以，我们想请您回答以下这些婚姻题目]

BE003_W2_1 How many times have you been married since last interview? 您结过几次婚？ _____ times 次

PROCEDURE 程序:
If BE003_W2_1 > 0, ask BE009. 如果 BE003_W2_1 > 0，询问 BE009

BE009 When was your most recent marriage? 你最近一次是什么时候结的婚？
_____ 1900...2015 (**BE009_1**) year 年 _____ 0...12 (**BE009_2**) month 月
[访员注意: 用 4 位数表示年，按照实际的月份填写月。例：1 月写作“1”，而不是“01”，12 月写作“12”。如果记不住月份，请填入“0”]

PROCEDURE 程序:

If HHseperated = 0, skip to BF008. 如果 HHseperated = 0， 跳至 BF008
If BE001 = 1/2/6/7, skip to BF008. 如果 BE001 = 1/2/6/7， 跳至 BF008
If ZBF001 = *nonresponse*, answer BF001. 如果 ZBF001 = *nonresponse*， 回答BF001
If ZBF002_1 = *nonresponse*, answer BF002. 如果 ZBF002_1 = *nonresponse*， 回答BF002
If BF002_2 ! = *nonresponse*, answer BF003. 如果 BF002_2 ! = *nonresponse*， 回答BF003

[IWER: I'd like to ask you a few questions about your current (or most recent) spouse/partner 访员注意：这里有几个关于受访者配偶的问题。若受访者属于结婚多次，则问最近一次婚姻的配偶]

[INTRO: An important part of this study is understanding how people make decisions during different stages of life, we also need to a general age range for your spouse 引语：了解人们在不同的人生阶段如何进行决策是我们研究的一个重要方面，因此接下来我们会问到您配偶的年龄]

BF001 What is your spouse/partner's Chinese Zodiac sign? 您配偶的属相是？
[访员注意：这里有几个关于受访者配偶的问题。若受访者属于结婚多次，则问最近一次婚姻的配偶] [Show Card 出示卡片 1] 您配偶的属相是？

1. 鼠
2. 牛
3. 虎
4. 兔
5. 龙
6. 蛇
7. 马
8. 羊
9. 猴
10. 鸡
11. 狗
12. 猪

BF002 When was your spouse/partner born? 您配偶的出生日期是？
[访员注意：用 4 位数表示年，按照实际的月份填写月。例：1 月写作"1"，而不是"01"，12 月写作"12"。如果记不住月份，请填入"0"] _____ 1900...2015 (**BF002_1**) year 年 _____ 0...12 (**BF002_2**) month 月

BF003 Soloar calendar or lunar calendar? 您刚才答的您配偶的生日是公历（阳历）还是农历（阴历）？

1. Solar calendar 公历（阳历）
2. Lunar calendar 农历（阴历）

BF004 What is the highest level of education your spouse/partner has attained? (not including adult education) 您配偶获得的最高学历是？（不包括成人教育）

1. No formal education (illiterate) 未受过教育（文盲）
2. Did not finish primary school but capable of reading and/or writing 未读完小学，但能够读、写
3. Sishu/home school 私塾毕业
4. Elementary school 小学毕业

5. Middle school 初中毕业
6. High school 高中毕业
7. Vocational school 中专（包括中等师范、职高）毕业
8. Two-/Three-Year College/Associate degree 大专毕业
9. Four-Year College/Bachelor's degree 本科毕业
10. Master's degree 硕士毕业
11. Doctoral degree/Ph.D. 博士毕业

BF004_W3_1 Are your spouse literate? 您配偶是否识字？
1. Yes 是
2. No 否

PROCEDURE 程序:
If BE001 = 3, ask BF005. 如果 BE001 = 3, 询问 BF005

BF005 When did you separate? 你们什么时候分居的？
[IWER: Mark the year using four digits. Take down the month as its actual number. For example, write January as "1" not "01", December as "12". If do not remember month and day, fill '0' 访员注意：用 4 位数表示年，按照实际的月份填写月。例：1 月写作"1"，而不是"01" ,12 月写作"12"。如果记不住月份和日期，请填入'0'] _____ 1900...2015 (**BF005_1**) year 年 0...12 (**BF005_2**) month 月

PROCEDURE 程序:
If BE001 = 4, ask BF006. 如果 BE001 = 4, 询问 BF006

BF006 When did you divorce? 你们什么时候离婚的？
[IWER: Mark the year using four digits. Take down the month as its actual number. For example, write January as "1" not "01", December as "12". If do not remember month and day, fill '0' 访员注意：用 4 位数表示年，按照实际的月份填写月。例：1 月写作"1"，而不是"01" ,12 月写作"12"。如果记不住月份和日期，请填入'0'] _____ 1900...2015 (**BF006_1**) year 年 _____ 0...12 (**BF006_2**) month 月

BF006_W2_1 What is the reason of your divorce? 您离婚的原因是？
1. Emotional feud 感情不和
2. Live in different places 两地分居
3. In order to facilitate the property purchase transactions 为了便利不动产等购买交易
4. Other 其他原因，请注明_____(**BF006_W2_1_1**)

BF006_W2_2 Divided assets, including real estate, how much is yours and how much is your ex-wife/husband? 关于双方共同财产（包括不动产、汽车等）分割情况，以货币价值计算，你得到_____ (**BF006_W2_2_1**) 元，前夫/妻得到_____ (**BF006_W2_2_2**) 元

BF006_W2_3 Do you have any infancy children? Owed to whom? 有未成年的孩子吗？归谁抚养？

1. Myself 我自己
2. My last spouse 我前夫/妻
3. None 没有未成年子女 → Skip to BF007

BF006_W2_4 How much money you or your ex-wife/husband pay for upbringing the children? 你或者你的前夫/前妻付给抚养孩子一方多少抚养费? _____ Yuan 元

**PROCEDURE 程序:**
If BE001 = 5, Ask BF007 如果 BE001 = 5, 询问 BF007

BF007 When did your spouse pass away? 您配偶是什么时候去世的?

_____ 1900...2015 (**BF007_1**) year 年 0...12 (**BF007_2**) month 月 [IWER: Mark the year using four digits. Take down the month as its actual number. For example, write January as "1" not "01", December as "12". If do not remember month and day, fill '0' 访员注意: 用 4 位数表示年, 按照实际的月份填写月。例: 1 月写作"1", 而不是"01" ,12 月写作"12"。如果记不住月份和日期, 请填入 '0']

BF008 How often did the respondent receive assistance in answering section Demographics? 受访者填写该部分问卷时是否求助?

[IWER: If it is answered by a proxy, please record the respondent's reaction 访员注意: 如果是协助回答, 请记录受访者的反应]

1. Never 从未 → Skip to next module 跳至下一模块
2. A few times 偶尔几次 → Skip to next module 跳至下一模块
3. Most or all of the time 大多数 → Skip to next module 跳至下一模块
4. The section was completed by a proxy respondent 完全请人代填

BF009 What is your relationship to R? 您和受访者是什么关系?

[IWER: What is the proxy's relationship to R? If unknown, please ask the proxy 访员注意: 代填问卷的人和受访者是什么关系。如果说不清楚, 请问代填者]

1. Spouse 配偶
2. Mother 母亲
3. Father 父亲
4. Mother-in-law 岳母/婆婆
5. Father-in-law 岳父/公公
6. Sibling 兄弟姐妹
7. Brother-in-law, sister-in-law 姐夫妹夫/嫂子弟媳
8. Child 孩子
9. Spouse of child 孩子的配偶
10. Grandchild 孙子女
11. Other relative 其他亲戚
12. Helper or other non-relative 帮忙的人或者其他非亲属
13. Others 其他

BF010 What is the main reason for proxy (the respondent is absent)? 受访者不在场，请人代填的主要原因是什么？

1. The respondent has serious physical handicaps 受访者有严重身体障碍
2. The respondent has serious mental handicaps 受访者有严重精神障碍
3. The respondent has rejected this interview 受访者拒访
4. Other 其他，请注明_____(**BF010_1**)

C FAMILY 家庭

Type of Interview R 受访者类型		
XRType = REIW	This is a reinterview R	受访者类型为回访受访者
XRType = NEWIW	This is a new interview R	受访者类型为新受访者

[IWER: Section CA and Section CC and Section CG and Section A are asked for each Rs and Section CB is only asked for Family R. If Family R is not in or couldn't answer the questions, spouse can be a proxy, but other people are not allowed. 访员注意：CA/CC/CG/A 部分需要询问每个受访者，CB，CD，CE 和 CF 部分只需要询问家庭受访者。如果家庭受访者不在家或者无法回答，可以由其配偶来替代回答，但除配偶以外的其他人不可以回答。]

[Section CA and section CC are asked for Family R first, then based on CC005_W3_1, ask questions about spouse's parents and siblings. Parents of Family R are encoded as: 1 biological father, 2 biological mother, 3 stepfather, 4 stepmother, 5 adopted father, 6 adopted mother. Parents of spouse are encoded as: 7 biological father, 8 biological mother, 9 stepfather, 10 stepmother, 11 adopted father, 12 adopted mother. Answers of spouse's siblings' information are recorded with variables with postfix S. 首先询问受访者 CA 和 CC 部分，然后根据 CC005_W3_1，询问配偶的父母与兄弟姐妹信息。受访者父母编码为：1 亲生父亲，2 亲生母亲，3 继父，4 继母，5 养父，6 养母。配偶父母编码为：7 亲生父亲，8 亲生母亲，9 继父，10 继母，11 养父，12 养母。记录配偶的兄弟姐妹信息的变量加后缀 S。]

C1 RARENT, CHLIDREARING AND SIBLING INFORMATION 父母、子女、兄弟姐妹信息

[In the following three parts: CA parent information, CB childrearing information and CC sibling information, I'd like to ask you some questions about your family. 接下来的三部分：CA 父母、CB 子女、CC 兄弟姐妹信息，我们想问您一些有关您这些家人的问题。]

CA PARENT INFORMATION 父母信息

[I'd like to ask you some questions about your parents. 首先我想问您一些关于您父母的问题。]

PROCEDURE 程序：

If this is a REIW, ask the following questions; if this is a NEWIW, then skip to CA000_W3_1. 如果这是一个回访受访者，回答以下问题；如果这是一个基线受访者，跳至 CA000_W3_1。

PROCEDURE 程序：

If ZCA001_1_ $\neq 1$, skip to procedure before CA001_W3_0

PROCEDURE 程序：

If ZCA002_inf_1_ is missing, skip CA000_W3_0

CA000_W3_0 Is [Name] your biological father? 您的亲生父亲是【姓名】吗?

1. Yes 对 → Skip to CA000_W3_2 跳至 CA000_W3_2
2. The name is wrong, and other information is right 名字不对，其他信息都对 → Skip to CA000_W3_5_1 跳至 CA000_W3_5_1
3. The name and other information are wrong 名字和其他信息都不对，上次问错了人

[Preload fathers' names. 加载父亲姓名。]

CA000_W3_5 What's the name of your biological father? 您亲生父亲的姓名是?

1. Name 姓名
3. Name 姓名
5. Name 姓名
7. Name 姓名
9. Name 姓名
11. Name 姓名
97. Other 其它

PROCEDURE 程序:
If CA000_W3_5 $\neq 97$, skip to CA000_W3_2

CA000_W3_5_1 What' s the name of your biological father? 您亲生父亲的姓名是? ______

PROCEDURE 程序:
If CA000_W3_0 $= 2$, skip to CA000_W3_2.

CA000_W3_1 What was your biological father's birth year? 您的亲生父亲是哪一年出生的?
____1850...2014
[IWER:Mark the year using four digits. 访员注意：用 4 位数表示年。]

CA000_W3_2 Is your biological father still living? 您父亲还健在吗?

1. Yes 是
2. No 否

PROCEDURE 程序:
If this is a REIW and CA000_W3_2 $= 1$, skip to procedure before CA001_W3_0; if this is a NEWIW and CA000_W3_2 $= 1$, skip to CA000_W3_5_1 and then skip to procedure before CA001_W3_0. 如果这是一个回访受访者并且 CA000_W3_2 $= 1$，跳至 CA001_W3_0 之前程序；如果这是一个基线受访者并且 CA000_W3_2 $= 1$，跳至 CA000_W3_5_1，之后跳至 CA001_W3_0 之前程序。

CA000_W3_3 When did he pass away? 他是什么时候去世的?

1. Year 年份 ____1850...2014 (**CA000_W3_3_1**)
2. Age 年龄 ___0...120 (**CA000_W3_3_2**)

[IWER:Mark the year using four digits. 访员注意：用 4 位数表示年。]

CA000_W3_4 What was the main cause of your biological father's death? 您亲生父亲去世的主要原因是什么？[访员注意：如果是因疾病死亡，请详细写明病因，例如因癌症去世，要注明是哪种类型的癌症（胃癌、肺癌等）；如果因传染病去世，要写明传染病的类型（结核病、痢疾）；如果是因为事故死亡，要写明事故类型，如车祸、火灾、意外中毒等。]

**PROCEDURE 程序：** If this is a REIW, ask the following questions; if this is a NEWIW, then skip to CA001_W3_1. 如果这是一个回访受访者，回答以下问题；如果这是一个基线受访者，跳至CA001_W3_1。

**PROCEDURE 程序：** If ZCA001_2_ $\neq 1$, skip to procedure before CA002_W3_1.

**PROCEDURE 程序：** If ZCA002_inf[2] is missing, skip CA001_W3_0

CA001_W3_0 Is your biological mother [Name]? 您的亲生母亲是【姓名】吗？

1. Yes 对 → Skip to CA001_W3_2 跳至CA001_W3_2
2. The name is wrong, and other information is right 名字不对，其他信息都对 → Skip to CA001_W3_5_1 跳至CA001_W3_5_1
3. The name and other information are wrong 名字和其他信息都不对，上次问错了人

[Preload mothers' names. 加载母亲姓名。]

CA001_W3_5 What's the name of your biological mother? 您亲生母亲的姓名是？

2. Name 姓名
4. Name 姓名
6. Name 姓名
8. Name 姓名
10. Name 姓名
12. Name 姓名
97. Other 其它

**PROCEDURE 程序：** If CA001_W3_5 $\neq 97$, skip to CA001_W3_2.

CA001_W3_5_1 What's the name of your biological mother? 您亲生母亲的姓名是？ ______

**PROCEDURE 程序：** If CA001_W3_0 $= 2$, skip to CA001_W3_2.

CA001_W3_1 What was your biological mother's birth year? 您的亲生母亲是哪一年出生的？

____1850...2014

[IWER:Mark the year using four digits. 访员注意：用 4 位数表示年。]

CA001_W3_2 Is your biological mother still living? 您母亲还健在吗？

1. Yes 是
2. No 否

PROCEDURE 程序:
If this is a REIW and CA001_W3_2 = 1, skip to procedure before CA002_W3_1; if this is a NEWIW and CA001_W3_2 = 1, skip to CA001_W3_5_1 and then skip to procedure before CA002_W3_1. 如果这是一个回访受访者并且 CA001_W3_2 = 1，跳至 CA002_W3_1 之前程序；如果这是一个基线受访者并且 CA001_W3_2 = 1，跳至 CA001_W3_5_1，之后跳至 CA002_W3_1 之前程序。

CA001_W3_3 In which year did she pass away? 她是哪一年去世的?____1850...2014 (**CA001_W3_3_1**)
Or how old was she then? 或者去世的时候多少岁？ ___0...120 (**CA001_W3_3_2**)
[IWER:Mark the year using four digits. 访员注意：用 4 位数表示年。]

CA001_W3_4 What was the main cause of your biological mother's death? 您亲生母亲去世的主要原因是什么？[访员注意：如果是因疾病死亡，请详细写明病因，例如因癌症去世，要注明是哪种类型的癌症（胃癌、肺癌等）；如果因传染病去世，要写明传染病的类型（结核病、痢疾）；如果是因为事故死亡，要写明事故类型，如车祸、火灾、意外中毒等。]

PROCEDURE 程序:
If CA000_W3_2 = 1 and CA001_W3_2 = 1, ask CA002_W3_1. 如果CA000_W3_2 = 1 且 CA001_W3_2 = 1，请询问CA002_W3_1。

CA002_W3_1 Had your biological parents divorced? 您的亲生父母有没有离过婚？（注意：因为感情问题长久分居也算离婚；离婚又复婚的不算离婚；父母一方去世的属于丧偶不属于离婚。）

1. Yes 有过
2. No 没有 → Skip to CA006_W3_1

PROCEDURE 程序:
If CA000_W3_2 = 2 or CA002_W3_1 = 1, ask CA004_W3_1; otherwise, skip to procedure before CA005_W3_1. 如果CA000_W3_2 = 2 或者 CA002_W3_1 = 1，请询问CA004_W3_1；否则，跳至 CA005_W3_1 之前程序。

CA004_W3_1 Have you had a stepfather? 您有继父吗？

1. Yes 有
2. No 没有 → Skip to procedure before CA005_W3_1 跳至CA005_W3_1 之前程序

CA004_W3_2 Is your step father living? 您的继父还健在吗？
[IWER: If the respondent has two and more stepfathers, we ask the last one. 访员注意：如果受访者有两个及以上的继父，我们询问最后一个。]

1. Yes 是
2. No 否 → Skip to procedure before CA005_W3_1 跳至CA005_W3_1 之前程序

PROCEDURE 程序:
If ZCA002_inf_3_ is missing, skip CA004_W3_0.

CA004_W3_0 Is your stepfather [Name]? 您的继父是【姓名】吗?
1. Yes 对 → Skip to procedure before CA005_W3_1 跳至 CA005_W3_1 之前程序
2. The name is wrong, and other information is right 名字不对，其他信息都对 → Skip to CA004_W3_4_1 跳至 CA004_W3_4_1
3. The name and other information are wrong 名字和其他信息都不对，上次问错了人

[Preload fathers' names. 加载父亲姓名。]

CA004_W3_4 What's the name of your stepfather? 您继父的姓名是?
1. Name 姓名
3. Name 姓名
5. Name 姓名
7. Name 姓名
9. Name 姓名
11. Name 姓名
97. Other 其它

PROCEDURE 程序:
If CA004_W3_4 $\neq$ 97, skip to procedure before CA005_W3_1

CA004_W3_4_1 What's the name of your stepfather? 您的继父的姓名是? ________

PROCEDURE 程序:
If CA004_W3_0 $=$ 2, skip to procedure before CA005_W3_1.

CA004_W3_3 What was your stepfather's birth year? 您的继父是哪一年出生的?____1850...2014
[IWER:Mark the year using four digits. 访员注意：用 4 位数表示年。]

PROCEDURE 程序:
If CA001_W3_2 $=$ 2 or CA002_W3_1 $=$ 1, ask CA005_W3_1. 如果 CA001_W3_2 $=$ 2 或者 CA002_W3_1 $=$ 1，请询问 CA005_W3_1。

CA005_W3_1 Have you had a stepmother? 您有继母吗?
1. Yes 有
2. No 没有 → Skip to CA006_W3_1 跳至 CA006_W3_1

CA005_W3_2 Is your stepmother living? 您的继母还健在吗?
[IWER: If the respondent has two and more stepmothers, we ask the last one. 访员注意：如果受访者有两个及以上的继母，我们询问最后一个。]
1. Yes 是
2. No 否 Skip to CA006_W3_1 跳至 CA006_W3_1

PROCEDURE 程序:
If ZCA002_inf_4_ is missing, skip CA005_W3_0.

CA005_W3_0 Is your stepmother [Name]? 您的继母是【姓名】吗?
1. Yes 对 → Skip to CA006_W3_1 跳至 CA006_W3_1
2. The name is wrong, and other information is right 名字不对，其他信息都对 → Skip to CA005_W3_4_1 跳至 CA005_W3_4_1
3. The name and other information are wrong 名字和其他信息都不对，上次问错了人

[Preload mothers' names. 加载母亲姓名。]

CA005_W3_4 What's the name of your stepmother? 您继母的姓名是?
2. Name 姓名
4. Name 姓名
6. Name 姓名
8. Name 姓名
10. Name 姓名
12. Name 姓名
97. Other 其它

PROCEDURE 程序:
If CA005_W3_4 $\neq 97$, skip to CA006_W3_1.

CA005_W3_4_1 What's the name of your stepmother? 您的继母的姓名是? ________

PROCEDURE 程序:
If CA005_W3_0 $= 2$, skip to CA006_W3_1.

CA005_W3_3 What was your stepmother's birth year? 您的继母是哪一年出生的? ____1850...2014
[IWER:Mark the year using four digits. 访员注意：用 4 位数表示年。]

CA006_W3_1 Have you had an adopted father? 您是否有养父?
1. Yes 是
2. No 否 → Skip to CA007_W3_1 跳至CA007_W3_1

CA006_W3_2 Is your adopted father living? 您的养父还健在吗?
1. Yes 是
2. No 否 → Skip to CA007_W3_1 跳至CA007_W3_1

PROCEDURE 程序:
If ZCA002_inf_5_ is missing, skip CA005_W3_0.

CA006_W3_0 Is your adopted father [Name]? 您的养父是【姓名】吗?
1. Yes 对 → Skip to CA007_W3_1 跳至 CA007_W3_1

2. The name is wrong, and other information is right 名字不对，其他信息都对 → Skip to CA006_W3_4_1 跳至 CA006_W3_4_1
3. The name and other information are wrong 名字和其他信息都不对，上次问错了人

[Preload fathers' names. 加载父亲姓名。]

CA006_W3_4 What's the name of your adopted father? 您养父的姓名是？

1. Name 姓名
3. Name 姓名
5. Name 姓名
7. Name 姓名
9. Name 姓名
11. Name 姓名
97. Other 其它

**PROCEDURE 程序：**
If CA006_W3_4 $\neq 97$, skip to CA007_W3_1.

CA006_W3_4_1 What's the name of your adopted father? 您的养父的姓名是？ ________

**PROCEDURE 程序：**
If CA006_W3_0 $= 2$, skip to CA007_W3_1.

CA006_W3_3 What was your adopted father's birth year? 您的养父是哪一年出生的？____1850...2014

[IWER:Mark the year using four digits. 访员注意：用 4 位数表示年。]

CA007_W3_1 Have you had an adopted mother? 您是否有养母？

1. Yes 是
2. No 否 → Skip to procedure before CA007_W3_5 跳至CA007_W3_5之前的程序

CA007_W3_2 Is your adopted mother living? 您的养母还健在吗？

1. Yes 是
2. No 否 → Skip to procedure before CA007_W3_5 跳至CA007_W3_5之前的程序

**PROCEDURE 程序：**
If ZCA002_inf_6_ is missing, skip CA007_W3_0.

CA007_W3_0 Is your adopted mother [Name]? 您的养母是【姓名】吗？

1. Yes 对 → Skip to procedure before CA007_W3_5 跳至CA007_W3_5之前的程序
2. The name is wrong, and other information is right 名字不对，其他信息都对 → Skip to CA007_W3_4_1 跳至 CA007_W3_4_1
3. The name and other information are wrong 名字和其他信息都不对，上次问错了人

[Preload mothers' names. 加载母亲姓名。]

CA007_W3_4 What's the name of your adopted mother? 您养母的姓名是？

2. Name 姓名
4. Name 姓名
6. Name 姓名
8. Name 姓名
10. Name 姓名
12. Name 姓名
97. Other 其它

PROCEDURE 程序:
If CA007_W3_4 $\neq 97$, skip to procedure before CA007_W3_5.

CA007_W3_4_1 What's the name of your adopted mother? 您的养母的姓名是？ ________

PROCEDURE 程序:
If CA007_W3_0 $= 2$, skip to procedure before CA007_W3_5.

CA007_W3_3 What was your adopted mother's birth year? 您的养母是哪一年出生的？____1850...2014
[IWER:Mark the year using four digits. 访员注意：用 4 位数表示年。]

PROCEDURE 程序:
If CA006_W3_2 $= 1$ and CA007_W3_2 $= 1$, ask CA007_W3_5. 如果 CA006_W3_2 $= 1$ 并且 CA007_W3_2 $= 1$，请询问 CA007_W3_5。

CA007_W3_5 Whether your adopted parents are a couple now? 您的养父和养母目前是否夫妻？

1. Yes 是
2. No 否

PROCEDURE 程序:
If the respondent has alive biological father, ask the following questions from CA009 to CA026_W3. 如果受访者的亲生父亲在世，请询问以下从CA009 到 CA026_W3 的问题。

[Show Card 出示卡片 5]

CA009 What is the highest level of education your biological father has completed? 您亲生父亲的最高学历是？

1. No formal education (illiterate) 未受过教育
2. Did not finish primary school 未读完小学
3. Sishu/home school 私塾毕业
4. Elementary school 小学毕业
5. Middle school 初中毕业
6. High school 高中毕业
7. Vocational school 中专（包括中等师范、职高）毕业

8. Two-/Three-Year College / Associate degree 大专毕业
9. Four-Year College / Bachelor's degree 本科毕业
10. Post-graduate, Master's degree 硕士毕业
11. Post-graduate, Doctoral degree/Ph.D 博士毕业

PROCEDURE 程序:
If CA009_W3 = 1/2/3, then ask the next question. 如果CA009_W3 = 1/2/3，询问下面一个问题。

CA009_W3 Is your biological father literate? 您的亲生父亲是否识字?
1. Yes 是
2. No 否

CA012 Dose your biological father work currently (work includes agricultural work, earning wage work, self-employed activities, and unpaid family business work, et. al.)? 请问您的亲生父亲现在工作吗（工作包括务农、挣工资工作、从事个体、私营活动或不拿工资为家庭经营活动帮工等）?
1. Yes 在工作
2. No 没工作

CA013 How is your biological father health? Very good, good, fair, poor or very poor? 请问您的亲生父亲身体情况怎么样？很好，好，一般，不好还是很不好?
1. Very good 很好
2. Good 好
3. Fair 一般
4. Poor 不好
5. Very poor 很不好

CA014 Which is/was the highest occupation of your biological father? 您的亲生父亲的职位最高的职业是什么?
1. Managers 国家机关、党群组织、企业、事业单位负责人
2. Professionals and technicians 专业技术人员
3. Clerks 办事人员和有关人员
4. Commercial and service workers 商业、服务业人员
5. Agricultural, forestry, husbandry and fishery producers 农、林、牧、渔、水利业生产人员
6. Production and transportation workers 生产、运输设备操作人员及有关人员
7. Can't be specified 不便分类的工作

CA015 What is your biological father average income at present? (not including the money from children, but including other all income like pension and government allowance). 目前您的亲生父亲的收入有多少？(不包括子女给的钱，但包括养老金和政府津贴等所有的收入)
______ yuan/year 元/年 (**CA015_1**) or _____ yuan/month 元/月 (**CA015_2**).

CA016_W3 Where was your biological father born? 您的亲生父亲在哪里出生?
1. The same birthplace as mine 和我的出生地一样
2. My permanent address's village/neighborhood 我的常住地所在社区
3. Another village/neighborhood in my permanent address's county/city/district _____(**CA016_W3_1**) 在我的常住地所在县/市/区的其他村/社区 _____(**CA016_W3_1**)
4. Other 其它:______(**CA016_W3_2**) province_city_county/city/district 省_市_县/市/区, ______(**CA016_W3_3**) township/street 乡/镇/街道, ______(**CA016_W3_4**) village/neighborhood 村/社区
5. Abroad 国外

[Show Card 出示卡片 7]

CA016 Where does your your biological father normally live? 您的亲生父亲一般住在哪里?
1. The same household as mine 与我同住
2. The same or an adjacent dwelling/courtyard with me 与我同一个院子（公寓）或者相邻的院子（公寓）
3. His birthplace 他的出生地
4. Another household in my permanent address's village/neighborhood 我的常住地所在村/社区的其他房子里
5. Another village/neighborhood in my permanent address's county/city/district ______(**CA016_1**) village/neighborhood, how far away ____(**CA016_2**) km 我的常住地所在县/市/区的其他村/社区 ______(**CA016_1**) 村/社区, 离这里有多远: ____(**CA016_2**) 公里
6. Other 其它:______(**CA016_3**) province_city_county/city/district 省_市_县/市/区, ______(**CA016_4**) township/street 乡/镇/街道, ______(**CA016_5**) village/neighborhood 村/社区
7. Abroad 国外

[IWER: county/city includes county-level administrative units/county-level city/district. 访员注意: 县/市包括县级行政单位/县级市/区]

PROCEDURE 程序:
If CA016 = 5/6/7, ask CA017. 如果CA016 = 5/6/7, 询问CA017。

[Show Card 出示卡片 2]

CA017 What kind of location does your biological father live in? 您的亲生父亲现在居住地的类型是?
1. Main city zone 主城区
2. Combination zone between urban and rural areas 城乡结合区
3. The town center 镇中心区
4. ZhenXiang area 镇乡结合区
5. Special area 特殊区域

6. Township central 乡中心区
7. Village 村庄

CA018 Is his hukou in the same place as his current residence? 他的户口是在他现在住的地方吗?

1. Yes 是 → Skip to CA020 跳至 CA020
2. No 否
3. Does not have Hukou 没有户口 → Skip to CA021 跳至 CA021

[CAPI: Preload the R's permanent address.]

CA019 What is the location of your biological father current hukou? 您的亲生父亲目前的户口所在地是?

1. His birthplace 他的出生地
2. My birthplace 我的出生地
3. My permanent address's village/neighborhood 我的常住地所在的村/社区
4. Another village/neighborhood in my permanent address's county/city/district ______(**CA019_1**) village/neighborhood 我的常住地所在县/市/区的其他村/社区 ______ (**CA019_1**) 村/社区
5. Other 其它:______(**CA019_2**) province_city_county/city/district 省 _ 市 _ 县/市/区, ______(**CA019_3**) township/street 乡/镇/街道, ______(**CA019_4**) village/neighborhood 村/社区
6. Abroad 国外

[IWER: county/city includes county-level administrative units/county-level city/district. 访员注意: 县/市包括县级行政单位/县级市/区]

CA020 What is your biological father current hukou status? 您的亲生父亲目前的户口类型是?

1. Agriculture Hukou 农业户口
2. Non-Agriculture Hukou 非农业户口
3. Unified Residency Hukou 统一居民户口
4. Does not have Hukou 没有户口

[F1:"统一居民户口"指的是某些地方实行户口制度改革后，不再区分农业与非农业户口，而是统一为"居民户口"。]

CA021 Does your biological father own a house? 您的亲生父亲有房产吗?

1. Yes 有
2. No 没有 → Skip CA022 跳过 CA022

CA022 Do you know the present value of your biological father house? 您的亲生父亲的房产现在值多少钱? ____ 10000 Yuan 万元

CA023_W3 Is your biological father a party member? 您的亲生父亲是否是共产党员?

1. Yes 是
2. No 否

CA024_W3 Does your biological father belongs to Ethnic Minorities? 您的亲生父亲是否是少数民族?

1. Yes 是
2. No 否

PROCEDURE 程序:
If CA024_W3 = 1, ask CA024_W3_1. 如果CA024_W3 = 1, 请询问 CA024_W3_1。

CA024_W3_1 Which Ethnic Minority does your biological father belong to? 您的亲生父亲是哪一个少数民族?

1. Hui 回族
2. Zhuang 壮族
3. Uighur 维吾尔族
4. Man 满族
5. Yi 彝族
6. Tibetan 藏族
7. Miao 苗族
8. Mongol 蒙古族
9. Dai 傣族
10. Tujia 土家族
11. Lahu 拉祜族
12. Other 其它 ______(**CA024_W3_1_1**)

CA025_W3 Does your biological father have any religious belief? 您的亲生父亲是否有宗教信仰?

1. Yes 是
2. No 否

PROCEDURE 程序:
Ask CA026_W3 if the respondent's biological father is older than 60. 如果受访者的亲生父亲大于 60 岁, 请询问 CA026_W3。

CA026_W3 Can your biological father take care of himself? 您的亲生父亲是否能生活自理?

1. Yes 是
2. No 否

PROCEDURE 程序:
If Family R has alive biological mother/ stepfather/ stepmother/ adopted father/ adopted mother, add one more section with questions identical to those asked about Family R's alive biological father. 如果受访者的亲生母亲/ 继父/ 继母/ 养父/ 养母在世, 请询问与受访者在世亲生父亲相同的问题。

PROCEDURE 程序:

If Family R's spouse has alive biological father/ mother/ stepfather/ stepmother/ adopted father/ adopted mother, add sections with questions identical to those asked the Family R. 如果受访者配偶的亲生父亲/ 母亲/ 继父/ 继母/ 养父/ 养母在世，请询问与受访者相同的问题。

CB CHILDREARING INFORMATION 子女信息

PROCEDURE 程序:

if this is a NEWIW, then ask CB050_W3 and fill in the next table. 如果这是一个基线受访者，则询问 CB050_W3 并且完成下面的表格。

CB050_W3 How many living children do you have? Including biological and step and adapted children. _____ 您有多少个健在的子女？包括亲生子女、继子女和养子女。____

[IWER: 访员注意：姓名和出生年份手动填写；性别为下拉框选项：1. 男 2. 女；子女类型为下拉框选项：我和配偶亲生、我或配偶领养、我自己亲生、我配偶亲生。]

PROCEDURE 程序:

- if this is a REIW, preload all the children's information, if missing, then fill in the blanks; if not missing, please confirm the answers, if the answers not true, then modify them. 加载系统里记录的所有子女相关信息，完成表格的填写，如果为空，需要访员填写，如果可以加载出来，则不需要访员填写，访员需确认答案是否正确，如果不正确，则修改为正确答案。

[访员注意：姓名和出生年份手动填写；性别为下拉框选项：1. 男 2. 女；去世年份和去世原因手动填写；子女类型为下拉框选项：我和配偶亲生、我或配偶领养、我自己亲生、我配偶亲生、不是我的子女。]

[访员注意：因为方言等问题，如果加载的兄弟姐妹姓名汉字不正确，但是属于谐音字的，不用修改姓名。]

[IWER: If the names are not very correctly, then ask if used names; the same sound names or likely sound names are the correct names. 访员注意：如果名字不太一样，则询问是否为曾用名，或者同音字近音字都可算为同一个人的。]

CB051_W3_1 Except recorded children, do you have other living children, including your step children and adapted children? 除了已记录的孩子以外，您还有其他健在的孩子吗？包括继子女和养子女？如果有，有几个？ ____0...25 个

[IWER: 子女类型为下拉框选项：我和配偶亲生、我或配偶领养、我自己亲生、我配偶亲生；姓名为手动填入；出生年份为手动填入。]

PROCEDURE 程序:

- repeat questions for each living child. 请针对每个健在的孩子询问。

PROCEDURE 程序:

- Preload the information of children, if there is none, then ask; otherwise, skip. 加载关于子女的数据，如果数据为空，则询问该问题；如果数据不为空，则跳过。

ChildName Child Name is ____ ? 子女姓名是 ____ ?
ChildBYear (ChildName)'s birth year is____ ?1850...2015【子女姓名】的出生年份是 ____ ?
ChildGender (ChildName)'s gender is ?【子女姓名】的性别是什么?

1. Male 男性
2. Female 女性

ChildSta What is (ChildName) doing now ?【子女姓名】现在在干什么?

1. studying in the school 上学
2. working 工作
3. studying while working 边工边读
4. neither studying nor working 既不读书也不工作

ChildYN Is (ChildName)'s record correct ?【子女姓名】的记录正确吗?

1. Yes 正确
2. No 错误

PROCEDURE 程序:
- repeat questions for each passed away. 请针对每个去世的孩子询问。

CB052_W3_Death When did (ChildName) pass away?The cause is____?
【子女姓名】是 ____(**CB052_W3_Death_1**) 年去世的。
去世原因是? ____(**CB052_W3_Death_2**)

CB052_W3 What is the highest level of education [child's name] have completed? (not including adult education) 【子女姓名】的最高学历是?(不包括成人教育)

1. No formal education (illiterate) 未受过正规教育
2. Did not finish primary school 未读完小学
3. Sishu/home school 私塾
4. Elementary school 小学毕业
5. Middle school 初中毕业
6. High school 高中毕业
7. Vocational school 中专(包括中等师范、职高)毕业
8. Two-/Three-Year College/Associate degree 大专毕业
9. Four-Year College/Bachelor's degree 本科毕业
10. Master's degree 硕士毕业
11. Doctoral degree/Ph.D. 博士毕业

PROCEDURE 程序:
If CB052_W3 = 1/2/3, ask the next question. 如果CB052_W3 = 1/2/3, 询问下面一个问题。

CB052_W3_1 Is [child's name] literate? 【子女姓名】是否识字?

1. Yes 是
2. No 否

PROCEDURE 程序:
- Preload the R's permanent address 加载受访者的常住地址

CB053 Where does this [child's name] normally live now? 目前,【孩子姓名】在哪里常住?
1. This household, and economicly dependent. 这个家里且经济上不独立
2. This household, but economicly independent. 这个家里,但是经济上独立
3. The same or adjacent dwelling/courtyard with you 与您同一个院子(公寓)或者相邻的院子(公寓)
4. Another household in your permanent address's village/neighborhood 您的常住地所在村/社区的其他房子里
5. Another village/neighborhood in your permanent address's county/city/district; distance from here: ____km 您的常住地所在县/市/区的其他村/社区 ______ 村/社区 (**CB053_1**), 离这里有多远: ____(**CB053_2**) 公里
6. Other 其它:province_city_county/city/district 省 _ 市 _ 县/市/区, ______(**CB053_3**) 乡/镇/街道 ______(**CB053_4**) village/neighborhood 村/社区
7. Abroad 国外

PROCEDURE 程序:
If CB053 = 5/6/7, ask CB054; otherwise, go to CB055. 如果CB053 = 5/6/7, 询问CB054; 否则询问CB055

CB054 In what type of location does [child's name] live? 【孩子姓名】现在居住地的类型是?
1. Main city zone 主城区
2. Combination zone between urban and rural areas 城乡结合区
3. The town center 镇中心区
4. ZhenXiang area 镇乡结合区
5. Special area 特殊区域
6. Township central 乡中心区
7. Village 村庄

CB055 What is the current hukou status of [child's name]? 【孩子姓名】目前的户口状态是什么?
1. Agriculture Hukou 农业户口
2. Non-Agriculture Hukou 非农业户口
3. Unified Residency Hukou 统一居民户口
4. Do not have Hukou 没有户口

[F1:"统一居民户口"指的是某些地方实行户口制度改革后,不再区分农业与非农业户口,而是统一为"居民户口"。]

PROCEDURE 程序:
If CB055= 3, ask CB055_W2_1 and CB055_W2_2; otherwise, skip to CB056_W3 如果CB055= 3, 则询问CB055_W2_1 和CB055_W2_2, 否则跳至CB056_W3

CB055_W2_1 What is [child's name]'s Hukou status before he/she has the unified residency hukou?? 【孩子姓名】在获得统一居民户之前是什么户口？

1. Agriculture Hukou 农业户口
2. Non-Agriculture Hukou 非农业户口
3. Do not have Hukou 没有户口

CB055_W2_2 When did [child's name] have the unified residence Hukou? ____ 2000...2015 【孩子姓名】什么时候得到的统一居民户口？ ____ 2000...2015

CB081 Where was [child's name] born? 【孩子姓名】在哪里出生？

1. The same birthplace as me 和我的出生地一样
2. My permanent address's village/neighborhood 我的常住地所在社区
3. Another village/neighborhood_____ in my permanent address's county/city/district 在我的常住地所在县/市/区的其他村/社区 _____**(CB081_1**)
4. Other 其它:province_city_county/city/district 省 _ 市 _ 县/市/区,______(**CB081_2**) 乡/镇/街道 ______**(CB081_3)** 村/社区
5. Abroad 国外

[Preload the R's permanent address. 加载受访者的常住地址]

CB057 What is the current hukou location of [child's name]? 【孩子姓名】目前的户口所在地是？

1. This household 这个家里
2. His/her birthplace 他/她的出生地
3. Your permanent address' s village/neighborhood 您的常住地所在的村/社区
4. Another village/neighborhood ______in your permanent address's county/city/district 您的常住地所在县/市/区的其他村/社区 ______**(CB057_1)**
5. Other 其它:province_city_county/city/district 省 _ 市 _ 县/市/区, ______**(CB057_2)** 乡/镇/街道; ______**(CB057_3)** 村/社区
6. Other(specify) 其他（请注明） ______**(CB057_4)**

[IWER: county/city includes county-level administrative units/county-level city/district. 访员注意: 县/市包括县级行政单位/县级市/区]

[CB063 BRANCHPOINT]

- If [child's name] is less than 16, go to CB063_W3_1 如果孩子不满 16 岁，跳至 CB063_W3_1

CB063 What is [child's name] status? 【孩子姓名】的婚姻状况是？

1. Married with spouse present 已婚并与配偶一同居住
2. Married but not living with spouse temporarily for reasons such as work 已婚，但因为工作等原因暂时没有跟配偶在一起居住
3. Separated 分居（不再作为配偶共同生活）
4. Divorced 离异

5. Widowed 丧偶
6. Never married 从未结婚
7. Cohabitated 同居

CB063_W3_1 How is [child's name] health? Very good, good, fair, poor or very poor? 请问【孩子姓名】的身体情况怎么样？很好，好，一般，不好还是很不好？

1. Very good 很好
2. Good 好
3. Fair 一般
4. Poor 不好
5. Very poor 很不好

CB063_W3_2 Is [child's name] the party member? 【孩子姓名】是不是党员？

1. Yes 是
2. No 否

CB063_W3_3 Is [child's name] the Ethnic Minorities? 【孩子姓名】是不是少数民族？

1. Yes 是
2. No 否 → Skip to CB063_W3_5 跳至 CB063_W3_5

CB063_W3_4 Which Ethnic Minorities of [name of child]? 【孩子姓名】是哪个少数民族？[Procedure: ask each child who was chose from CB063_W3_3. 对于每个在 CB063_W3_3 中选中的孩子依次询问。]

1. Hui 回族
2. Zhuang 壮族
3. Weiwuer 维吾尔族
4. Man 满族
5. Yi 彝族
6. Zang 藏族
7. Miao 苗族
8. Mongol 蒙古族
9. Dai 傣族
10. Tujia 土家族
11. Lahu 拉祜族
12. other 其它：______ （**CB063_W3_4_1**）

CB063_W3_5 Does [child's name] have any religious belief? 【孩子姓名】是否有宗教信仰？

1. Yes 是
2. No 否

PROCEDURE 程序:

- If [child's name] is older than 60, ask CB063_W3_6 如果孩子超过 60 岁，询问 CB063_W3_6

CB063_W3_6 Can [child's name] take care of him/herself? 【孩子姓名】是否能生活自理?

1. Yes 是
2. No 否

PROCEDURE 程序:

- If the children has never married (CB063= 6), please skip to CB064_W3_2
  如果这个孩子从未结婚 (CB063= 6), 跳至CB064_W3_2

CB065 How many children does [child's name] have? 【孩子姓名】有多少个子女? _____ 0...25 个

PROCEDURE 程序:

- If [child's name] does not have children (CB065 = 0) , please skip to CB064_W3_2
  如果回答 "0" 个, 跳至CB064_W3_2

CB066 How many children under 16 does [child's name] have? 【孩子姓名】有多少个 16 岁以下的子女? ____ 0...25 个

CB067 How many grandchildren does [child's name] have? [孩子姓名] 有多少个孙子女? __ 0...25 个

PROCEDURE 程序:

- If [child's name] havs grandchildren (CB065 > 0), please ask CB068
  如果回答有孙子女, 回答CB068

CB068 How many grandchildren under age 16 does [child's name] have? [孩子姓名] 有多少个 16 岁以下的孙子女? __ 0...25 个
[Softcheck: if the number of grandchildren under age 16 daughters is smaller than the number of grandchildrenadult daughters. 如果CB067 <CB068, 则进行检查]

PROCEDURE 程序:

- 如果【孩子姓名】是 65 岁以下非农业户口, 则询问CB064_W3_2-CB064_W3_4, 否则跳至CB069

CB064_W3_2 Does [child's name] participate in the New Rural Social Pension Insurance? 【孩子姓名】是否参加了新农保?

1. Yes 是
2. No 否 → Skip to CB069 跳至CB069

CB064_W3_3 In what month and year was [child's name] first cover by New Rural Social Pension Insurance?
【孩子姓名】是从什么时候加入新型农村社会养老保险(新农保)的? ____ 2008...2013 (**CB063_W3_3_1**) year 年 ____ 0...12 (**CB063_W3_3_2**) month 月

CB064_W3_4 Does [child's name]'s participatation related to parents?【孩子姓名】当年参加新农保与您和配偶领取新农保是否有关联?(多选题)

1. Yes 是的，孩子参加新农保的时候，我和配偶到了领取新农保的年龄，当年村里要求子女参加了新农保父母才能有资格领取
2. No 不是，孩子自己认为新农保很划算，自愿参加，与父母领取没有关系
3. Other 因为其它原因 ________(CB064_W3_4_1)，村里要求孩子强制参加，与我和配偶领取没有关系（请详细说明其它原因）

CB069 How much did the total income of [child's name] (and his/her spouse) in the past year? 【孩子姓名】(和他/她的配偶）去年的总收入属于下面哪一类?

1. 0 没有收入
2. less than 2000 yuan 少于 2 千元
3. 2000-5000 yuan 2 千与 5 千之间
4. 5000-10000 yuan 5 千与 1 万之间
5. 10000-20000 yuan 1 万与 2 万之间
6. 20000-30000 yuan 2 万与 3 万之间
7. 30000-50000 yuan 3 万与 5 万之间
8. 50000-100000 yuan 5 万与 10 万之间
9. 100000-150000 yuan 10 万与 15 万之间
10. 150000-200000 yuan 15 万与 20 万之间
11. 200000-300000 yuan 20 万与 30 万之间
12. more than 300000 yuan 多于 30 万

CB071_W3 Does [child's name] own a house? 【孩子姓名】有房产吗?

1. Yes 有
2. No 没有→ Skip to next child 询问下一个孩子

CB072_W3 Do you know the present value of your child's house? 您孩子的房产现在值多少钱? _____ 10000 Yuan 万元

PROCEDURE 程序:
- repeat questions for additional children. 返回询问另一个子女。

PROCEDURE 程序:
- go/proceed to the next person. 请询问下一个人。

CC SIBLINGS 兄弟姐妹

[INTRO: Next I have some questions about your brothers and sisters. 接下来我们会问一些有关您兄弟姐妹的问题。]

PROCEDURE 程序:

If this is a REIW and XSiblingNum > 0, ask the following questions displayed as a table. 如果这是一个回访受访者并且 XSiblingNum > 0, 回答以下以表格形式显示的问题。

CC000_W3 Is the following information of your living siblings in 2014 correct? If not, modify them in the blanks. 下面是我们记录的您的 2014 年健在的兄弟姐妹，请问这些信息正确吗？如果不对，请直接在表格中修改。
[访员注意：姓名、出生年份为手动填写；性别为下拉框选项：1. 男 2. 女；与自己的关系为下拉框选项：1. 同父同母 2. 同父异母 3. 异父同母 4. 异父异母；正误类型：1. 正确 2. 错误。]
[IWER: If the names are not very correctly, then ask if used names; the same sound names or likely sound names are the correct names. 访员注意：如果名字不太一样，则询问是否为曾用名，或者同音字近半字都可算为同一个人的名字。]
[访员注意：如果部分信息不对，请修改相应信息，并选择正确；如果信息完全不对，请选择错误]

CC001_W3 Is there any of your siblings died since 2014? Choose all those apply. 您的下列兄弟姐妹有去世的吗？可多选。

1. Sibling's name 兄弟姐妹姓名
2. Sibling's name 兄弟姐妹姓名

⋮

15. Sibling's name 兄弟姐妹姓名
97. None 没有去世的 → Skip CC002_W3 跳过CC002_W3

CC002_W3 What's the cause of [sibling's name]'s death? 【兄弟姐妹姓名】去世的原因是？ ________

CC000_W3_1 Except you, how many living siblings do you have by 2014, including your step siblings and adopted siblings? 除您以外，您总共有几个活到 2014 年的兄弟姐妹，包括继兄弟姐妹和养兄弟姐妹？ ____0...15 person 个

PROCEDURE 程序:

If CC000_W3_1 > 0, ask CC003_W3_2. 如果 CC000_W3_1 > 0, 请询问 CC003_W3_2。

CC003_W3_2 Ask the information of living siblings from oldest to youngest (except recorded siblings). 按照年龄从大到小的顺序填写您的的兄弟姐妹相关信息（除了已经记录的以外）。
[访员注意：姓名、出生年份为手动填写；性别为下拉框选项：1. 男 2. 女；与自己的关系为下拉框选项：1. 同父同母 2. 同父异母 3. 异父同母 4. 异父异母。]

PROCEDURE 程序:

If XSiblingName is not missing, ask each one the next questions from CC003_W3 to CC019_W3. 如果兄弟姐妹的姓名没有缺失，循环询问下列从CC003_W3 到 CC019_W3 的问题。

CC003_W3 What's the highest level of education [sibling's name] got? (not including adult education)【兄弟姐妹姓名】获得的最高学历是什么？（不包括成人教育）

1. No formal education (illiterate) 未受过正规教育
2. Did not finish primary school 未读完小学
3. Sishu/home school 私塾
4. Elementary school 小学毕业
5. Middle school 初中毕业
6. High school 高中毕业
7. Vocational school 中专（包括中等师范、职高）毕业
8. Two-/Three-Year College/Associate degree 大专毕业
9. Four-Year College/Bachelor's degree 本科毕业
10. Master's degree 硕士毕业
11. Doctoral degree/Ph.D. 博士毕业

PROCEDURE 程序:
If CC003_W3 = 1/2/3, ask the next question. 如果CC003_W3 = 1/2/3, 询问下面一个问题。

CC003_W3_0 Is [sibling's name] literate? 【兄弟姐妹姓名】是否识字？
1. Yes 是
2. No 否

PROCEDURE 程序:
If CC000_W3_1 > 0, ask the next questions from CC004_W3 to CC018_W3 for each of the newly added siblings. 如果CC000_W3_1 > 0, 依次对于新添加的兄弟姐妹询问下列从CC004_W3 到 CC018_W3 的问题。

CC004_W3 Is [sibling's name] the party member? 【兄弟姐妹姓名】是不是共产党员？
1. Yes 是
2. No 否

CC005_W3 Does [sibling's name] belong to the Ethnic Minorities? 【兄弟姐妹姓名】是不是少数民族？
1. Yes 是
2. No 否 → Skip to CC016_W3 跳至 CC016_W3

[Show Card 出示卡片 8]

CC006_W3 Which Ethnic Minority does [sibling's name] belong to? 【兄弟姐妹姓名】是哪个少数民族？
1. Hui 回族
2. Zhuang 壮族
3. Uighur 维吾尔族
4. Man 满族
5. Yi 彝族

6. Tibetan 藏族
7. Miao 苗族
8. Mongol 蒙古族
9. Dai 傣族
10. Tujia 土家族
11. Lahu 拉祜族
12. other 其它: ______(**CC006_W3_1**)

CC016_W3 Did [sibling's name] have the experience of Down to the Countryside Movement? 【兄弟姐妹姓名】是否有上山下乡的经历?

1. Yes 是
2. No 否

CC017_W3 Did [sibling's name] have the experience of army? 【兄弟姐妹姓名】是否有参军的经历?

1. Yes 是
2. No 否

CC018_W3 Does [sibling's name] have any religious belief? 【兄弟姐妹姓名】是否有宗教信仰?

1. Yes 是
2. No 否

PROCEDURE 程序:
Ask CC006_W3_2 for those siblings who are older than 60. 对于 60 岁以上的兄弟姐妹询问 CC006_W3_2。

CC006_W3_2 Can [sibling's name] take care of him/herself? 【兄弟姐妹姓名】是否能生活自理?

1. Yes 是
2. No 否

CC007_W3 How many children does [sibling's name] have? 【兄弟姐妹姓名】有多少个子女? ___ 0...25 person 个

PROCEDURE 程序:
If CC007_W3 = 0, skip to CC011_W3. 如果CC007_W3 = 0, 跳至CC011_W3。

CC008_W3 How many children under age 16 does [sibling's name] have? 【兄弟姐妹姓名】有多少个 16 岁以下的子女? ___ 0...25 person 个

CC009_W3 How many grandchildren does [sibling's name] have? 【兄弟姐妹姓名】有多少个孙子女? ___ 0...25 person 个

PROCEDURE 程序:
If CC009_W3 = 0, skip to CC011_W3. 如果CC009_W3 = 0, 跳至CC011_W3。

CC010_W3 How many grandchildren under age 16 does [sibling's name] have? 【兄弟姐妹姓名】有多少个 16 岁以下的孙子女？___ 0...25 person 个

CC011_W3 What is [sibling's name] marital status? 【兄弟姐妹姓名】的婚姻状况是？
1. Married with spouse at present 已婚并与配偶一同居住
2. Married but not living with spouse temporarily for reasons such as work 已婚，但因为工作等原因暂时没有跟配偶在一起居住
3. Separated 分居（不再作为配偶共同生活）
4. Divorced 离异
5. Widowed 丧偶
6. Never married 从未结婚
7. Cohabitated 同居

CC012_W3 How is [sibling's name] health? Very good, good, fair, poor or very poor? 请问【兄弟姐妹姓名】的身体情况怎么样？很好，好，一般，不好还是很不好？
1. Very good 很好
2. Good 好
3. Fair 一般
4. Poor 不好
5. Very poor 很不好

CC013_W3 Does [sibling's name] work now? 【兄弟姐妹姓名】现在工作吗？
1. Yes 是
2. No 否

[Show Card 出示卡片 9]

CC015_W3 Which was the highest occupation of [sibling's name]? 您【兄弟姐妹姓名】做到的职位最高的职业是什么？
1. Managers 国家机关、党群组织、企业、事业单位负责人
2. Professionals and technicians 专业技术人员
3. Clerks 办事人员和有关人员
4. Commercial and service workers 商业、服务业人员
5. Agricultural, forestry, husbandry and fishery producers 农、林、牧、渔、水利业生产人员
6. Production and transportation workers 生产、运输设备操作人员及有关人员
7. Can't be specified 无法分类的工作

CC019_W3 How about the life of [sibling's name] compared with yours? 【兄弟姐妹姓名】和你相比过得怎么样？
1. Much better than mine 比我好很多
2. A little better than mine 比我好一点
3. Same with mine 比我差不多

4. A little worse than mine 比我差一点
5. Much worse than mine 比我差很多

PROCEDURE 程序:
If proxy = 0, answer CG003_W2. 如果 proxy = 0，回答 CG003_W2。

CG003_W2 How often did the respondent receive assistance in answering section CA/CC? 受访者填写 CA/CC 部分问卷时是否求助？
1. Never 从未
2. A few times 偶尔几次
3. Most or all of the time 大多

PROCEDURE 程序:
If Spouse's name (XSName) is not missing, skip CA024. 如果配偶姓名没有缺失，跳过CA024。

CA024 Do you keep in contact with your parents-in-law? 您和岳父母/公公婆婆保持联系吗？
1. Yes, keep in contact 是，保持联系
2. No, have no contact or no parents-in-law 不，没有联系或没有岳父母/公公婆婆

PROCEDURE 程序:
If Spouse's name (XSName) is not missing and Spouse is not dead (XSDied!=1), or CA024 = 1, plus proxy = 0 and XSDied! = 1, ask CC005_W2_1. 如果配偶姓名没有缺失并且配偶没有过世，或者CA024 = 1，并且 proxy = 0 和 XSDied! = 1，询问CC005_W2_1。

CC005_W2_1 If the Family R's Spouse is in, please ask the Spouse about Spouse's parents and siblings questions; if the Spouse is not in, please ask Family R for proxy. For the following questions about Spouse's parents and siblings (Section CA/ Section CC), who answered the questions? Please record. 如果受访者配偶在的话，就请配偶回答他/她自己的父母/兄弟姐妹的问题；如果配偶不在，则由受访者回答。接下来的配偶的父母/兄弟姐妹部分的问题是由谁来回答的？请记录。
1. Family R 家庭受访者自己回答
2. Spouse 配偶回答

PROCEDURE 程序:
If Family R's Spouse has sibling(s), add one more section with questions identical to those asked about Family R's siblings (CC000_W3_S to CC019_W3_S). And if CC005_W2_1 = 2 and proxy = 0, answer CG003_W2. 如果受访者的配偶有兄弟姐妹，请询问与受访者的兄弟姐妹相同的问题 (CC000_W3_S 至 CC019_W3_S)。如果 CC005_W2_1 = 2 and proxy = 0，回答 CG003_W2。

PROCEDURE 程序:
Variables standing for the information of spouse's sibling end up with "_S". 反映配偶兄弟姐妹信息的变量以 "_S" 结尾。

C2 TIME TRANSFER AND TRANSFERS 家庭交往与经济帮助

[Introduction: In the following three parts: CD time transfer, CE transfer and CF time spent providing care. we will ask you how you contact with parents and children, and economic trancfers. 接下来的三部分，我们将询问您与父母、子女间的交往和家庭得到及提供的经济帮助。]

CD TIME TRANSFER 与父母、子女间的交往

CONTACT WITH PARENTS 与父母的交往

PROCEDURE 程序:

Skip to CD003 if father/mother/father-in-law/mother-in-law is not alive OR father/mother/father-in-law/mother-in-law is household member. 如果父亲/母亲/岳父(公公)/岳母 (婆婆) 去世了，或者父亲/母亲/岳父 (公公)/岳母 (婆婆) 是家户成员，跳至CD003.

LOOP:

Repeat each living and non-resident parents couple about biological parents/step parents/adapted parents/parents-in-law, if each couple both have father and mother, then ask CD001

if the couple just have one person, then ask CD001_W3_1/CD001_W3_2

循环提问健在的没住在一起的父母夫妻，包括亲生父母/养父母/岳父母（公公婆婆），如果每对父母夫妻都是完整的两人，则询问CD001；如果父母夫妻只有一个人，则询问CD001_W3_1/CD001_W3_2

CD001 [If the father/mother/father-in-law/mother-in-law is alive] Whom does your father/mother/father-in-law/mother-in-law live with (choose all that apply)? 【如果父母亲/岳父母（公公婆婆）/继父母/养父母健在】您父母亲/岳父母 (公公婆婆)/继父母/养父母和谁住在一起 (可多选)?

1. Live alone with him/herself 自己单独住
2. his/her spouse 他/她的配偶
3. my spouse and I 我和我配偶
4. my siblings 我的兄弟姐妹
5. siblings of my spouse 我配偶的兄弟姐妹
6. my children 我的子女
7. my siblings' children 我兄弟姐妹的子女
8. my spouse's siblings' children 我配偶的兄弟姐妹的子女
9. other relatives 其它亲戚
10. Take turns in children's homes 在子女家轮流住
11. Nursing home 养老院
12. Other 其他

PROCEDURE 程序:

If CD001 $\neq 2$, then ask CD002. 如果CD001 $\neq 2$, 询问 CD002.

CD002 How often do you/your spouse see your father/mother/ father-in-law/mother-in-law? 您或您的配偶多长时间去看望一次您的父亲/母亲/岳父 (公公)/岳母 (婆婆)?

1. Almost every day 差不多每天
2. 2-3 times a week 每周 2-3 次
3. Once a week 每周一次
4. Every two weeks 每半个月一次
5. Once a month 每月一次
6. Once every three months 每三个月一次
7. Once every six months 半年一次
8. Once a year 每年一次
9. Almost never 几乎从来没有
10. Other 其他

PROCEDURE 程序:
- If answer to (**CD001**) is "my siblings", please ask CD001_W3_1
  如果回答"我的兄弟姐妹",询问CD001_W3_1

CD001_W3_1 Which siblings? 我的哪些兄弟姐妹?(加载所有的我的兄弟姐妹的信息)

PROCEDURE 程序:
- If answer to (**CD001**) is "siblings of my spouse", please ask CD001_W3_2
  如果回答"我配偶的兄弟姐妹",询问CD001_W3_2

CD001_W3_2 Which siblings of my spouse? 我配偶的哪些兄弟姐妹?(加载所有配偶的兄弟姐妹信息)

PROCEDURE 程序:
- If answer to (**CD001**) is "my children", please ask CD001_W3_3
  如果回答"我的子女",询问CD001_W3_3

CD001_W3_3 Which children? 哪些子女?(加载所有我的子女的信息)

PROCEDURE 程序:
- If answer to (**CD001**) is "my siblings' children", please ask CD001_W3_4
  如果回答"我兄弟姐妹的子女",询问CD001_W3_4

CD001_W3_4 Which siblings' children? 哪些兄弟姐妹的子女?(加载所有我的兄弟姐妹的子女的信息)

PROCEDURE 程序:
- If answer to (**CD001**) is "my spouse's siblings' children", please ask CD001_W3_5
  如果回答"我配偶的兄弟姐妹的子女",询问CD001_W3_5

CD001_W3_5 Which spouse's siblings' children? 哪些配偶的兄弟姐妹的子女?(加载所有我的配偶的兄弟姐妹的子女的信息)

[IWER: If the father and mother or father-in-law and mother-in-law live together, do not repeat the other one. 访员注意：如果父母亲或者岳父母/公婆两人住在一起，那访问完其中一个人之后不再循环问另一个人。]

CONTACT WITH CHILDREN 与子女的交往

PROCEDURE 程序:
If respondent has no non-cohabiting children, skip to CF001. 如果受访者没有不在一起生活的子女，跳至CF001

PROCEDURE 程序:
For each of the non-coresident child, ask the following two questions CD003-CD004. 对每一个不住在一起的子女，询问以下两个问题CD003-CD004.

CD003 How often do you see (child's name)? 您多长时间见到 [孩子姓名]?

1. Almost every day 差不多每天
2. 2-3 times a week 每周 2-3 次
3. Once a week 每周一次
4. Every two weeks 每半个月一次
5. Once a month 每月一次
6. Once every three months 每三个月一次
7. Once every six months 半年一次
8. Once a year 每年一次
9. Almost never 几乎从来没有
10. Other 其他

PROCEDURE 程序:
IF (CD003= 1 – 3), skip (CD004). 如果 (CD003= 1 – 3), 跳过 (CD004).

CD004 How often do you have contact with (child's name) either by phone, text message, mail, or email, when you didn't live with (child's name)? 您和 [孩子姓名] 不在一起住的时候，您多长时间跟 [孩子姓名] 通过电话、短信、信件或者电子邮件联系?

1. Almost every day 差不多每天
2. 2-3 times a week 每周 2-3 次
3. Once a week 每周一次
4. Every two weeks 每半个月一次
5. Once a month 每月一次
6. Once every three months 每三个月一次
7. Once every six months 半年一次
8. Once a year 每年一次
9. Almost never 几乎从来没有
10. Other 其他

CE TRANSFERS 家庭得到及提供的经济帮助

[Introduction: Families sometimes help one another in a variety of ways, and each type of help can be important. The next questions are about help you (and your spouse) have given to or received from your non-coresident family members in the past year. 有时候，家庭之间会有多种形式的互相帮助，而每一种形式的帮助都很重要。所以，接下来我们想了解一下，您和您配偶有没有从其他人那里得到或者给其他人什么经济帮助。]

Receipt of Economic Assistance including Cash and In-kind Transfers 得到经济帮助（包括现金和实物等）

[CAPI: Preload parents' names.

1. [Biological father's name] [Biological mother's name] if both biological parents are living and not divorced; [Biological father's name] [Stepmother's name] if biological parents are divorced and names of biological father and stepmother are nonmissing; [Biological father's name] if biological parents are divorced, biological father's name is nonmissing and stepmother's name is missing; [Stepmother's name] if biological parents are divorced, biological father's name is missing and stepmother's name is nonmissing; [] if biological parents are divorced and names of biological father and stepmother are missing
2. [Biological mother's name] [Stepfather's name] if biological parents are divorced and names of biological mother and stepfather are nonmissing; [Biological mother's name] if biological parents are divorced, biological mother's name is nonmissing and stepfather's name is missing; [Stepfather's name] if biological parents are divorced, biological mother's name is missing and stepfather's name is nonmissing; [] if biological parents are divorced and names of biological mother and stepfather are missing
3. [Adopted father's name] [Adopted mother's name] if adopted parents are the spouse; [Adopted father's name] if adopted parents are not the spouse and adopted father's name is nonmissing
4. [Adopted mother's name] if adopted parents are not the spouse and adopted mother's name is nonmissing

5, 6, 7 and 8 display the name(s) of the spouse's biological father / biological mother / stepfather / stepmother / adopted father / adopted mother in the similar manner.]

PROCEDURE 程序:

Repeat questions from CE002 to CE023 according to the list of the nonmissing parents' names. 根据列出的未缺失的父母亲姓名，重复询问 CE002 到 CE023。

[IWER: money/in-kind support means support living expenses / foodstuff / vegetables / clothes / water and electricity / telephone rate and other daily consumption; Marriage and funeral / move to new house / in hospital/ go to university / new born and other economic transfer. 访员注意：给钱/东西是指提供生活费、粮食、买菜、衣服、水电费及电话费等日常消费支出，以及过年过节、婚丧嫁娶、搬迁新房、生病、升学、有新生儿及其他情况下的经济往来。]

CE002 In the past year, how much economic supports did you or your spouse receive from

your [father's name][mother's name]? 过去一年，您或您的配偶从您的【父亲姓名】【母亲姓名】那里共收到过多少经济支持？

1. Total money support ____(**CE002_1**) yuan, among which how much is regular ____(**CE002_2**) yuan (for example, support living expenses/water and electricity/telephone rate/return loan or other cost in regular). 总共给钱 ____(**CE002_1**) 元，其中，定期给 ____(**CE002_2**) 元（如定期提供生活费、支付每月水电费及电话费、支付房贷/房租费用或其他定期的费用）。
2. Total in-kind support ____(**CE002_3**) yuan, among which how much is regular ____(**CE002_4**) yuan (for example, support food/vegetables/clothes or other in-kind support in regular). 总共给物 ____(**CE002_3**) 元，其中，定期给物 ____**CE002_4**) 元（如定期提供粮食、买菜、买衣服或其他物品等）。

[IWER: Regular means supporting at fixed time such as per month/quarter of a year/half of a year/year, etc. 访员注意：定期是指按月、按季度、按半年、按年给钱或东西，时间上大致固定。]
[IWER: If give no money or in-kind support, please fill in "0"; if respondent answer RF or DK, then ask CE003, or skip to CE022. 访员注意：如果没有给钱或者没有给物，请填入"0"；拒绝回答或不知道的询问CE003，否则跳至CE022.]

CE003 Add unfolding brackets (100/200/400/800/1600 yuan) for CE002_1, CE002_2, CE002_3 or CE002_4 answered RF or DK. 请在此处对 CE002_1, CE002_2, CE002_3 或者 CE002_4 中回答不知道或拒绝回答的添加分级展开问题（100/200/400/800/1600 元）。

CE022 In the past year, how much economic supports did you or your spouse provide to your [father's name][mother's name]? 过去一年，您或您的配偶给您的【父亲姓名】【母亲姓名】多少经济支持？

1. Total money support ____(**CE022_1**) yuan, among which how much is regular ____(**CE022_2**) yuan (for example, support living expenses/water and electricity/telephone rate/return loan or other cost in regular) . 总共给钱 ____(**CE022_1**) 元，其中，定期给 ____(**CE022_2**) 元（如定期提供生活费、支付每月水电费及电话费、支付房贷/房租费用或其他定期的费用）。
2. Total in-kind support ____(**CE022_3**) yuan, among which how much is regular ____(**CE022_4**) yuan (for example, support food/vegetables/clothes or other in-kind support in regular). 总共给物 ____(**CE022_3**) 元，其中，定期给物 ____(**CE022_4**) 元（如定期提供粮食、买菜、买衣服或其他物品等）。

[IWER: Regular means supporting at fixed time such as per month/quarter of a year/half of a year/year, etc. 访员注意：定期是指按月、按季度、按半年、按年给钱或东西，时间上大致固定。]
[IWER: If give no money or in-kind support, please fill in "0"; if respondent answer RF or DK, then ask CE023, otherwise skip to CE009. 访员注意：如果没有给钱或者没有给物，请填入"0"；拒绝回答或不知道的询问CE023，否则跳至CE009.]

CE023 Add unfolding brackets (100/200/400/800/1600 yuan) for CE022_1, CE022_2, CE022_3 or CE022_4 answered RF or DK. 请在此处对 CE022_1, CE022_2, CE022_3 或者 CE022_4 中回答不知道或拒绝回答的添加分级展开问题（100/200/400/800/1600 元）。

[CAPI: Preload all living children's names. 加载所有活着的子女姓名。]

CE009 In the past year, how much economic supports did you or your spouse receive from your [child's name]? 过去一年，您或您的配偶从您的【孩子姓名】那里收到过多少经济支持？访员注意：包括从【孩子姓名】的孩子那里收到的经济支持。

1. Total money support _____(**CE009_1**) yuan, among which how much is regular _____(**CE009_2**) yuan (for example, support living expenses/water and electricity/telephone rate/return loan or other cost in regular). 总共给钱 _____(**CE009_1**) 元，其中，定期给 _____(**CE009_2**) 元（如定期提供生活费、支付每月水电费及电话费、支付房贷/房租费用或其他定期的费用）。
2. Total in-kind support _____(**CE009_3**) yuan, among which how much is regular _____(**CE009_4**) yuan (for example, support food/vegetables/clothes or other in-kind support in regular). 总共给物 _____(**CE009_3**) 元，其中，定期给物 _____(**CE009_4**) 元（如定期提供粮食、买菜、买衣服或其他物品等）。

[IWER: Regular means supporting at fixed time such as per month/quarter of a year/half of a year/year, etc. 访员注意：定期是指按月、按季度、按半年、按年给钱或东西，时间上大致固定。]
[IWER: If give no money or in-kind support, please fill in "0"; if respondent answer RF or DK, then ask CE010, or skip to CE029. 访员注意：如果没有给钱或者没有给物，请填入"0"；拒绝回答或不知道的询问CE010，否则跳至CE029.]

CE010 Add unfolding brackets (100/200/400/800/1600 yuan) for CE009_1, CE009_2, CE009_3 or CE009_4 answered RF or DK. 请在此处对 CE009_1, CE009_2, CE009_3 或者 CE009_4 中回答不知道或拒绝回答的添加分级展开问题（100/200/400/800/1600 元）。

CE029 In the past year, how much economic supports did you or your spouse provide to your [child name]? 过去一年，您或您的配偶给您的 [孩子姓名] 多少经济支持？

1. Total money support _____(**CE029_1**) yuan, among which how much is regular _____(**CE029_2**) yuan (for example, support living expenses/water and electricity/telephone rate/return loan or other cost in regular). 总共给钱 _____(**CE029_1**) 元，其中，定期给 _____(**CE029_2**) 元（如定期提供生活费、支付每月水电费及电话费、支付房贷/房租费用或其他定期的费用）。
2. Total in-kind support _____(**CE029_3**) yuan, among which how much is regular _____(**CE029_4**) yuan (for example, support food/vegetables/clothes or other in-kind support in regular). 总共给物 _____(**CE029_3**) 元，其中，定期给物 _____(**CE029_4**) 元（如定期提供粮食、买菜、买衣服或其他物品等）。

[IWER: Regular means supporting at fixed time such as per month/quarter of a year/half of a year/year, etc. 访员注意：定期是指按月、按季度、按半年、按年给钱或东西，时间上大致固定。]
[IWER: If give no money or in-kind support, please fill in "0"; if respondent answer RF or DK, then ask CE030, or skip to CE072. 访员注意：如果没有给钱或者没有给物，请填入"0"；拒绝回答或不知道的询问 CE030_1，否则跳至CE072_W3之前的跳转点.]

CE030 Add unfolding brackets (100/200/400/800/1600 yuan) for CE029_1, CE029_2, CE029_3 or CE029_4 answered RF or DK. 请在此处对 CE029_1, CE029_2, CE029_3 或者 CE029_4 中回答不知道或拒绝回答的添加分级展开问题（100/200/400/800/1600 元）。

CE072_W3 Including both giving to you and receiving from you, did any of your non-coresident siblings have economic supports with you or your spouse? 过去一年，您或您的配偶从以下哪一些兄弟姐妹那里收到过或给过经济支持？

1. Sibling's name 兄弟姐妹姓名
2. Sibling's name 兄弟姐妹姓名

⋮

30. Sibling's name 兄弟姐妹姓名
99. None 以上都没有 → Skip to CE016_W3

PROCEDURE 程序:

Repeat questions from CE072_W2 to CE075_W2 according to the selected names in CE072_W3 if the sibling is living and name is nonmissing. 对于活着且名字没有缺失的兄弟姐妹，根据 CE072_W3 中选中的名字重复询问 CE072_W2 到 CE075_W2。

CE072_W2 In the past year, how much economic supports did you or your spouse receive from your [sibling's name]? 过去一年，您或您的配偶从【兄弟姐妹姓名】那里收到过多少经济支持？

1. Total money support _____(**CE072_W2_1**) yuan, among which how much is regular _____(**CE072_W2_2**) yuan (for example, support living expenses/water and electricity/telephone rate/return loan or other cost in regular). 总共给钱 _____(**CE072_W2_1**) 元(给钱是指婚丧嫁娶、搬迁新房、新生儿、子女升学等情况下的随礼，以及生病、生活困难等情况下的经济资助等，但不包括借钱)，其中，定期给 _____(**CE072_W2_2**) 元（如定期提供生活费、支付每月水电费及电话费、支付房贷/房租费用或其他定期的费用）
2. Total in-kind support _____(**CE072_W2_3**) yuan, among which how much is regular _____(**CE072_W2_4**) yuan (for example, support food/vegetables/clothes or other in-kind support in regular). 总共给物 _____(**CE072_W2_3**) 元，其中，定期给物 _____(**CE072_W2_4**) 元（如定期提供粮食、买菜、买衣服或其他物品等）。

[IWER: Regular means supporting at fixed time such as per month/quarter of a year/half of a year/year, etc. 访员注意：定期是指按月、按季度、按半年、按年给钱或东西，时间上大致固定。]
[IWER: If give no money or in-kind support, please fill in "0"; if respondent answer RF or DK, then ask CE073_W2, or skip to CE074_W2. 访员注意：如果没有给钱或者没有给物，请填入"0"；拒绝回答或不知道的询问CE073_W2，否则跳至CE074_W2.

CE073_W2 Add unfolding brackets (100/200/400/800/1600 yuan) for CE072_W2. 请在此处对CE072_W2中回答不知道或拒绝回答的添加分级展开问题（100/200/400/800/1600 元）。

CE074_W2 In the past year, how much economic supports did you or your spouse provide to your [sibling's name]? 过去一年，您或您的配偶给【兄弟姐妹姓名】过多少经济支持？

1. Total money support _____(**CE074_W2_1**) yuan, among which how much is regular _____(**CE074_W2_2**) yuan (for example, support living expenses/water and electricity/telephone rate/return loan or other cost in regular). 总共给钱 _____ (**CE074_W2_1**) 元(给钱是指婚丧嫁娶、搬迁新房、新生儿、子女升学等情况下的随礼,以及生病、生活困难等情况下的经济资助等,但不包括借钱),其中,定期给 _____(**CE074_W2_2**) 元(如定期提供生活费、支付每月水电费及电话费、支付房贷/房租费用或其他定期的费用)
2. Total in-kind support _____(**CE074_W2_3**) yuan, among which how much is regular _____(**CE074_W2_4**) yuan (for example, support food/vegetables/clothes or other in-kind support in regular). 总共给物 _____(**CE074_W2_3**) 元，其中，定期给物 _____(**CE074_W2_4**) 元（如定期提供粮食、买菜、买衣服或其他物品等)。

[IWER: Regular means supporting at fixed time such as per month/quarter of a year/half of a year/year, etc. 访员注意：定期是指按月、按季度、按半年、按年给钱或东西，时间上大致固定。]
[IWER: If give no money or in-kind support, please fill in “0”; if respondent answer RF or DK, then ask CE075_W2, or skip to CE016_W3. 访员注意：如果没有给钱或者没有给物，请填入“0”；拒绝回答或不知道的询问CE075_W2，否则跳至CE016_W3。]

CE075_W2 Add unfolding brackets (100/200/400/800/1600 yuan) for CE074_W2. 请在此处对CE074_W2中回答不知道或拒绝回答的添加分级展开问题（100/200/400/800/1600元)。

CE016_W3 In the past year, how much cash gift did you or your spouse receive from your non-coresident other relatives or friends? 过去一年,您或您的配偶从您的没住在一起的其他亲戚朋友那里收到过多少礼金?Total money and in-kind support ______ yuan (for example, marriage and funeral/move to new house/new born/go to university, and economic aid for fall ill or difficult to live, but not including borrowing money). 总共给钱物 ______ 元(给钱是指婚丧嫁娶、搬迁新房、新生儿、子女升学等情况下的随礼)。

CE017_W3 Add unfolding brackets (100/200/400/800/1600 yuan) for CE016_W3. 请在此处对CE016_W3中回答不知道或拒绝回答的添加分级展开问题（100/200/400/800/1600元)。

CE036_W3 In the past year, how much cash gift did you or your spouse provide to your non-coresident other relatives or friends? 过去一年，您或您的配偶给您的没住在一起的其他亲戚朋友过多少礼金？Total money and in-kind support ______ yuan (for example, marriage and funeral/move to new house/new born/go to university, and economic aid for fall ill or difficult to live, but not including borrowing money). 总共给钱物 ______ 元（给钱是指婚丧嫁娶、搬迁新房、新生儿、子女升学等情况下的随礼)。

[IWER:If give no money nor in-kind support, please fill in “0”; if respondent answered RF or DK, please ask CE037_W3, or skip to CE066_W2BR. 访员注意：如果没有给钱物，请填入“0”；拒绝回答或不知道的询问CE037_W3，否则跳至 CE066_W2BR.]

CE037_W3 Add unfolding brackets (100/200/400/800/1600 yuan) for CE036_W3. 请在此处对CE036_W3中回答不知道或拒绝回答的添加分级展开问题（100/200/400/800/1600元）。

CE016 In the past year, how much economic supports did you or your spouse receive from your non-coresident other relatives or friends, excluding cash gift? 过去一年，除了收到的礼金之外，您或您的配偶从您的没住在一起的其他亲戚朋友那里收到过多少经济支持？Total money and in-kind support ______ yuan. (for example, marriage and funeral/move to new house/new born/go to university, and economic aid for fall ill or difficult to live, but not including borrowing money). 总共给钱物 ______ 元（给钱包括生病、生活困难等情况下的经济资助等，但不包括借钱。）

[IWER: If give no money nor in-kind support, please fill in “0”; if respondent answered RF or DK, please ask CE017, or skip to CE036. 访员注意：如果没有给钱物，请填入“0”；拒绝回答或不知道的询问CE017，否则跳至CE036.]

CE017 Add unfolding brackets (100/200/400/800/1600 yuan) for CE016. 请在此处对CE016中回答不知道或拒绝回答的添加分级展开问题（100/200/400/800/1600 元）。

CE036 In the past year, how much economic supports did you or your spouse provide to your non-coresident other relatives or friends,excluding cash gift? 过去一年，除了送出的礼金之外，您或您的配偶给您的没住在一起的其他亲戚朋友过多少经济支持？Total money and in-kind support ______ yuan. (for example, marriage and funeral/move to new house/new born/go to university, and economic aid for fall ill or difficult to live, but not including borrowing money). 总共给钱物 ______ 元（给钱包括生病、生活困难等情况下的经济资助等，但不包括借钱）。

[IWER: :If give no money nor in-kind support, please fill in “0”; if respondent answered RF or DK, please ask CE037, or skip to CE066_W2BR. 访员注意：如果没有给钱物，请填入 “0”；拒绝回答或不知道的询问CE037， 否则跳至 CE066_W2BR.]

CE037 Add unfolding brackets (100/200/400/800/1600 yuan) for CE036. 请在此处对CE036中回答不知道或拒绝回答的添加分级展开问题（100/200/400/800/1600 元）。

CE066_W2 BRANCHPOINT:

IF [CHILD'S NAME] IS NOT UNMARRIED (CB063 = 1/2/3/4/5/7), ASK CE066_W2

[Preload each child that is not unmarried. 加载每一个非未婚的子女。]

[Preload the information of them, if there is none, then ask; otherwise, then skip. 加载已婚子女信息，如果为空的再询问，不为空的就跳过不再询问。]

[IWER: please ask about each child CE066_W2 - CE070_W2_1. 访员注意：询问每一个非未婚的子女CE066_W2 - CE070_W2_1。]

[IWER: If the child got married more than one times, please ask about the first one. 访员注意：如果子女结婚次数大于 1 次，则询问第 1 次结婚的情形。]

CE066_W2 When did your [childn's name] get married? [孩子姓名] 是什么时候结婚的?
____1900...2015 (**CE066_W2_1**) year 年 __0...12 (**CE066_W2_2**) month 月 __0...31 (**CE066_W2_3**) day 日

CE067_W2_1 Did you give betrothal gifts when [child name] got married? [孩子姓名] 结婚时您给彩礼了吗?
1. Yes 是
2. No 否 → Skip to CE069_W2_1 跳至CE069_W2_1

CE068_W2_1 At that time, how much was the total value of the betrothal gifts? ____ yuan. 在当时的物价水平下，这些彩礼值总共值多少钱? ____ 元。

CE069_W2_1 Did you buy a house for him/her when [child name] got married? [孩子姓名] 结婚时您给买房了吗?
1. Yes 是
2. No 否 → Skip to procedure before CF001 跳至 CF001 之前程序

CE070_W2_1 At that time, how much was the total value of the house? ____10000 yuan. 在当时的物价水平下，这房子值多少钱? ____ 万元。

CF TIME SPENT PROVIDING CARE 提供照料时间

PROCEDURE 程序:
If the respondent has any grandchildren, ask CF001 - CF003; otherwise, skip to CF004. 如果受访者有孙子/孙女，提问CF001 - CF003；否则，请跳至CF004 .

CF001 Did you spend any time taking care of your grandchildren last year? 过去一年，您或您配偶是否花时间照看了您的孙子女?
1. Yes 是
2. No 否 → Skip to the procedure before CF004 跳至CF004之前的程序

CF002 For which child's children did you provide care? 您或您配偶照看哪一个子女的孩子?
1. Child's name 子女姓名
2. Child's name 子女姓名
⋮
25. Child's name 子女姓名
99. 以上都没有 → Skip to CE074_W3

[IWER: Please list all the children including coresident ones, and add a choice "deceased children" in CAPI list. Select from list displayed by CAPI (child's name) 访员注意：请列出所有人，包括在一起住的孩子和去世的子女名单，从孩子名单中选出]

PROCEDURE 程序:
Repeat question CF003 according to the list of names in CF002. 根据CF002 的名单中名字重复问 CF003。

CF003 Approximately how many weeks and how many hours per week did you spend last year taking care of this child’s children? 过去一年，您和您配偶大约花几周，每周花多少时间来照看这个子女的孩子？Myself 我 ____(**CF003_1**)weeks 周；____(**CF003_2**) hours per week 小时/周；My spouse 我爱人 ____(**CF003_3**) weeks 周；____(**CF003_4**) hours per week 小时/周

[IWER: Mark ’1’ if the period is less than 7 days. 访员注意：如果小于 7 天填写“1”]

[Hardcheck: if more than 52 weeks are reported or more than 140 hours are reported. 如果答案大于 52 周或者大于 140 小时。]

PROCEDURE 程序:

If both parents were not living at last IW or both of them are household members, or one parent were not living at last IW and the other is a household member, go to CF007_W2.

CF004 Did you or your spouse take care of your parents or parents-in-law during the last year in assisting them in their daily activities or other activities (e.g., household chores, meal preparation, laundry, going out, grocery shopping, financial management, etc.)? 过去一年，您或您配偶有没有在日常活动（或其他活动）方面给您父母或您配偶的父母提供帮助(例如家务劳动，做饭，洗衣，外出，购物和财务管理)？

1. Yes 是
2. No 否 → Skip to next module 跳至下一个模块

CF005 Approximately how many weeks and how many hours per week did you yourself spend last year taking care of your parents or parents-in-law? 过去一年，您自己大约花几周，每周花多少时间来照看您的父母和岳父母（或公公婆婆)？

1. Your father 您父亲 __(**CF005_1**) weeks 周; ___(**CF005_2**) hours per week 小时/周
2. Your mother 您母亲 __(**CF005_3**) weeks 周; ___(**CF005_4**) hours per week 小时/周
3. Your father-in-law 您的岳父（或公公） __(**CF005_5**) weeks 周; ___(**CF005_6**) hours per week 小时/周
4. Your mother-in-law 您的岳母（或婆婆） __(**CF005_7**) weeks 周; ___(**CF005_8**) hours per week 小时/周

[Softcheck: if (1) is checked in CF004 and 0 or missings are reported in CF005. 如果CF004 = 1，而且CF005 = 0 或答案缺失]

[Softcheck: if more than 52 weeks are reported or more than 140 hours are reported. 如果答案大于 52 周或者大于 140 小时]

CF006 Approximately how many weeks and how many hours per week did your spouse spend last year taking care of your parents or parents-in-law? 过去一年，您的配偶大约花几周，每周花多少时间来照看您的父母和岳父母（或公公婆婆)？

1. Your father 您父亲 __(**CF006_1**) weeks 周; ___(**CF006_2**) hours per week 小时/周
2. Your mother 您母亲 __(**CF006_3**)weeks 周; ___(**CF006_4**) hours per week 小时/周
3. Your father-in-law 您的岳父（或公公） __(**CF006_5**) weeks 周; ___(**CF006_6**) hours per week 小时/周

4. Your mother-in-law 您的岳母（或婆婆）__(**CF006_7**) weeks 周; ___(**CF006_8**) hours per week 小时/周

[Softcheck: if (1) is checked in CF004 and 0 or missings are reported in CF006. 如果CF004 = 1，而且CF006 = 0 或答案缺失]

[Softcheck: if more than 52 weeks are reported or more than 140 hours are reported. 如果答案大于 52 周或者大于 140 小时]

CF007_W2 How often did the respondent receive assistance in answering sections CB, CD, CE and CF? 受访者填写 CB, CD, CE 和 CF 部分问卷时是否求助?

[IWER: If it is answered by a proxy, please record the respondent's reaction. If the Family R isn't in or can't answer questions, and proxyed by his/her spouse, please choose the (4). 访员注意：如果是协助回答，请记录受访者的反应。如果家庭受访者不在家或者无法回答问题，而是由其配偶回答的，请选择（4）。]

1. Never 从未
2. A few times 偶尔几次
3. Most or all of the time 大多数
4. Proxy by the spouse. 由家庭受访者的配偶代理回答

A HOUSEHOLD MEMBER 家户成员

[INTRO: Relatives can have important effects on your life. We'd like to ask you some questions about other members of your household. 引语：您的家人对您的生活可能会有重要影响，因此我们想问问除了您和您配偶外的其他家户成员的情况。] 家户成员的界定：与您长期分开居住的（一般为一个月以上）不属于家户成员；由于工作等原因在外居住但是每个周末或者每个月都回家的、共享生活费用的算是家户成员。]

A001_W3 Whom do you live together? (preload all the names of spouse/parents/stepparents/children/sib

choose all that apply. 您现在跟谁一起住？加载系统里配偶、父母、岳父母、子女、兄弟姐妹的姓名，可多选。

以下哪些成员现在跟你住在一起或者过去一年跟你住在一起的时间超过了 11 个月?

1. Spouse 加载配偶的姓名
2. My parents or parents in law 我和配偶的父母
3. Children 子女
4. My Siblings 我的兄弟姐妹
5. My spouse's Siblings 我配偶的兄弟姐妹
6. None of the above 以上都没有

PROCEDURE 程序:

- If answer to (**A001_W3**) is "My parents or parents in law", please ask A001_W3_0 如果回答" 我和配偶的父母"，询问A001_W3_0

A001_W3_0 Which parents? 那些父母？（加载所有的自己和配偶的父母的信息）

PROCEDURE 程序:
• If answer to (**A001_W3**) is "Children", please ask A001_W3_1 如果回答" 子女"，询问A001_W3_1

A001_W3_1 Which children? 那些子女？（加载所有子女的信息）

PROCEDURE 程序:
• If answer to (**A001_W3**) is "My Siblings", please ask A001_W3_2 如果回答" 我的兄弟姐妹"，询问A001_W3_2

A001_W3_2 Which siblings? 那些兄弟姐妹？（加载所有我的兄弟姐妹的信息）

PROCEDURE 程序:
• If answer to (**A001_W3**) is "My spouse's Siblings", please ask A001_W3_3 如果回答" 我配偶的兄弟姐妹"，询问A001_W3_3

A001_W3_3 Which siblings? 那些兄弟姐妹？（加载所有我配偶的兄弟姐妹的信息）

A002_W3 Except the ones in A001_W3, any other people do you live together now?How many? ____ 0...10 (Preload the household members name in our system in 2011 and 2013) choose all that apply. 除了上述的人们以外，您现在还跟谁住在一起？（加载 2011 年和 2013 年系统里的家户成员名单）一共有几个？ ____ (**A002_W3**)
If answer to A002_W3 is larger than 0，record their names. 如果A002_W3的答案大于0，则记录所有同住人的姓名 ____ (**A002_W3_1_1**)

A004_W3 How many months did you live together in last year? ____ month (Preload all the names, ask each one) 过去一年，一起住了 ____ 月（加载所有的姓名，依次询问）

PROCEDURE 程序:
• Ask each new one in A002_W3 依次询问A002_W3里的每个新增加的人

A005_W3 Gender of [household member name] 【家户成员姓名】的性别是？
1. Male 男
2. Female 女

A006_W3 When was [household member name] born? 【家户成员姓名】的出生日期是？ ____ (**A006_W3_1**) 1900...2013 (HBirthyear) Year 年 ____ (**A006_W3_2**) 0...12 (HBirthmonth) Month 月
Is it lunar or solar? 您回答的是公历还是农历？(**A006_W3**)
1 公历 (阳历)
2 农历 (阴历)

A006_W3_1 How long have you been lived with [household member name]？过去一年，你和【家户成员姓名】一起住了几个月？____ 0...12

A006 What is the relationship of [household member name] to you? 【家户成员姓名】是您的？

1. Mother 母亲
2. Father 父亲
3. Mother-in-law 岳母/婆婆
4. Father-in-law 岳父/公公
5. Sibling 兄弟姐妹
6. Brother-in-law, sister-in-law 姐夫妹夫/嫂子弟媳
7. Child 孩子
8. Spouse of child 儿媳/女婿
9. Grandchild 孙子女
10. Nanny 保姆
11. Driver 司机
12. Other relative（specify）其他亲戚（请注明）____(**A006_1**)

D HEALTH STATUS AND FUNCTIONING 健康状况和功能

Type of Interview R 受访者类型		
XRType = REIW	This is a reinterview R	受访者类型为回访受访者
XRType = NEWIW	This is a new interview R	受访者类型为新受访者
XRType = EXITIW	This is a exit R	受访者类型为退出受访者

Gender of Interview R 受访者性别		
R IS MALE	(XRGender = 1)	受访者为男性 (XRGender = 1)
R IS FEMALE	(XRGender = 2)	受访者为女性 (XRGender = 2)

R's LAST IW Time (ZIWTime) 上次访问的日期 (ZIWTime)

HEALTH CONDITIONS REPORTED IN LAST WAVE INTERVIEW: 基线访问时受访者报告的健康状况

ZDA005[i]	IF ZDA005[i]=Yes, R had kind of disabilities listed in DA005 at ZIWTime
ZDA006[i]	IF ZDA006[i]=Yes, R reported disabled time at ZIWTime
ZDA007[1]	IF ZDA007[1]=Yes, R had hypertension at ZIWTime
ZDA007[2]	IF ZDA007[2]=Yes, R had dyslipidemia at ZIWTime
ZDA007[3]	IF ZDA007[3]=Yes, R had diabetes or high blood sugar at ZIWTime
ZDA007[4]	IF ZDA007[4]=Yes, R had cancer or malignant tumor (excluding minor skin cancers) at ZIWTime
ZDA007[5]	IF ZDA007[5]=Yes, R had chronic lung diseases, such as chronic bronchitis, emphysema at ZIWTime
ZDA007[6]	IF ZDA007[6]=Yes, R had liver disease at ZIWTime
ZDA007[7]	IF ZDA007[7]=Yes, R had heart attack, coronary heart disease, angina, congestive heart failure, or other heart problems at ZIWTime
ZDA007[8]	IF ZDA007[8]=Yes, R had stroke at ZIWTime
ZDA007[9]	IF ZDA007[9]=Yes, R had kidney disease at ZIWTime
ZDA007[10]	IF ZDA007[10]= Yes, R had stomach or other digestive disease (except for tumor or cancer) at ZIWTime
ZDA007[11]	IF ZDA007[11]= Yes, R had emotional, nervous, or psychiatric problems at ZIWTime
ZDA007[12]	IF ZDA007[12]= Yes, R had memory-related disease at ZIWTime
ZDA007[13]	IF ZDA007[13]= Yes, R had arthritis or rheumatism at ZIWTime
ZDA007[14]	IF ZDA007[14]= Yes, R had asthma at ZIWTime
ZDA008[1]	IF ZDA008[1]= Yes, R had known R had hypertension at ZIWTime

ZDA008[5]	IF ZDA008[5]= Yes, R had known R had chronic lung diseases at ZIWTime
ZDA008[11]	IF ZDA008[11]= Yes, R had known R had emotional problems at ZIWTime
ZDA009[i]	When was the condition first dignosed or known by yourself?
ZDA027	IF ZDA027=Yes, R had started menopause at R's last interview
ZDA028	Time of menopause
ZDA036	IF ZDA036= 1, R had cataract surgery<br>for one eye at ZIWTime
ZDA037	IF ZDA037=Yes, R had Glaucoma at ZIWTime
ZDA038	IF ZDA038=Yes, R had ever wear hearing aid at ZIWTime
ZDA040	IF ZDA040=Yes, R had lost all teeth at ZIWTime
ZDA059	IF ZDA059=Yes, R had ever smoked at R's last interview
ZDA065	At what age did you start to smoke on a regular basis?

NOTE: NOTE ON PRELOADED HEALTH CONDITIONS:
IN THIS SECTION MUCH OF THE FLOW OF THE INTERVIEW AND THE PHRASING OF THE QUESTIONS DEPENDS ON WHETHER THE RESPONDENT REPORTED HAVING CERTAIN HEALTH CONDITIONS IN A PREVIOUS IW AND/OR CONFIRMED THEM IN THE LAST IW
这一模块大部分的流量和问题的问法取决于受访者在上一期访问时报告的身体状况。

DA HEALTH STATUS 健康状况

PROCEDURE 程序
SKIP PATTERN CHECKPOINT: SELF-REPORTED HEALTH STATUS
TWO SCALES ARE USED TO MEASURE SELF-REPORTED HEALTH STATUS. R WILL BE ASKED TO RATE THEIR HEALTH STATUS TWICE, ONCE AT THE BEGINNING OF THIS SECTION AND AGAIN AT THE END OF THE SECTION. QUESTION ORDER WILL BE ASSIGNED RANDOMLY.

IF R IS RANDOMLY ASSIGNED TO ORDER 1(SEC_DA_LIST= 1), SKIP TO DA001。
IF R IS RANDOMLY ASSIGNED TO ORDER 2(SEC_DA_LIST= 2), SKIP TO DA002。

跳转检查：自我报告健康状况
在这个部分中，我们将要求受访者完成两次自我健康状况评价。第一次是在这个部分的开头；第二次则放在这个部分的结尾处。在问卷中自我评价的问题以随机的形式出现。

如果受访者被随机分在第一组（DA 部分 = 1），跳至 DA001。
如果受访者被随机分在第二组（DA 部分 = 2），跳至 DA002。

PART I: GENERAL HEALTH STATUS AND DISEASE HISTORY 第一部分：一般健康状况和疾病史

DA001 Next, I have some questions about your health. Would you say your health is excellent, very good, good, fair, or poor? 接下来我们问一些与您健康状况相关的问题。您觉得您

的健康状况怎么样？是极好，很好，好，一般，还是不好？[IWER：Interviewer should read all the following options 访员注意：必须读出所有的选项]

1. Excellent 极好
2. Very good 很好
3. Good 好
4. Fair 一般
5. Poor 不好

DA002 Next, I have some questions about your health. Would you say your health is very good, good, fair, poor or very poor? 下面我将问到一些关于您的健康状况的问题。您认为您的健康状况怎样？是很好，好，一般，不好，还是很不好？[IWER：Interviewer should read all the following options 访员注意：必须读出所有的选项]

1. Very good 很好
2. Good 好
3. Fair 一般
4. Poor 不好
5. Very poor 很不好

PROCEDURE 程序:

If XRType = REIW, ASK DA002_W2_1 如果是回访受访者，询问DA002_W2_1

DA002_W2_1 Compared with your health when we talked with you in R's LAST IW MONTH, YEAR, would you say that your health is better now, about the same, or worse? 与上一次访问时 [加载日期] 您的健康相比，您觉得您的健康状况变好了，差不多，还是变差了？

1. Better 变好
2. About the same 差不多
3. Worse 变差

DA005 BRANCHPOINT:

IF XRType = REIW WHO DID HAVE DISABILITY IN LAST IW TIME (ZDA005[i]=yes) AND (ZDA006[i]≠null), GO TO DA007 如果是上期回答有此项残疾的回访受访者且告知了患病时间，跳至DA007。

IF XRType = REIW WHO DID HAVE DISABILITY IN LAST IW TIME (ZDA005[i]=yes) AND (ZDA006[i]=null), GO TO DA006 如果是上期回答有此项残疾的回访受访者且没有告知患病时间，跳至 DA006

IF THIS IS A NEW INTERVIEW R OR THIS IS A REINTERVIEW R WHO DID NOT REPORT YES IN LAST WAVE (ZDA005[i]≠yes) 如果是新的受访者或上期回答没患有此项残疾的回访受访者询问

DA005 Do you have one of the following disabilities? 您是否有下列残疾问题？

1. Physical disabilities 躯体残疾
2. Brain damage/mental retardation 大脑受损/智力缺陷
3. Vision problem 失明或半失明

4. Hearing problem 聋或半聋
5. Speech impediment 哑或严重口吃

DA006 BRANCHPOINT:
If DA005[i] = 1, ask DA006 如果受访者回答患有残疾问题，询问DA006

DA006 In what year did you become disabled? 您是什么时候开始患有 [preload DA005]?

If XRType = REIW, ask: Our records from your last interview in R's LAST IW MONTH, YEAR show that you have had [preload ZDA005], In what year did you become disabled? 如果是回访受访者，询问：上一次的访问记录显示您患有 [...]，您是什么时候开始患有 [preload ZDA005]? _____Year 年

[IWER: Mark the year using four digits. 访员注意：用 4 位数表示年]

[IWER: If the records from last wave is wrong, please note -9999. 访员注意：如果从未患有此项残疾，请填-9999]

PROCEDURE 程序:
IF XRType = NEWIW, ask DA007 如果是新的受访者，询问DA007

DA007 Have you been diagnosed with [conditions listed below, read one by one] by a doctor? 是否有医生曾经告诉过您有以下这些慢性病?

1. Hypertension 高血压病
2. Dyslipidemia (elevation of low density lipoprotein, triglycerides (TGs),and total cholesterol, or a low high density lipoprotein level) 血脂异常（高血脂或低血脂）
3. Diabetes or high blood sugar 糖尿病或血糖升高（包括糖耐量异常和空腹血糖升高）
4. Cancer or malignant tumor (excluding minor skin cancers) 癌症等恶性肿瘤（不包括轻度皮肤癌）
5. Chronic lung diseases, such as chronic bronchitis , emphysema (excluding tumors, or cancer) 慢性肺部疾患如慢性支气管炎或肺气肿、肺心病（不包括肿瘤或癌）
6. Liver disease (except fatty liver, tumors, and cancer) 肝脏疾病（除脂肪肝、肿瘤或癌外）
7. Heart attack, coronary heart disease, angina, congestive heart failure, or other heart problems 心脏病（如心肌梗塞、冠心病、心绞痛、充血性心力衰竭和其他心脏疾病）
8. Stroke 中风
9. Kidney disease (except for tumor or cancer) 肾脏疾病（不包括肿瘤或癌）
10. Stomach or other digestive disease (except for tumor or cancer) 胃部疾病或消化系统疾病（不包括肿瘤或癌）
11. Emotional, nervous, or psychiatric problems 情感及精神方面问题
12. Memory-related disease 与记忆相关的疾病（如老年痴呆症、脑萎缩、帕金森症）
13. Arthritis or rheumatism 关节炎或风湿病
14. Asthma 哮喘

NOTE: The screen displays whether or not this condition was reported in R's LAST IW 屏幕上显示受访者上期访问时是否有慢性病

DA007_W2_1 Our records from your last interview in R's last IW month, year show that you have had/not had [conditions listed below], is this right? 上一次的访问记录显示您患有/没有 [加载慢性病], 这个记录对吗?

1. Agree 上次访问记录正确
2. Disagree 上次访问记录错误

PROCEDURE 程序:

For R reported in last iw that he/she had [conditions listed below] (ZDA007[i] = Yes or ZDA008[i] = Yes) 受访者上次访问时报告患有 [加载慢性病]

If DA007_W2_1 = 1, Skip to next loop 如果上次访问正确, 询问下一个慢性病

If DA007_W2_1 = 2, Skip to DA007_W2_2 如果上次访问错误, 询问上次访问以后是否患有此项慢性病DA007_W2_2

PROCEDURE 程序:

For R reported in last iw that he/she did not have [conditions listed below],(ZDA007[i] $\neq$ yes and ZDA008[i] $\neq$ yes) 受访者上次访问时报告没有患有 [加载慢性病]

If DA007_W2_1 = 1, Skip to DA007_W2_2 如果上次访问正确, 询问上次访问以后是否患有此项慢性病DA007_W2_2

If DA007_W2_1 = 2, Skip to DA008_W2_1 如果上次访问错误, 说明上次访问已经患有此项慢性病, 询问何种方式知道DA008_W2_1

DA007_W2_2 Have you been diagnosed with [conditions listed below, read one by one] by a doctor [since R's LAST IW MONTH, YEAR/ in the last two years]? 自上次访问以后 (过去两年), 是否有医生曾经告诉过您有以下这些慢性病?

1. Yes 是 Skip to DA008_W2_1 跳至DA008_W2_1
2. No 否 Skip to next loop 开始下一个慢性病的循环

DA008 BRANCHPOINT:

For XRType = REIW and XRType = NEWIW, ask DA008 if $i = 1, 5, 11$, and DA007[i] $\neq 1$ or DA007_W2_2[i] $\neq 1$. 对于每个DA007和DA007_W2_2的第 1, 5, 11 选项中没有回答是的选项, 问DA008

DA008 Do you know if you have [preload the current choice in DA007]? 您是否知道自己患有? [依次显示所有DA007中第 1, 5, 11 选项中回答否的选项的疾病名称]

1. Yes 知道自己患有
2. No 知道自己没有
3. Don't know 不知道有没有

PROCEDURE 程序:

If DA007[i] = 1 or DA007_W2_2[i] = 1 or DA008[i] = 1 如果医生诊断或自我诊断患有慢性病

DA008_W2_1 How did you know that you had had [preload disease], through routine or charls physical examination, or any other? 您是通过何种方式知道自己患有 [加载慢性病] 的, 是生病后体检, [加载慢性病] 发作后体检, 常规体检, charls 体检还是其他方式?

1. Physical examination after had [preload disease] attack [加载慢性病] 发作后体检
2. Physical examination after had ill 生病后体检
3. Physical examination organized by work unit 单位组织的体检
4. Physical examination organized by community 社区组织的体检
5. Charls physical examination charls 体检
6. Other, please specify 其他方式，请注明_____(**DA008_W2_1_1**)

DA009 BRANCHPOINT:

For XRType = REIW and XRType = NEWIW, if DA007[i]= 1 or DA007_W2_2[i] = 1 or DA008[i]= 1 or (((ZDA007[i] = 1 or ZDA008[i] = 1) and DA007_W2_1 = 1) and ZDA009[i]= null) or (ZDA007[i] ≠ 1 and ZDA008[i] ≠ 1 and DA007_W2_1 = 2), ask DA009 如果医生诊断或自我诊断患有慢性病,但不知道第一次诊断出慢性病的时间,询问DA009

DA009 When was the condition first diagnosed or known by yourself? 第一次诊断出或者自己知道患有 [...] 在_____(**DA009_1**) Year 年或_____(**DA009_2**) Age 岁？[IWER: Mark the year using four digits 访员注意：用 4 位数记录年份]

PROCEDURE 程序:

Answer DA010 if you have No. 2, 5, 6, 7, 9, 10, 12, 13 chronic dieases of DA007 如果医生诊断或自己知道患有DA007中第 2，5，6，7，9，10，12，13 项慢性病，回答DA010

DA010 Are you now taking any of the following treatments to treat [...] or its complications (Check all that apply)? Taking Chinese traditional medicine, taking Western modern medicine, other treatments? 您目前有没有正在采用以下方式来治疗 [...] 及其并发症 (选择所有采用的方式)？服用中药，服用西药，吃药以外的其他治疗方法？（可多选）[IWER: Read one by one 访问员注意：请逐项读出以下答案，并让受访者逐一回答]

1. Taking Chinese traditional medicine 服用中药
2. Taking Western morden medicine 服用西药
3. Other treatments 吃药以外的其他治疗方法
4. None of the above 以上都没有

PROCEDURE 程序:

Answer DA010_W2_1 if you have hypertension or diabetes 如果医生诊断或自己知道患有高血压或糖尿病，回答DA010_W2_1

DA010_W2_1 Is your [Blood pressure/ sugar] generally under control? 您的 [血压/血糖] 现在是否控制住了？

1. Yes 是
2. No 否

PROCEDURE 程序:

IF XRType = REIW that had condition in last wave ((ZDA007[i] = 1 or ZDA008[i] = 1) and DA007_W2_1[i] = 1) or (ZDA007[i] ≠ 1 and ZDA008[i] ≠ 1 and DA007_W2_1[i] = 2) 如果是回访受访者，上次访问时的确患有 [...] 慢性病

DA010_W2_2 Compared to when we interviewed you in R's LAST IW MONTH, YEAR, is your condition better, about the same as it was then or worse? 与上次访问相比 (加载上期访问时间)，您的 [...] 好一些了，跟原来差不多还是更差？

1. Better 更好
2. Worse 更差
3. Same 与原来差不多

PROCEDURE 程序: If respondents have hypertension then answer DA011 如果医生诊断或自我诊断患有高血压，回答DA011。

DA011 Are you now taking any of the following treatments to treat or control your hypertension?(Check all that apply) Taking Chinese traditional medicine, taking Western modern medicine? 您目前有没有正在采用以下方式来治疗控制高血压？服用中药，服用西药？（可多选）[IWER: Read one by one 访问员注意：请逐项读出以下答案，并让受访者逐一回答]

1. Taking Chinese traditional medicine 服用中药
2. Taking Western modern medicine 服用西药
3. None of the above 以上都没有

DA011_W2_1 **PROCEDURE 程序**: If XRType = REIW 如果是回访受访者

Since R's LAST IW MONTH, YEAR, have you had your blood pressure checked by a doctor or nurse? 自上次访问以来 [加载上次访问日期]，是否曾有医生或护士给您检查血压？

PROCEDURE 程序: OTHERWISE:

Have you ever had your blood pressure checked by a doctor or nurse? 是否曾有医生或护士给您检查血压？

1. Yes 是
2. No 否 Skip to DA013 跳至DA013

DA011_W2_2 When did you last have it checked? 最近一次检查是什么时候？

Year 年_____(**DA011_w2_2_1**) Month 月_____(**DA011_w2_2_2**)

DA012 During last year (last 12 months), how many times have you had blood pressure examination? 在过去一年（12 个月）中，您检查过几次血压？ _____0...999 Times 次

DA012_W3 Have you had blood pressure examination by community/village doctors regularly? 是否有社区医生/村医经常为您检查血压？

1. Yes 是
2. No 否 Skip to DA013 跳至DA013

DA012_W3_1 How often did you have blood pressure examination by community/village doctors? 社区医生/村医多长时间为您检查一次血压？

1. Once a week 一周一次

2. Once half a month 半月一次
3. Once a month 一月一次
4. Once every two months 两月一次
5. Once every three months 一季度一次
6. Once half a year 半年一次
7. Once a year 一年一次

DA012_W3_2 Do you have to pay for the blood pressure examination by community/village doctors? 社区医生/村医为您检查血压需要付费吗?
1. Yes 需要
2. No 不需要

**PROCEDURE 程序**: If respondents have hypertension then answer DA013 如果医生诊断或自我诊断患有高血压，回答DA013。

DA013 Have your care providers ever given you health education/advice on the following (check all that apply)? Weight control, exercise, diet and/or smoking control? 有没有医生建议您注意以下问题?（可多选）控制体重，身体锻炼，饮食调理，控烟? [IWER: Read one by one 访问员注意：请逐项读出以下答案，并让受访者逐一回答]
1. Weight control 控制体重
2. Exercise 身体锻炼
3. Diet 饮食调理
4. Smoking control 控烟
5. None of the above 以上都没有

**PROCEDURE 程序**：If respondents have diabetes, then answer DA014-DA016 如果受访者患有糖尿病，回答DA014-DA016。

DA014 Are you now taking any of the following treatments to treat or control your diabetes? 您目前有没有正在采用以下方式来治疗控制糖尿病? 服用中药，服用西药，注射胰岛素?（可多选）[IWER: Read one by one 访问员注意：请逐项读出以下答案，并让受访者逐一回答]
1. Taking Chinese traditional medicine 服用中药
2. Taking Western modern medicine 服用西药
3. Taking insulin injections 注射胰岛素
4. None of the above 以上都没有

DA015 During last year (last 12 months), how many times have you had the following? 过去一年（12 个月）中，下列各种检测您分别做了几次?
1. Blood glucose test 血糖检测 **(DA015_1)**_____ 0...999 Times 次
2. Urine glucose test 尿糖检测 **(DA015_2)**_____ 0...999 Times 次
3. Fundus examination 眼底检查 **(DA015_3)**_____ 0...999 Times 次
4. Micro-albuminuria test 微蛋白尿检查 **(DA015_4)**_____ 0...999 Times 次
5. None of the above 以上都没有 Skip to DA016 跳至DA016

DA016_W3 Have you had diabetes examination by community/village doctors regularly? 是否有社区医生/村医经常为您做糖尿病检查?

1. Yes 是
2. No 否 Skip DA016_W3_1 and DA016_W3_2 跳过DA016_W3_1和DA016_W3_2

DA016_W3_1 How oftern did you have diabetes examination by community/village doctors? 社区医生/村医多长时间为您做一次糖尿病的检查?

1. Once a week 一周一次
2. Once half a month 半月一次
3. Once a month 一月一次
4. Once every two months 两月一次
5. Once every three months 一季度一次
6. Once half a year 半年一次
7. Once a year 一年一次

DA016_W3_2 Do you have to pay for diabetes examination done by community/village doctors? 社区医生/村医为您做糖尿病检查需要付费吗?

1. Yes 需要
2. No 不需要

DA016 Have your care providers ever given you health education/advice on the following? (check all that apply) 有没有医生建议您注意以下问题? 控制体重，身体锻炼，饮食调理，控烟，足部自我护理?（可多选）[IWER: Read one by one 访问员注意：请逐项读出以下答案，并让受访者逐一回答]

1. Weight control 控制体重
2. Exercise 身体锻炼
3. Diet 饮食调理
4. Smoking control 控烟
5. Foot self-care 足部自我护理
6. None of the above 以上都没有

**PROCEDURE 程序**: If reinterview respondents have heart attack, then answer DA007_W2_5 如果回访受访者患有心脏病，回答DA007_W2_5。

DA007_W2_5 [Since R's LAST IW MONTH, YEAR/In the last two years], have you had a heart attack? 自上次访问以来的两年内，您是否发作过心脏病?

1. Yes 是
2. No 否 → Skip DA007_W2_6 跳过DA007_W2_6

DA007_W2_6 When was [his/her] (most recent) heart attack? 最近一次发病是什么时候?

_____ Year 年 (**DA007_W2_6_1**) _____ Age 岁 (**DA007_W2_6_2**)

**PROCEDURE 程序**: If respondents have cancer or maligant tumor (excluding minor skin cancers), answer DA017 and DA018 如果受访者患有癌症或恶性肿瘤（不包括轻度皮肤癌），回答DA017 及 DA018。

DA017 In which organ or part of your body do you have cancer? Including the origins and metastasis of tumor.（circle all that apply）身体的哪个器官或部位患有或曾经患有癌症？包括原发和已转移的肿瘤。（可多选） [IWER: Read one by one. We should still ask R even if he/she has already been cured 访员注意：依次读出所有选项，若受访者所患癌症已痊愈，仍需记录]

1. Brain 大脑
2. Oral cavity 口腔
3. Larynx 喉
4. Other pharynx 咽
5. Thyroid 甲状腺
6. Lung 肺
7. Breast 乳房
8. Oesophagus 食管
9. Stomach 胃
10. Liver 肝脏
11. Pancreas 胰腺
12. Kidney 肾脏
13. Prostate 前列腺
14. Testicle 睾丸
15. Ovary 卵巢
16. Cervix 子宫颈
17. Endometrium 子宫内膜
18. Colon or rectum 结肠或直肠
19. Bladder 膀胱
20. Skin 皮肤
21. Non-Hodgkin lymphoma 非何杰金淋巴瘤（非霍奇金淋巴瘤）
22. Leukemia 白血病
23. Other organ 其他器官 (**DA017_1**)

DA018 Have you taken any of the following treatments to treat your cancer or relieve its/their symptoms (e.g., pain, nausea, etc.) in the past two years? (Check all that apply) Taking Chinese traditional medicine, taking Western modern medicine, chemotherapy, surgery, radiation therapy? 在过去的两年中，您有没有采用以下方式来治疗肿瘤或缓解肿瘤所引起的疼痛、恶心等症状（可多选）？服用中药，服用西药，化学疗法，手术治疗，放射疗法？ [IWER: Read one by one 访问员注意：请逐项读出以下答案，并让受访者逐一回答]

1. Taking Chinese traditional medicine 服用中药
2. Taking Western morden medicine 服用西药
3. Chemotherapy 化学疗法
4. Surgery 手术治疗
5. Radiation therapy 放射疗法
6. None of the above 以上都没有

[F1：（1）“化学疗法”指用化学合成药物治疗疾病的方法。化学药物治疗（简称化疗）是目前治

疗肿瘤及某些身免疫性疾病的主要手段之一。
(2)"手术治疗"是最早应用的治疗癌症的方法，也是目前许多早期癌症治疗的首选疗法。
(3)"放射疗法"是用各种不同能量的射线照射肿瘤，以抑制和杀灭癌细胞的一种治疗方法]

PROCEDURE 程序：If respondents have stroke, then answer DA019 如果受访者患有中风，回答DA019。

DA019 Are you now taking any of the folloing treatments because of your stroke?(Check all that apply) Taking Chinese traditional medicine, taking Western modern medicine, physical therapy, acupuncture and moxibustion, occupational therapy? 您目前有没有正在采用以下方式来治疗或控制由于中风引起的痉挛或者其它并发症？服用中药，服用西药，物理治疗，针灸治疗，职业治疗？（可多选）[IWER: Read one by one 访问员注意：请逐项读出以下答案，并让受访者逐一回答]

1. Taking Chinese traditional medicine 服用中药
2. Taking Western morden medicine 服用西药
3. Physical therapy 物理治疗
4. Acupuncture and moxibustion 针灸治疗
5. Occupational therapy 职业治疗
6. None of the above 以上都没有

[F1：(1)"物理治疗"指主要借着自然界中的物理因子（声光水冷电热力)、运用人体生理学原理法则等，针对人体局部或全身性的功能障碍或病变，施予适当的非侵入性、非药物性治疗来处理患者身体不适和病痛治疗方式，使其尽可能地恢复其原有的生理功能。
(2)"针灸治疗"指针法和灸法的合称。针法是把毫针按一定穴位刺入患者体内，运用捻转与提插等针刺手法来治疗疾病。灸法是把燃烧着的艾绒按一定穴位熏灼皮肤，利用热的刺激来治疗疾病。
(3)"职业治疗"指透过悉心选择而适用于病者的康复活动，帮助在精神、情绪、体能或智能上有障碍的人恢复/增强身体及心智功能，阻止/减轻伤残所带来的影向，使他们能适应工作，社会及家庭上的要求，达至正常和独立的生活]

PROCEDURE 程序：If reinterview respondents have stroke, then answer DA019_W2_1 and DA019_W2_2 如果回访受访者上次访问的确患有中风，询问DA019_W2_1和DA019_W2_2。

DA019_W2_1 Since R's LAST IW MONTH, YEAR, has a doctor told you that you had another stroke? [加载上次访问日期] 自上次访问以来，是否有医生诊断您中风复发？

1. Yes 是
2. No 否 Skip DA019_W2_2 跳过DA019_W2_2

DA019_W2_2 When was your most recent stroke? 最近一次中风的诊断时间是？

_____ Year 年_____ Age 岁

PROCEDURE 程序：If respondents have emotional, nervous, or psychiatric problems, then answer DA020 如果受访者患有精神、心理和情感方面的疾病，回答DA020

DA020 Are you now taking any of the folloing treatments for your emotional, nervous, or psychiatric problems?(Check all that apply) Receiving psychiatric or psychological treatment, taking anti depressants, taking tranquilizers or sleeping pills? 您目前有没有正在采用以下方式来治疗精神、心理和情感方面的疾病？（可多选）[IWER: Read one by one 访问员注意：请逐项读出以下答案，并让受访者逐一回答]

1. Receiving psychiatric or psychological treatment 接受精神科治疗或心理治疗
2. Taking anti depressants 服用抗抑郁药物
3. Taking tranquilizers or sleeping pills 服用镇静或安眠药物
4. None of the above 以上都没有

DA021 **PROCEDURE 程序**: If XRType = NEWIW 如果是新的受访者

Have you ever been in a traffic accident or any other kind of major accidental injury and received medical treatment? 您是否经历过交通事故，或任何的重大意外伤害，并接受了治疗？

PROCEDURE 程序: If XRType = REIW 如果是回访受访者

Have you ever been in a traffic accident or any other kind of major accidental injury and received medical treatment [since R's LAST IW MONTH, YEAR/ in the last two years]? 自上次访问以来 (加载上次访问日期), 您是否经历过交通事故，或任何的重大意外伤害，并接受了治疗？

1. Yes 是
2. No 否 → Skip to DA023 跳至 DA023

DA022 Does your injury caused by the accident limit your daily activities? 事故导致的伤害是否影响您现在的日常活动？

1. Yes 是
2. No 否

DA023 **PROCEDURE 程序**: If XRType = NEWIW 如果是新的受访者

Have you fallen down? 您有没有摔倒过？

PROCEDURE 程序: If XRType = REIW 如果是回访受访者

Have you fallen down since R's last IW month, year? 自上次访问以来 [加载上次访问日期]，您有没有摔倒过？

1. Yes 是
2. No 否 → Skip to DA025 跳至 DA025

DA024 How many times have you fallen down seriously enough to need medical treatment? 有多少次摔倒受伤严重到需要接受治疗?_____ times 次

DA025 **PROCEDURE 程序**: If XRType = NEWIW 如果是新的受访者

Have you ever fractured your hip? 您有没有过髋骨骨折？

PROCEDURE 程序: If XRType = REIW 如果是回访受访者

Have you fractured your hip since we talked in R's last IW month, year? 自上次访问以来 [加载上次访问日期]，您有没有过髋骨骨折？

1. Yes 是
2. No 没有

[F1:“髋骨”指人体腰部的骨骼，共左右两块。幼年时，髋骨分为髂骨，坐骨和耻骨以及软骨连接。成年后，它们之间的软骨会骨化，成为一个整体，即髋骨]

**PROCEDURE 程序**: IF R IS MALE, SKIP TO DA029 男性跳至DA029。

BRANCHPOINT:

IF XRType = REIW reported in previous wave that has not started menopause, skip to DA027 如果是回访受访者并且没有绝经，跳至DA027题

IF XRType = REIW reported in previous wave that has started menopause, skip to DA032 如果回访受访者已绝经，跳至DA032题

IF XRType = NEWIW 如果是新的受访者，询问DA026-DA028 题

DA026 When did you begin the menarche? 您什么时候开始来月经的？

_____ 1900...2015 (**DA026_1**) Year 年 Or 或 Age 年龄_____ 1...120 (**DA026_2**) Years 岁

[IWER: Mark the year using four digits 访员注意：用 4 位数表示年]

DA027 Have you started menopause? 您绝经了吗？

1. Yes 是
2. No 没有 → Skip to DA032 跳至 DA032

DA028 When did you begin the menopause? 您什么时候开始绝经的？

_____ (**DA028_1**) Year 年 Or 或 Age 年龄_____ (**DA028_2**) Years 岁 → Skip to DA032 跳至 DA032 [IWER: Mark the year using four digits 访员注意：用 4 位数表示年]

DA029 **PROCEDURE 程序**: If XRType = NEWIW and is male 如果是新的男性受访者

Have you ever been diagnosed with a prostate illness, such as prostate hyperplasia (excluding prostatic cancer) ? 有没有医生诊断您得了前列腺疾病如前列腺增生（排除前列腺癌）？

PROCEDURE 程序: If XRType = REIW and is male 如果是回访男性受访者

Have you ever been diagnosed with a prostate illness, such as prostate hyperplasia (excluding prostatic cancer) since we talked (in R's LAST IW MONTH,YEAR/in the last two years)? 上一次访问之后（过去两年），有没有医生诊断您得了前列腺疾病如前列腺增生（排除前列腺癌）？

1. Yes 是 → Skip to DA030 跳至 DA030
2. No 没有

[F1:“前列腺增生”主要症状有排尿困难，轻者夜里起床小便次数增多，有尿不净或尿完后还有少量排出的现象；严重者出现尿流变细，甚或排不出的现象；同时常伴有腰酸腰痛、四肢无力、遗精等症状]

DA029_W2_1 Do you know if you had a prostate illness, such as prostate hyperplasia (excluding prostatic cancer) ? 您是否知道自己患有前列腺疾病如前列腺增生（排除前列腺癌）？

1. Yes 知道自己患有
2. No 知道自己没有 → Skip to DA032 跳至 DA032
3. Don't know 不知道有没有 → Skip to DA032 跳至 DA032

DA030 When was the condition first diagnosed? 第一次诊断出您有前列腺疾病是在什么时候？ _____ (**DA030_1**)Year 年 Or 或 Age 年龄_____ (**DA030_2**) Years 岁 [IWER: Mark the year using four digits 访员注意：用 4 位数表示年]

DA031 Are you now taking medication or other treatment for your prostate illness? 您目前是否正在服药或以其他方式治疗前列腺疾病？

1. Yes 是
2. No 否

DA032 Now I have some questions about your eyesight. Do you usually wear glasses or corrective lenses? 下面问您一些关于视力的问题。您通常是否戴眼镜（包括矫正视力镜片）？

1. Yes 是
2. Legally blind 失明 → Skip to DA038 跳至 DA038
3. No 否
4. Sometimes 偶尔

DA033 How good is your eyesight for seeing things at a distance, like recognizing a friend from across the street (with glasses or corrective lenses if you wear them)? Would you say your eyesight for seeing things at a distance is excellent, very good, good, fair, or poor? 您看远处的东西怎么样？比如说能不能隔着马路认出朋友（包括戴着眼镜）。是极好，很好，好，一般还是不好？

1. Excellent 极好
2. Very good 很好
3. Good 好
4. Fair 一般
5. Poor 不好

DA034 How good is your eyesight for seeing things up close, like reading ordinary newspaper print (with glasses or corrective lenses if you wear them)? Would you say your eyesight for seeing things up close is excellent, very good, good, fair, or poor? 您看近处的东西怎么样？比如说戴着眼镜能不能看报纸？是极好，很好，好，一般还是不好？

1. Excellent 极好
2. Very good 很好
3. Good 好
4. Fair 一般
5. Poor 不好

**PROCEDURE 程序**： If XRType = NEWIW 如果是新的受访者：

DA035 Have you ever had cataract surgery? 您是否做过白内障手术?

1. Yes 是
2. No 否 → Skip to DA037 跳至 DA037

**PROCEDURE 程序**: If XRType = REIW and R reported in last IW that had cataract surgery on one eye (ZDA036 = 1) 如果是回访受访者，上期访问回答一只眼睛做过白内障手术（ZDA036 = 1）:

DA035_W2_1 Have you had another cataract surgery since we last talked to you (in R's LAST IW MONTH, YEAR) other than what you told us about then? 上次访问记录显示您一只眼睛做过白内障，（加载上次访问日期）之后（过去两年）您的另一只眼睛是否做过白内障手术?

1. Yes 是
2. No 否

**PROCEDURE 程序**: If XRType = REIW and R did not report in last IW that had cataract surgery ((ZDA035 ≠ 1) 如果是回访受访者，上期访问回答没有做过白内障手术:

DA035_W2_2 Have you had cataract surgery (since R's LAST IW MONTH, YEAR/in the last two years)? （加载上次访问日期）自上次访问以来，您是否做过白内障手术?

1. Yes 是
2. No 否 → Skip to DA037 跳至 DA037

DA036 Have you had cataract surgery on both eyes or just one? 白内障手术是做的一只眼还是两只眼?

1. One eye only 一只眼
2. Both eyes 两只眼

**PROCEDURE 程序**: If XRType = NEWIW or XRType = REIW and R did not report in last IW that had glaucoma (ZDA037 ≠ 1) 如果是新的受访者或者是上期回答没有得过青光眼的回访受访者（ZDA037 ≠ 1）:

DA037 Has a doctor/nurse/paramedical/doctor of traditional Chinese medicine doctor ever treated you for glaucoma? 是否有医生诊断过您得了青光眼?

1. Yes 是
2. No 否

**PROCEDURE 程序**: If XRType = REIW and R reported in last IW that had glaucoma (ZDA037 = 1) 如果是上期回答得过青光眼的回访受访者（ZDA037 = 1）:

DA037_W2 You told us you had glaucoma, has a doctor/nurse/paramedical/ doctor of traditional Chinese medicine doctor ever treated you for glaucoma relapses since last iw time? [加载基线时间] 上一次的访问记录显示您患有青光眼，之后您的青光眼是否复发?

1. Yes 是

2. No 否
3. Never had glaucoma 一直没有青光眼

PROCEDURE 程序: If R reported in previous IW that wears hearing aid (ZDA038 = 1), skip to DA039 如果是回访受访者，上期访问回答戴助听器，跳至DA039

DA038 Now I have some questions about your hearing. Do you ever wear a hearing aid? 现在有几个关于听力的问题。您有没有戴过助听器？

1. Yes 是
2. No 否

DA039 Is your hearing very good, good, fair, poor, or very poor (with a hearing aid if you normally use it and without if you normally don't)? Would you say your hearing is excellent, very good, good, fair, or poor? 您的听力如何？（如果您经常戴助听器，那么戴助听器时听力如何？如果您不经常戴助听器，那么不戴助听器时听力如何？）是极好，很好，好，一般还是不好？

1. Excellent 极好
2. Very good 很好
3. Good 好
4. Fair 一般
5. Poor 不好

PROCEDURE 程序: If XRType = NEWIW or XRType = REIW that did not lost all teeth in last iw(ZDA040 $\neq$ 1), ask DA040 如果是新的受访者或上期回答牙齿未掉光的回访受访者，询问DA040:

DA040 Have you lost all of your teeth? 您的牙齿是否已经掉光？

1. Yes 是
2. No 否

DA041 Are you often troubled with any body pains? 您经常为身体疼痛而感到苦恼吗？

1. Yes 有
2. No 没有 skip to DA045请跳至DA045

DA042 On what part of your body do you feel pain? Please list all parts of body you are currently feeling pain. 身体哪些部位感到疼痛？请列出所有部位。

1. Head (Headache) 头
2. Shoulder 肩膀
3. Arm 胳膊
4. Wrist 手腕
5. Fingers 手指
6. Chest 胸
7. Stomach (Stomachache) 胃
8. Back 背

9. Waist 腰
10. Buttocks 臀部
11. Leg 腿
12. Knees 膝盖
13. Ankle 脚踝
14. Toes 脚趾
15. Neck 脖子
16. Other, please specify 其它部位，请注明_____(**DA042_1**)

DA042_W2_1 Are you taking measures to reduce the pain? 您是否采取以下措施以减轻疼痛？（可多选）

1. Taking Chinese traditional medicine 服用中药
2. Taking Western modern medicine 服用西药
3. Acupuncture treatment 针灸疗法
4. Professional massage therapy 推拿按摩
5. Other, please specify 其他
6. None 以上都没有

DA045 Are there any other medical diseases or conditions that are important to your health now that we have not talked about? 是否还有其他严重的疾病或身体不适我没问到？

1. Yes 是
2. No 否 → Skip DA046 跳过DA046

DA046 What illness is that? 这些疾病是什么？ _____

DA048 How would you evaluate your health during childhood, up to and including age 15? Excellent, very good, good,fair, poor? 您 15 岁之前（包括 15 岁）的身体状况怎么样？极好，很好，好，一般，不好？

1. Excellent 极好
2. Very Good 很好
3. Good 好
4. Fair 一般
5. Poor 不好

PART II: LIFESTYLE AND HEALTH BEHAVIORS 第二部分：生活方式和健康行为

DA049 During the past month, how many hours of actual sleep did you get at night (average hours for one night)? (This may be shorter than the number of hours you spend in bed.) 过去一个月内，您平均每天晚上真正睡着的时间大约是几小时？（可能短于您在床上躺着的时间）

_____ 0...24 hours 小时

DA050 During the past month, how long did you take a nap after lunch? 过去一个月内，您通常午睡多长时间？_____Minutes 分钟 [IWER：If R didn't take a nap, please record for 0 访员注意：如果受访者不午睡，请记录为 0]

PROCEDURE 程序： DA051 will be presented only to a random subsample of households (half). Main respondent and spouse in the selected households should answer DA051-DA055 仅仅在二次抽样被选中的家庭中询问DA051-DA055题。选中家庭中的主要受访者及其配偶都要回答 DA051 题。

DA051 Now we would like to ask about the amount of time you spend on different types of physical activities in a usual week. 下面有一些问题是有关您通常每周花了多少时间做这些活动？

PHYSICAL ACTIVITIES 身体活动 (KKTYPE)	**DA051**<br>During a usual week, did you do any [...] for at least 10 minutes continuously? 您通常每周有没有至少持续做 [...] 十分钟？	**DA052**<br>During a usual week, on how many days did you do [...] for at least 10 minutes? 您通常每周有多少天做 [...] 至少十分钟？	How much time did you usually spend doing [...] on one of those days? 在做 [...] 的这些天里，您一天花多少时间做 [...]？	
A. Now, think about all the vigorous activities requiring hard/high-intensity physical effort that you do in a usual week. Vigorous activities make you breathe much harder than normal and may include heavy lifting, digging, plowing, aerobics, fast bicycling, and cycling with a heavy load. Think only about those physical activities that you did for at least 10 minutes at a time. 下面请回忆下您通常每周做的非常消耗体力的激烈活动。激烈的活动会让你呼吸急促，比如搬运重物、挖地、耕作、有氧运动、快速骑车、骑车载货等。只需回忆您每次运动了至少十分钟的活动。	2 No 否 ↓<br>1 Yes 是 →	______ 1...7 days 天	**DA053**<br>1. $<$ 2 hours 小时<br>2. $\geq$ 2 hours 小时	**DA054**<br>1. $<$ 30 minutes 分钟<br>2. $\geq$ 30 minutes 分钟<br>**DA055**<br>3. $<$ 4 hours 小时<br>4. $\geq$ 4 hours 小时
B. Now think about activities which take moderate physical effort that you do in a usual week. Moderate physical activities make you breathe somewhat harder than normal and may include carrying light loads, bicycling at a regular pace, or mopping the floor. Again, think about only those physical activities that you did for at least 10 minutes at a time. 下面请回忆下您通常每周做的中等强度的体力活动。中等体力的活动让您的呼吸比平时快一些，比如搬运轻便的东西、常规速度骑自行车、拖地、打太极拳、疾走。只要回忆您每次持续了至少十分钟的活动。	2 No 否 ↓<br>1 Yes 是 →	______ 1...7 days 天	**DA053**<br>1. $<$ 2 hours 小时<br>2. $\geq$ 2 hours 小时	**DA054**<br>1. $<$ 30 minutes 分钟<br>2. $\geq$ 30 minutes 分钟<br>**DA055**<br>3. $<$ 4 hours 小时<br>4. $\geq$ 4 hours 小时
C. Now think about the time you spend walking in a usual week. This includes at work and at home, walking to travel from place to place, and any other walking that you might do solely for recreation, sport, exercise, or leisure. 下面回忆一下您通常每周走路花了多少时间。走路包括工作或者在家的时候从一个地方走到另一个地方，以及其他您为了休闲、运动、锻炼或娱乐而散步。	2 No 否 ↓<br>1 Yes 是 →	______ 1...7 days 天	**DA053**<br>1. $<$ 2 hours 小时<br>2. $\geq$ 2 hours 小时	**DA054**<br>1. $<$ 30 minutes 分钟<br>2. $\geq$ 30 minutes 分钟<br>**DA055**<br>3. $<$ 4 hours 小时<br>4. $\geq$ 4 hours 小时

DA051_1 What's the purpose for doing these physical activities, for entertainment, job demand or exercise in doing these physical activities? 是因为工作需要、娱乐活动、体育锻炼还是其他？

1. Job demands 工作需要
2. Entertainments 娱乐

3. Exercise 体育锻炼
4. Other, please specify 其他

[ACTIVITIES IN LAST MONTH 过去一个月的活动]

DA056 Have you done any of these activities in the last month? (Code all that apply) 您过去一个月是否进行了下列社交活动？(可多选)

1. Interacted with friends 串门、跟朋友交往
2. Played Ma-jong, played chess, played cards, or went to community club 打麻将、下棋、打牌、去社区活动室
3. Provided help to family, friends, or neighbors who do not live with you 向与您不住在一起的亲人、朋友或者邻居提供帮助
4. Went to a sport, social, or other kind of club 跳舞、健身、练气功等
5. Took part in a community-related organization 参加社团组织活动
6. Done voluntary or charity work 志愿者活动或者慈善活动
7. Cared for a sick or disabled adult who does not live with you 照顾与您不住在一起的病人或残疾人
8. Attended an educational or training course 上学或者参加培训课程
9. Stock investment 炒股（基金及其他金融证券）
10. Used the Internet 上网
11. Other 其他社交活动
12. None of these 以上均没有

[CHECK: You cannot select "None of these" together with any other answer. Please change your answer 检查点：您不能同时选择"以上均没有"和其他选项。请重新选择]

**PROCEDURE 程序**：If DA056= 10, ask DA056_W3 如果DA056= 10，询问DA056。

DA056_W3 请问您使用以下哪些工具上网?

1. Desktop computer 台式电脑
2. Laptop computer 笔记本电脑
3. Tablet computer 平板电脑（如 IPAD）
4. Cellphone 手机
5. Other devices 其他设备，请注明_____ （DA056_W3_1）

**PROCEDURE 程序**：循环询问过去一个月参加的社交活动的频率

DA057 Frequency of activity in the last month 过去一个月的活动频率

How often in the last month [did/have][you] [do voluntary or charity work/cared for a sick or disabled adult/provided help to family, friends or neighbors/attended an educational or training course/ Interacted with friends /go to a sport,social or other kind of club/taken part in a community-related organization]? Almost daily, almost every week, or not regularly? 过去一个月，您每隔多长时间会做刚才说的这些活动（志愿者活动或者慈善活动、照顾病人或残疾人、给家里人、朋友或者邻居提供帮助、接受教育或者参加培训课程、与朋友来往、去运动娱乐、社交或者其他类型的俱乐部、参加社团组织活动、炒股、上网）？差不多每天，差不多每周或不经常？

1. Almost daily 差不多每天
2. Almost every week 差不多每周
3. Not regularly 不经常

[INTRO: Next, I would like to ask whether you have had the habit of smoking cigarettes/smoking a pipe/chewing tobacco, now or in the past. By smoking we mean smoking more than 100 cigarettes in your life 下面我想知道您现在或者过去是否有抽烟、用烟管吸烟或咀嚼烟草的习惯（吸烟指的是一生吸烟 100 支以上）]

BRANCHPOINT:
If XRType = REIW and R reported ever smoked (ZDA059 = 1), skip to DA061 如果回访受访者吸过烟，跳至DA061
If XRType = REIW and R did not report ever smoked (ZDA059≠ 1); or XRType = NEWIW, ask DA059 如果是从未吸过烟的回访受访者或新的受访者，询问DA059

DA059 Have you ever chewed tobacco, smoked a pipe, smoked self-rolled cigarettes, or smoked cigarettes/cigars? 您吸过烟吗？（包括香烟、旱烟、用烟管吸烟或咀嚼烟草）
1. Yes 是
2. No 否 Skip to DA067 请跳至DA067

DA061_W3 If XRType = REIW, R reported ever smoked (ZDA059= 1) and he/she did not answer DA061 in last interview, ask: 如果是吸过烟的回访受访者且上次访问时没有回答DA061，询问：In last interview，did you have the habit or have you totally quit? 上一次访问时，您在吸烟还是戒烟了？
1. Still have 仍然抽烟
2. Quit 戒烟
3. Never smoked 从未吸过烟

DA061 **PROCEDURE 程序**: If XRType = REIW 如果是回访受访者
Our records from your last interview show that you have ever smoked, 上一次的访问记录显示您曾经吸烟，
Do you still have the habit or have you totally quit? 您现在还在吸烟还是戒烟了？
PROCEDURE 程序: If XRType = NEWIW 如果是新受访者
Do you still have the habit or have you totally quit? 您现在还在吸烟还是戒烟了？
1. Still have 仍然抽烟 Skip DA062 请跳过DA062
2. Quit 戒烟
3. Never smoked 从未吸过烟 Skip to DA067 请跳至DA067

DA060 Which products did/do you normally use? 吸烟时，一般抽什么烟？
1. Smoking a pipe 用烟管吸烟（烟袋、旱烟）
2. Smoking self-rolled cigarettes 自己卷烟抽
3. Filtered cigarette 带滤咀香烟
4. Unfiltered cigarette 不带滤咀香烟

5. Cigar 雪茄
6. Water cigarettes 水烟

DA062 At what age did you totally quit smoking? 您这次成功戒烟是在多少岁或那一年? Age 年龄_____1...120 (**DA062_1**) Years 岁 or 或_____1900...2015 (**DA062_2**) Year 年 [IWER: Mark the year using four digits 访员注意: 用 4 位数表示年]

PROCEDURE 程序: If DA060= 3 or 4, ask DA063 and DA064 如果DA060= 3 or 4, 询问DA063和DA064。

DA063 In one day about how many cigarettes do/did you consume [preload:now/before totally quitting]? 您 [系统加载: 现在/戒烟前] 平均一天抽多少支香烟? _____Cigarettes 支

DA064 How much does/did it cost per pack = 20 cigarettes? 您 [系统加载: 现在/戒烟前], 抽的香烟多少钱一包 (一包 20 支) ? _____Yuan 元 [IWER: Prompt R: we are asking price at that time, not current price 访员注意: 提示受访者, 此处询问的是当时价格, 非现在的价格]

PROCEDURE 程序: If XRType = REIW that has been asked when started smoking, skip DA065 如果回访受访者之前被问到过开始吸烟的年龄, 跳过DA065。

DA065 At what age did you start to smoke on a regular basis? 您是多少岁开始经常抽烟的? Age 年龄_____ 1...120 (**DA065_1**) years 岁 Or 或_____ 1900...2015 (**DA065_2**) Year 年 [IWER: Mark the year using four digits 访员注意: 用 4 位数表示年]

DA067 Did you drink any alcoholic beverages, such as beer, wine, or liquor in the past year? How often? 在过去的一年, 您喝酒吗, 包括啤酒、葡萄酒或白酒等? 喝酒频率如何?

1. Drink more than once a month. 喝酒, 每月超过一次
2. Drink but less than once a month 喝酒, 但每月少于一次 Skip to DA069 请跳至DA069
3. None of these 什么都不喝 Skip to DA069 请跳至DA069

DA068 What type of alcoholic beverages did you drink? Liquor, wine, or beer? (code all that apply) 您平时喝哪种酒? 烈性酒、葡萄酒、啤酒还是其他? (可多选)

1. Liquor, including white liquor, whisky, and others 烈性酒, 包括白酒、威士忌和其他酒 Skip to DA071 请跳至DA071
2. Beer 啤酒 Skip to DA071 请跳至DA071
3. Wine or rice wine 葡萄酒、米酒或黄酒 Skip to DA071 请跳至DA071

PROCEDURE 程序: If XRType = REIW that has been asked whether drinking alcoholic beverages in the past, skip DA069 如果回访受访者之前被问过以前是否喝酒, 跳过DA069。

DA069 Did you ever drink alcoholic beverages in the past? How often? 您以前喝酒吗? 喝酒频率如何?

1. I never had a drink. 我从不或极少喝酒 → Skip to procedure before DA079 请跳至DA079 前面的程序控制
2. I used to drink less than once a month. 我很少喝酒，每月少于一次 → Skip to procedure before DA079 请跳至DA079 前面的程序控制
3. I used to drink more than once a month. 我以前喝酒，每月超过一次

**PROCEDURE 程序**：If XRType = REIW that has been asked when quiting alcoholic beverages in the past, skip DA070 如果回访受访者之前被问过何时戒酒的，跳过DA070。

DA070 When did you quit or reduce drinking? 请问您什么时候戒酒或减少喝酒次数的？
_____1900...2015 (**DA070_1**)Year 年 or Age 或年龄: _____1...120 (**DA070_2**) Years 岁
[IWER: Record year in 4 digits 访员注意：年请用 4 位数表示]

**PROCEDURE 程序**：If XRType = REIW that has been asked when started drinking, skip DA071 如果回访受访者之前被问到过开始饮酒的年龄，跳过DA071 题。

DA071 When did you start drinking? 请问您什么时候开始饮酒？
_____1900...2015 (**DA071_1**)Year 年 or Age 或年龄: _____1...120 (**DA071_2**) Years 岁
[IWER: Record year in 4 digits. 访员注意：年请用 4 位数表示]

[INTRO: Now, I am going to ask you how often and how much you drank during the past year. Please tell me how often you drank per month, and how much you drank at a time on average. I will repeat the questions for different types of alcoholic beverages 现在我想了解过去一年您喝酒的情况。请回答您平均每个月喝多少次酒，一次大概喝多少。我会针对不同的酒类，分别提问]

**CAPI 程序**：If DA068= 1, ask DA072 如果DA068= 1，询问DA072。

DA072 How often did you drink liquor, including white liquor, whisky, and others per month in the last year? 过去一年内您平均一个月喝几次烈性酒，包括白酒、威士忌？
1. Once a month 每月一次
2. 2-3 times a month 每月 2-3 次
3. Once a week 每周一次
4. 2-3 times a week 每周 2-3 次
5. 4-6 times a week 每周 4-6 次
6. Once a day 每天一次
7. Twice a day 一天两次
8. More than twice a day 一天超过两次

DA073 The last time you drank liquor last year, how many liang of liquor did you drink? (1 liang = $50cc/50ml$) 请问您过去一年内最近一次喝烈性酒，喝了多少两？（1 两 = 50 毫升）
_____ Liang 两

**CAPI 程序**：If DA068 = 2, ask DA074 如果DA068 = 2，询问DA074。

DA074 How many times per month did you drink beer in the last year? 过去一年内您平均一个月喝几次啤酒?

1. Once a month 每月一次
2. 2-3 times a month 每月 2-3 次
3. Once a week 每周一次
4. 2-3 times a week 每周 2-3 次
5. 4-6 times a week 每周 4-6 次
6. Once a day 每天一次
7. Twice a day 一天两次
8. More than twice a day 一天超过两次

DA075 The last time you drank beer last year, how many bottles of beer did you drink? (1bottle= 2.5 mugs, 1mug= 220*cc*) 请问您过去一年内最近一次喝啤酒，喝了多少瓶?（1 瓶 =2.5 杯，1 杯 =220 毫升）_____ **(DA075_1**)0...120 Bottles 瓶 or 或者 _____**(DA075_2**)0...300 Mugs 杯

**CAPI 程序**：If DA068 = 3, ask DA076 如果DA068 = 3，询问DA076。

DA076 How often did you drink wine or rice wine per month in the last year? 过去一年内您平均一个月喝几次葡萄酒、米酒或黄酒?

1. Once a month 每月一次
2. 2-3 times a month 每月 2-3 次
3. Once a week 每周一次
4. 2-3 times a week 每周 2-3 次
5. 4-6 times a week 每周 4-6 次
6. Once a day 每天一次
7. Twice a day 一天两次
8. More than twice a day 一天超过两次

DA077 The last time you drank it last year, how many liang of wine did you drink? (1 liang=50cc) 请问您过去一年内最近一次喝葡萄酒、米酒或黄酒，喝了多少两?（1 两 =50 毫升）_____0.00...100.00 Liang 两

[IWER: Please do not ask proxy the following question DA079 and DA080 访员注意：DA079 and DA080 题不允许请代理人回答]

DA079 How would you rate your health status? Would you say your health is very good, good, fair, poor or very poor? 您觉得您的健康状况是很好，好，一般，不好，还是很不好?

1. Very good 很好
2. Good 好
3. Fair 一般
4. Poor 不好

5. Very poor 很不好

DA080 Next I have some questions about your health. Would you say your health is excellent, very good, good, fair, or poor? 您觉得您自己的健康状况怎么样？是极好，很好，好，一般还是不好？[IWER：interviewer should read all the following options 访员注意：请读出所有的选项]

1. Excellent 极好
2. Very good 很好
3. Good 好
4. Fair 一般
5. Poor 不好

**INTERVIEWER CHECK AGE OF RESPONDENT? 访员请检查受访者的年龄。**	**1.** < 65 **YEAR 岁** → **COLUMN A A 栏**<br>**2.** 65 – 69 **YEAR 岁** → **COLUMN B B 栏**<br>**3.** 70 – 74 **YEAR 岁** → **COLUMN C C 栏**<br>**4.** 75 – 79 **YEAR 岁** → **COLUMN D D 栏**<br>**5.** 80 – 84 **YEAR 岁** → **COLUMN E E 栏**				**6.** 85 – 89 **YEAR 岁** → **COLUMN F F 栏**<br>**7.** 90 – 94 **YEAR 岁** → **COLUMN G G 栏**<br>**8.** 95 – 99 **YEAR 岁** → **COLUMN H H 栏**<br>**9.** ≥ 100 **YEAR 岁** → **COLUMN I I 栏**			
	A	B	C	D	E	F	G	H	I
AGE 年龄	75 years 岁	80 years 岁	85 years 岁	90 years 岁	95 years 岁	100 years 岁	105 years 岁	110 years 岁	115 years 岁
**DA081** Suppose there are 5 steps, where the lowest step represents the smallest chance and the highest step represents the highest chance, on what step do you think is your chance in reaching the age of [...]? 假定有五个级别，最低一级代表可能性最小，最高一级代表可能性最大，您设想您活到这个年龄的可能性有多大。1 Almost impossible 几乎不可能 2 Not very likely 不太可能 3 Maybe 有可能 4 Very likely 很可能 5 Almost certain 简直一定	1<br>2<br>3<br>4<br>5	1<br>2<br>3<br>4<br>5	1<br>2<br>3<br>4<br>5	1<br>2<br>3<br>4<br>5	1<br>2<br>3<br>4<br>5	1<br>2<br>3<br>4<br>5	1<br>2<br>3<br>4<br>5	1<br>2<br>3<br>4<br>5	1<br>2<br>3<br>4<br>5

DB FUNCTIONAL LIMITATIONS AND HELPERS 身体功能障碍以及辅助者

CAPI 程序：If R is younger than 50 (year of birth is after 1965) and if DA001 = 1 or 2 or DA002 = 1 or 2 and DA005 = 2 and DA007 = 2 and DA008 = 2, skip DB001- DB015 如果受访者年龄小于 50 岁（出生年份在 1965 年之后），并且自我评价健康状况良好、没有残疾和慢性病，跳过 DB001-DB015。

[We need to understand difficulties people may have with various activities because of a health or physical problem. Please tell me whether you have difficulty performing any of the following tasks on a regular basis. Exclude any difficulties that you expect to last less than three months 人们可能会由于健康或身体的原因而在多种活动中有困难。请说说您每天做事的时候是否遇到了这些困难（排除那些你预计在三个月内能解决的问题）。每道题目的选项都是一样的，包括没有困难，有困难但仍可以完成，有困难且需要帮助，无法完成。请选择合适的答案]

DB001 Do you have any difficulty with running or jogging about 1 Km? 您现在跑或慢跑 1 公里，有困难吗？

1. No, I don't have any difficulty 没有困难 → Skip to DB004 跳至DB004
2. I have difficulty but can still do it 有困难但仍可以完成
3. Yes, I have difficulty and need help 有困难，需要帮助
4. I can not do it 无法完成

DB002 Do you have difficulty with walking 1 km? 您现在走 1 公里，有困难吗？

1. No, I don't have any difficulty 没有困难 → Skip DB003 跳过DB003
2. I have difficulty but can still do it 有困难但仍可以完成
3. Yes, I have difficulty and need help 有困难，需要帮助
4. I can not do it 无法完成

DB003 Do you have difficulty with walking 100 metres? 您走 100 米，有困难吗？

1. No, I don't have any difficulty 没有困难
2. I have difficulty but can still do it 有困难但仍可以完成
3. Yes, I have difficulty and need help 有困难，需要帮助
4. I can not do it 无法完成

DB004 Do you have difficulty with getting up from a chair after sitting for a long period? 您在椅子上坐时间久了再站起来，有困难吗？

1. No, I don't have any difficulty 没有困难
2. I have difficulty but can still do it 有困难但仍可以完成
3. Yes, I have difficulty and need help 有困难，需要帮助
4. I can not do it 无法完成

DB005 Do you have difficulty with climbing several flights of stairs without resting? 您连续不停地爬几层楼，有困难吗？

1. No, I don't have any difficulty 没有困难
2. I have difficulty but can still do it 有困难但仍可以完成
3. Yes, I have difficulty and need help 有困难，需要帮助
4. I can not do it 无法完成

DB006 Do you have difficulty with stooping, kneeling, or crouching? 弯腰、屈膝或者下蹲，您有困难吗？

1. No, I don't have any difficulty 没有困难
2. I have difficulty but can still do it 有困难但仍可以完成
3. Yes, I have difficulty and need help 有困难，需要帮助
4. I can not do it 无法完成

DB007 Do you have difficulty with reaching or extending your arms above shoulder level? (he/she is regarded as not having difficulty only if he/she can extend both of his/her arms, otherwise he/she is regarded as having difficulty.) 您把手臂沿着肩向上伸展，有困难吗？（两个手都没困难才算没困难，否则算有困难）

1. No, I don't have any difficulty 没有困难
2. I have difficulty but can still do it 有困难但仍可以完成
3. Yes, I have difficulty and need help 有困难，需要帮助
4. I can not do it 无法完成

DB008 Do you have difficulty with lifting or carrying weights over 10 jin, like a heavy bag of groceries? 您提 10 斤重的东西，有困难吗？（注意是市斤）
1. No, I don't have any difficulty 没有困难
2. I have difficulty but can still do it 有困难但仍可以完成
3. Yes, I have difficulty and need help 有困难，需要帮助
4. I can not do it 无法完成

DB009 Do you have difficulty with picking up a small coin from a table? 您从桌上拿起一小枚硬币，有困难吗？
1. No, I don't have any difficulty 没有困难
2. I have difficulty but can still do it 有困难但仍可以完成
3. Yes, I have difficulty and need help 有困难，需要帮助
4. I can not do it 无法完成

**CAPI 程序**：If (DB001 = 1 & DB003 = 1 ... DB009 = 1), then skip to DB016 如果受访者在DB001～ DB009 没有任何困难，请跳至DB016

[Here are a few more everyday acivities. Please tell me if you have any difficulties with these because of a physical, mental, emotional or memory problem. Again, exclude any that you expect to last less than three months 下面我们想了解一下您日常生活的情况。请问您目前是否因为身体、精神、情感或者记忆方面的原因导致完成下面我们提到的一些日常行为有困难。我们指的“困难”不包括那些预计三个月内能够解决的困难]

DB010 Because of health and memory problems, do you have any difficulty with dressing? Dressing includes taking clothes out from a closet, putting them on, buttoning up, and fastening a belt. 请问您是否因为健康和记忆的原因，自己穿衣服有困难？穿衣服包括从衣橱中拿出衣服，穿上衣服，扣上钮扣，系上腰带。
1. No, I don't have any difficulty 没有困难 → Skip to DB011 跳至DB011
2. I have difficulty but can still do it 有困难但仍可以完成
3. Yes, I have difficulty and need help 有困难，需要帮助
4. I can not do it 无法完成

DB010_W2 Does anyone ever help you dress? 穿衣服的时候是否有人帮助你？
1. Yes 有
2. No 没有

DB011 Because of health and memory problems, do you have any difficulty with bathing or showering? 请问您是否因为健康和记忆的原因，洗澡有困难？

1. No, I don't have any difficulty 没有困难 → Skip to DB012 跳至DB012
2. I have difficulty but can still do it 有困难但仍可以完成
3. Yes, I have difficulty and need help 有困难，需要帮助
4. I can not do it 无法完成

DB011_W2 Does anyone ever help you bathe? 洗澡的时候是否有人帮助你？

1. Yes 有
2. No 没有

DB012 Because of health and memory problems, do you have any difficulty with eating, such as cutting up your food? (Definition: By eating, we mean eating food by oneself when it is ready) 请问您是否因为健康和记忆的原因，自己吃饭有困难，比如自己夹菜？（定义：当饭菜准备好以后，自己吃饭定义为用餐。）

1. No, I don't have any difficulty 没有困难 → Skip to DB013 跳至DB013
2. I have difficulty but can still do it 有困难但仍可以完成
3. Yes, I have difficulty and need help 有困难，需要帮助
4. I can not do it 无法完成

DB012_W2 Does anyone ever help you eat? 吃饭的时候是否有人帮助你？

1. Yes 有
2. No 没有

DB013 Do you have any difficulty with getting into or out of bed? 您起床、下床有没有困难？

1. No, I don't have any difficulty 没有困难 → Skip to DB014 跳至DB014
2. I have difficulty but can still do it 有困难但仍可以完成
3. Yes, I have difficulty and need help 有困难，需要帮助
4. I can not do it 无法完成

DB013_W2 Does anyone ever help you get in or out of bed? 起床、下床是否有人帮你？

1. Yes 有
2. No 没有

DB014 Because of health and memory problems, do you have any difficulties with using the toilet, including getting up and down? 请问您是否因为健康和记忆的原因，上厕所有困难，包括蹲下、站起？

1. No, I don't have any difficulty 没有困难 → Skip to DB015 跳至DB015
2. I have difficulty but can still do it 有困难但仍可以完成
3. Yes, I have difficulty and need help 有困难，需要帮助
4. I can not do it 无法完成

DB014_W2 Does anyone ever help you use the toilet? 上厕所是否有人帮你？

1. Yes 有
2. No 没有

DB015 Because of health and memory problems, do you have any difficulties with controlling urination and defecation? If you use a catheter (conduit) or a pouch by yourself, then you are not considered to have difficulties. 请问您是否因为健康和记忆的原因，控制大小便有困难？（自己能够使用导尿管或者尿袋算能够控制自理）

1. No, I don’t have any difficulty 没有困难
2. I have difficulty but can still do it 有困难但仍可以完成
3. Yes, I have difficulty and need help 有困难，需要帮助
4. I can not do it 无法完成

DB016 Because of health and memory problems, do you have any difficulties with doing household chores? (Definition: By doing household chores, we mean house cleaning, doing dishes, making the bed, and arranging the house) 请问您是否因为健康和记忆的原因，做家务活的时候有困难？（定义：做家务，我们指的是房屋清洁，洗碗盘，整理被褥和房间摆设） [IWER: If R cannot mop the floor, but can scrub, or R cannot fold heavy bedding, but is able to do light ones, then mark (3) 访员注意：如果受访者不能拖地，但是可以擦洗桌子，或者受访者不能整理重的被褥，但是可以整理一些轻便的，请选择（3）]

1. No, I don’t have any difficulty 没有困难 → skip to DB017 跳至DB017
2. I have difficulty but can still do it 有困难但仍可以完成
3. Yes, I have difficulty and need help 有困难，需要帮助
4. I can not do it 无法完成

DB016_W2 Does anyone help you do household chores? 做家务的时候是否有人帮助你？

1. Yes 有
2. No 没有

DB017 Because of health and memory problems, do you have any difficulties with preparing hot meals? (Definition: By preparing hot meals, we mean preparing ingredients, cooking, and serving food) 请问您是否因为健康和记忆的原因，做饭有困难？（定义：做饭我们定义为准备原材料，做饭菜，端上餐桌） [IWER: If another person prepares ingredients or if R can cook rice, but is not able to prepare side dishes, then mark (3) 访员注意：如果由于健康原因，受访者需要别人帮忙洗菜切菜，或者受访者只能自己煮米饭但不能做菜，也就是说，由于健康原因受访者只能完成做饭的一些简单的动作，那么选择（3）]

1. No, I don’t have any difficulty 没有困难 → skip to DB018 跳至DB018
2. I have difficulty but can still do it 有困难但仍可以完成
3. Yes, I have difficulty and need help 有困难，需要帮助
4. I can not do it 无法完成

DB017_W2 Does anyone help you prepare hot meals? 做饭的时候是否有人帮助你？

1. Yes 有
2. No 没有

DB018 Because of health and memory problems, do you have any difficulties with shopping for groceries? By shopping, we mean deciding what to buy and paying for it. 请问您是

否因为健康和记忆的原因，自己去商店买食品杂货有困难？我们这里说的买东西是指决定买什么和付钱。

1. No, I don't have any difficulty 没有困难 → skip to DB035 跳至DB035
2. I have difficulty but can still do it 有困难但仍可以完成
3. Yes, I have difficulty and need help 有困难，需要帮助
4. I can not do it 无法完成

DB018_W2 Does anyone help you shop for groceries? 是否有人帮助你去商店买食品杂货等？

1. Yes 有
2. No 没有

DB035 Because of health and memory problems, do you have any difficulties with making phone calls? 请问您是否因为健康和记忆的原因，拨打电话有困难？

1. No, I don't have any difficulty 没有困难 → skip to DB020 跳至DB020
2. I have difficulty but can still do it 有困难但仍可以完成
3. Yes, I have difficulty and need help 有困难，需要帮助
4. I can not do it 无法完成
5. Not relevant to me (no phone) 不适用，家里没有电话 → skip to DB020 跳至DB020

DB035_W2 Does anyone help you make telephone calls? 打电话的时候是否有人帮助你？

1. Yes 有
2. No 没有

DB020 Because of health and memory problems, do you have any difficulties with taking medications? By taking medications, we mean taking the right portion of medication right on time. 请问您是否因为健康和记忆的原因，自己吃药有困难？吃药是指能记得什么时间吃和吃多少。

1. No, I don't have any difficulty 没有困难 → skip to DB019 跳至DB019
2. I have difficulty but can still do it 有困难但仍可以完成
3. Yes, I have difficulty and need help 有困难，需要帮助
4. I can not do it 无法完成

DB020_W2 Does anyone help you take medications? 吃药的时候是否有人帮助你？

1. Yes 有
2. No 没有

DB019 Because of health and memory problems, do you have any difficulties with managing your money, such as paying your bills, keeping track of expenses, or managing assets? 请问您是否因为健康和记忆的原因，管钱有困难，比如支付账单、记录支出项目、管理财物？

1. No, I don't have any difficulty 没有困难 → skip DB019_W2 跳过DB019_W2
2. I have difficulty but can still do it 有困难但仍可以完成
3. Yes, I have difficulty and need help 有困难，需要帮助
4. I can not do it 无法完成

DB019_W2 Does anyone help you manage your money? 是否有人帮助你管钱?

1. Yes 有
2. No 没有

PROCEDURE 程序: If DB010_W2= 1 or DB011_W2= 1 or DB012_W2= 1 or DB013_W2= 1 or DB014_W2= 1 or DB016_W2= 1 or DB017_W2= 1 or DB018_W2= 1 or DB035_W2= 1 or DB020_W2= 1 or DB019_W2= 1, ask DB022_W3_1; otherwise, skip to DB029

DB022_W3_1 Who most often helps you with [make sure we ask this only once for all these activities; do not ask for each problem separately] (dressing, bathing, eating, getting out of bed, using the toilet, controlling urination and defecation, doing chores, preparing hot meals, shopping, managing money, making phone calls, taking medications) (May choose up to 3 persons)? 请问在以上困难中，谁帮助您最多？(在穿衣、洗澡、吃饭、起床、入厕、家务、做饭、购物、打电话、吃药、管钱等困难中）(多选题)

1. Spouse 配偶
2. Father, Mother, Father-in-law, Mother-in-law 父母、岳父母、公公、婆婆
3. Children, Children's spouses, Grandson, Granddaughter 子女、儿媳/女婿、孙子女/外孙子女
4. Sibling, Brother-in-law, Sister-in-law, Sibling of spouse, Children of sibling, Sibling of spouse, Brother-in-law, Sister-in-law of spouse, Children of brother-in-law, Children of sister-in-law 兄弟姐妹及其配偶、子女，您配偶的兄弟姐妹及其配偶、子女
5. Other relative 其他亲属
6. Paid helper (such as nanny) 雇佣人员（如保姆），共_____位（**DB022_W3_1_1**）
7. Volunteer or Employee of facility 志愿者或者志愿机构人员
8. Nursing home 养老院人员
9. 社区提供的帮助
10. Other 其他人员

[Note: "employee(s) of facility" appears on list only for an R currently living in a nursing home or who was living in a nursing home or hospice when he/she died 注意：养老院人员选项仅针对住在养老院或在养老院去世的受访者]

PROCEDURE 程序: If DB022_W3_1= 2, ask DB023_W3_1

DB023_W3_1 Father, mother, father-in-law, monther-in-law, Who help you most? 在父母、岳父母、公公、婆婆中，帮助您的是哪几位？(多选题)

1. Father 父亲
2. Mother 母亲
3. Father-in-law 岳父/公公
4. Mother-in-law 岳母/婆婆

PROCEDURE 程序: If DB022_W3_1= 3, ask DB023_W3_2

DB023_W3_2 For the children, children-in-law, grandson, granddaughter who helped you, which children's family are they from? 帮助您的子女、儿媳/女婿、孙子女/外孙子女，是以下哪个子女家的？(多选题)

1-25 [Preload children's name] [加载子女姓名]
26 None of the above 以上都没有

[For each helpers from every children's family, repeat the question 对来自每个子女家的帮助者，循环询问]

DB023_W3_3 [加载子女姓名] 家的哪些人亲自帮助您？
1 [Preload child's name] himself/herself [加载子女姓名] 本人
2 [Preload child's name] his/her spouse [加载子女姓名] 的配偶
3 [Preload child's name] his/her children [加载子女姓名] 的孩子，即您的（外）孙子女，亲自帮助您的 [加载子女姓名] 的孩子有_____个 (**DB023_W3_3_1**)

**PROCEDURE 程序**：If DB022_W3_1= 4, ask DB023_W3_4

DB023_W3_4 For the siblings, spouse and children of siblings, siblings of your spouse, spouse and children of siblings of your spouse who helped you, which siblings' family are they from? 帮助您的兄弟姐妹及其配偶、子女，您配偶的兄弟姐妹及其配偶、子女，是以下哪个兄弟姐妹家的？（多选题）
1-15 [Preload siblings' name] [加载兄弟姐妹的姓名]
16-30 [Preload spouse's siblings' name] [加载配偶的兄弟姐妹的姓名]
99 None of the above 以上都没有

[For each helpers from every siblings, siblings of spouse, repeat the question 对来自每个兄弟姐妹、配偶兄弟姐妹家的帮助者，循环询问]

DB023_W3_5 For the family members of [Preload name of siblings, siblings of spouse], who help you in person? [加载兄弟姐妹姓名/配偶兄弟姐妹的姓名] 家的哪些人亲自帮助您？
1 [Preload siblings' (of spouse) name] himself/herself [加载（配偶的）兄弟姐妹姓名] 本人
2 [Preload siblings' (of spouse) name] his/her spouse [加载（配偶的）兄弟姐妹姓名] 的配偶
3 For the children from [Preload siblings' (of spouse) name], how many children from [Preload siblings' (of spouse) name] help you in person? [加载（配偶的）兄弟姐妹姓名] 的孩子，亲自帮助您的 [加载（配偶的）兄弟姐妹姓名] 的孩子有_____ 个 (**DB023_W3_5_1**)

**PROCEDURE 程序**：If DB022_W3_1= 5, ask DB023_W3_6

DB023_W3_6 The number of the relatives who help you in person 亲自为您提供帮助的其他亲属共有_____位，What's their relationship with you 都是您什么人？_____(**DB023_W3_6_1**)

**PROCEDURE 程序**：If DB022_W3_1= 10, ask DB023_W3_7

DB023_W3_7 The number of the others who help you in person 亲自为您提供帮助的其他人共有_____位，What's that person's relationship with you 都是您什么人？_____(**DB023_W3_7_1**)

Note: 如果帮助者人数大于 7 个，询问 DB023_W3_9

DB023_W3_9 From all he helpers list below, please select the most important 7 helpers for you 在下面所列的所有帮助者中，请选择帮助您最多的 7 个人。

[Name of all helpers] [列出帮助者的姓名]

PROCEDURE 程序：For each helper chosen in DB022_W3_1-DB023_W3_7, ask DB023-DB026 对 DB022_W3_1-DB023_W3_7 选择的每一位提供帮助的人，提问 DB023-DB026。

DB023 During the last month, on about how many days did [helper's name chosen from DB022_W3_1-DB023_W3_7] help you? 在过去一个月内，[DB022_W3_1-DB023_W3_7中选择的帮助者] 帮助了您多少天？ _____1...31 Days 天

DB024 On the days [helper's name chosen from DB022_W3_1-DB023_W3_7] helps you, about how many hours per day is that? 在 [DB022_W3_1-DB023_W3_7中选择的帮助者] 帮助您的那些天，他/她大概每天花多少小时帮助您？ _____ 1...24 Hours 小时 [IWER: less than an hour, mark 1 访员注意：少于一个小时请记为 1]

DB025 Is he/she living in your home? 他/她在照顾您的时候，是否和您住在一起？

1. Yes 是
2. No 否

DB026 Is [helper's name chosen from DB022_W3_1-DB023_W3_7] paid to help you? 请问 [DB022_W3_1-DB023_W3_7中选择的帮助者] 帮助您，您要付钱吗？

1. Yes 是
2. No 否

PROCEDURE 程序：If R paid for help in DB026, skip to DB027 如果在DB026受访者为获得帮助而支付费用，请跳转至DB027。If R did not pay for help, skip to DB029 如果受访者不需要帮助付费，那么跳转至DB029。

DB027 About how much in total did you pay (including value of the goods you gave them as repayment for their help) for the help during the past month? 在过去一个月内您给这些帮助您的人大约总共付了多少钱（包括作为报答给他们财物的总价值）？ _____ Yuan 元

DB028 Who contributed most to paying this cost? Please choose one person who paid the most. 这些钱谁出的最多，请选择一个支付的最多的人。

1. Spouse 配偶
2. Father, Mother, Father-in-law, Mother-in-law 父母、岳父母、公公、婆婆
3. Children, Children's spouses, Grandson, Granddaughter 子女、儿媳/女婿、孙子女/外孙子女
4. Sibling, Brother-in-law, Sister-in-law, Sibling of spouse, Children of sibling, Sibling of spouse, Brother-in-law, Sister-in-law of spouse, Children of brother-in-law, Children of sister-in-law 兄弟姐妹及其配偶、子女，您配偶的兄弟姐妹及其配偶、子女

5. Other relative 其他亲属
6. Paid helper(such as nanny) 雇佣人员（如保姆）
7. Volunteer or Employee of facility 志愿者或者志愿机构人员
8. Nursing home 养老院人员
9. Community 社区提供的帮助
10. Other 其他人员

**PROCEDURE 程序**：If DB028= 2, ask DB028_W3_1

DB028_W3_1 Parents, parents-in-law, which of them paid the most? 在父母、岳父母、公公、婆婆中，支付最多的是哪一个？

1. Father 父亲
2. Mother 母亲
3. Father-in-law 岳父/公公
4. Mother-in-law 岳母/婆婆

**PROCEDURE 程序**：If DB028= 3, ask DB028_W3_2

DB028_W3_2 For the families of your children, children-in-law, grandchildren, which family paid the most? 在您的子女、儿媳/女婿、孙子女/外孙子女家中，支付最多的是哪一家？

1-25 [Preload children's name] [加载子女姓名]
26 None of the above 以上都没有

[For Payers from children's family, ask the question 对来自子女家的支付者，询问]

DB028_W3_3 For the family members of [Preload children's name], who paid you most? [加载子女姓名] 家的哪个人为您支付最多？

1 [Preload name of children] himself/herself [加载子女姓名] 本人
2 [Preload name of children] his/her spouse [加载子女姓名] 的配偶
3 [Preload name of children] his/her children, your grandchildren [加载子女姓名] 的孩子，即您的（外）孙子女

**PROCEDURE 程序**：If DB028= 4, ask DB028_W3_4

DB028_W3_4 For your siblings, spouse and children of your siblings, your spouse's siblings, spouse and children of your spouse's siblings, who paid you most? 在您的兄弟姐妹及其配偶、子女，您配偶的兄弟姐妹及其配偶、子女中，支付最多的是哪一家？

1-15 [Preload name of siblings] [加载兄弟姐妹的姓名]
16-30 [Preload name of siblings of spouse] [加载配偶的兄弟姐妹的姓名]
99 None of the above 以上都没有

[For payers from every siblings, siblings of spouse, repeat the question 对来自每个兄弟姐妹、配偶兄弟姐妹家的支付者，循环询问]

DB028_W3_5 For the family members of [Preload name of siblings, siblings of spouse], who paid you most? [加载兄弟姐妹姓名/配偶兄弟姐妹的姓名] 家的哪个人为您支付的最多?

1 [Preload name of siblings (of spouse)] himself/herself [加载（配偶的）兄弟姐妹姓名] 本人
2 [Preload name of siblings (of spouse)] him/her spouse [加载（配偶的）兄弟姐妹姓名] 的配偶
3 For the children of [Preload name of siblings (of spouse)], how many chilren of [Preload name of siblings (of spouse)] help you in person [加载(配偶的)兄弟姐妹姓名] 的孩子, 亲自帮助您的 [加载(配偶的)兄弟姐妹姓名] 的孩子有_____个 (**DB028_W3_5_1**)

DB029 Do you use the following auxiliary? (Code all that apply) 你使用以下辅助么?（可多选）

1. Walking stick 拐杖
2. Travel device 代步器
3. Manual wheelchair 手动轮椅
4. Electric Wheelchair 电动轮椅
5. Catheter, urine collection bag 导尿管，导尿袋
6. Toilet Series 便携式座便器
7. None of the above 以上都没有

DB030 Suppose that in the future, you needed help with basic daily activities like eating or dressing. Do you have relatives or friends (besides your spouse/partner) who would be willing and able to help you over a long period of time? 如果以后您在日常生活方面需要照顾，比如吃饭，穿衣，有亲人（除了配偶以外）或朋友能长期照顾您吗?

1. Yes 是
2. No 否 → Skip to DB035_W3_1 请跳至 DB035_W3_1

DB031 What is the relationship to you of that person or those persons? （Choose all that apply）他/她是您的什么人?（可多选）

1. Spouse 配偶
2. Father, Mother, Father-in-law, Mother-in-law 父母、岳父母、公公、婆婆
3. Children, Children’s spouses, Grandson, Granddaughter 子女、儿媳/女婿、孙子女/外孙子女
4. Sibling, Brother-in-law, Sister-in-law, Sibling of spouse, Children of sibling, Sibling of spouse, Brother-in-law, Sister-in-law of spouse, Children of brother-in-law, Children of sister-in-law 兄弟姐妹及其配偶、子女，您配偶的兄弟姐妹及其配偶、子女
5. Other relative 其他亲属
6. Paid helper (such as nanny) 雇佣人员（如保姆）
7. Volunteer or Employee of facility 志愿者或者志愿机构人员
8. Nursing home 养老院人员
9. Community 社区提供的帮助
10. Other 其他人员

**PROCEDURE 程序**： If DB031= 2, ask DB031_W3_1

DB031_W3_1 Father, mother, father-in-law, mother-in-law, who will help you in future? 在父母、岳父母、公公、婆婆中，以后会帮助您的是哪些？

1. Father 父亲
2. Mother 母亲
3. Father-in-law 岳父/公公
4. Mother-in-law 岳母/婆婆

**PROCEDURE 程序**：If DB031= 3, ask DB031_W3_2

DB031_W3_2 For the children, children-in-law, grandchildren who will help you in future, which children's family are they from? 以后会帮助您的子女、儿媳/女婿、孙子女/外孙子女，是以下哪个子女家的？

1-25 [Preload children's name] [加载子女姓名]
26 None of the above 以上都没有

[For helpers from each children's family, repeat the question 对来自每个子女家的帮助者，循环询问]

DB031_W3_3 For the family members of [Preload children's name], who will help you in person in future? [加载子女姓名] 家的哪些人以后会亲自帮助您？

1 [Preload children's name] himself/herself [加载子女姓名] 本人
2 [Preload children's name] his/her spouse [加载子女姓名] 的配偶
3 [Preload children's name] his/her children, your grandchildren, the number of them who will help you in person [加载子女姓名] 的孩子，即您的（外）孙子女，亲自帮助您的 [加载子女姓名] 的孩子有_____个 (**DB031_W3_3_1**)

**PROCEDURE 程序**：If DB031= 4, ask DB031_W3_4

DB031_W3_4 For the siblings, spouse and children of siblings, spouse's siblings, spouse and children of spouse's siblings who will help you in future, which children's family are they from? 以后会帮助您的兄弟姐妹及其配偶、子女，您配偶的兄弟姐妹及其配偶、子女，是以下哪个兄弟姐妹家的？

1-15 [Preload siblings' name] [加载兄弟姐妹的姓名]
16-30 [Preload siblings' name of spouse] [加载配偶的兄弟姐妹的姓名]
99 None of the above 以上都没有

[For helpers from each siblings' family, siblings of spouse's family, repeat the question 对来自每个兄弟姐妹、配偶兄弟姐妹家的帮助者，循环询问]

DB031_W3_5 For the family members of [Preload name of siblings, siblings of spouse], who will help you in person in future? [加载兄弟姐妹姓名/配偶兄弟姐妹的姓名] 家的哪些人以后会亲自帮助您？

1 [Preload name of siblings (of spouse)] himself/herself [加载（配偶的）兄弟姐妹姓名] 本人

2 [Preload name of siblings (of spouse)] his/her spouse [加载（配偶的）兄弟姐妹姓名]的配偶

3 For the children of [Preload name of siblings (of spouse)] who will help you in person, how many of the children of [Preload name of siblings (of spouse)] will help you [加载（配偶的）兄弟姐妹姓名] 的孩子，亲自帮助您的 [加载（配偶的）兄弟姐妹姓名] 的孩子有_____个 (**DB031_W3_5_1**)

**PROCEDURE 程序**：If DB031= 5, ask DB031_W3_6

DB031_W3_6 The number of other relatives who will help you in person in future, and what's their relationship with you? 以后亲自为您提供帮助的其他亲属共有_____位，都是您什么人？_____(**DB031_W3_6_1**)

**PROCEDURE 程序**：If DB031= 10, ask DB031_W3_7

DB031_W3_7 The number of others who will help you in person in future, what's their relationship with you? 以后亲自为您提供帮助的其他人共有_____位，都是您什么人？_____(**DB031_W3_7_1**)

DB035_W3_1 Did you hire a nurse or hourly worker last month? 过去一个月，您家是否请了保姆或小时工？（多选题）[If DB022_W3_1= 6, show IWER: paid helper (such as nanny) provided help for the respondent 显示访员注意：前面已经问到雇佣人员（如保姆）为该受访者提供了帮助]

1. Nurse 保姆
2. Hourly worker 小时工
3. None 都没有

[访员注意：（1）"小时工"，指按照小时计费的受雇者，如受访家庭雇的做饭、做清洁的人。"小时工"一般完成工作以后就离开，不住在受访家庭。"小时工"，又叫钟点工，是指在法定劳动年龄内，存在雇佣关系的劳动者，受雇于同一雇主的劳动时间不超过 5 个小时，劳动报酬以小时作为计算单位的一种非全日工作制用工形式。（2）"保姆"通常是按月计费的受雇者，和小时工所做的工作略有区别，一般住在受雇家庭。（3）农村家庭在农忙时，请的短期帮工如帮助收割的帮工也算小时工]

**PROCEDURE 程序**：If DB035_W3_1= 1, ask DB035_W3_2-DB035_W3_3; If DB035_W3_1= 2, ask DB035_W3_5-DB035_W3_6

DB035_W3_2 How much is the total wage of nurse last month? 上个月发给保姆的工资总额是多少？_____ Yuan 元

[IWER: The "total wage" means the amount of money paid to nurse, including the equivalent value of goods paid to nurse 访员注意："工资总额"指以货币或折合为货币的实物支付给保姆的劳动报酬数额]

DB035_W3_3 How many months did this nurse work for you? 这个保姆已经在您家干了几个月？_____ Month 月

[IWER: Record one hour if it was less than one hour. If you had more than one nurse, record the working hours of the nurse who worked for the longest time 访员注意：不满 1 个月按 1 个月来计算，如果有多个保姆，填写工作时间最长的那个保姆的工作时间]

DB035_W3_5 How long did the hourly worker work per week last month? 上个月，小时工平均每周的工作总时间为多少小时？_____ Hour 小时

[IWER: (1) The total working hours of all hourly workers would be recorded one hour if it was less than one hour. (2) If you hired more than one hourly worker, the "total working hours" means the working time of all hourly workers 访员注意：（1）如果所有小时工平均每周工作时间总和在 1 小时以下，按照 1 小时计算。（2）如果雇佣 1 个以上小时工，该处"总时间"指的是所有小时工工作的时间总和]

DB035_W3_6 How much is the wage per hour of hourly worker last month? 上个月，小时工平均每小时报酬是多少_____ Yuan 元

[IWER: If you hired more than one hourly worker, the "wage per hour" means the total payments to all hourly workers divided by the total working hours 访员注意：如果雇佣 1 个以上小时工，该处"平均每小时报酬"指 1 个月内支付给所有小时工的报酬总额分摊到全部工作小时的平均值]

DB036_W3 Do you unable to do some kind of work or cannot do that work for a long time because of disability or health reasons? 您有没有因为残疾或者健康原因，使您无法从事某种工作或者劳动，或者使您无法长时间工作或者劳动？

1. Yes 是
2. No 否
3. Too old for work 年龄太大，不能工作

**PROCEDURE 程序**：If DB036_W3= 1, skip to DB038_W3_1; If DB036_W3= 2 or 3, ask DB037_W3

DB037_W3 Do you unable to do some kind of housework or cannot do that housework for a long time because of disability or health reasons? 您有没有因为残疾或者健康原因，使您无法干某种家务，或者无法长时间做家务？

1. Yes 是 → Skip to DB038_W3_2
2. No 否
3. Too old for work 年龄太大，不能工作

DB038_W3_1 Do you unable to do work completely because of disability or health reasons? 您有没有由于残疾或健康原因，使您完全不能工作吗？

1. Yes 是
2. No 否 → Skip to DB039_W3

DB038_W3_2 Do you unable to do housework completely because of disability or health reasons? 您有没有由于残疾或健康原因，使您完全不能做家务吗？

1. Yes 是 → Skip DB039_W3 跳过DB039_W3
2. No 否

DB039_W3 Do you able to work for 8 hours a day? Or just work for a few time? 您是否有能力一天 8 小时正常工作或者劳动，还是仅仅能干少量时间？

1. Yes 是
2. No 否

DB032 How often did the respondent receive assistance in answering this section 受访者填写该部分问卷时是否求助？[IWER: If it is answered by a proxy, please record the respondent's reaction 访员注意：如果是协助回答，请记录受访者的反应]

1. Never 从不
2. A few times 有一些时候
3. Most or all of the time 大多数时候
4. The section was completed by a proxy respondent（the respondent is absent）受访者不在场，完全请人代填 → Skip to DB033 请跳至DB033

DB033 What is your relationship to R? 您和受访者是什么关系？[IWER: What is the proxy's relationship to R? If unknown, please ask the proxy 访员注意：代填问卷的人和受访者是什么关系。如果不清楚，请问代填者]

1. Spouse 配偶
2. Mother 母亲
3. Father 父亲
4. Mother-in-law 岳母/婆婆
5. Father-in-law 岳父公公
6. Sibling 兄弟姐妹
7. Brother-in-law, sister-in-law 姐夫妹夫/嫂子弟媳
8. Child 孩子
9. Spouse of child 孩子的配偶
10. Grandchild 孙子女
11. Other relative 其他亲戚
12. Helper or other non-relative 帮忙的人或者其他非亲属

DB034 [IWER: Please record the reason for proxy 访员注意：请记录代答的原因]
What is the main reason for proxy（the respondent is absent）？受访者不在场，完全请人代填的主要原因是什么？

1. The respondent has serious physical handicaps 受访者有严重身体障碍
2. The respondent has serious mental handicaps 受访者有严重精神障碍
3. The respondent has rejected this interview. 受访者拒访
4. Other 其他_____(**DB034_1**)

DC COGNITION & DEPPRESSION 认知和抑郁

[IWER: If DB032 = 4, then skip to Section E, health care and insurance. Sections DC must not be answered by proxy respondents 访员注意：如果DB032 = 4，请跳至 E 部分医疗保健与保险。DC 部分不能由别人代答]

DC001 Now I'm going to ask several simple questions. Some may be easy and some may be hard to answer. Please try to answer as honestly as you can. Are you ready? Please tell me today's date. （Check all that apply）现在我将问几个问题，有些问题对于您来说可能很简单、有些问题可能就比较难。请告诉我今天的日期，是哪一年哪一月哪一日？（可多选）[IWER: R doesn't have to answer in this order. If R is an elderly person and marked the date by lunar calendar, that date is correct if it matches with the solar calendar. You can check the accuracy, using the converter 受访者不必按顺序回答，如果受访者是老人，并按农历来回答，而且如果日期对应的阳历是准确的，那么您可以按日历转换来检查其准确性]

1. Year is correct 年正确
2. Month is correct 月正确
3. Day is correct 日正确

DC002 Please tell me the day of the week. Is it Monday, Tuesday, Wednesday, Thursday, Friday, Saturday, or Sunday? 请告诉我今天是星期几？

1. Day of week is correct 正确
2. Day of week is incorrect 错误

DC003 What is the current season (among Spring, Summer, Fall, or Winter)? 现在是什么季节？

1. Season is correct 季节正确
2. Season is incorrect 季节不正确

DC004 How would you rate your memory at the present time? Would you say it is excellent, very good, good, fair or poor? 您觉得自己现在的记忆力怎么样？是极好，很好，好，一般还是不好？

1. Excellent 极好
2. Very good 很好
3. Good 好
4. Fair 一般
5. Poor 不好

[We are going to read a list consisting of 10 words and we would like you to memorize as many as you can. We deliberately made the list long to make it difficult for anyone to memorize all of the words; most people will only remember a few of them. Please listen carefully as we read the list because we cannot repeat it. When we finish reading the list, we will ask you to recall and tell us as many words as you can remember, and they don't have to be in the

order that you heard them. Is this explanation clear? 我们将会给您读十个词，请仔细听，我们不会重复读。请尽可能多地记。我们是有意列这么多词以增加记忆的难度。读完后，会请您回忆这些词，不需要按顺序。您明白了吗？]

DC008 [CAPI automatically record the current time: hour and minute. CAPI 自动输入现在的时间：_____(**DC008_1**) 时_____(**DC008_2**) 分（24 小时制）]

DC006 Please tell me any of the words that you remember now. 请告诉我现在您还记得的词汇。[IWER: Answers are displayed only for interviewer. Please do not show the screen to R 访员注意：只有访员可以看到答案。请勿将显示屏展示给受访者看]

[Try to remember the words I just read to you. I'll ask you to recall them later 请尽量记住我刚才念给您听的词，一会我将请您回忆它们]

[The 10 items below refer to how you have felt and behaved during the last week. Choose the appropriate response 下面 10 道问题是有关您上周的感觉及行为，每道题目的答案都是一样的，包括很少或者根本没有，不太多，有时或者说有一半的时间还是大多数的时间，请您选择合适的答案]

DC009 I was bothered by things that don't usually bother me. 我因一些小事而烦恼。

1. Rarely or none of the time (< 1 day) 很少或者根本没有（< 1 天）
2. Some or a little of the time (1 – 2 days) 不太多（1 – 2 天）
3. Occasionally or a moderate amount of the time (3 – 4 days) 有时或者说有一半的时间（3 – 4 天）
4. Most or all of the time (5 – 7 days) 大多数的时间（5 – 7 天）

DC010 I had trouble keeping my mind on what I was doing. 我在做事时很难集中精力。

1. Rarely or none of the time (< 1 day) 很少或者根本没有（< 1 天）
2. Some or a little of the time (1 – 2 days) 不太多（1 – 2 天）
3. Occasionally or a moderate amount of the time (3 – 4 days) 有时或者说有一半的时间（3 – 4 天）
4. Most or all of the time (5 – 7 days) 大多数的时间（5 – 7 天）

DC011 I felt depressed. 我感到情绪低落。

1. Rarely or none of the time (< 1 day) 很少或者根本没有（< 1 天）
2. Some or a little of the time (1 – 2 days) 不太多（1 – 2 天）
3. Occasionally or a moderate amount of the time (3 – 4 days) 有时或者说有一半的时间（3 – 4 天）
4. Most or all of the time (5 – 7 days) 大多数的时间（5 – 7 天）

DC012 I felt everything I did was an effort. 我觉得做任何事都很费劲。

1. Rarely or none of the time (< 1 day) 很少或者根本没有（< 1 天）
2. Some or a little of the time (1 – 2 days) 不太多（1 – 2 天）
3. Occasionally or a moderate amount of the time (3 – 4 days) 有时或者说有一半的时间（3 – 4 天）

4. Most or all of the time (5 – 7 days) 大多数的时间（5 – 7 天）

DC013 I felt hopeful about the future. 我对未来充满希望。

1. Rarely or none of the time (< 1 day) 很少或者根本没有（< 1 天）
2. Some or a little of the time (1 – 2 days) 不太多（1 – 2 天）
3. Occasionally or a moderate amount of the time (3 – 4 days) 有时或者说有一半的时间（3 – 4 天）
4. Most or all of the time (5 – 7 days) 大多数的时间（5 – 7 天）

DC014 I felt fearful. 我感到害怕。

1. Rarely or none of the time (< 1 day) 很少或者根本没有（< 1 天）
2. Some or a little of the time (1 – 2 days) 不太多（1 – 2 天）
3. Occasionally or a moderate amount of the time (3 – 4 days) 有时或者说有一半的时间（3 – 4 天）
4. Most or all of the time (5 – 7 days) 大多数的时间（5 – 7 天）

DC015 My sleep was restless. 我的睡眠不好。

1. Rarely or none of the time (< 1 day) 很少或者根本没有（< 1 天）
2. Some or a little of the time (1 – 2 days) 不太多（1 – 2 天）
3. Occasionally or a moderate amount of the time (3 – 4 days) 有时或者说有一半的时间（3 – 4 天）
4. Most or all of the time (5 – 7 days) 大多数的时间（5 – 7 天）

DC016 I was happy. 我很愉快

1. Rarely or none of the time (< 1 day) 很少或者根本没有（< 1 天）
2. Some or a little of the time (1 – 2 days) 不太多（1 – 2 天）
3. Occasionally or a moderate amount of the time (3 – 4 days) 有时或者说有一半的时间（3 – 4 天）
4. Most or all of the time (5 – 7 days) 大多数的时间（5 – 7 天）

DC017 I felt lonely. 我感到孤独。

1. Rarely or none of the time (< 1 day) 很少或者根本没有（< 1 天）
2. Some or a little of the time (1 – 2 days) 不太多（1 – 2 天）
3. Occasionally or a moderate amount of the time (3 – 4 days) 有时或者说有一半的时间（3 – 4 天）
4. Most or all of the time (5 – 7 days) 大多数的时间（5 – 7 天）

DC018 I could not get ”going”. 我觉得我无法继续我的生活。

1. Rarely or none of the time (< 1 day) 很少或者根本没有（< 1 天）
2. Some or a little of the time (1 – 2 days) 不太多（1 – 2 天）
3. Occasionally or a moderate amount of the time (3 – 4 days) 有时或者说有一半的时间（3 – 4 天）
4. Most or all of the time (5 – 7 days) 大多数的时间（5 – 7 天）

[IWER: Try to persuade R to answer if R refuses at first. Record the exact number R says 访员注意：尽力说服受访者作答。正确地记录受访者回答的数字]

DC019 Let's try some subtraction of numbers this time. What does 100 minus 7 equal? 我们将问您一些减法。100 减去 7 等于多少？ _____

DC020 And 7 from that? （加载DC019答案）再减去 7 等于多少？ _____

DC021 And 7 from that? （加载DC020答案）再减去 7 等于多少？ _____

DC022 And 7 from that? （加载DC021答案）再减去 7 等于多少？ _____

DC023 And 7 from that? （加载DC022答案）再减去 7 等于多少？ _____

DC024 [IWER: Please indicate whether the respondent used paper and pencil or any other aid when completing the number subtraction 访员注意：受访者在回答这些算术题时，是否用了纸、笔或其他辅助工具]

1. Used aid 用了辅助工具
2. Did not use aid 没用辅助工具

DC025 Do you see this picture? Please draw that picture on this paper. 你看到这张图片了吗？请在这张纸上把该图片画出来。[IWER: Show the picture of two pentagons overlapped 访员注意：出示一张两个五角星重叠的图片]

1. Drew the picture 画出了图片
2. Failed to draw the picture 不能画出该图片

DC026 [CAPI automatically record the current time: hour and minute. 输入现在的时间：_____(**DC026_1**) 时_____(**DC026_2**) 分（24 小时制）]

DC027 A little while ago, I read you a list of words and you repeated the ones you could remember. Please tell me any of the words that you remember now. 刚才我给您读了一些词汇，您也重复了您记得的词汇。请告诉我现在您还记得的词汇。[IWER: Answers are displayed only for interviewer. Please do not show the screen to R 访员注意：只有访员可以看到答案。请勿将显示屏展示给受访者看]

[IWER: Please look at the computer with interviewees and do these tests following the instructions. Interviewees could use pen and paper, but can not use computers or other devices for help 访员注意：以下测试过程请受访者共同看电脑屏幕，并直接按提示进行操作。受访者可以使用铅笔和纸作为辅助工具，但不得使用计算器等其他辅助工具]

[Now we will show you several series of numbers on the computer screen. In each series there will be one number that is missing. The missing number will be indicated by a question mark "?". Please look at the pattern of the numbers. Based on this pattern, tell me what the number that is missing 接下来，电脑屏幕上会显示的一行数字，其中有一位是空缺的，这个空缺的

数字使用“?”标记。请从左往右仔细观察这组数字，试着找出这组数字的规律，并按照这个规律填出空缺处应有的数字]

PROCEDURE 程序:

DC031_W3_1, DC031_W3_2 and DC031_W3_3 are for all respondents. 所有的受访者回答DC031_W3_1, DC031_W3_2和DC031_W3_3

DC031_W3_1 7，8，_____，10

DC031_W3_2 8，_____，12，14

DC031_W3_3 18，10，6，_____，3

PROCEDURE 程序:

DC032_W3_1, DC032_W3_2 and DC032_W3_3 are for respondents who got 0 (zero) question correct in the first 3 questions (DC031_W3_1-DC031_W3_3) 如果受访者在最开始的三道题目（DC031_W3_1-DC031_W3_3）中回答正确的题数为 0，回答DC032_W3_1, DC032_W3_2 和DC032_W3_3

DC032_W3_1 1，2，3，_____

DC032_W3_2 6，5，4，_____

DC032_W3_3 12，_____，16，18

PROCEDURE 程序:

DC033_W3_1, DC033_W3_2 and DC033_W3_3 are for respondents who got 1 (one) question correct in the first 3 questions (DC031_W3_1-DC031_W3_3) 如果受访者在最开始的三道题目（DC031_W3_1-DC031_W3_3）中回答正确的题数为 1，回答DC033_W3_1, DC033_W3_2 和DC033_W3_3

DC033_W3_1 5，_____，3，2

DC033_W3_2 4，7，10，_____

DC033_W3_3 _____，4，6，8

PROCEDURE 程序:

DC034_W3_1, DC034_W3_2 and DC034_W3_3 are for respondents who got 2 (two) question correct in the first 3 questions (DC031_W3_1-DC031_W3_3) 如果受访者在最开始的三道题目（DC031_W3_1-DC031_W3_3）中回答正确的题数为 2，回答DC034_W3_1, DC034_W3_2 和DC034_W3_3

DC034_W3_1 1，3，3，5，7，7，_____

DC034_W3_2 3，_____，8，12，17

DC034_W3_3 17， _____， ,12， 8

PROCEDURE 程序:
DC035_W3_1, DC035_W3_2 and DC035_W3_3 are for respondents who got 3 (three) question correct in the first 3 questions (DC031_W3_1-DC031_W3_3) 如果受访者在最开始的三道题目（DC031_W3_1-DC031_W3_3）中回答正确的题数为 3，回答DC035_W3_1, DC035_W3_2 和DC035_W3_3

DC035_W3_1 10， _____， 3， 1

DC035_W3_2 18， 17， 15， _____， 8

DC035_W3_3 3， 3， 4， 6， 6， 7， _____， _____

DC042_W3 How satisfied are you with your health? 您对您的健康满意吗？
1. Completely satisfied 极其满意
2. Very satisfied 非常满意
3. Somewhat satisfied 比较满意
4. Not very satisfied 不太满意
5. Not at all satisfied 一点也不满意

PROCEDURE 程序:
If BE001 = 1 or 2, ask DC043_W3

DC043_W3 How satisfied are you with your marriage (relationship with spouse)? 您对您的婚姻满意吗？也就是说您对您和您配偶的关系满意吗？
1. Completely satisfied 极其满意
2. Very satisfied 非常满意
3. Somewhat satisfied 比较满意
4. Not very satisfied 不太满意
5. Not at all satisfied 一点也不满意
6. No spouse now 现在没有配偶

DC044_W3 How satisfied are you with your relationship with children? 您对您和您子女的关系满意吗？[访员注意：仅对当前有存活子女的受访者询问此问题]
1. Completely satisfied 极其满意
2. Very satisfied 非常满意
3. Somewhat satisfied 比较满意
4. Not very satisfied 不太满意
5. Not at all satisfied 一点也不满意
6. No child now 现在没有子女

DC028 Please think about your life-as-a-whole. How satisfied are you with it? Are you completely satisfied, very satisfied, somewhat satisfied, not very satisfied, or not at all satisfied? 总体来看，您对自己的生活是否感到满意？是极其满意，非常满意，比较满意，不太满意还是一点也不满意？

1. Completely satisfied 极其满意
2. Very satisfied 非常满意
3. Somewhat satisfied 比较满意
4. Not very satisfied 不太满意
5. Not at all satisfied 一点也不满意

This page intentionally left blank

E HEALTH CARE AND INSURANCE 医疗保健与保险

CARD21 ：Health facilities 医疗机构

1. General hospital 综合医院（即全科医院，不包括中医院）
2. Specialized hospital 专科医院（不包括中医院）
3. Chinese medicine hospital 中医院
4. Community healthcare center 社区卫生服务中心
5. Township hospital 乡镇卫生院
6. Health care post 卫生服务站
7. Village clinic/private clinic 村诊所/私人诊所
8. Other 其他

CARD20 ：Health insurance 医疗保险

1. Urban employee medical insurance (yi-bao) 城镇职工医疗保险（医保）
2. Urban resident medical insurance 城镇居民医疗保险
3. New cooperative medical insurance (he-zuo-yi-liao) 新型农村合作医疗保险（合作医疗）
4. Urban and rural resident medical insurance 城乡居民医疗保险（合并城镇居民和新型农村合作医疗保险）
5. Government medical insurance (gong-fei) 公费医疗
6. Medical aid 医疗救助
7. Private medical insurance: purchased by work unit 商业医疗保险：单位购买
8. Private medical insurance: purchased by individual 商业医疗保险：个人购买
9. Urban non-employed persons's health insurance 城镇无业居民大病医疗保险
10. Other medical insurance (specify) 其他医疗保险（请注明）
11. No insurance 没有保险

CARD23 ：Health facilities for inpatient care 住院治疗的医疗机构

1. General Hospital 综合医院（即全科医院，不包括中医院）
2. Specialized hospital 专科医院（不包括中医院）
3. Chinese Medicine Hospital 中医院
4. Community Healthcare Center 社区卫生服务中心
5. Township Hospital 乡镇卫生院
6. Health care post 卫生服务站
7. Other 其他

PART I MEDICAL INSURANCE 第一部分：医疗保险

Now we would like to know about health insurance or benefits that you might have. 现在我们想了解一下您享受的健康保险或福利。

[Show Card 出示卡片 14]

EA001 Are you the policy holder/primary beneficiary of any of the types of health insurance listed below? (circle all that apply) 您本人目前是否参加了以下医疗保险？（可多选）

1. Urban employee medical insurance (yi-bao) 城镇职工医疗保险（医保）
2. Urban resident medical insurance 城镇居民医疗保险
3. New cooperative medical insurance (he-zuo-yi-liao) 新型农村合作医疗保险（合作医疗）
4. Urban and rural resident medical insurance 城乡居民医疗保险（合并城镇居民和新型农村合作医疗保险）
5. Government medical insurance (gong-fei) 公费医疗
6. Medical aid 医疗救助
7. Private medical insurance: purchased by work unit 商业医疗保险：单位购买
8. Private medical insurance: purchased by individual 商业医疗保险：个人购买
9. Urban non-employed persons's health insurance 城镇无业居民大病医疗保险
10. Other medical insurance (specify) 其他医疗保险，请注明 ____ (**EA001_1**)
11. No insurance 没有保险 Skip to EA009 请跳至 EA009

[Soft check: If pick 11, cannot pick any other, "you chose no insurance and a specific type of insurance, this is not possible" 如果选 11，不能选其他项，"您不能既选择没有保险，又选择有某一种保险"]

F1:（1）"城镇职工医疗保险"指依法对城镇职工的基本医疗权利给予保障的社会医疗保险制度。

(2)"城镇居民医疗保险"指 2007 年 7 月国务院决定在全国 79 个城市实行城镇居民基本医疗保险试点。参保范围：城镇非从业居民，包括：不属于城镇职工基本医疗保险制度覆盖范围的中小学阶段的学生（包括职业高中、中专、技校学生）、少年儿童和其他非从业城镇居民。

(3)"新型农村合作医疗保险"指由政府组织、引导、支持，农民自愿参加，个人、集体和政府多方筹资，以大病统筹为主的农民医疗互助共济制度。采取个人缴费、集体扶持和政府资助的方式筹集资金。

(4)"城乡居民医疗保险"指一些地区率先将城镇居民医疗保险与新型农村合作医疗合并，推行统一的城乡居民医疗保险制度。

(5)"外来务工人员医疗保险"指在某些地区在城镇职工基本医疗保险以外，专为外来务工人员设置的一种医疗保险制度。

(6)"公费医疗"指国家为保障国家工作人员身体健康而实行的一项社会保障制度。其享受对象主要是各级国家机关、党派、团体以及文化、教育、科研、卫生、体育等事业单位的工作人员和离退休人员，二等乙级以上革命伤残军人以及在校大学生等。

(7)"医疗救助"指在政府的支持下，依靠社会力量建立的旨在向困难群体提供某些或全部基本医疗服务的制度。

(8)"商业医疗保险"是相对于社会保险而言的。商业医疗保险由商业保险公司经办，以营利为目的，企业或职工自愿参加。

(9)"其他医疗保险"如中国职工保险互助会《在职职工重大疾病互助保障计划》和《生育保险》。

(10)"医疗救助"在部分区县试点，救助标准是由各区县根据自身经济情况制定，导致救助标准有高有低。主要三种，一是提供社会医疗救助金，给救助对象以经济补偿；二是给医疗机构一定的经济补贴，使后者直接减免救助对象的部分医疗费；三是由社会医疗救助机构举办专门医疗机构，免费为救助对象提供医疗服务。

(11)"医疗保险"是指基本医疗保险，是为补偿疾病所带来的医疗费用的一种保险。职工因疾病、负伤、

生育时，由社会或企业提供必要的医疗服务或物质帮助的社会保险。如中国的公费医疗、劳保医疗。
(12) 城镇无业居民大病医疗保险参保范围是具有本市非农业户籍，男满 16 周岁不满 60 周岁，女满 16 周岁不满 50 周岁，未纳入城镇职工基本医疗保险覆盖范围的居民。筹资标准为每人每年 700 元，其中个人缴纳 600 元、财政补助 100 元。

For each circled type of insurance (1-10), ask the following questions EA002 - EA008. 对以上选择的 1-10 的保险类型，回答以下问题EA002 - EA008。

**PROCEDURE 程序**：IF EA001 = 7 or 8, skip EA002 如果参保的是商业保险，不询问是否参加补充医保

EA002 Do you have supplemental insurance to this plan? 你有没有参加补充医疗保险？（例如大病医疗等）

1. Yes 有
2. No 无

F1：“补充医疗保险”指由于国家的基本医疗保险只能满足参保人的基本医疗需求，超过基本医疗保险范围之外的医疗需求可以通过补充医疗保险予以补充。是相对于基本医疗保险而言的，包括企业补充医疗保险、商业医疗保险、社会互助和社区医疗保险等多种形式。

EA003 Where did you set up your insurance account/policy? 您的保险是在哪里办的?

1. This county 本县/市
2. (if it is not in this county) the place of your hukou （如户口不在本县/市）户籍所在地
3. Other 其它 ______(**EA003_1**)province 省 ________(**EA003_2**)county 市/县

EA005 Through which agency did you purchase your primary plan? 您的主要保险是通过什么机构办理的？

1. Community committee/ village committee 居委会/村委会
2. Work unit (incl. village collective) 单位
3. Agency of Social insurance 社保经办机构
4. Private insurance company 商业保险机构
5. Other 其他

EA006 What's your out-of-pocket yearly premium? 每年您要自己缴纳多少保险费 (包括主保险费和补充医疗保险费)？

1. 1 ____Yuan 元 (**EA006_1**) [soft check upper limit: 15,000 for choice 1, 3,000 choice 2, 1,000 choice 3, 1,500 choice 4, 4,000 choice 7, 2,500 choice 9 程序检查：上限：选项 1，15,000；选项 2，3,000；选项 3，1,000；选项 4，1,500；选项 7，4,000；选项 9，2,500]
2. 2 无需缴纳保险费

EA007 Who pays the premium for you? (choose all that apply) 谁给您交保费？(可多选)

1. Myself 自己

2. Children 子女
3. Relatives 亲属
4. Government 政府
5. Work unit (incl. village collective) 单位（包括村集体）
6. Loan 借款
7. Donate 捐赠
8. Others 其他 Specify 请注明 (**EA007_1**)

EA008 When did this benefit begin? 您是什么时候参加这份保险的？[IWER: Mark the year using four digits. Take down the month as its actual number. For example, write January as "1" not "01", December as "12". If do not remember month, fill '0'. 访员注意：用 4 位数表示年，按照实际的月份填写月。例：1 月写作 "1"，而不是 "01"，12 月写作 "12"。如果记不住月份，请填入 '0'。]

1900...2015Year 年 (**EA008_1**)____, 0...12Month 月 (**EA008_2**)____

EA009 What is your main reason for not having health insurance? 您目前没有参加任何医疗保险的主要原因是什么？
1. I do not need it 不需要
2. Cannot afford it 支付不起保险费
3. Do not know where or from whom to get it 不知道该去哪办
4. Do not trust the institutions that offer health insurance 不相信健康保险机构
5. Do not have suitable programs for me to buy 没有合适的保险项目
6. Do not know/never thought of it 从没有想过这个问题
7. Others 其他原因 (**EA009_1**)

**PROCEDURE 程序**：Skip to EC001 for new R 如果是新受访者，请跳至EC001

**PROCEDURE 程序**：For XRType = REIW who had health insurance in last wave 上期参保的回访受访者：

EA001_W3_1 Our records from your last interview show that you have had [preload health insurance], is this right? [加载上次访问日期] 的访问记录显示您当时参加了 [医疗保险加载]，这个记录对吗？
1. Yes 是
2. No 否

EA001_W3_2 Are you still the policy holder/primary beneficiary of any of the types of health insurance [preload health insurance]? [您现在是不是仍然参加 [医疗保险加载]
1. Yes 是
2. No 否

EA001_W3_3 Between the last interview and today, were you the policy holder/primary beneficiary of any of the types of health insurance [preload health insurance]? [自上次访问以后，到目前您是否参加了 [医疗保险加载]

1. Yes 是
2. No 否

F1：(1)“城镇职工医疗保险”指依法对城镇职工的基本医疗权利给予保障的社会医疗保险制度。
(2)“城镇居民医疗保险”指 2007 年 7 月国务院决定在全国 79 个城市实行城镇居民基本医疗保险试点。参保范围：城镇非从业居民，包括：不属于城镇职工基本医疗保险制度覆盖范围的中小学阶段的学生（包括职业高中、中专、技校学生）、少年儿童和其他非从业城镇居民。
(3)“新型农村合作医疗保险“指由政府组织、引导、支持，农民自愿参加，个人、集体和政府多方筹资，以大病统筹为主的农民医疗互助共济制度。采取个人缴费、集体扶持和政府资助的方式筹集资金。
(4)“城乡居民医疗保险”指一些地区率先将城镇居民医疗保险与新型农村合作医疗合并，推行统一的城乡居民医疗保险制度。
(5)“外来务工人员医疗保险”指在某些地区在城镇职工基本医疗保险以外，专为外来务工人员设置的一种医疗保险制度。
(6)“公费医疗”指国家为保障国家工作人员身体健康而实行的一项社会保障制度。其享受对象主要是各级国家机关、党派、团体以及文化、教育、科研、卫生、体育等事业单位的工作人员和离退休人员，二等乙级以上革命伤残军人以及在校大学生等。
(7)“医疗救助”指在政府的支持下，依靠社会力量建立的旨在向困难群体提供某些或全部基本医疗服务的制度。
(8)“商业医疗保险”是相对于社会保险而言的。商业医疗保险由商业保险公司经办，以营利为目的，企业或职工自愿参加。
(9)“其他医疗保险”如中国职工保险互助会《在职职工重大疾病互助保障计划》和《生育保险》。
(10)“医疗救助”在部分区县试点，救助标准是由各区县根据自身经济情况制定，导致救助标准有高有低。主要三种，一是提供社会医疗救助金，给救助对象以经济补偿；二是给医疗机构一定的经济补贴，使后者直接减免救助对象的部分医疗费；三是由社会医疗救助机构举办专门医疗机构，免费为救助对象提供医疗服务。
(11)“医疗保险”是指基本医疗保险，是为补偿疾病所带来的医疗费用的一种保险。职工因疾病、负伤、生育时，由社会或企业提供必要的医疗服务或物质帮助的社会保险。如中国的公费医疗、劳保医疗。
(12) 城镇无业居民大病医疗保险参保范围是具有本市非农业户籍，男满 16 周岁不满 60 周岁，女满 16 周岁不满 50 周岁，未纳入城镇职工基本医疗保险覆盖范围的居民。筹资标准为每人每年 700 元，其中个人缴纳 600 元、财政补助 100 元。

PROCEDURE 程序：For each circled type of insurance (1-10), ask EA008_W2_1 and EB003
针对每种所选的 1-10 的保险，分别问EA008_W2_1 and EB003

EA008_W2_1 When did you start participating in this insurance? 您是什么时候参加这份保险的？[IWER: Mark the year using four digits. Take down the month as its actual number. For example, write January as “1” not “01”, December as “12”. If do not remember month, fill ‘0’. 访员注意：用 4 位数表示年，按照实际的月份填写月。例：1 月写作“1”，而不是“01”,12 月写作“12”。如果记不住月份，请填入’0’。]
1900...2013 Year 年 (**EA008_W2_1_1**)____, 0...12 Month 月 (**EA008_W2_1_2**)____

EB003 When did you stop participating in this insurance? 请问您什么时候退出这种保险的？[IWER: Mark the year using four digits. Take down the month as its actual number.

For example, write January as "1" not "01", December as "12". If do not remember month, fill '0'. 访员注意: 用 4 位数表示年，按照实际的月份填写月。例：1 月写作"1"，而不是"01",12 月写作"12"。如果记不住月份，请填入'0'。]
1900...2013 Year 年 (**EB003_1**)____, 0...12Month 月 (**EB003_2**)____

EB004 Why did you stop participating in this insurance? 请问您为什么会退出这种保险？
1. Employer no longer exists. 单位不存在了
2. Insurance not provided anymore locally. 本地不再提供这种保险
3. I resigned/was fired. 我从单位辞职/被辞退了
4. Other (please specify____ 其它，请注明 ____(**EB004_1**)

PART II HEALTH CARE COSTS AND UTILIZATION 第二部分：医疗成本与使用情况

[IWER: Please do not allow proxy to answer Part II. 访员注意：“医疗成本与使用情况”这部分不允许请别人代为回答]

EC001 **PROCEDURE 程序**: For XRType = NEWIW 新的受访者
When did you take the last physical examination? 您最近一次常规体检时什么时候？
PROCEDURE 程序: For XRType = REIW 回访受访者
When did you take the last physical examination since R's LAST IW MONTH, YEAR/in the last two years? 自上次访问以来，您最近一次常规体检时什么时候？
[IWER: Mark the year using four digits. Take down the month as its actual number. For example, write January as "1" not "01", December as "12". If do not remember month, fill '0'. 访员注意: 用 4 位数表示年，按照实际的月份填写月。例：1 月写作"1"，而不是"01",12 月写作"12"。如果记不住月份，请填入 '0'。]
1. 1900...2013Year 年 (**EC001_1**)____0...12 Month 月 (**EC001_2**)____
2. Didn't ever take physical examination yet 一辈子都没有参加过体检
3. Didn't take physical examination last two years 过去两年没有参加过常规体检

EC001_W3_1 Which item do you take in this physical examination? 这次常规体检，您检查了哪些项目？
1. Physical examination 体格检查
2. Routine blood test 血常规
3. Routine urine test 尿常规
4. Liver function test 肝功能
5. Kidney function test 肾功能
6. Lipids profile test 血脂三项
7. Blood glucose test 空腹血糖
8. Surgical 外科

9. Internal medicine 内科
10. Five sense organ test 五官科
11. Electrocardiogram 心电图
12. B-type ultrasonic 腹部 B 超
13. Chest fluoroscopy 胸部透视
14. Male or female specialist 男女专科
15. Other 其他 ____, please specify 请注明 ____(**EC001_W3_2**)

EC002 Who paid for the physical examination? 谁付的体检费?

1. Myself 自己
2. Children 子女
3. Relatives 亲属
4. Government 政府
5. Work unit (incl. village collective) 单位（包括村集体）
6. Insurance 保险
7. Loan 借款
8. Donation 捐赠
9. Other 其他 ____, please specify 请注明 ____(**EC002_1**)

EC003_W3 Did you get public health service voucher last year? 在过去一年里，您是否领取过基本公共卫生服务券?

1. Yes 是 ____Yuan 元 (**EC004_W3**)RMB 元 [softcheck upper limit: 100000 上限: 100000]
2. No 否

The next questions pertain to medical facilities or medical providers you may have visited for outpatient care during the past 1 month (excluding hospitalization). 下面我们要问问您过去一个月，到门诊看病或者接受治疗的情况（不包括住院）。

ED001 In the last month have you visited a public hospital, private hospital, public health center, clinic, or health worker's or doctor's practice, or been visited by a health worker or doctor for outpatient care? 过去一个月里，您是否去医疗机构看过门诊或者接受过上门医疗服务? (不包括去医院做体检)

1. Yes 是 Skip to ED004请跳至ED004
2. No 否

ED002 Have you been ill in the last month? 您在过去一个月内生过病吗?

1. Yes 是
2. No 否

PROCEDURE 程序:

If ED001 = 2 and ED002 = 2 skip to EE001 如果ED001 = 2 并且 ED002 = 2, 请跳至EE001。

If ED001 = 1 skip to ED004 如果ED001 = 1, 请跳至ED004。

If ED002 = 1 and ED001 = 2 go to ED003. 如果ED002 = 1 并且ED001 = 2, 请跳至ED003。

ED003 What's the main reason for not seeking medical treatment? 您没有去看病的主要原因是什么？

1. Already under treatment 之前已经看过医生了
2. Illness is not serious, don't need treatment 病情不严重，不需要看医生
3. No money 没有钱看病
4. No time 没有时间看病
5. Inconvenient traffic 去医院交通不方便
6. Poor service in hospital 医院服务态度不好
7. Treatment is not useful 认为看医生没有用
8. Other 其他

[Show Card 出示卡片 15]

ED004 Which types of medical facilities have you visited in the last 4 weeks for outpatient treatment? (circle all that apply) 过去一个月，您去过哪些医疗机构接受门诊治疗？(可多选)

1. General hospital 综合医院（即全科医院，不包括中医院）
2. Specialized hospital 专科医院（不包括中医院）
3. Chinese medicine hospital 中医院
4. Community healthcare center 社区卫生服务中心
5. Township hospital 乡镇卫生院
6. Health care post 卫生服务站
7. Village clinic/Private clinic 村诊所/私人诊所
8. Other 其他

**PROCEDURE 程序**：For each item 1-7 checked in ED004, ask ED005：对ED004 选择的 1-7 的每一种医疗机构，提问ED005：

ED005 How many times did you visit/been visited by [...] during the last month? 过去一个月中，您去这家医疗机构看过几次门诊？ ____Times 次

**PROCEDURE 程序**：If sum(ED005)> 1, then ask ED006; otherwise, skip ED006。如果ED005 中的次数总和 > 1，则问ED006；否则，就跳过ED006。

ED006 How much did all the visits to [ED004 answer] cost during the last month? 您过去一个月去 [ED004 answer] 看病的总费用大概是多少？ [IWER: If possible, please check the list of cost. 访员注意：如果可能，查看一下明细单]

1. Total cost 总费用 ____(ED006_1)Yuan 元; [soft check upper bound: 30,000 上限：30,000. Brackets50/100/200/500/1000]
2. Didn't pay anything 没有付任何钱

**PROCEDURE 程序**：IF ED006 = 1, ASK ED007

ED007 1. Self-paid part 其中自付部分 ____ (ED007_1)Yuan 元 [Brackets50/100/200/500/1000]

2. Didn't pay anything. 没有付任何钱

Now I'd like to ask you some questions about your most recent visit to a health care provider in the last month. 下面我想了解一下您过去一个月最近一次就诊的情况。

ED008 Which health care provider did you visit most recently during the past month? 过去一个月，您最近一次去了哪种医疗机构就诊？[CAPI：Preload the health care providers in ED004. 程序：加载 ED004填的医疗机构。]

ED009 Is this facility public or private? 这家机构是公立的还是私立的？

1. Public 公立的
2. Private 私立的

**PROCEDURE 程序：** If ED008 = 1 – 3, ask ED010 如果 ED008 = 1 – 3，请回答 ED010

ED010 What's the level of this facility? 这家医疗机构是什么级别的？

1. County/district 县/市/区级
2. Regional/city 地/市
3. Provincial/affiliated to a ministry 省/部属
4. Military 军队
5. Others 其他 ____ Specify 请注明 ____(**ED011**)
6. Not applicable 不适用

ED012 Did the provider visit you at home? 这次是不是医护人员上门服务？

1. Yes 是 skip ED013 to ED016 and ED022，跳过 ED013 到 ED016 和 ED022
2. No 否

ED014 What is the travel time (one-way) to that facility? 您上次去这家医疗机构单程花多少时间？Minutes 分钟 ____ (**ED014_1**)

How to go to the facility? 用什么方式去这家医疗机构？(**ED014_2**)

1. Walk 步行
2. Bus 公交车
3. Car 私家车或出租车
4. Ambulance 救护车
5. Bicycle or other manual vehicles 自行车或其他人力车
6. Electric bicycle/electric tricycle 电动自行车/电动三轮车
7. Motorcycle 摩托车
8. Tractor 拖拉机
9. Train 火车
10. Animal or animal-pulled cart 骑牲畜或坐畜力车

**PROCEDURE 程序：** If ED014 = 1, skip ED015 如果 ED014 = 1，跳过 ED015

ED015 What was the total transportation cost to the facility (including fuel cost, one way trip)? 您上次去这家医疗机构的单程交通成本是多少？（包括燃料费）____ RMB 元 [softcheck upper limit: 600 上限：600]

ED017 What was the purpose of your visit? (circle all that apply) 您上次去这家医疗机构的目的是什么？(可多选)

1. Immunization 免疫接种
2. Consultation 咨询
3. Medical check-up 体检
4. Treatment of illness 看病
5. Other 其他

**PROCEDURE 程序**：If ED017 = 4, then ask ED018 —ED021, ELSE ASK ED023. 如果ED017 答案中包含 4 "看病"，继续问ED018 —ED021 的问题，否则提问ED023。

ED018 Could you tell me the disease name? 您能告诉我您得了什么病吗？ ____

ED019 Was the visit a first visit or a follow-up visit for the symptom? 这次看病是第一次就诊还是复诊？

1. First 第一次就诊
2. Follow-up 复诊

ED020 Was the visit for ordinary outpatient service or an emergency? 这次看病是普通门诊还是急诊？

1. Ordinary 普通门诊
2. Emergency 急诊

[Show Card 出示卡片 16]

ED021 What kind of treatment did you receive? (circle all that apply) 您这次去看病接受了以下哪种治疗？(可多选)

1. Injection 注射
2. Laboratorium test 化验
3. Surgery 手术
4. X-ray, CT, B ultrasonic, MRI X-光、CT、B 超、核磁共振等检查
5. Medication or retrieve medicine 用药、拿药
6. IV (Drip Infusion) 输液
7. Traditional treatment, e.g. massage, accupuncture 中医治疗，如按摩、针灸等
8. Other 其他

ED023 What was the total cost of this visit, including both treatment and medication cost (includes prescriptions you received)? 这次就诊（包括药费和诊疗费，药费包括在这家医疗机构或其他药店购买医生开的处方药）总共花了多少钱？

1. ____ RMB 元 (**ED023_1**) [softcheck upper limit 上限：30,000]
   [Brackets 25/50/120/400/1200]
2. There was no cost 没有费用

**PROCEDURE 程序**：IF ED023 = 1, ASK ED024

ED024 How much did you pay out of pocket, after reimbursement from insurance? 这次就诊所花的费用里，您自己支付了多少？

1. ____RMB 元 (**ED024_1**) [soft check upper limit: 30,000, also ED025 <= ED023_1, else "Pay out of pocket cannot be more than total cost" 上限：30,000，而且ED025 <= ED023_1，因为"自己支付的费用不可能多于总费用"]
2. Did not pay anything 没有付任何钱 Go to ED026 请跳至 ED026
   [Brackets 15/10/100/300/1000]

ED025 Who contribute most for paying the out-of-pocket cost? 自付部分谁给您交得最多？

1. Myself 自己
2. Children 子女
3. Relatives 亲属
4. Government 政府
5. Work unit (incl. village collective) 单位（包括村集体）
6. Loan 借款
7. Donation 捐赠
8. Other 其他 ____(**ED025_1**)

ED026 What was the total medication cost for this visit, including prescriptions you received? 这次就诊所付的药费 (包括在这家医疗机构或其他药店购买医生开的处方药) 一共是多少钱？

1. ____ RMB 元 (**ED026_1**) [soft check upper limit: 5,000, and must be no more than ED023_1. 上限：5,000，而且不能多于ED023_1] [Brackets 10/30/80/250/600]
2. Doctor did not write a prescription 没有开药 skip ED027 跳过ED027
3. Didn't fill prescription 开了方子，没有去取药 skip ED027 跳过ED027

ED027 How much will you eventually pay out of pocket for the medications from this visit, including prescriptions you received? 这次就诊所花的药费 (包括在这家医疗机构或其他药店购买医生开的处方药) 里，您自己支付了多少钱？

1. ____RMB 元 (**ED027_1**) [softcheck upper limit: 5,000 and ED027_1 must be no more than ED026_1 and no more than ED023_1. 上限：5,000 而且ED027_1 必须少于ED026_1，少于ED023_1] [Brackets 10/20/70/200/500]
2. Didn't pay anything 没有自付费用

[Show Card 出示卡片 14]

ED028 What insurance did you use or will you use? (circle all that apply) 这次就诊的费用（包含诊疗费和药费）您使用或将使用哪种医疗保险报销？（可多选）

1. Urban employee medical insurance (yi-bao) 城镇职工医疗保险（医保）
2. Urban resident medical insurance 城镇居民医疗保险
3. New cooperative medical insurance (he-zuo-yi-liao) 新型农村合作医疗保险（合作医疗）

4. Urban and rural resident medical insurance 城乡居民医疗保险（合并城镇居民和新型农村合作医疗保险）
5. Government medical insurance (gong-fei) 公费医疗
6. Medical aid 医疗救助
7. Private medical insurance: purchased by work unit 商业医疗保险：单位购买
8. Private medical insurance: purchased by individual 商业医疗保险：个人购买
9. Urban non-employed persons's health insurance 城镇无业居民大病医疗保险
10. Other medical insurance (specify) 其他医疗保险，请注明 ____(**ED028_1**)
11. Reimbursed by Work unit (incl. village collective) 单位报销
12. No insurance 没有保险
13. Not applicable 不适用

F1：（1）"城镇职工医疗保险"指依法对城镇职工的基本医疗权利给予保障的社会医疗保险制度。
(2)"城镇居民医疗保险"指 2007 年 7 月国务院决定在全国 79 个城市实行城镇居民基本医疗保险试点。参保范围：城镇非从业居民，包括：不属于城镇职工基本医疗保险制度覆盖范围的中小学阶段的学生（包括职业高中、中专、技校学生）、少年儿童和其他非从业城镇居民。
(3)"新型农村合作医疗保险"指由政府组织、引导、支持，农民自愿参加，个人、集体和政府多方筹资，以大病统筹为主的农民医疗互助共济制度。采取个人缴费、集体扶持和政府资助的方式筹集资金。
(4)"城乡居民医疗保险"指一些地区率先将城镇居民医疗保险与新型农村合作医疗合并，推行统一的城乡居民医疗保险制度。
(5)"外来务工人员医疗保险"指在某些地区在城镇职工基本医疗保险以外，专为外来务工人员设置的一种医疗保险制度。
(6)"公费医疗"指国家为保障国家工作人员身体健康而实行的一项社会保障制度。其享受对象主要是各级国家机关、党派、团体以及文化、教育、科研、卫生、体育等事业单位的工作人员和离退休人员，二等乙级以上革命伤残军人以及在校大学生等。
(7)"医疗救助"指在政府的支持下，依靠社会力量建立的旨在向困难群体提供某些或全部基本医疗服务的制度。
(8)"商业医疗保险"是相对于社会保险而言的。商业医疗保险由商业保险公司经办，以营利为目的，企业或职工自愿参加。
(9)"其他医疗保险"如中国职工保险互助会《在职职工重大疾病互助保障计划》和《生育保险》。
(10)"医疗救助"在部分区县试点，救助标准是由各区县根据自身经济情况制定，导致救助标准有高有低。主要三种，一是提供社会医疗救助金，给救助对象以经济补偿；二是给医疗机构一定的经济补贴，使后者直接减免救助对象的部分医疗费；三是由社会医疗救助机构举办专门医疗机构，免费为救助对象提供医疗服务。
(11)"医疗保险"是指基本医疗保险，是为补偿疾病所带来的医疗费用的一种保险。职工因疾病、负伤、生育时，由社会或企业提供必要的医疗服务或物质帮助的社会保险。如中国的公费医疗、劳保医疗。
(12)"单位报销"指一些福利较好的单位可以对本单位职工发生的除基本医疗保险报销部分以外的医疗费用进行全额或一定额度的报销。
(13) 城镇无业居民大病医疗保险参保范围是具有本市非农业户籍，男满 16 周岁不满 60 周岁，女满 16 周岁不满 50 周岁，未纳入城镇职工基本医疗保险覆盖范围的居民。筹资标准为每人每年 700 元，其中个人缴纳 600 元、财政补助 100 元。

ED029 Did you give any “red envelopes” to the doctors for this visit? 您最近一次就诊有没有给医生送红包？

1. Yes 是
2. No 否

The following questions pertain to hospitalization (inpatient care) that you have had during the past year. 下面我们想了解一下您过去一年接受住院治疗的情况。

EE001 In the past year, did a doctor suggest that you needed inpatient care but you did not get hospitalized? 过去一年，是否有医生说您应该住院而没有住院的情况？

1. Yes 有
2. No 没有 Skip to EE003 请跳至EE003

EE002 What's the main reason for not seeking inpatient care? 您没去住院的主要原因是什么？

1. Not enough money 没有钱
2. Not willing to go to the hospital 不愿意住院
3. Felt that hospital was unlikely to cure problem—hospital quality poor 住院也没有用，医院医疗条件差
4. Felt that care was unlikely to cure the problem—problem too serious 觉得问题很严重，住院也没用
5. No ward available 医院没有床位
6. Other 其他

EE003 Have you received inpatient care in the past year? 过去一年内，您住过院吗？

1. Yes 是
2. No 否 Skip to EF001 请跳至 EF001

EE004 How many times have you received inpatient care during the past year? 过去一年，您接受过几次住院治疗？ ____Times 次

**PROCEDURE 程序**： If EE004 = 1, skip to EE007. 如果EE004 = 1， 跳至EE007。

EE005 What was the medical cost for all the inpatient care you received during the past year? (Only include fees paid to the hospital, including ward fees but excluding wages paid to a hired nurse, transportation costs, and accommodation costs for yourself or family members.) 过去一年住院的总费用大概是多少？ （只包括付给医院的费用，不包括陪护的工资、自己或家人的交通费和住宿费，但包括医院病房费。）

1. Total cost 总费用 ____(**EE005_1**)Yuan 元; [Brackets 1500/3000/7000/15000/30000]

2. Didn't pay anything. 没有付任何钱。[softcheck upper limit 上限: 300,000]

**PROCEDURE 程序**： IF EE005 = 1 ASK EE006.

EE006

1. Self-paid part 其中自付部分 ____(**EE006_1**)Yuan 元
2. Didn't pay anything. 没有付任何钱

[softcheck upper limit: 300,000 and EE005 B.1<=EE005 A.1. 上限:300,000,而且EE005 B.1<=EE005 A.1.] [Brackets 600/1500/4000/8000/18000]

We want details about the last hospitalization you had in the past year. 我们想了解您在过去一年中，最后一次住院的详细情况。

PROCEDURE 程序: If ED001 = 1 ask EE007. 如果ED001 = 1 问EE007。

EE007 Is this the same facility as mentioned in ED008 for outpatient care? 这家医院是[ED008] 吗？
1. Yes 是 Skip to EE016 跳至EE016
2. No 否

PROCEDURE 程序: If EE007 = 2 or ED001 = 2 ask EE008 -EE015. 如果EE007 = 2 或者ED001 = 2 问EE008-EE015。

[Show Card 出示卡片 17]

EE008 What is the type of health service facility which you visited for last inpatient care (hospital admissions)/for your most recent hospitalization in the past year? 过去一年中，您最后一次接受住院治疗所在的医疗机构的类型是什么？
1. General Hospital 综合医院（即全科医院，不包括中医院）
2. Specialized hospital 专科医院（不包括中医院）
3. Chinese Medicine Hospital 中医院
4. Community Healthcare Center 社区卫生服务中心
5. Township Hospital 乡镇卫生院
6. Health care post 卫生服务站
7. Other 其他

EE009 Is this facility public or private? 这家医疗机构是公立的还是私立的？
1. Public 公立的
2. Private 私立的

PROCEDURE 程序: If EE008 = 1 − 3, ask EE010, 如果EE008 = 1 − 3, 请回答EE010。

EE010 What's the level of this facility? 这家医疗机构是什么级别的？
1. County/district 县/市/区级
2. Regional/city 地/市
3. Provincial/affiliated to a ministry 省/部属
4. Military 军队
5. Others 其他

EE012 What is the location of this facility? 这家医疗机构的具体的地址是什么？

1. province 省 (**EE012_1**)
   A. This province 本省
   B. Other province, (preload province) 外省 (选择省份) (**EE012_1_1**) [IWER: Choose from the list of provinces, see appendix 2 访员注意：请选择省份，见附表二]
2. county/city 县/市 (**EE012_2_1**)
   A. This county/city 本县/市
   B. Other county/city, specify 外县/市县/市 (**EE012_2_2**)
3. township/district 乡/区 (**EE012_3_1**)
   A. This township/district 本乡/ 区
   B. Other township/district, specify 外乡/区 (**EE012_3_2**) 乡/区
4. village/street 村/街道 (**EE012_1_1**)
   A. This village/street 本村/街道
   B. Other village/street, specify 外村/街道 (**EE012_4_2**)

EE013 How many kilometers is it from the medical facility to your residence? 从您家到这家医疗机构有几公里? ____ Km 公里 [softcheck upper limit 上限: 3000]

EE014 What is the travel time (one-way) to that facility? 您上次去这家医疗机构接受住院治疗单程花多少时间?

Unit 单位 (**EE014_2**)

1. ____Minute 分钟 [check range 范围: 1-59]
2. ____Hour 小时 [softcheck upper limit 上限: 20]

How to go to the facility? 用什么方式去这家医疗机构? (**EE014_3**)

1. Walk 步行
2. Bus 公交车
3. Car 私家车或出租车
4. Ambulance 救护车
5. Bicycle or other manual vehicles 自行车或其他人力车
6. Electric bicycle/electric tricycle 电动自行车/电动三轮车
7. Motorcycle 摩托车
8. Tractor 拖拉机
9. Train 火车
10. Animal or animal pulled cart 骑牲畜或坐畜力车

PROCEDURE 程序: IF EE014_2 $= 2 - 10$, ask EE015

EE015 What was the total transportation cost to the facility (including fuel cost, one way trip)? 您上次去这家医疗机构接受住院治疗的单程交通成本是多少钱? (包括燃料费) ____RMB 元 [soft check upper limit 上限: 600]

EE016 How many nights were you hospitalized there? 您在那里住了几晚? ____Nights 个晚上 [softcheck upper limit 上限: 40]

EE017 What was the starting date of your hospital stay? 您从哪一天开始住院?

____1900...2015 (**EE017_1**)Year 年 ____0...12 (**EE017_2**)Month 月 ____0...31 (**EE017_3**)Day 日 [IWER: Mark the year using four digits. Take down the month as its actual number. For example, write January as "1" not "01", December as "12". If do not remember month and day, fill '0'. 访员注意: 用 4 位数表示年，按照实际的月份填写月。例: 1 月写作"1"，而不是"01"，12 月写作"12"。如果记不住月份和日期，请填入'0'。]

EE018 What was your date of exit? 您哪天出院?

1. ____1900...2015 (**EE018_1**) Year 年 ____0...12 (**EE018_2**) Month 月 ____0...31 (**EE018_3**) Day 日
2. Still there 仍然在住院

[soft check: date of exit should be not before starting date, "exit date is before starting date, please ask R again", also exit date should be within 1 year of today 出院日期应该比住院日期要晚，"出院日期早于住院日期，请再问一次受访者"，出院日期应该在从今天往前数的一年之内。]

EE019 Why were you hospitalized? (Choose one choice) 您住院的原因是什么?（单选）

1. Sickness 生病
2. Accident 意外
3. Violence 暴力
4. Other 其他

EE020 Could you tell me the name of the disease? 您能告诉我们您是得了什么病住院的吗? ____

[Show Card 出示卡片 18]

EE021 During hospitalization, what kind of treatment did you receive? (circle all that apply) 在最近的一次住院期间，您接受了哪些治疗?（可多选）[IWER: Read one by one. 访问员注意: 请逐项读出以下答案。]

1. Medical check-up/consultation 体检或者咨询
2. Injection 注射
3. Laboratory test 化验
4. Surgery 手术
5. X-ray, CT, B ultrasonic, MRI X-光、CT、B 超、核磁共振等检查
6. Medication or medicine pick-up 用药、拿药
7. IV (drip infusion) 输液
8. Traditional treatment, e.g., massage, acupuncture 中医治疗，如按摩、针灸等
9. Delivery 分娩
10. Other 其他

**PROCEDURE 程序**: IF EE018 = 2, please skip EE022. 程序: 如果EE018 = 2，请跳过EE022。

EE022 Under what conditions did you leave the hospital? 您这次是在什么情况下出院的?

1. Fully recovered from illness, received doctor's approval 病完全好了，医生同意出院 Skip EE023 跳过EE023
2. Hadn't recovered from illness, but doctor suggested to leave 病没有完全好，医生建议出院 Skip EE023 跳过 EE023
3. Hadn't recovered from illness, requested to leave without doctor's suggestion 病没有完全好，自己要求出院
4. Other reasons 其他原因 skip EE023 请跳过 EE023

EE023 Why did you want to leave the hospital before you recovered? 您要求提前出院的原因是什么？

1. Can't recover from illness 久病不愈
2. Financial reason 经济困难
3. No space in the hospital 没有床位
4. Limited hospital conditions 医院医疗条件所限
5. Bad work attitute of medical personnel 医护人员服务态度不好
6. Other reasons 其他原因

EE024 What was the total medical cost of hospitalization? (Only include the fees paid to the hospital, excluding the wage of hired nurse, the fare or rent, but including the ward fees.) 这次住院一共花了多少钱？（只包括付给医院的费用，不包括陪护的工资、自己或家人的交通费和住宿费，但包括医院病房费。）

1. ____RMB 元 (**EE024_1**)[softcheck upper limit 上限：100,000] [Brackets 700/1500/3500/8000/15000]
2. There was no cost 没有费用

EE025 What was the total cost for hired nurse? 雇佣陪护照顾病人花了多少钱?

1. ____RMB 元 (**EE025_1**)
2. There was no cost 没有费用

EE026 What was the total cost for transportation, food and accommodation of patient and relatives? 自己和家人往返医院交通费、伙食费以及家人或陪护的住宿费一共花了多少钱?

1. ____RMB 元 (**EE026_1**)
2. There was no cost 没有费用

EE027 How much did you or will you eventually pay out of pocket for the total costs of hospitalization? 这次住院，您最后自己总共付了（将要付）多少钱？

1. ____RMB 元 (**EE027_1**) [softcheck upper limit: 100,000 and EE027_1 must be no more than EE024_1 上限：100,000，而且EE027_1 必须小于等于EE024_1]
2. Didn't pay anything. 没有付任何钱 Skip EE028 请跳过EE028

EE028 Who contributes most for paying the out-of-pocket cost? 自付部分谁给您交得最多？

1. Myself 自己
2. Children 子女

3. Relatives 亲属
4. Government 政府
5. Work unit (incl. village collective) 单位（包括村集体）
6. Loan 借款
7. Donate 捐赠
8. Others 其他 Specify 请注明 (**EE028_1**)

EE029 What was the total medication cost during this visit? 这次住院药费一共花了多少钱？

1. ____RMB 元 (**EE029_1**) [softcheck upper limit: 60,000 and EE029_1 should be no more than EE024_1 上限：60,000，而且EE029_1 应该小于等于EE024_1。]
2. Didn't receive 没有用药 Skip EE030 请跳过EE030
   [Brackets 200/500/1800/4000/8000]

EE030 How much did you pay out of pocket for medication costs during this visit? 这次住院，您自己一共付了多少钱的药费？

1. ____RMB 元 (**EE030_1**) [softcheck upper limit: 60,000 and EE030_1 should be no more than EE029_1 and EE024_1 上限：60,000，而且EE030_1 应该小于等于EE029_1 和EE024_1] [Brackets 100/300/1000/2500/5000]
2. Didn't pay anything. 没有付任何钱

[Show Card 出示卡片 14]

EE031 (Preload from EA001 or EB002) What insurance did you use or will you use? (circle all that apply) 您使用或将使用哪种医疗保险报销？（可多选）

1. Urban employee medical insurance (yi-bao) 城镇职工医疗保险（医保）
2. Urban resident medical insurance 城镇居民医疗保险
3. New cooperative medical insurance (he-zuo-yi-liao) 新型农村合作医疗保险（合作医疗）
4. Urban and rural resident medical insurance 城乡居民医疗保险（合并城镇居民和新型农村合作医疗保险）
5. Government medical insurance (gong-fei) 公费医疗
6. Medical aid 医疗救助
7. Private medical insurance: purchased by work unit 商业医疗保险：单位购买
8. Private medical insurance: purchased by individual 商业医疗保险：个人购买
9. Urban non-employed persons's health insurance 城镇无业居民大病医疗保险
10. Other medical insurance (specify) 其他医疗保险，请注明 ____
11. Reimbursed by work unit (incl. village collective) 单位报销
12. No insurance 没有保险
13. Not applicable 不适用

F1：（1）"城镇职工医疗保险"指依法对城镇职工的基本医疗权利给予保障的社会医疗保险制度。（2）"城镇居民医疗保险"指 2007 年 7 月国务院决定在全国 79 个城市实行城镇居民基本医疗保险试点。参保范围：城镇非从业居民，包括：不属于城镇职工基本医疗保险制度覆盖范围的中小学阶段的学

生（包括职业高中、中专、技校学生）、少年儿童和其他非从业城镇居民。
(3)“新型农村合作医疗保险“指由政府组织、引导、支持，农民自愿参加，个人、集体和政府多方筹资，以大病统筹为主的农民医疗互助共济制度。采取个人缴费、集体扶持和政府资助的方式筹集资金。
(4)“城乡居民医疗保险”指一些地区率先将城镇居民医疗保险与新型农村合作医疗合并，推行统一的城乡居民医疗保险制度。
(5)“外来务工人员医疗保险”指在某些地区在城镇职工基本医疗保险以外，专为外来务工人员设置的一种医疗保险制度。
(6)“公费医疗”指国家为保障国家工作人员身体健康而实行的一项社会保障制度。其享受对象主要是各级国家机关、党派、团体以及文化、教育、科研、卫生、体育等事业单位的工作人员和离退休人员，二等乙级以上革命伤残军人以及在校大学生等。
(7)“医疗救助”指在政府的支持下，依靠社会力量建立的旨在向困难群体提供某些或全部基本医疗服务的制度。
(8)“商业医疗保险”是相对于社会保险而言的。商业医疗保险由商业保险公司经办，以营利为目的，企业或职工自愿参加。
(9)“其他医疗保险”如中国职工保险互助会《在职职工重大疾病互助保障计划》和《生育保险》。
(10)“医疗救助”在部分区县试点，救助标准是由各区县根据自身经济情况制定，导致救助标准有高有低。主要三种，一是提供社会医疗救助金，给救助对象以经济补偿；二是给医疗机构一定的经济补贴，使后者直接减免救助对象的部分医疗费；三是由社会医疗救助机构举办专门医疗机构，免费为救助对象提供医疗服务。
(11)“医疗保险”是指基本医疗保险，是为补偿疾病所带来的医疗费用的一种保险。职工因疾病、负伤、生育时，由社会或企业提供必要的医疗服务或物质帮助的社会保险。如中国的公费医疗、劳保医疗。
(12)“单位报销”指一些福利较好的单位可以对本单位职工发生的除基本医疗保险报销部分以外的医疗费用进行全额或一定额度的报销。
(13)城镇无业居民大病医疗保险参保范围是具有本市非农业户籍，男满 16 周岁不满 60 周岁，女满 16 周岁不满 50 周岁，未纳入城镇职工基本医疗保险覆盖范围的居民。筹资标准为每人每年 700 元，其中个人缴纳 600 元、财政补助 100 元。

EE032 Did you pay any “Red Envelopes” to the doctors for this visit? 这次住院期间有没有给医生送红包？

1. Yes 是
2. No 否

Now we'd like to know whether you have treated yourself during the past month. 下面我们想了解在过去一个月，您进行自我治疗的情况。
[Show Card 出示卡片 19]

EF001 Did you use any of the following self-treatment methods during the past month? (circle all that apply) 过去一个月，你是否进行了以下自我治疗（注意：此处不包括凭处方取药的情况）？（可多选）[check, if choose 7 cannot choose other options 如果选择 7，就不能选其他选项。]

1. Purchased over-the-counter modern medicines 自己买非处方西药

2. Purchased prescription medicine 自己买处方西药
3. Traditional herbs or traditional medicine 用传统中草药或者传统方法治疗
4. Vitamins/health supplements 吃维生素/补品/保健品
5. Health care equipment 使用保健设备
6. Other 其他
7. None 没有 Skip to EH001 跳至EH001

**PROCEDURE 程序**：For each circled self-treatment method (1-6), ask questions EF002, EF003, EF005. 对每一种选择的 1-6 的自我治疗方式，均回答EF002，EF003，EF005。

EF002 What is the approximate total cost for [preload EF001] during the last month? 过去一个月，自我治疗 [preload EF001] 的总成本大概是多少？

1. ____RMB 元 (**EF002_1**) [softcheck upper limits: 2,000 上限检查：2,000] [Brackets 10/30/100/200/300]
2. There was no cost. 没有费用。

**PROCEDURE 程序**：IF EF002 = 1 ASK EF003. 如果EF002 = 1，请回答EF003。

EF003 How much did you pay out-of-pocket? 除了报销的部分，您自己支付了多少？

1. ____RMB 元 (**EF003_1**) [softchecks upper limits: 2,000 and EF003_1 should be no more than EF002_1. 上限检查：2,000，而且EF003_1 应该小于等于EF002_1] [Brackets 10/30/100/200/300]
2. Didn't pay anything. 没有付任何钱 Go to EF005 请跳至EF005。

EF004 Who contributed most for paying the out-of-pocket cost? 自付部分谁给您交得最多？

1. Myself 自己
2. Children 子女
3. Relatives 亲属
4. Government 政府
5. Work unit (incl. village collective) 单位（包括村集体）
6. Loan 借款
7. Donation 捐赠
8. Other 其他 Specify 请注明 (**EF004_1**)

[Show Card 出示卡片 14]

EF005 What insurance did you use? (circle all that apply) 您使用了哪种医疗保险报销？（可多选）

1. Urban employee medical insurance (yi-bao) 城镇职工医疗保险（医保）
2. Urban resident medical insurance 城镇居民医疗保险
3. New cooperative medical insurance (he-zuo-yi-liao) 新型农村合作医疗保险（合作医疗）
4. Urban and rural resident medical insurance 城乡居民医疗保险（合并城镇居民和新型农村合作医疗保险）

5. Government medical insurance (gong-fei) 公费医疗
6. Medical aid 医疗救助
7. Private medical insurance: purchased by work unit 商业医疗保险：单位购买
8. Private medical insurance: purchased by individual 商业医疗保险：个人购买
9. Urban non-employed persons's health insurance 城镇无业居民大病医疗保险
10. Other medical insurance (please specify) 其他医疗保险，请注明 ____
11. Reimbursed by work unit 单位报销
12. No insurance 没有保险
13. Not revelent to me 不适用

F1：（1）“城镇职工医疗保险”指依法对城镇职工的基本医疗权利给予保障的社会医疗保险制度。
(2)“城镇居民医疗保险”指 2007 年 7 月国务院决定在全国 79 个城市实行城镇居民基本医疗保险试点。参保范围：城镇非从业居民，包括：不属于城镇职工基本医疗保险制度覆盖范围的中小学阶段的学生（包括职业高中、中专、技校学生）、少年儿童和其他非从业城镇居民。
(3)“新型农村合作医疗保险“指由政府组织、引导、支持，农民自愿参加，个人、集体和政府多方筹资，以大病统筹为主的农民医疗互助共济制度。采取个人缴费、集体扶持和政府资助的方式筹集资金。
(4)“城乡居民医疗保险”指一些地区率先将城镇居民医疗保险与新型农村合作医疗合并，推行统一的城乡居民医疗保险制度。
(5)“外来务工人员医疗保险”指在某些地区在城镇职工基本医疗保险以外，专为外来务工人员设置的一种医疗保险制度。
(6)“公费医疗”指国家为保障国家工作人员身体健康而实行的一项社会保障制度。其享受对象主要是各级国家机关、党派、团体以及文化、教育、科研、卫生、体育等事业单位的工作人员和离退休人员，二等乙级以上革命伤残军人以及在校大学生等。
(7)“医疗救助”指在政府的支持下，依靠社会力量建立的旨在向困难群体提供某些或全部基本医疗服务的制度。
(8)“商业医疗保险”是相对于社会保险而言的。商业医疗保险由商业保险公司经办，以营利为目的，企业或职工自愿参加。
(9)“其他医疗保险”如中国职工保险互助会《在职职工重大疾病互助保障计划》和《生育保险》。
(10)“医疗救助”在部分区县试点，救助标准是由各区县根据自身经济情况制定，导致救助标准有高有低。主要三种，一是提供社会医疗救助金，给救助对象以经济补偿；二是给医疗机构一定的经济补贴，使后者直接减免救助对象的部分医疗费；三是由社会医疗救助机构举办专门医疗机构，免费为救助对象提供医疗服务。
(11)“医疗保险”是指基本医疗保险，是为补偿疾病所带来的医疗费用的一种保险。职工因疾病、负伤、生育时，由社会或企业提供必要的医疗服务或物质帮助的社会保险。如中国的公费医疗、劳保医疗。
(12)“单位报销”指一些福利较好的单位可以对本单位职工发生的除基本医疗保险报销部分以外的医疗费用进行全额或一定额度的报销。
(13)城镇无业居民大病医疗保险参保范围是具有本市非农业户籍，男满 16 周岁不满 60 周岁，女满 16 周岁不满 50 周岁，未纳入城镇职工基本医疗保险覆盖范围的居民。筹资标准为每人每年 700 元，其中个人缴纳 600 元、财政补助 100 元。

The following questions pertain to dental care that you have had during the past year. 下面我们想了解一下您过去一年看牙医的情况。

EH001 In the past year, have you seen a dentist for dental care, including dentures? 过去一年，您是否看过牙医？

1. Yes 有
2. No 没有 skip this part 跳过整个看牙部分

EH002 How many times have you received dental care during the past year? 过去一年，您看过几次牙医？ ____Times 次

EH003 What was the medical cost for all the dental care in the past year? 过去一年，看牙的总费用大概是多少?

1. Total cost 总费用 ____Yuan 元;
2. Didn't pay anything. 没有付任何钱。[softcheck upper limit 上限：30,000]

**PROCEDURE 程序**：IF EH003 = 1 ASK EH004

EH004

1. Self-paid part 其中自付部分 _______(**EH004_1**)Yuan 元
2. Didn't pay anything 没有付任何钱

**PROCEDURE 程序**：IF EH004 = "Didn't pay anything", skip EH005

EH005 Who contribute most for paying the out-of-pocket cost? 自付部分谁给您交得最多？

1. Myself 自己
2. Children 子女
3. Relatives 亲属
4. Government 政府
5. Work unit (incl. village collective) 单位（包括村集体）
6. Loan 借款
7. Donate 捐赠
8. Others 其他 Specify 请注明 (**EH005_1**)

[Show Card 出示卡片 14]

EH006 What insurance did you use? (circle all that apply) 您使用了哪种医疗保险报销？(可多选)

1. Urban employee medical insurance (yi-bao) 城镇职工医疗保险（医保）
2. Urban resident medical insurance 城镇居民医疗保险
3. New cooperative medical insurance (he-zuo-yi-liao) 新型农村合作医疗保险（合作医疗）
4. Urban and rural resident medical insurance 城乡居民医疗保险（合并城镇居民和新型农村合作医疗保险）
5. Government medical insurance (gong-fei) 公费医疗
6. Medical aid 医疗救助

7. Private medical insurance: purchased by work unit 商业医疗保险：单位购买
8. Private medical insurance: purchased by individual 商业医疗保险：个人购买
9. Urban non-employed persons's health insurance 城镇无业居民大病医疗保险
10. Other medical insurance (specify) 其他医疗保险，请注明 ____(**EH006_1**)
11. Reimbursed by work unit 单位报销
12. No insurance 没有保险
13. Not revelent to me. 不适用

EH007_W3 Are you satisfied with the quality, cost, and convenience of local health care services? 您对本地医疗服务的质量、成本和方便程度满意吗？

1. Very satisfied 非常满意
2. Somewhat satisfied 比较满意
3. Neutral 一般
4. Somewhat dissatisfied 比较不满意
5. Very dissatisfied 一点也不满意

F1：(1)“城镇职工医疗保险”指依法对城镇职工的基本医疗权利给予保障的社会医疗保险制度。
(2)“城镇居民医疗保险”指 2007 年 7 月国务院决定在全国 79 个城市实行城镇居民基本医疗保险试点。参保范围：城镇非从业居民，包括：不属于城镇职工基本医疗保险制度覆盖范围的中小学阶段的学生（包括职业高中、中专、技校学生）、少年儿童和其他非从业城镇居民。
(3)“新型农村合作医疗保险“指由政府组织、引导、支持，农民自愿参加，个人、集体和政府多方筹资，以大病统筹为主的农民医疗互助共济制度。采取个人缴费、集体扶持和政府资助的方式筹集资金。
(4)“城乡居民医疗保险”指一些地区率先将城镇居民医疗保险与新型农村合作医疗合并，推行统一的城乡居民医疗保险制度。
(5)“外来务工人员医疗保险”指在某些地区在城镇职工基本医疗保险以外，专为外来务工人员设置的一种医疗保险制度。
(6)“公费医疗”指国家为保障国家工作人员身体健康而实行的一项社会保障制度。其享受对象主要是各级国家机关、党派、团体以及文化、教育、科研、卫生、体育等事业单位的工作人员和离退休人员，二等乙级以上革命伤残军人以及在校大学生等。
(7)“医疗救助”指在政府的支持下，依靠社会力量建立的旨在向困难群体提供某些或全部基本医疗服务的制度。
(8)“商业医疗保险”是相对于社会保险而言的。商业医疗保险由商业保险公司经办，以营利为目的，企业或职工自愿参加。
(9)“其他医疗保险”如中国职工保险互助会《在职职工重大疾病互助保障计划》和《生育保险》。
(10)“医疗救助”在部分区县试点，救助标准是由各区县根据自身经济情况制定，导致救助标准有高有低。主要三种，一是提供社会医疗救助金，给救助对象以经济补偿；二是给医疗机构一定的经济补贴，使后者直接减免救助对象的部分医疗费；三是由社会医疗救助机构举办专门医疗机构，免费为救助对象提供医疗服务。
(11)“医疗保险”是指基本医疗保险，是为补偿疾病所带来的医疗费用的一种保险。职工因疾病、负伤、生育时，由社会或企业提供必要的医疗服务或物质帮助的社会保险。如中国的公费医疗、劳保医疗。
(12)“单位报销”指一些福利较好的单位可以对本单位职工发生的除基本医疗保险报销部分以外的医疗费用进行全额或一定额度的报销。

（13）城镇无业居民大病医疗保险参保范围是具有本市非农业户籍，男满 16 周岁不满 60 周岁，女满 16 周岁不满 50 周岁，未纳入城镇职工基本医疗保险覆盖范围的居民。筹资标准为每人每年 700 元，其中个人缴纳 600 元、财政补助 100 元。

EF006 How often did the respondent receive assistance in answering section D-Health care and insurance? 受访者填写该部分问卷时是否求助？[IWER: If it is answered by a proxy, the respondent's reaction. 访员注意：如果是协助回答，请记录受访者的反应。]

1. Never 从未
2. A few times 偶尔几次
3. Most or all of the time 大多数
4. The section was completed by a proxy respondent（the respondent is absent）受访者不在场，完全请人代填 → Skip to EF007 请跳至EF007

EF007 What is your relationship to R? 您和受访者是什么关系？[IWER: What is the proxy's relationship to R? If unknown, please ask the proxy. 访员注意：代填问卷的人和受访者是什么关系。如果不清楚，请问代填者。]

1. Spouse 配偶
2. Mother 母亲
3. Father 父亲
4. Mother-in-law 岳母/婆婆
5. Father-in-law 岳父/公公
6. Sibling 兄弟姐妹
7. Brother-in-law, sister-in-law 姐夫妹夫/嫂子弟媳
8. Child 孩子
9. Spouse of child 孩子的配偶
10. Grandchild 孙子女
11. Other relative 其他亲戚
12. Helper or other non-relative 帮忙的人或者其他非亲属

EF008 [IWER: Please record the reason for proxy 访员注意：请记录代答的原因] What is the main reason for proxy（the respondent is absent）？受访者不在场，完全请人代填的主要原因是什么？

1. The respondent has serious physical handicaps 受访者有严重身体障碍
2. The respondent has serious mental handicaps, 受访者有严重精神障碍
3. The respondent has rejected this interview 受访者拒访
4. Other 其他请注明 ____(**EF008_1**)

F WORK, RETIREMENT AND PENSION 工作、退休和养老金

Type of Interview R 受访者类型		
XRType = REIW	This is a reinterview R	受访者类型为回访受访者
XRType = NEWIW	This is a new interview R	受访者类型为新受访者

R's LAST IW Time (ZIWTime) 上次访问的日期 (ZIWTime)

PRELOAD VARIABLE ACCORDING TO LAST WAVE INTERVIEW: 根据上一轮调查形成的加载变量

**ZF1**= 1	Old R was only doing agricultural work in the last IW<br>老受访者上一轮调查时只从事农业工作
**ZF1**= 2	Old R was only doing non-agricultural work in the last IW<br>老受访者上一轮调查时只从事非农工作
**ZF1**= 3	Old R was doing both agricultural and non-agricultural work in the last IW<br>老受访者上一轮调查时既从事农业工作又从事非农工作
**ZF1**= 4	Old R was not working in the last IW<br>老受访者上一轮调查时不工作
**ZF5**= 1	Old R was doing employed work in the last IW<br>老受访者上一轮调查时从事受雇工作
**ZF6**= 1	Old R was doing self-employed work in the last IW<br>老受访者上一轮调查时从事自雇工作
**ZF7**= 1	Old R was doing unpaid family business in the last IW<br>老受访者上一轮调查时无偿为家庭经营活动帮工
**ZF8**	The name of old R's employer in the last IW<br>老受访者上一轮调查时的雇主名称
**ZF9**	The name of old R's company or business in the last IW<br>老受访者上一轮调查时公司或生意的名称
**ZF10**	The name of company or workplace old R worked without wage in the last IW<br>老受访者上一轮调查时不拿工资帮工的公司或生意的名称
**ZF13**= 1	Old R was working in the last IW<br>老受访者上一轮调查时有工作
**ZF14**= 1	Old R had not worked prior to the last IW<br>老受访者至上一轮调查时从未工作过
**ZF14**= 2	Old R had worked prior to the last IW<br>老受访者至上一轮调查时曾经工作过
**ZF15**= 1	Old R had completed retirement procedure (including early retirement) or internal retirement in the last IW. 老受访者上一轮调查时已经办理了退休（包括提前退休）或内退
**ZF16**= 1	Old R had completed receding position procedure in the last IW<br>老受访者上一轮调查时已经办理了退职

ZF18 Old R's position in employed work in the last IW
老受访者上一轮调查时从事的受雇工作的职务

ZF22_1= 1 Old R's last job is not missing, FL001, FL004 and FL005 are not missing
老受访者的最近一份工作没有缺失（FL001、FL004 和 FL005 没有缺失）

ZF22_1= 2 Old R's last job is missing, FL001-FL022_bracket are all missing
老受访者的最近一份工作缺失（FL001、FL004 和 FL005 至少有一个缺失）

ZF23= 1 Old R had completed internal retirement but not yet regular retirement prior to the last IW. 老受访者上一轮调查时已经办理内退但是还没有办理正式退休

ZF24= 1 Old R had not completed retirment procedure or receding position procedure prior to the last IW. 老受访者上一轮调查时还没有办理退休或退职

ZF25_1= 1 Old R had completed retirment or receding position procedure in the last IW, but the work unit is missing.
上一轮调查时，老受访者办理了退休/退职，但是办理的单位地址缺失

ZF25_2= 1 Old R had been receding position procedure in the last IW, but the time is missing.
上一轮调查时，老受访者办理了退职，但是办理的时间缺失

ZF25_3= 1 Old R had been receding position procedure in the last IW, but the wage is missing.
上一轮调查时，老受访者办理了退职，但是退职前月工资缺失

ZF25_4= 1 Old R had completed retirement or early retirement.
上一轮调查时，老受访者办理了 [正式/提前] 退休

ZF25_5= 1 Old R had completed retirement or early retirement, but the time is missing.
上一轮调查时，老受访者办理了 [正式/提前] 退休，但是办理的时间缺失

ZF25_6= 1 Old R had completed retirement or early retirement, but the wage is missing.
上一轮调查时，老受访者办理了 [正式/提前] 退休，但是退休前月工资缺失

ZF25_8= 1 Old R had completed internal retirement.
上一轮调查时，老受访者办理了内退

ZF25_9= 1 Old R had completed internal retirement, but the time is missing.
上一轮调查时，老受访者办理了内退，但是办理的时间缺失

ZF25_10= 1 Old R had completed internal retirement, but the wage prior internal retirement is missing.
上一轮调查时，老受访者办理了内退，但是内退前的工资缺失

ZF25_11= 1 Old R had completed internal retirement, but the wage at internal retirement is missing.
上一轮调查时，老受访者办理了内退，但是内退时的工资缺失

ZF26= 1 Old R had competed regular retirment in the last IW
老受访者上一轮调查时已经办理正式退休

ZF27= 1 Old R had not worked after processing retirement procedure or receding position procedure by the last IW. 上一轮调查时，老受访者办理退休/退职后没有工作过

XF1= 1 Old R is working in the current IW
老受访者本轮调查时在工作

XF1= 2 Old R is not working in the current IW
老受访者本轮调查时没有工作

XF2= 1 R (including new R and old R) did not do faming last year but did non-agricultural

work last week. 受访者（包括新受访者和老受访者）去年没有从事过农业工作但是上周从事过非农工作

FA JOB STATUS 工作情况

FA001 Did you engage in agricultural work (including farming, forestry, fishing, and husbandry for your own family or others) for more than 10 days in the past year? 过去一年，您有没有从事 10 天以上的农业生产经营活动（包括为自家或其他农户种地、管理果树、采集农林产品、养鱼、打鱼、养牲畜以及去市场销售自家生产的农产品等）?

1. Yes 是 → NEWIW skips to FC001; REIW skips to FA006_w2_1 BRANCHPOINT 新受访者跳至 FC001；回访受访者跳至FA006_w2_1 BRANCHPOINT
2. No 否

FA002 Did you work for at least one hour last week? We consider any of the following activities to be work: earn a wage, run your own business and unpaid family business work, et. al. Work does not include doing your own housework or doing activities without pay, such as voluntary work. 上周您工作了至少一个小时吗？挣工资工作、从事个体、私营经济活动或不拿工资为家庭经营活动帮工都算是工作，但不包括家务劳动、义务的志愿劳动。

1. Yes 是 → NEWIW skips to FC019; REIW skips to FA006_w2_1 BRANCHPOINT 新受访者跳至 FC019; 回访受访者跳至FA006_w2_1 BRANCHPOINT
2. No 否

FA003 Do you have a job but are temporarily laid-off, or on sick or other leave, or in-job training? 您是不是有工作但是目前正处在临时放假、休病假，或其他假期中，或者正在在职培训?

1. Yes 是
2. No 否 → NEWIW skips to FA007; REIW skips to FA006_w2_1 BRANCHPOINT 新受访者跳至FA007; 回访受访者跳至FA006_w2_1 BRANCHPOINT

FA004 In what month and year did you leave or attend training? 您是什么时候开始休假或参加培训的?

_____ 1900...2015 (**FA004_1**) year 年 _____ 0...12 (**FA004_2**) month 月

[IWER: Mark the year using four digits. Take down the month as its actual number. For example, write January as “1” not “01”, December as “12”. If do not remember month, fill “0” 访员注意：用 4 位数表示年，按照实际的月份填写月。例：1 月写作“1”，而不是“01”,12 月写作“12”。如果记不住月份，请填入“0”]

FA005 Do you expect to go back to this job at a definite time in the future or within 6 months? 您能够在确定的时间或者 6 个月以内，回到原来的工作岗位么?

1. Yes 是 → NEWIW skips to FC019; REIW skips to FA006_w2_1 BRANCHPOINT 新受访者跳至FC019; 回访受访者跳至FA006_w2_1 BRANCHPOINT

2. No 否

FA006 Do you still receive any salary from this job? 这个单位是否仍然给你发工资?

1. Yes 是 → NEWIW skips to FC019; REIW skips to FA006_w2_1 BRANCHPOINT 新受访者跳至FC019; 回访受访者跳至FA006_w2_1 BRANCHPOINT
2. No 否 → NEWIW skips to FA007; REIW skips to FA006_w2_1 BRANCHPOINT 新受访者跳至FA007; 回访受访者跳至FA006_w2_1 BRANCHPOINT

FA006_w2_1 BRANCHPOINT:

For REIW doing agricultuarl work (FA001= 1): If he/she was only doing agricultural work in the last IW (ZF1= 1), SKIP TO FA006_w3_1; If he/she was only doing non-agricultural work (ZF1= 2) or not doing any work in the last IW (ZF1= 4), SKIP TO FA006_w2_2; If he/she was doing both agricultural and non-agricultural work in the last IW(ZF1= 3), skip to FA006_w2_2a Branchpoint.

本次调查从事农业工作 (FA001= 1) 的回访受访者：如果上一轮调查时只从事农业工作 (ZF1= 1)，跳至FA006_w3_1；如果上一轮调查时只从事非农业工作 (ZF1= 2) 或者不从事任何工作 (ZF1= 4)，跳至FA006_w2_2；如果上一轮调查时既从事农业工作又从事非农工作 (ZF1= 3)，跳至FA006_w2_2a Branchpoint

For REIW not doing agricultural work but doing non-agricultural work (FA001= 2 & FA002= 1): If he/she was only doing agricultural work in the last IW (ZF1= 1) or doing both agricultural and non-agricultural work (ZF1 = 3) in the last IW, skip to FA006_w2_1; If he/she was only doing non-agricultural work in the last IW (ZF1= 2), skip to FA006_w2_2a Branchpoint; If he/she was not doing any work in the last IW (ZF1= 4), SKIP TO FA006_w3_1;

本次调查没有从事农业工作但是从事非农工作 (FA001= 2 & FA002= 1) 的回访受访者：如果上一轮调查时只从事农业工作 (ZF1= 1) 或者既从事农业工作又从事非农工作 (ZF1 = 3)，跳至FA006_w2_1；如果上一轮调查时只从事非农工作 (ZF1= 2)，跳至 FA006_w2_2a Branchpoint；如果上一轮调查时不从事任何工作 (ZF1= 4)，跳至 FA006_w3_1

For REIW not doing any work(XF1= 2): If he/she was only doing agricultural work in the last IW (ZF1= 1), or doing both agricultural and non-agricultural work in the last IW (ZF1= 3), skip to FA006_w2_1; If he/she was only doing non-agricultural work in the last IW (ZF1= 2), skip to FA006_w2_2a Branchpoint; If he/she was not doing any work in the last IW (ZF1= 4), skip to FA006_w3_1.

本次调查时不从事任何工作 (XF1= 2) 的回访受访者：如果上一轮调查时只从事农业工作 (ZF1= 1) 或者既从事农业工作又从事非农工作 (ZF1 = 3)，跳至 FA006_w2_1；如果上一轮调查时只从事非农工作 (ZF1= 2)，跳至 FA006_w2_2a Branchpoint；如果上一轮调查时不从事任何工作 (ZF1= 4)，跳至 FA006_w3_1

FA006_w2_1 You told us that you were doing agricultural work (ZF1= 1 or ZF1= 3) in the last IW, when did you stop doing it? 上一轮调查时 [ZIWTime] 您告诉我们您从事农业工作（包括为自家或其他农户种地、管理果树、采集农林产品、养鱼、打鱼、养牲畜以及去市场销售自家生产的农产品等）（ZF1= 1 或 ZF1= 3），您是什么时候停止农业工作的？

1. _____ (**FA006_w2_1_1**) year 年 _____ (**FA006_w2_1_2**) month 月

2. I was not doing agricultural work in the last IW 上一轮时我没有从事农业工作 → Skip to FA006_w2_1a Branchpoint 跳至FA006_w2_1a Branchpoint

FA006_w2_1a What was the reason for you to stop doing this job? 您停止做这份工作的原因是什么?

1. Business closed 单位破产/倒闭了
2. Quit 辞职
3. I was laid off 下岗
4. I was fired 被辞退
5. I went to school 我上学了
6. I went abroad 我出国了
7. I stopped working for health reasons 因为健康原因中断工作
8. I stopped working for family reasons 因为家庭原因中断工作
9. I was transferred to another job 工作调动
10. I started working off-farm locally 我在本地从事非农工作了
11. I went to work away from home 我外出打工了
12. Better job in local area 本地更好的新工作
13. Better job in another location 外地更好的新工作
14. I retired 我退休了
15. Other 其他

FA006_w2_1a BRANCHPOINT:

IF XRType = REIW AND HE/SHE was only doing agricultural work (ZF1= 1) IN THE LAST WAVE, THEN SKIP TO FA006_w3_3 BRANCHPOINT.

如果上一轮调查时只从事农业工作的回访受访者 (ZF1= 1), 跳至 FA006_w3_3 BRANCHPOINT

IF XRType = REIW AND HE/SHE was doing both agricultural and non-agricultural work (ZF1= 3) IN THE LAST WAVE, THEN SKIP TO FA006_w2_2a Branchpoint.

如果上一轮调查时既从事农业工作又从事非农工作的回访受访者 (ZF1= 3), 跳至 FA006_w2_2a Branchpoint

FA006_w2_2 You told us that you were not doing agricultural work in the last IW (ZF1= 2 or ZF1= 4), when did you starting doing agricultural work? 上一轮时 [ZIWTime] 您告诉我们您没有从事农业工作（ZF1= 2 或 ZF1= 4），您是什么时候开始从事农业工作的?

1. _____ (**FA006_w2_2_1**) year 年 _____ (**FA006_w2_2_2**) month 月
2. I was doing agriclutural work in the last IW 上一轮时我就从事农业工作 Skip to FA006_w3_3 跳至 FA006_w3_3

PROCEDURE 程序:

If XRType = REIW and he/she was only doing non-agricultural work in the last IW (ZF1= 2), skip to FA006_w2_2a Branchpoint;

如果上一轮调查时只从事非农工作的回访受访者 (ZF1= 2), 跳至 FA006_w2_2a Branchpoint

If XRType = REIW and he/she was not doing any work in the last IW (ZF1= 4), skip to FA006_w3_1;

如果上一轮调查时不从事任何工作的回访受访者 (ZF1= 4), 跳至 FA006_w3_1

FA006_w2_2a BRANCHPOINT:

For old R who was doing employed work in the last IW (ZF5= 1): If the name of the employer is not missing in the last IW (ZF8≠ .), skip to FA006_w2_2a; If the name of the employer is missing in the last IW (ZF8= .), skip to FA006_w2_2a_2.

上一轮时从事受雇工作的回访受访者 (ZF5= 1)：如果上一轮调查时雇主名称没有缺失 (ZF8 ≠ .)，跳至FA006_w2_2a；如果上一轮调查时雇主名称缺失 (ZF8= .)，跳至 FA006_w2_2a_2

For old R who was doing self-employed work in the last IW (ZF6= 1): If the name of the company/business is not missing in the last IW (ZF9≠ .), skip to FA006_w2_2b; If the name of the company/business is missing in the last IW (ZF9= .), skip to FA006_w2_2b_2.

上一轮从事自雇工作的回访受访者 (ZF6= 1)：如果上一轮调查时公司/生意名称没有缺失 (ZF9≠ .)，跳至 FA006_w2_2b；如果上一轮调查时公司/生意名称缺失 (ZF9= .)，跳至 FA006_w2_2b_2

For old R who was working for unpaid family business (ZF7= 1): If the name of the family business is not missing in the last IW (ZF10≠ .), skip to FA006_w2_2c. If the name of the family business is missing in the last IW (ZF10= .), skip to FA006_w2_2c_2.

上一轮不拿工资为家庭经营活动帮工的回访受访者 (ZF7= 1)：如果上一轮调查时家庭经营活动没有缺失 (ZF10≠ .)，跳至 FA006_w2_2c；如果上一轮调查时家庭经营活动缺失 (ZF10= .)，跳至 FA006_w2_2c_2

FA006_w2_2a You told us that you were working for [Preload the name of the employer from the last IW(ZF8)] in the last IW, is the name correct? 您告诉我们上一轮调查时 [ZIWTime] 您为 [下载上一轮调查时的雇主名称（ZF8）] 工作，这个名称正确吗？

1. Yes 是 → Skip to FA006_w2_2a_3 跳至FA006_w2_2a_3
2. No 否

[访员注意：如果受访者回答说上一轮不从事受雇（为别人）工作，请选择"(2) 否"]

FA006_w2_2a_1 What is the correct name? 请问正确的名称是？

[IWER: Write the name of the household head if R works for a family 访员注意：如果雇主是家庭，工作单位请写户主的姓名]

1. Fill in the employer's name 填写雇主名称 _____ (**FA006_w2_2a_1_1**)（例 1：北京大学餐饮服务中心；例 2：北京华信服装服饰有限责任公司）Name of the department 所在部门名称：_____ （例 1：农园餐厅；例 2：纺纱生产车间）→ Skip to FA006_w2_2a_3 跳至FA006_w2_2a_3
2. Last IW, you ran your own business, fill in the firm's name 上一轮不从事受雇，而是自己开公司/做生意，请填写公司/生意名称 _____ (**FA006_w2_2a_1_2**)（例 1：北京华信服装服饰有限责任公司；例 2：丽丽服装店）→ Skip to FA006_w3_1 跳至 FA006_w3_1
3. Last IW, you were not employed and did work for family business without being paid, fill in the firm's name 上一轮不从事受雇，而是为家庭经营活动无偿帮工，请填写帮工的公司/生意名称 _____ (**FA006_w2_2a_1_3**)（例 1：北京华信服装服饰有限责任公司；例 2：丽丽服装店）→ Skip to FA006_w3_1 跳至 FA006_w3_1
4. Last IW, you were not employed and did farm work 上一轮不从事受雇，而是务农 → Skip to FA006_w3_1 跳至 FA006_w3_1

5. Last IW, you did not work 上一轮不工作 → Skip to FA006_w3_1 跳至 FA006_w3_1

FA006_w2_2a_2 You told us that you were working for someone else in the last IW, but you did not tell us the name of the employer in the last IW, what is the name of the employer you were working for in the last IW? 上一轮调查时 [ZIWTime] 您告诉我们您为其他人工作，但是没有告诉我们雇主的名称，请问您当时的雇主名称是？

[IWER: Write the name of the household head if R works for a family 访员注意：如果雇主是家庭，工作单位请写户主的姓名]

1. Fill in the employer's name 填写雇主名称 _____ (**FA006_w2_2a_2_1**)（例 1：北京大学餐饮服务中心；例 2：北京华信服装服饰有限责任公司）Name of the department 所在部门名称：_____ （例 1：农园餐厅；例 2：纺纱生产车间）
2. Last IW, you ran your own business, fill in the firm's name 上一轮不从事受雇，而是自己开公司/做生意，请填写公司/生意名称 _____ (**FA006_w2_2a_2_2**)（例 1：北京华信服装服饰有限责任公司；例 2：丽丽服装店）→ Skip to FA006_w3_1
3. Last IW, you were not employed and did work for family business without being paid, fill in the firm's name 上一轮不从事受雇，而是为家庭经营活动无偿帮工，请填写帮工的公司/生意名称 _____ (**FA006_w2_2a_2_3**)（例 1：北京华信服装服饰有限责任公司；例 2：丽丽服装店）→ Skip to FA006_w3_1 跳至 FA006_w3_1
4. Last IW, you were not employed and did farm work 上一轮不从事受雇，而是务农 → Skip to FA006_w3_1 跳至 FA006_w3_1
5. Last IW, you did not work 上一轮不工作 → Skip to FA006_w3_1 跳至 FA006_w3_1

FA006_w2_2a_3 Are you still working for this employer? 您还在为这个雇主工作吗？

1. Yes 是 → Skip to FA006_w3_1 跳至 FA006_w3_1
2. No 否 → Skip to FA006_w2_3 跳至FA006_w2_3

FA006_w2_2b You told us that you were running _____ [Preload the name of the company/workplace from the last IW (ZF9), is the name correct? 上一轮调查时 [ZIWTime] 您告诉我们您开/做 [下载上一轮调查时公司/生意的名称（ZF9）] 公司/生意，这个名称正确吗？

1. Yes 是 → Skip to FA006_w2_2b_3 跳至FA006_w2_2b_3
2. No 否

[访员注意：如果受访者回答说上一轮不从事自雇 (自己开公司/做生意) 工作，请选择"(2) 否"]

FA006_w2_2b_1 What is the correct name? 请问正确的名称是？

1. Fill in the firm's name 填写开的公司/做的生意的名称 _____ (**FA006_w2_2b_1_1**)（例 1：北京华信服装服饰有限责任公司；例 2：丽丽服装店）→ Skip to FA006_w2_2b_3 跳至FA006_w2_2b_3
2. Last IW, you were employed, fill in the firm's name 上一轮不从事自雇，而是受雇（挣工资工作），请填写受雇的公司/生意名称 _____ (**FA006_w2_2b_1_2**)（例 1：北京大学餐饮服务中心；例 2：北京华信服装服饰有限责任公司）Name of the department 所在部门名称：_____ （例 1：农园餐厅；例 2：纺纱生产车间）→ Skip to FA006_w3_1 跳至 FA006_w3_1

3. Last IW, you were not self-employed and did work for family business without being paid, fill in the firm' s name 上一轮不从事自雇，而是为家庭经营活动无偿帮工，请填写帮工的公司/生意名称 _____ (**FA006_w2_2b_1_3**) （例 1：北京华信服装服饰有限责任公司；例 2：丽丽服装店）→ Skip to FA006_w3_1 跳至 FA006_w3_1
4. Last IW, you were not self-employed and did farm work 上一轮不从事自雇，而是务农 → Skip to FA006_w3_1 跳至 FA006_w3_1
5. Last IW, you did not work 上一轮不工作 → Skip to FA006_w3_1 跳至 FA006_w3_1

FA006_w2_2b_2 You told us that you were running a company/business in the last IW, but you did not tell us the name of the company/business in the last IW, what is the name of the company/business you were running in the last IW? 上一轮调查时 [ZIWTime] 您告诉我们您在开公司/做生意，但是没有告诉我们公司/生意的名称，请问您当时的公司/生意名称是？

1. Fill in the firm's name 填写开的公司/做的生意的名称 _____ (**FA006_w2_2b_2_1**) (例 1：北京华信服装服饰有限责任公司；例 2：丽丽服装店)
2. Last IW, you were employed, fill in the firm's name 上一轮不从事自雇，而是受雇（挣工资工作），请填写受雇的公司/生意名称 _____ (**FA006_w2_2b_2_2**) （例 1：北京大学餐饮服务中心；例 2：北京华信服装服饰有限责任公司）Name of the department 所在部门名称：_____ （例 1：农园餐厅；例 2：纺纱生产车间）→ Skip to FA006_w3_1 跳至 FA006_w3_1
3. Last IW, you were not self-employed and did work for family business without being paid, fill in the firm's name 上一轮不从事自雇，而是为家庭经营活动无偿帮工，请填写帮工的公司/生意名称 _____ (**FA006_w2_2b_2_3**) （例 1：北京华信服装服饰有限责任公司；例 2：丽丽服装店）→ Skip to FA006_w3_1 跳至FA006_w3_1
4. Last IW, you were not self-employed and did farm work 上一轮不从事自雇，而是务农 → Skip to FA006_w3_1 跳至 FA006_w3_1
5. Last IW, you did not work 上一轮不工作 → Skip to FA006_w3_1 跳至 FA006_w3_1

FA006_w2_2b_3 Are you still running this company/business? 您还在开这个公司/做这项生意吗？

1. Yes 是 → Skip to FA006_w3_1 跳至 FA006_w3_1
2. No 否 → Skip to FA006_w2_3 跳至FA006_w2_3

FA006_w2_2c You told us that you were working for an unpaid family business [Preload the name of the unpaid family business from the last IW (ZF10)], is the name correct? 上一轮调查时 [ZIWTime] 您告诉我们您不拿工资为家庭经营活动 [下载上一轮无偿帮工家庭经营活动的名称（ZF10）] 帮工，这个名称正确吗？

1. Yes 是 → Skip to FA006_w2_2c_3 跳至 FA006_w2_2c_3
2. No 否

[访员注意：如果受访者回答说上一轮不是为家庭经营活动无偿帮工，请选择“(2) 否”]

FA006_w2_2c_1 What is the correct name? 请问正确的名称是？

1. Fill in the firm's name 填写帮工的公司/生意的名称 _____ (**FA006_w2_2c_1_1**)（例 1：北京华信服装服饰有限责任公司；例 2：丽丽服装店）→ Skip to FA006_w2_2c_3 跳至FA006_w2_2c_3
2. Last IW, you were employed, fill in the firm's name 上一轮不从事无偿帮工，而是受雇（挣工资工作），请填写受雇的公司/生意名称 _____ (**FA006_w2_2c_1_2**)（例 1：北京大学餐饮服务中心；例 2：北京华信服装服饰有限责任公司）Name of the department 所在部门名称：_____ （例 1：农园餐厅；例 2：纺纱生产车间）→ Skip to FA006_w3_1 跳至 FA006_w3_1
3. Last IW, you ran your own business, fill in the firm's name 上一轮不从事无偿帮工，而是自己开公司/做生意，请填写公司/生意名称 _____ (**FA006_w2_2c_1_3**)（例 1：北京华信服装服饰有限责任公司；例 2：丽丽服装店）→ Skip to FA006_w3_1 跳至 FA006_w3_1
4. 上一轮不从事无偿帮工，而是务农 → Skip to FA006_w3_1 跳至 FA006_w3_1
5. 上一轮不工作 → Skip to FA006_w3_1 跳至 FA006_w3_1

FA006_w2_2c_2 You told us that you were working for an family business without a wage in the last IW, but you did not tell us the name of the name of the family business, what is the name of this family business you were working for without a wage in the last IW? 上一轮调查时 [ZIWTime] 您告诉我们您不拿工资为一项家庭经营活动帮工，但是没有告诉我们这项家庭经营活动的名称，请问您上一轮调查时不拿工资帮工的家庭经营活动的名称是？

1. Fill in the firm's name 填写帮工的公司/生意的名称 _____ (**FA006_w2_2c_2_1**)（例 1：北京华信服装服饰有限责任公司；例 2：丽丽服装店）
2. Last IW, you were employed, fill in the firm's name 上一轮不从事无偿帮工，而是受雇（挣工资工作），请填写受雇的公司/生意名称 _____ (**FA006_w2_2c_2_2**)（例 1：北京大学餐饮服务中心；例 2：北京华信服装服饰有限责任公司）Name of the department 所在部门名称：_____ （例 1：农园餐厅；例 2：纺纱生产车间）→ Skip to FA006_w3_1 跳至 FA006_w3_1
3. Last IW, you ran your own business, fill in the firm's name 上一轮不从事无偿帮工，而是自己开公司/做生意，请填写公司/生意名称 _____ (**FA006_w2_2c_2_3**)（例 1：北京华信服装服饰有限责任公司；例 2：丽丽服装店）→ Skip to FA006_w3_1 跳至 FA006_w3_1
4. 上一轮不从事无偿帮工，而是务农 → Skip to FA006_w3_1 跳至 FA006_w3_1
5. 上一轮不工作 → Skip to FA006_w3_1 跳至 FA006_w3_1

FA006_w2_2c_3 Are you still working for this family business? 您还在为这项家庭经营活动帮工吗？

1. Yes 是 → Skip to FA006_w3_1 跳至 FA006_w3_1
2. No 否 → Skip to FA006_w2_3 跳至FA006_w2_3

FA006_w2_3 When did you stop doing this job? 您是什么时候停止做这份工作的？_____ (**FA006_w2_3_1**) year 年 _____ (**FA006_w2_3_2**) month 月

FA006_w2_4 What was the reason for you to stop doing this job? 您停止做这份工作的原因是什么？

1. Business closed 单位破产／倒闭了
2. Quit 辞职
3. I was laid off 下岗
4. I was fired 被辞退
5. I went to school 我上学了
6. I went abroad 我出国了
7. I stopped working for health reasons 因为健康原因中断工作
8. I stopped working for family reasons 因为家庭原因中断工作
9. I was transferred to another job 工作调动
10. I started working off-farm locally 我在本地从事非农工作了
11. I went to work away from home 我外出打工了
12. Better job in local area 本地更好的新工作
13. Better job in another location 外地更好的新工作
14. I retired 我退休了
15. Other 其他

FA006_w3_1 Did you engage in agricultural work (including farming, forestry, fishing, and husbandry for your own family or others) in the last IW? 上一次访问时 [ZIWTime]，您有没有从事农业生产经营活动（包括为自家或其他农户种地、管理果树、采集农林产品、养鱼、打鱼、养牲畜以及去市场销售自家生产的农产品等）？

1. Yes 有
2. No 没有 → Skip to FA006_w3_3 跳至FA006_w3_3

FA006_w3_2 What agricultural work did you engage in the last IW? 您当时从事的农业生产经营活动是？（可多选）

1. Household agricultural work 自家农业生产活动
2. Farm employed 为其他农户／农场打工

FA006_w3_3 BRANCHPOINT:

如果上一轮调查时受访者从事非农业工作 (**ZF1**= 2 or **ZF1**= 3)，跳过 FA006_w3_3—FA006_w3_7

如果上一轮调查时受访者没有从事非农工作 (**ZF1**= 1)，询问 FA006_w3_3

如果上一轮调查时受访者没有工作 (**ZF1**= 4)，询问 FA006_w3_3

FA006_w3_3 Did you work in the last IW? We consider any of the following activities to be work: earn a wage, run your own business and unpaid family business work, et. al. Work does not include doing your own housework or doing activities without pay, such as voluntary work. 上一次访问时 [ZIWTime]，您有没有从事非农工作（包括挣工资工作、从事个体、私营经济活动或不拿工资为家庭经营活动帮工等，但不包括家务劳动、义务的志愿劳动）？

1. Yes 有

2. No 没有 → Skip FA006_w3_4 – FA006_w3_7 跳过 FA006_w3_4 – FA006_w3_7

FA006_w3_4 What non-agricultural work did you do in the last IW? 您当时主要从事的非农工作是?

1. Employed 挣工资工作
2. Self-employed 从事个体、私营经济活动 → Skip FA006_w3_6 跳至FA006_w3_6
3. Unpaid family business 不拿工资为家庭经营活动帮工 → Skip FA006_w3_7 跳至FA006_w3_7

FA006_w3_5 The detail information of employed work in the last IW. 当时从事的挣工资工作的具体情况

1. What is the name of your workplace/employer? Please state specifically the name of your company or institution. 您的工作单位或者雇主是什么名称?(请标明详细的单位名称)
   工作单位名称:_____ **(FA006_w3_5_1)** (例 1:北京大学餐饮服务中心;例 2:北京华信服装服饰有限责任公司)

2. 所在部门名称:_____ **(FA006_w3_5_2)** (例 1:农园餐厅;例 2:纺纱生产车间)
3. What kind of business or industry do you work in—that is, what does your workplace primarily make or do? 该单位主要是做什么的,也就是,他们制造什么产品或者从事什么活动?_____ **(FA006_w3_5_3)** (例 1:为校内提供餐饮服务;例 2:制造纱质布料)
4. What sort of work do you do? 您具体从事什么工作?_____ **(FA006_w3_5_4)** 例 1:餐厅面点厨师;例 2:生产线绕线工人)

[IWER: Type of business 访员注意:产业或行业类型。注意:回答中请不要用逗号]

**PROCEDURE 程序:**
Skip FA006_w3_6 – FA006_w3_7 跳过 FA006_w3_6 – FA006_w3_7

FA006_w3_6 The detail information of self-employed work in the last IW. 当时从事个体、私营经济活动的具体情况

1. What is the name of your company or workplace? 您的企业或者生意的名称是?(请标明详细的单位名称) _____ **(FA006_w3_6_1)** (例 1:北京华信服装服饰有限责任公司;例 2:丽丽服装店)
2. What kind of business or industry do you work in—that is, what does your company do or make? 您主要做什么生意,即生产什么产品或者从事什么经营活动? _____ **(FA006_w3_6_2)** (例 1:制造纱质布料;例 2:销售服装)

[IWER: If there is more than one company, ask about the main one. Mark 0 if there is no name 访员注意:如果在几个企业工作或者做了多项生意,问主要工作的企业或者主要从事的生意。如果没有名称,请标明"0"]

**PROCEDURE 程序:**
Skip FA006_w3_7 跳过 FA006_w3_7

FA006_w3_7 The detail information of unpaid family business in the last IW. 当时从事不拿工资为家庭经营活动帮工的具体情况

1. What is the name of your company or workplace that you work in without wage? 您无偿帮忙的企业或者生意的名称是？（请标明详细的单位名称）_____ (**FA006_w3_7_1**)（例 1：北京华信服装服饰有限责任公司；例 2：丽丽服装店）
2. What kind of business or industry did they work in—that is, what did their company do or make? 他们主要做什么生意，即生产什么产品或者从事什么经营活动？_____ (**FA006_w3_7_2**)（例 1：制造纱质布料；例 2：销售服装）
3. What sort of work do you do? 您具体从事什么工作？_____ (**FA006_w3_7_3**) 例 1：会计；例 2：帮店主销售服装）

FA007 BRANCHPOINT:

If REWIW is working now, or was working in the last IW, or have ever worked (ZF13=1 or ZF14=2 or XF1=1), then skip to FC001 BRANCHPOINT 如果回访受访者目前有工作，或者上一轮调查时有工作，或者这辈子曾经工作过，请跳至FC001 BRANCHPOINT

If REWIW had not worked prior to the last IW (ZF14= 1), and is not working now(XF1= 2), then skip to FA007 如果回访受访者上一轮调查之前从来没有工作过 (ZF14= 1)，同时现在没有工作 (XF1= 2)，请跳至FA007

If NEWIW is not working now(XF1= 2), then skip to FA007 如果新受访者现在没有工作 (XF1= 2)，请跳至FA007

FA007 Have you worked for at least three months during your lifetime (work includes agricultural work,earning wage work, self-employed activities, and unpaid family business work, et. al.)? 您这辈子是否曾经工作了至少三个月的时间？(工作包括务农、挣工资工作、从事个体、私营活动或不拿工资为家庭经营活动帮工等)

1. Yes 是 → Skip to FK Section BRANCHPOINT 请跳至 FK Section BRANCHPOINT
2. No 否

FA008 Work includes all kinds of labour excluding doing your own housework, whether you earn wages or not. Are you sure that you didn’t work at least three months during your lifetime? 我们所说的工作指所有除家务劳动以外的所有干活的劳动，不管是不是有工资。您确定这辈子没有工作过至少三个月的时间？

1. Yes, never worked before 是的，从未工作过
2. No, ever worked. 不是，以前工作过 → Skip to FK Section BRANCHPOINT 请跳至 FK Section BRANCHPOINT

FA009 What is the main reason for you not to work in your lifetime? 请问您不工作的主要原因是什么？

1. Disabled (physical or psychological) 残疾（包括生理和心理上的残疾）
2. Homemaker 操持家务
3. My family is too rich that I don’t need to work 我家太有钱了，我不需要工作
4. Taking care of siblings 照看兄弟姐妹
5. Other, _____ (**FA009_1**) 其他，请注明 _____ (**FA009_1**)

PROCEDURE 程序:

Skip to FK Section BRANCHPOINT 请跳至FK Section BRANCHPOINT

FC AGRICULTURE WORK 农业工作

FC001 BRANCHPOINT]:

If R (including new R and old R) did farming last year (FA001= 1), ask FC001 如果受访者(包括新受访者和老受访者)过去一年从事过农业工作(FA001= 1),询问FC001

if R did not do farming last year, but did non-agricultural work last week (XF2= 1), ask FC019 如果受访者过去一年没有从事农业工作,但是上周从事过非农工作(XF2= 1),询问FC019

FARM EMPLOYED 农业打工

FC001 Did you work for other famers in wage for at least ten days in the past year? (Agricultural work in wages) 过去一年,您有没有为其他农户打工至少 10 天?(指从事农业劳动挣钱)

1. Yes 是
2. No 否

[访员注意:"为其他农户打工"指为其他农户从事农业生产劳动挣钱]

HOUSEHOLD AGRICULTURAL WORK 自家农业生产活动

FC008 Did you work for your own household for at least ten days in the past year? 过去一年,您有没有为自家从事农业生产经营活动至少 10 天?

1. Yes 是
2. No 否 → skip to FC014 跳至 FC014

FC009 How many months did you work on [cropping (forestry), livestock, and fishing] for your own householdin the past year? 过去一年中,您有几个月为自家从事 [种植业及林业(包括种地、管理果树、采集农林产品)] 或 [畜牧业(饲养牲畜)] 或 [渔业(养殖水产品)]? _____ 0...12 Months 月

FC010 How many days did you work for your own household per week on average during a normal work month in the past year? 过去一年中,在您为自家从事 [种植业及林业(包括种地、管理果树、采集农林产品)] 或 [畜牧业 (饲养牲畜)] 或 [渔业(养殖水产品)] 的月份里,您一般每周干几天? _____ 0...7 Days 天

FC011 How many hours did you usually work for your own household per day during a normal work day in the past year? 过去一年中,在您为自家从事 [种植业及林业(包括种地、管理果树、采集农林产品)] 或 [畜牧业 (饲养牲畜)] 或 [渔业(养殖水产品)] 的日子里,您一般每天要干几个小时? _____ 0...24 Hours 小时

[A check similar to the one for FC006. FC011 > 16 is unreasonable]

FC012 Where is your workplace for most time? 大多数时间里，您工作的地点是？[preload sampling community ID]

1. The same as permanent address 和目前常住地一样
2. Another village/neighborhood in permanent address's county/city/district 常住地所在县/市/区的其他乡/镇/街道，_____ (**FC012_1**) village/neighborhood 村/社区
3. Other _____ (**FC012_2**) 其它 province_city_county/city/district 省/市/县/区，_____ (**FC012_3**) village/neighborhood 乡/镇/街道/村/社区
4. Abroad 国外

[访员注意：如果记不清详细的地址名称，请在相应输入框中填入"其它"]

FC012_w3 How satisfied are you with your job? 请问您对当前的工作满意吗？

1. Completely satisfied 极其满意
2. Very satisfied 非常满意
3. Some what satisfied 比较满意
4. Not very satisfied 不太满意
5. Not at all satisfied 一点也不满意

[访员注意：这里指的是对自家务农这份工作的满意度]

FC013 How many days of work did you miss last year due to health problems? 过去一年您有多少天因为健康原因干不了活？_____ 0...366 Days 天

[IWER: Mark 0 if you didn't miss any work days 访员注意：如果没有，请注明"0"]

FC014 Besides agricultural work, did you work for at least one hour last week in wage or self-employed work or umpaid family business? 除农业生产经营活动外，上周您有没有工作（包括挣工资工作、从事个体、私营活动或不拿工资为家庭经营活动帮工等）至少一个小时？

1. Yes 是 → skip to FC019 跳至 FC019
2. No 否

FC015 Do you have wage or self-employed work but are temporarily laid-off or are on sick, seasonal, or other leave or in-job training? 您是否有其他工作（包括挣工资工作、从事个体、私营活动或不拿工资为家庭经营活动帮工等），但是目前正暂时放假、由于生病或者季节原因休假、歇业或者处于其他假期中或正在在职培训？

1. Yes 是
2. No 否 → skip to FC018 跳至 FC018

FC016 In what month and year did leave or attend training? 您是什么时候开始休假/歇业/在职培训的？_____ 1900...2015 (**FC016_1**) Year 年 _____ 0...12 (**FC016_2**) Month 月

[IWER: Mark the year using four digits. Take down the month as its actual number. For example, write January as "1" not "01", December as "12". If do not remember month, fill "0" 访员注意：用 4 位数表示年，按照实际的月份填写月。例：1 月写作"1"，而不是"01"，12 月写作"12"。如果记不住月份，请填入"0"]

[Same check as the one for FA004. (Interview Year −FC016_1 + (Interview month − FC016_2)/12 > 1]

FC017 Do you expect to go back to this job at a definite time in the future or within 6 months? 您能够在确定的时间或者 6 个月以内，回到原来的工作岗位么？

1. Yes 是 → skip to FC019 跳至 FC019
2. No 否

FC018 At what age do you plan to stop working? Stopping work in this context shall refer to having stopped all income-related activities, unpaid family business and having no intention of engaging in anything more serious than small pastime work. 您计划在多大年龄时停止农业工作（自家务农），即停止从事一切以挣钱为目的的活动，也不再为家庭经营活动帮工，将来也不打算从事比消遣性工作更劳累的工作? _____ 1···120 Years old 岁

[IWER: Please tell me the approximate age. Mark 0 if you plan to keep working until you are physically able 访员注意：请告知大概年龄。如果您计划只要健康允许，就一直工作，请标明 “0”]

[访员注意：“消遣性工作”指不是以挣钱为目的的活动，以及不再为家庭经营活动帮工的工作]

PROCEDURE 程序:

Skip to FB011 BRANCHPOINT if IWR only have household agricultural work (FC001= 2 and FC008= 1 and FC014= 2) 如果受访者只从事自家农业生产经营活动的话，请跳至 FB011 BRANCHPOINT

Skip to FD001 if IWR only have farm employed work (FC001= 1 and FC008= 2 and FC014= 2) or have both farm employed work and household farm work (FC001= 1 and FC008= 1 and FC014= 2) 如果受访者只从事农业受雇工作的话或者既从事农业受雇又从事自家务农的话，请跳至 FD001

FC019 Besides agricultural work, do you currently hold more than one non-agricultural job? 除农业生产经营外，您目前有两份（及以上）的非农业工作吗？

[IWER: Non-agricultural job includes paid jobs, self-employed activities, unpaid family business work, et. al. Activities without pay, such as voluntary work, are not included 访员注意：工作包括挣工资工作、从事个体、私营活动或不拿工资为家庭经营活动帮工等，但没有工资的比如志愿者之类的工作不包括在内；此处务农不算工作，即如果一个人除了务农外只有一份工作，此处选 2]

1. Yes 是 → skip to FC020 跳至 FC020
2. No 否 → skip to FC021 跳至 FC021

FC020 Among all your jobs, which one is your main job? [Main job is defined as the job at which you work the longest hours] Do you earn a wage or do you run your own business or work for unpaid family business? 在这几份工作中，您目前的主要工作 [主要工作的定义：工作时间最长的工作] 是什么？是挣工资工作，从事个体或者私营经济活动，还是不拿工资为家庭经营活动帮工？

1. Employed 挣工资工作 → skip to FD001 跳至 FD001
2. Self-employed 从事个体或者私营经济活动 → skip to FH001 跳至 FH001
3. unpaid family business 不拿工资为家庭经营活动帮工 → skip to FH001 跳至 FH001

FC021 How do you describe your non-agricultural job? Do you earn a wage or do you run your own business or work for unpaid family business? 您怎么描述您现在的工作？是挣工资工作，从事个体或者私营经济活动还是不拿工资为家庭经营活动帮工？

1. Employed 挣工资工作 → skip to FD001 跳至 FD001
2. Self-employed 从事个体或者私营经济活动 → skip to FH001 跳至 FH001
3. unpaid family business 不拿工资为家庭经营活动帮工 → skip to FH001 跳至 FH001

[IWER: 前面回答了有农业打工（受雇）工作的话，这里选 1 挣工资工作]

FD EMPLOYED 受雇

FD001 Do you receive wages from your current workplace or receive them from a dispatch/contract company? 您的工资是从工作单位直接拿到还是要通过某个派遣单位拿？

1. Place of work 工作单位
2. Labor dispatch company 劳务派遣单位
3. Individual 个人

[访员注意：“工作单位”是指受访者当前从事劳动的单位。“派遣单位”是指劳动者签订合同，或者人事所在的单位，比如劳务输出公司。“个人”是指诸如包工头之类的工头或个体老板之类的情形]

[CAPI: For dispatched/contract workers (FD001= 2), mention the following for questions FD002 -FD016 对派遣工人 (FD001= 2)，对 FD002-FD016 均提示] The next few questions pertain to the situation at your current workplace, and not to the company that has dispatched/contracted you out. 下面几个问题是关于您为之工作的单位的情况，而不是派遣单位。]

FD002 Do you work for a government organization, institution, firm, NGO, individual farmer, or resident household? 您是为政府机构、事业单位、企业单位、非赢利结构（如社团、协会、学会等）、个体户、农户，还是居民户工作？

1. Government 政府部门
2. Institutions 事业单位
3. NGO 非营利机构（如社团、协会、学会等）
4. Firm 企业
5. Individual firm 个体户
6. Farmer 农户
7. Individual household 居民户
8. Other 其他

FD003 BRANCHPOINT:

For REWIW: If REWIW is working for the same employer (FA006_w2_2a_3= 1), skip FD003. 回访受访者：回访老受访者为同一个雇主工作（FA006_w2_2a_3= 1），跳过FD003

[INTRO: We will ask your work history later.In order to distinguish the work units, we need the full name and address of your employer 我们在后面的问卷还会问有关工作史的问题，为了便于区分不同的工作单位，我们需要单位名称]

FD003 What is the name of your workplace/employer? Please state specifically the name of your company or institution. 您的工作单位或者雇主是什么名称？（请标明详细的单位名称）
工作单位名称：_____ **(FD003_1)** （例 1：北京大学餐饮服务中心；例 2：北京华信服装服饰有限责任公司）
所在部门名称：_____ **(FD003_2)** （例 1：农园餐厅；例 2：纺纱生产车间）
[IWER: Write the name of the household head if R works for a family 访员注意：如果雇主是家庭，工作单位请写户主的姓名]

FD004 Where is your workplace located? 您工作单位的地点是？(preload sampling community ID)

1. The same as permanent address 和目前常住地一样
2. Another village/neighborhood in permanent address's county/city/district 常住地所在县/市/区的其他 _____ **(FD004_1)** 乡/镇/街道，_____ **(FD004_2)** village/neighborhood 村/社区
3. Other 其它：_____ **(FD004_3)** province_city_county/city/district 省 _ 市 _ 县/市/区，_____ **(FD004_4)** 乡/镇/街道，_____ **(FD004_5)** village/neighborhood 村/社区
4. Abroad 国外

[访员注意：如果记不清详细的地址名称，请在相应输入框中填入"其它"]

PROCEDURE 程序：
If R is working for individual farmer or resident household (FD002= 6, 7), skip to FD011
如果受访者为农户、居民户工作（FD002= 6, 7），请跳至FD011

FD005 What kind of business or industry do you work in—that is, what does your workplace primarily make or do? 该单位主要是做什么的，也就是，他们制造什么产品或者从事什么活动？_____ （例 1：为校内提供餐饮服务；例 2：制造纱质布料）
[IWER: Type of business 访员注意：产业或行业类型。注意：回答中请不要用逗号]

PROCEDURE 程序：
If government employee (FD002= 1), ask FD006 to FD007, then skip to FD011 如果是政府雇员，问FD006 to FD007，然后跳转到 FD011

FD006 Are you a civil servant? 您是公务员吗？

1. Yes 是
2. No 否

FD007 Are you a formal employee of an establishment? 您是正式编制内的员工吗？

1. Yes 是
2. No 否

PROCEDURE 程序：
If institution (FD002 = 2), ask FD009, then skip to FD011. 如果是事业单位，问 FD009，然后跳转到 FD011

FD009 Are you a formal employee of an establishment? 您是正式编制内的员工吗？

1. Yes 是
2. No 否

PROCEDURE 程序：

If firm (FD002= 4), ask from FD010, 如果是企业，从 FD010 开始问

FD010 What is the ownership type of the business? 您单位的所有制类型是？

1. 100% State owned firm 国有企业
2. State-controlled firm 国有控股企业
3. 100% Collective-owned firm 集体所有制企业
4. Collective-controlled firm 集体控股企业
5. 100% Private firm 私营/个体
6. Private-controlled firm 私人控股企业
7. 100% foreign-owned 外商独资
8. Joint venture 中外合资
9. Other joint-ownership 其他联营企业
10. Other 其他

FD011 When did you start working for this employer? 您什么时候开始在这个单位工作的？

_____ 1900...2015 (**FD011_1**) Year 年 _____ 0...12 (**FD011_2**) Month 月

[IWER: Mark the year using four digits. Take down the month as its actual number. For example, write January as "1" not "01", December as "12". If do not remember month, fill '0' 访员注意：用 4 位数表示年，按照实际的月份填写月。例：1 月写作"1"，而不是"01",12 月写作"12"。如果记不住月份，请填入"0"]

[Soft Check: Consistency of employment start date. Make Sure that the individual was at least a minimum age when he/she started working for this employer (e.g., prompt to check if under 16). Specifically (FD011_1+FD011_2/12)-(CV009_a+CV009_b/12)<16 prompts a soft check.]

[Replace 16 in the above soft check with FB001. (FD011_1 + FD011_2/12)-(CV009_a + CV009_b/12) < FB001_1 or FD011_1 < FB001_2]

PROCEDURE 程序：

如果受访者上一轮调查时不工作（ZF1 = 4），但是本轮 FD011 的回答FD011_1和FD011_2早于上一轮的调查时间 [ZIWTime]，询问FD011_w2_1和FD011_w2_2

FD011_w2_1 You told us that you were not working for someone else in the last IW, but you said you have worked since [(FD011_1) Year(FD011_2) Month]. Is our record wrong? 上一轮调查时 [ZIWTime] 您告诉我们您不工作，但是刚才您说您 [(FD011_1) 年 (FD011_2) 月] 就开始现在这份工作了，是不是上一轮调查时我们的访员记录错了？

1. Yes, I worked in last IW 上次访员记录错误，上一轮的时候就有工作 → skip to FD012 跳至FD012

2. No 上次访员记录正确，上一轮调查时确实不工作

FD011_w2_2 When did you begin working in this unit? 您开始在现在这个单位工作的准确时间是？ _____ 1900...2015 (**FD011_w2_2_1**) Year 年 _____ 0...12 (**FD011_w2_2_2**) Month 月

[FD011_w2_2回答的时间需在上一轮调查（ZIWTime）与本轮调查时间之间]

FD012 What sort of work do you do? 您具体从事什么工作？ _____ (**FD012_1**) 例 1：餐厅面点厨师；例 2：生产线绕线工人）是否有相关的职业资格证书？ _____ (**FD012_2**)（例 1：有高级中式烹调师证；例 2：没有职业资格证书）→

[IWER: Ask about the specific work that R does 访员注意：请详细填写具体工作内容。注意：回答中请不要用逗号]

FD013 What is your current position? 您目前的职务是什么？

1. Clerk/worker 普通职工
2. Team Leader 组长（股长）
3. Section Chief 科长
4. Director of a division 处长
5. Director-General of a bureau and above 局长及以上
6. Village Leader 村干部
7. Township Leader 乡镇干部
8. Division manager 单位部门经理
9. Overall/General manager 单位总经理
10. Other, _____ (**FD013_1**) 其他，请注明 _____ (**FD013_1**)

FD014 What is your current professional/technical level? 你目前的专业/技术职称是什么？

1. Technician 技术员
2. Primary level 初级职称
3. Intermediate level 中级职称
4. Advanced level 高级职称
5. No professional/technical level 无职称

[访员注意："专业/技术职称"指专业技术人员的专业技术与学识水平和工作能力的等级称号]

FD015 Are you in a position to supervise others? 您是否管理别人？

1. Yes 是
2. No 否 → Skip to FD017. 跳至FD017

FD016 How many people are there under your supervision? 您管着多少人？

1. 1~5 people 1~5 人
2. 6~10 people 6~10 人
3. 11~15 people 11~15 人
4. 16~30 people 16~30 人
5. 31~99 people 31~99 人

6. More than 100 people 超过 100 人

[CAPI: For dispatched worker(FD001 = 2), prompt for FD017-FD031 对派遣工人 (FD001= 2), 对FD017-FD030 均提示：

The next few questions are about your dispatch work unit. 下面几个问题是关于您派遣单位的情况。]

PROCEDURE 程序:
Do not ask FD017 if FD013= 3 – 5 (government officials) 如果FD013= 3 – 5（政府官员），跳过FD017

FD017 What is your employment type at your current workplace? 您目前的受聘类型是？
1. Regular worker 劳务派遣工
2. Contract worker 合同制员工
3. Casual/Part-time worker 日工/钟点工

FD020 Did you receive a labor contract (or employment contract) in written form from your current workplace(or labor dispatch company)? 您与当前单位/劳务派遣单位签了书面的劳动合同吗？
1. Yes 是
2. No 否 → Skip to FD024. 跳至FD024

FD021 What is the agreed period of employment (labor contract period)? 您的劳动合同的期限是多长？
1. Defined period 固定期限 _____ 0...100 (**FD021_1**) Years 年 _____ 0...11 (**FD021_2**) Months 月
   [IWER: If do not remember months, please fill '0' 访员注意：如果记不住多少个月，请填"0"]
2. Not defined 无固定期限 → Skip to FD024. 跳至 FD024
3. Same as the term of the project 以完成一定工作任务为期限的劳动合同

FD022 Has the current employment contract ever been renewed? 现在的劳动合同是续签过的吗？
1. Yes 是
2. No 否 → Skip to FD024. 跳至FD024

FD023 How many times has the contract been renewed? 您的劳动合同是第几次续签？_____ 1...50 times 次

FD024 How long do you expect to work at your current workplace? 您预期还会在现在的单位干多久？
1. Less than one year 不到 1 年
2. One to two years 1-2 年

3. Two to three years 2-3 年 → Skip FD025. 请跳过FD025
4. More than three years 超过 3 年 → Skip FD025. 请跳过FD025

FD025 Why do you expect so? 你为什么这么觉得呢?
1. Because the predefined contract period will expire 因为之前签的合同快到期了
2. Because typically the contract expires (although there's no written contract) 因为之前约定的时间快到了（尽管没有书面合同）
3. Because I was hired under the condition that I would resign upon the request of my employer 因为估计雇主会让我辞职
4. Because the current job/project will be completed 因为目前的工作/项目即将结束
5. Because the person I am substituting/replacing will return to work 因为我顶替的那个人要回来了
6. Because I can only work during certain seasons 因为我只能在某些时间段工作
7. Because I plan to find another job that better suits my job aptitude, abilities, and preferences 因为我想找一份更适合我的工作
8. Because I will reach retirement age as set by regulations/practice 因为我快到退休年龄了
9. Because of family care responsibilities, poor health, etc. 因为家庭或健康方面的原因
10. Other 其他

FD029 Except for national/public holidays, how many days of paid vacation do you have this year at your current workplace? 除国家法定节假日以外，今年您可以享受几天的带薪休假? _____ 0...366 Days 天

[IWER: Mark 0 if there is no paid vacation 访员注意：如果没有带薪休假，请标明“0”]

[Soft Check for reasonable range: If FD029> 30, prompt for verification]

FD030 How many days of work did you miss at this current job in the past year due to health problems? 在过去的一年中，您由于健康原因，在这个单位请假多少天? _____ 0...366 Days 天

[IWER: Mark 0 if you didn't miss work 访员注意：如果您没有不在职的情况，请标明“0”]

PROCEDURE 程序:
If FD030= 0, skip FD031. 程序：如果FD030= 0，跳过FD031

FD031 In these days, how many did not deduct wage or bonus? 在这些天里，有多少天是不扣工资或奖金的? _____ 0...366 Days 天

[Soft Check for reasonable range: If FD031>FD030, prompt for verification]

FD032_w3 Do employer buy the personal accident insurance for you？请问您的雇主给您买了人生意外伤害保险了吗?
1. Yes 有
2. No 没有

PROCEDURE 程序:
If FD032_w3= 2, skip FD033_w3. 程序：如果 FD032_w3= 2，跳过 FD033_w3

FD033_w3 How much money can you receive if you encounter the catastrophe accident (e.g. death) ? _____ 如果您发生了意外伤害，最高能拿到多少钱的赔偿? _____

FE QUESTIONS ABOUT LABOR SUPPLY 劳动力供给

[CAPI: For dispatched worker(FD001= 2), prompt for FE001-FE003 对派遣工人 (FD001= 2), 对FE001-FE003 均提示] The next few questions about labor supply are about the situation of your work place, not dispatch work unit. 下面几个关于劳动力供给的问题是指您工作单位的情况，而不是派遣单位。

FE001 Counting paid vacations and sick leave not deducting wage as work, how many months did you work in the past year? 过去一年中，您工作了几个月（带工资的假期和不扣工资的病假算作工作时间）? _____ 0...12 Months 月

FE002 How many days a week did you work on average in the past year? 过去一年中，您一般每周工作几天? _____ 0...7 Days 天

FE003 How many hours did you work per day on average in the past year, excluding meal breaks but including any paid or unpaid overtime? 过去一年中，您一般每天工作多少个小时? 工作时间不包括午休时间，但包括加班时间（不管是否有报酬）_____ 0...24 Hours 小时
[Soft Check: Verify if number of hours per day is unreasonable, e.g., FE003 >16]

FF QUESTIONS ABOUT WAGES 受雇工作的工资问题

[CAPI: For dispatched worker(FD001= 2), prompt for FF001-FG002 对派遣工人 (FD001= 2), 对 FF001-FG002均提示] The following questions about salary and benefits refer to what you receive from the dispatch company. 下面几个问题是关于您从派遣单位获得工资和福利的情况。

FF001 How is your wage paid mainly? Is it regularly paid, contract-based, performance-based, or other? If it is regularly paid, please tell me how often you receive your wages. Do you have a yearly contract, monthly, weekly, daily, or hourly? Please select one. 您的工资主要是怎么支付的？是定期支付、按项目支付、按绩效支付还是其他？如果是定期支付，请回答您是多久领一次工资？您是否按年、月、周、日还是小时领取工资？请选择一个

1. Yearly salary 年薪
2. Monthly salary 月薪 → Skip to FF004. 跳至FF004
3. Weekly salary 周薪 → Skip to FF006. 跳至FF006
4. Daily salary 日薪 → Skip to FF008. 跳至FF008
5. Hourly salary 小时工资 → Skip to FF010. 跳至FF010

6. Contract-based 按项目 → Skip to FF012. 跳至FF012
7. Performance-based 按绩效 → Skip to FF012. 跳至FF012
8. Other 其他 → Skip to FF012. 跳至FF012

FF002 What is the after-tax salary including bonus in the last year? 把奖金等各种收入都算在内，您过去一年从单位拿到多少钱（扣除社会保险和个人所得税后）? _____ (**FF002_1**) Yuan 元其中，奖金有多少? _____ (**FF002_2**) Yuan 元或 _____ (**FF002_3**)%
[Soft Check: Prompt for clarification if under a low threshold, e.g. under 1200 RMB annual. Specifically, prompt to clarify if FF002< 1200 RMB.]

FF003 [IWER: If R is unwilling to answer or does not remember, ask unfolding bracket questions here 访员注意：如果受访者不愿回答或者忘记了，在此处分级展开提问] 把奖金等各种收入都算在内，您过去一年从单位拿到多少钱（扣除社会保险和个人所得税后）？10,000 /30,000 /50,000 /100,000 /200,000 yuan 元

FF003_w2 [IWER: If R is unwilling to answer or does not remember, ask unfolding bracket questions here 访员注意：如果受访者不愿回答或者忘记了，在此处分级展开提问] 其中，奖金有多少？10,000 /30,000 /50,000 /100,000 /200,000 yuan 元

PROCEDURE 程序:
Skip to FF014. 跳至 FF014

FF004 What is the after-tax salary including bonus in the last month? 把奖金等各种收入都算在内，您上个月从单位拿到多少钱（扣除社会保险和个人所得税后）? _____ (**FF004_1**) Yuan 元其中，奖金有多少? _____ (**FF004_2**) Yuan 元或 _____ (**FF004_3**)%
[Soft Check: Prompt for clarification if under a low threshold, e.g. under 100 RMB per month. Specifically, prompt to clarify if FF004 < 100 RMB.]

FF005 [IWER: If R is unwilling to answer or does not remember, ask unfolding bracket questions here 访员注意：如果受访者不愿回答或者忘记了，在此处分级展开提问] 把奖金等各种收入都算在内，您上个月从单位拿到多少钱（扣除社会保险和个人所得税后）？500 /1,000 /2,500 /5,000 /10,000 yuan 元

FF005_w2 [IWER: If R is unwilling to answer or does not remember, ask unfolding bracket questions here 访员注意：如果受访者不愿回答或者忘记了，在此处分级展开提问] 其中，奖金有多少？500 /1,000 /2,500 /5,000 /10,000 yuan 元

PROCEDURE 程序:
Skip to FF014. 跳至 FF014

FF006 What is the wage including bonus last week? 把奖金等各种收入都算在内，您上周从单位拿到多少钱（扣除社会保险和个人所得税后）? _____ Yuan 元
[Soft Check: Prompt for clarification if under a low threshold, e.g. under 25 RMB per week. Specifically, prompt to clarify if FF006 < 25 RMB.]

PROCEDURE 程序:
Skip to FF012. 跳至 FF012

FF008 What is the usual daily wage? 您一般每天挣多少钱? _____ Yuan 元
[Soft Check: Prompt for clarification if under a low threshold, e.g. under 5 RMB per day. Specifically, prompt to clarify if FF008 < 5 RMB.]

FF009 [IWER: If R is unwilling to answer or does not remember, ask unfolding bracket questions here 访员注意: 如果受访者不愿回答或者忘记了, 在此处分级展开提问] 20 /50 /100 /200 /500 yuan 元

PROCEDURE 程序:
Skip to FF012. 跳至 FF012

FF010 What is your hourly wage? 您每小时的工资是多少? _____ Yuan 元
[Soft Check: Prompt for clarification if under a low threshold, e.g. under 1 RMB per hour. Specifically, prompt to clarify if FF010 < 1 RMB.]

FF011 [IWER: If R is unwilling to answer or does not remember, ask unfolding bracket questions here 访员注意: 如果受访者不愿回答或者忘记了, 在此处分级展开提问] 10 /30 /50 /100 /200 yuan 元

PROCEDURE 程序:
Skip to FF012. 跳至 FF012

FF012 How much on average do you receive last month after taxes (including bonus)? 把奖金等各种收入都算在内, 您上个月扣除社会保险和个人所得税后拿到多少钱? _____ **(FF012_1)** Yuan 元其中, 奖金有多少? _____ **(FF012_2)** Yuan 元或 _____ **(FF012_3)**%
[Soft Check: Prompt for clarification if under a low threshold, e.g. under 100 RMB per month. Specifically, prompt to clarify if FF012 < 100 RMB.]

FF013 [IWER: If R is unwilling to answer or does not remember, ask unfolding bracket questions here 访员注意: 如果受访者不愿回答或者忘记了, 在此处分级展开提问] 把奖金等各种收入都算在内, 您上个月扣除社会保险和个人所得税后拿到多少钱? 1,000 /3,000 /5,000 /10,000 /20,000 yuan 元

FF013_w2 [IWER: If R is unwilling to answer or does not remember, ask unfolding bracket questions here 访员注意: 如果受访者不愿回答或者忘记了, 在此处分级展开提问] 其中, 奖金有多少? 1,000 /3,000 /5,000 /10,000 /20,000 yuan 元

FF014 What is the value of all other bonuses (not paid at same time as regular wage) received in the past year? 过去一年中, 您从单位拿到的其他所有奖金 (不和薪酬一起按期支付, 比如年终奖等) 一共有多少钱 (扣除社会保险和个人所得税后)? _____ Yuan 元
[Soft Check: Prompt on bonus and monthly earnings if bonus is more than five times monthly net income]

FF015 [IWER: If R is unwilling to answer or does not remember, ask unfolding bracket questions here 访员注意: 如果受访者不愿回答或者忘记了, 在此处分级展开提问] 1,000 /3,000 /5,000 /10,000 /20,000 yuan 元

FG Fringe Benefits 单位福利

[Show Card 出示卡片 21]

FG001 The following are fringe benefits which may be provided by a company. Please answer if the following are provided by your current workplace and whether you benefit from the following. (Check all that apply) 下面我们将列举一些福利。请回答您目前公司的福利情况以及您是否享受以下福利？(可多选)

1. Free lunch 免费午餐
2. Free breakfast 免费早餐
3. Free dinner 免费晚餐
4. Meal cash subsidy 餐费补贴
5. Transportation cash subsidizations 交通费补贴
6. Free housing 免费住宿
7. Subsidization of housing 住房补贴
8. Company car 单位配车
9. Company bus 单位班车
10. Other subsidies, _____ **(FG001_1)** 其他补贴(包括实物折合), 请注明 _____ **(FG001_1)**
11. None 没有

**PROCEDURE 程序:**
For each choice of FG001, ask FG002. 对上题的每一个答案，都分别提问 FG002

FG002 How much is the value of the subsidy per month? 每个月这种补助有多少（值多少钱）?
_____ Yuan 元

**PROCEDURE 程序:**
Skip to FJ001. 跳至 FJ001

FH NON-FARM SELF-EMPLOYED AND UNPAID FAMILY BUSINESS 非农自雇和为家庭经营活动帮工

FH001 How many months did you work in the past year? 过去一年中, 您工作了几个月? _____ 0...12 Months 月

FH002 How many days did you work per week on average in the past year? 过去一年中，您一般每周工作几天? _____ 0...7 Days 天

FH003 How many hours did you work per day on average in the past year, excluding meal breaks but including any paid or unpaid overtime on a normal work month? 过去一年中，您一般每天工作多少个小时? 不包括午餐和其他休息时间，但是包括加班时间，不管是否有报酬 _____ 0...24 hours 小时
[Soft Check: Verify if number of hours per day is unreasonable, e.g., FH003 >16]

FH004 How many days of work did you miss in the past year due to health problems? 过去一年中，您由于健康原因，有多少天没有干活？ _____ 0...366 Days 天
[IWER: Mark 0 if you didn't miss any work days 访员注意：如果没有，请注明“0”]

[Next are some questions about the main self-employed work 下面是关于您从事的主要个体或者私营经济活动的问题]

PROCEDURE 程序:
IF FC020= 2 or FC021= 2[Self-employed], ask 如果 FC020= 2 或者 FC021= 2，受访者自雇，询问:
If old R is running the same company or workplace (FA006_w2_2b_3= 1), skip FH005 如果老受访者开同一家公司/做同一个生意，跳过 FH005

FH005 What is the name of your company or workplace? 您的企业或者生意的名称是？(请标明详细的单位名称) _____ （例 1：北京华信服装服饰有限责任公司；例 2：丽丽服装店）[IWER: If there is more than one company, ask about the main one. Mark 0 if there is no name 访员注意：如果在几个企业工作或者做了多项生意，问主要工作的企业或者主要从事的生意。如果没有名称，请标明“0”]

FH006 Where is your company or workplace located? 您的公司/生意的地址是？
1. The same as permanent address 和目前常住地一样
2. Another village/neighborhood in permanent address's county/city/district 常住地所在县/市/区的其他 _____ (**FH006_1**) 乡/镇/街道，_____ (**FH006_2**) village/neighborhood 村/社区
3. Other 其它：_____ (**FH006_3**) province_city_county/city/district 省 _ 市 _ 县/市/区，_____ (**FH006_4**) 乡/镇/街道，_____ (**FH006_5**) village/neighborhood 村/社区
4. Abroad 国外

[IWER: 访员注意：如果记不清详细的地址名称，请在相应输入框中填入“其它”]

FH007 What kind of business or industry do you work in—that is, what does your company do or make? 您主要做什么生意，即生产什么产品或者从事什么经营活动？ _____ （例 1：制造纱质布料；例 2：销售服装）
[IWER: Type of business 产业或行业类型。注意：回答中请不要用逗号]

PROCEDURE 程序:
If old R is running the same company or business(FA006_w2_2b_3= 1), skip FH008 如果老受访者开同一家公司/做同一个生意，跳过 FH008

FH008 When did you start working at the current company or workplace? 您什么时候开始经营这个企业/这项生意？ _____ 1900...2015 (**FH008_1**) Year 年 _____ 0...12 (**FH008_2**) Month 月
[IWER: Mark the year using four digits. Take down the month as its actual number. For example, write January as “1” not “01”, December as “12”. If do not remember

month, fill '0' 访员注意: 用 4 位数表示年，按照实际的月份填写月。例：1 月写作"1"，而不是"01"，12 月写作"12"。如果记不住月份，请填入'0']
[A check similar to the one for FD011. (FD011_1 + FD011_2 /12)- (CV009_a + CV009_b/12) < 16 or (FD011_1 + FD011_2 /12)- (CV009_a + CV009_b/12) < FB001_1 or FH008_1 < FB001_2]

FH009 Do any other household members work in the same self-employed activity? 您是否还有其他家人也从事同样的个体或者私营经济活动？

1. Yes 是
2. No 否

[IWER: Income from self-employment if R is the single operator（FH009= 2）. If other family member involved, income is asked in household section 访员注意：如果受访者是自己一个人开公司、做生意等，那么接下来问收入；如果还有其他家人一起做事，那么在家庭问卷中问收入]

FH010 Not including spending on fixed capital, what is your best estimate of net income earned from this activity in the last year? Remember to consider the following types of costs: energy, housing or equipment rental, raw materials, transportation, marketing, wages, taxes, and other fees. 不包括在固定资本上的投入，您能否估计一下过去一年您开这个公司/做这项生意挣了多少钱？我们指的是刨除能源、住房和设备租用费、原材料、交通费、营销、工资、税收和杂费之后的净收入。_____ Yuan 元
[Soft Check: Prompt for verification if net income is less than 1200 RMB / year. Or, prompt if FH010 <1200 RMB]

FH011 [IWER: If R is unwilling to answer or does not remember, ask unfolding bracket questions here 访员注意：如果受访者不愿回答或者忘记了，在此处分级展开提问] 5,000 /10,000 /50,000 /100,000 /200,000 yuan 元

PROCEDURE 程序:
Skip to FJ001. 跳至 FJ001

[Next are some questions about your unpaid family business 下面是关于您不拿工资为家庭经营活动帮工的问题]

PROCEDURE 程序:
If old R is working for the same company or workplace without wage (FA006_w2_2c_3= 1), skipFH012 如果老受访者为同一家公司/生意不拿工资帮工，跳过FH012

FH012 What is the name of company or workplace that you work in without wage? 您无偿帮忙的企业或者生意的名称是？_____（例 1：北京华信服装服饰有限责任公司；例 2：丽丽服装店）
[IWER:Mark 0 if there is no name 访员注意：如果没有名称，请标明"0"]

FH013 Where is this company or workplace loacted? 这个公司/生意的地址是？
1. The same as permanent address 和目前常住地一样
2. Another village/neighborhood in permanent address's county/city/district 常住地所在县/市/区的其他 _____ **(FH013_1)** 乡/镇/街道，_____ **(FH013_2)** village/neighborhood 村/社区
3. Other 其它：_____ **(FH013_3)** province_city_county/city/district 省 _ 市 _ 县/市/区，_____ **(FH013_4)** 乡/镇/街道，_____ **(FH013_5)** village/neighborhood 村/社区
4. Abroad 国外

[访员注意：如果记不清详细的地址名称，请在相应输入框中填入"其它"]

FH014 What kind of business or industry do you work in—that is, what does this company do or make? 他们主要做什么生意，即生产什么产品或者从事什么经营活动？_____（例 1：制造纱质布料；例 2：销售服装）

[IWER: Type of business 产业或行业类型。注意：回答中请不要用逗号]

FH015 What sort of work did you do? 您具体从事什么工作？_____（例 1：会计；例 2：帮助店主销售服装）

[IWER: Ask the specific work 访员注意：请详细填写具体工作内容。注意：回答中请不要用逗号]

FH016 How many family members, relatives or friends who work without payment are there including yourself? 您有多少家人、亲戚或朋友在这个公司/生意不拿工资做事的？（包括您本人）_____ People 人

FH017 When did this company or workplace start operation? 这个企业/生意是什么时候开办/经营的？_____ 1900...2015 **(FH017_1)** Year 年 _____ 0...12 **(FH017_2)** Month 月

FH018 When did you start working at the current company or workplace? 您什么时候开始在这个企业/生意帮忙的？_____ 1900...2015 **(FH018_1)** Year 年 _____ 0...12 **(FH018_2)** Month 月

FH019 Do any other household members work in the same company or workplace? 您是否还有其他家人也在这个企业/生意工作？
1. Yes 是
2. No 否

[IWER: Income from self-employment if no other household members work in（FH019= 2）. If other family member involved, income is asked in household section 访员注意：如果没有其他家人在企业/生意工作，那么接下来问收入；如果还有其他家人一起做事，那么在家庭问卷中问收入]

FH020 Not including spending on fixed capital, what is your best estimate of net income earned from this activity in the last year? Remember to consider the following types of costs: energy, housing or equipment rental, raw materials, transportation, marketing,

wages, taxes, and other fees. 不包括在固定资本上的投入，您能否估计一下过去一年这个公司/做这项生意挣了多少钱？我们指的是刨除能源、住房和设备租用费、原材料、交通费、营销、工资、税收和杂费之后的净收入。_____ Yuan 元
[Soft Check: Prompt for verification if net income is less than 1200 RMB / year. Or, prompt if FH020 $<$ 1200 RMB]

FH020_bracket [IWER: If R is unwilling to answer or does not remember, ask unfolding bracket questions here 访员注意：如果受访者不愿回答或者忘记了，在此处分级展开提问] 5,000 /10,000 /50,000 /100,000 /200,000 yuan 元

FJ SIDE JOB (EMPLOYED OR SELF-EMPLOYED) 非主要职业（受雇或自雇）

If FC019 = 1(more than one job), proceed with the following section. 如果 FC019 = 1(工作超过一份）问这部分

FJ001 How many jobs do you currently hold, excluding your main job? 除了主要工作，您目前还从事其他几份工作？_____ 1...20 份

FJ002 How many hours a week do you work on average at your side job(s), not considering your main job? 除了主要工作，您每周在其他工作上平均工作多少个小时？_____ 0...168 Hours per week 小时/周

FJ003 What is the average monthly income or wage that you get from side job(s) other than your main job? 除了主要工作的收入外，您从事其他工作每月的平均收入是？_____ Yuan per month 元/月

FJ004 [IWER: If R is unwilling to answer or does not remember, ask unfolding bracket questions here 访员注意：如果受访者不愿回答或者忘记了，在此处分级展开提问] 500 /1,000 /2,500 /5,000 /10,000 yuan 元

FK UNEMPLOYMENT AND JOB SEARCH ACTIVITIES 失业，求职经历

FK000_w3 BRANCHPOINT:
如果 FC019 不等于 missing， 问 FK000_w3

FK000_w3_1 How satisfied are you with your job? 请问您对当前的工作满意吗？
1. Completely satisfied 极其满意
2. Very satisfied 非常满意

3. Some what satisfied 比较满意
4. Not very satisfied 不太满意
5. Not at all satisfied 一点也不满意

FK000_w3_2 At what age do you plan to stop working? Stopping work in this context shall refer to having stopped all income-related activities, unpaid family business and having no intention of engaging in anything more serious than small pastime work. 您计划在多大年龄时停止工作，即停止从事一切以挣钱为目的的活动，也不再为家庭经营活动帮工，将来也不打算从事比消遣性工作更劳累的工作?_____ 1⋯120 Years old 岁

[IWER: Please tell me the approximate age. Mark 0 if you plan to keep working until you are physically able 访员注意：请告知大概年龄。如果您计划只要健康允许，就一直工作，请标明“0”]

[访员注意："消遣性工作"指不是以挣钱为目的的活动，以及不再为家庭经营活动帮工的工作]

FK Section BRANCHPOINT:
- If new R is not working but has worked before (FA007= 1 or FA008= 2), or if new R never worked (FA007= 2 and FA008= 1) before , answer FK. 如果新受访者现在没有工作但是曾经工作过，或者至今从未工作过，询问 FK 部分
- If old R is not working (XF1= 2) but worked before (ZF13= 1 or ZF14= 2) or if old R never worked (FA007= 2 and FA008= 1) before, answer FK 如果老受访者现在没有工作但是曾经工作过，或者从未工作过，询问 FK 部分

FK001 BRANCHPOINT :
Please skip FK001 if R never worked(FA007 = 2 and FA008 = 1). 如果受访者从未工作过 (FA007 = 2 and FA008 = 1)，跳过FK001

FK001 Next are some questions about circumstances about your non-employment and job search activities. In what month and year did you last work? 下面是一些关于您停止工作前后的一些情况和找工作的问题。您什么时候开始不工作的？_____ 1900...2015 (**FK001_1**) Year 年 _____ 0...12 (**FK001_2**) Month 月

[IWER: Mark the year using four digits. Take down the month as its actual number. For example, write January as “1” not “01”, December as “12”. If do not remember month, fill ‘0’ 访员注意：用 4 位数表示年，按照实际的月份填写月。例：1 月写作“1”，而不是“01”,12 月写作“12”。如果记不住月份，请填入’0’]

FK002 Did you search for a new job during the last month? 过去一个月您是否找过工作？
1. Yes 是
2. No 否 → Skip to **FL Section** 请跳至 **FL Section**

FK003 At what age do you plan to stop working? Stopping work in this context shall refer to having stopped all income-related activities, unpaid family business and having no intention of engaging in anything more serious than small pastime work. 您计划在多大年龄时停止工作，即停止从事一切以挣钱为目的的活动，也不再为家庭经营活动帮工，将来也不

打算从事比消遣性工作更劳累的工作?_____ 1⋯120 Years old 岁
[IWER: Please tell me the approximate age. Mark 0 if you plan to keep working until you are physically able 访员注意：请告知大概年龄。如果您计划只要健康允许，就一直工作，请标明“0”]
[访员注意：“消遣性工作”指不是以挣钱为目的的活动，以及不再为家庭经营活动帮工的工作]

FL LAST JOB 最近一份工作

FL001 BRANCHPOINT：
For Old R: 老受访者：If old R’s last job is not missing (ZF22_1 = 1), skip FL. 如果老受访者最近一份工作没有缺失，跳过 FL 部分 If old R’s last job is missing (ZF22_1 = 2), please skip to procedure before FL001. 如果老受访者最近一份工作缺失，请跳至FL001前的程序控制
For new R: 新受访者：Please skip to procedure before FL001. 新受访者，请跳至FL001前的程序控制

PROCEDURE 程序:
目前没有工作的人，但曾经工作过的人回答 Ask if FA007 = 1 or FA008 = 2
从没工作过的人 (FA007 = 2 且 FA008 = 1)，跳到 FN002_w3 If FA007 = 2 and FA008 = 1, skip to FN002_w3

[IWER：The next questions are about the last main job you had, which could be farming, earning a wage, running your own business or working for unpaid family business. It does not include doing your own housework or doing activities without pay, such as voluntary work. If you have more than one job, we are interested in the job at which you worked the longest hours. We’re interested in your situation near the termination of this job 下面的问题是关于您最近一份工作的情况，可以是务农、挣工资工作、从事个体、私营经济活动、或者不拿工资为家庭经营活动帮工，但不包括家务劳动或者志愿者劳动等没有报酬的工作。如果您之前同时从事不止一份工作，我们想了解您的主要工作，即占用您时间最多的那份工作]

FL001 Did you work for someone else(including work for unpaid family business), were you self-employed, did you farm, or were you otherwise employed? 您最近一份工作是为别人工作（包括不拿工资为家庭经营活动帮工）还是自己从事个体或者私营经济活动，或者务农？
1. Employed 受雇（包括非农打工和在外地的农业打工等）
2. Self-employed 自雇（从事个体或者私营经济活动）
3. Unpaid family business 不拿工资为家庭经营活动帮工
4. Farming 农业生产活动（农业生产经营活动，包括为自家或其他农户种地、管理果树、采集农林产品、养鱼、打鱼、养牲畜以及去市场销售自家生产的农产品等；但不包括在外地的农业打工）

FL002 In which year and month did you start working at that job? 您从哪年哪月开始做这份工作的？_____ 1900...2015 (**FL002_1**) Year 年 _____ 0...12 (**FL002_2**) Month 月

[IWER: Mark the year using four digits. Take down the month as its actual number. For example, write January as "1" not "01", December as "12". If do not remember month, fill '0' 访员注意：用 4 位数表示年，按照实际的月份填写月。例：1 月写作"1"，而不是"01",12 月写作"12"。如果记不住月份，请填入'0'] [Soft Check: Prompt for Verification/Clarification if the Respondent was Less than 16 at time of starting this job, e.g. Prompt if (FL002_1+FL002_2 /12)-(CV009_a+CV009_b/12)<16] [Replace 16 in the above check with FB001 . FL002_1 < FB001_2 or FL002_1 < (CV009_a + FB001_1)]

FL003 In which year and month did you stop working at that job? 您什么时候停止做这份工作的？ _____ 1900...2015 (**FL003_1**) Year 年 _____ 0...12 (**FL003_2**) Month 月
[IWER: Mark the year using four digits. Take down the month as its actual number. For example, write January as "1" not "01", December as "12". If do not remember month, fill '0' 访员注意：用 4 位数表示年，按照实际的月份填写月。例：1 月写作"1"，而不是"01",12 月写作"12"。如果记不住月份，请填入'0'] [Soft Check: Prompt for Verification/clarification if End Date is Before the Start Date, e.g. FL003_1 < FL002_1]

FL004 BRANCHPOINT：
For old R, if FL003_1<Last IW Year （ZIWYear）or FL003_1=Last IW Year （ZIWYear）and FL003_2<Last IW Month （ZIWMonth），then skip to Section FM 对于老受访者，如果FL003_1< 上一轮调查年（ZIWYear）或者 FL003_1= 上一轮调查年（ZIWYear）并且 FL003_2< 上一轮调查月（ZIWMonth），请跳至 FM 部分

FL004 Where was the job located? 工作地点在哪里？
1. The same as permanent address 和目前常住地一样
2. Another village/neighborhood in permanent address's county/city/district 常住地所在县/市/区的其他 _____ (**FL004_1**) 乡/镇/街道，_____ (**FL004_2**) village/neighborhood 村/社区
3. Other 其它：_____ (**FL004_3**) province_city_county/city/district 省 _ 市 _ 县/市/区，_____ (**FL004_4**) 乡/镇/街道，_____ (**FL004_5**) village/neighborhood 村/社区
4. Abroad 国外

[IWER: 访员注意：如果记不清详细的地址名称，请在相应输入框中填入"其它"]

PROCEDURE 程序:
If FL001 = 4 Skip to FL020 如果FL001 = 4，跳至FL020
If non-agricultural work (FL001= 1/2/3), ask FL005 to FL008 . 如果是非农工作 (FL001= 1/2/3)，问FL005 to FL008

FL005 What was the name of your workplace/employer? Please state specifically the name of your company or business. 您的工作单位或者雇主是什么名称？（请详细标明名称）工作单位名称：_____ (**FL005_1**) （例 1：北京大学餐饮服务中心；例 2：北京华信服装服饰有限责任公司），所在部门名称：_____ (**FL005_2**) （例 1：农园餐厅；例 2：纺纱生产车间）

FL006 What kind of business or industry was it—that is, what did they make or do at the place where you worked? 该单位主要是做什么的，也就是，他们制造什么产品或者从事什么活动？ _____ （例 1：为校内提供餐饮服务；例 2：制造纱质布料）
[IWER: Type of business 访员注意：产业或行业类型。注意：回答中请不要用逗号]

FL007 Is this employer still in existence? 这个单位还存在吗？
1. Yes 是
2. No 否

FL008 How many hours a week did you usually work [for this employer/in this business]? 您当时一般每周工作几个小时？ [做这份工作 _____ 0...168 Hours per week 小时/周
[Soft Check: Prompt for Verification if FL008 > 80]

**PROCEDURE 程序:**
Ask only if employed. 只询问受雇（FL001= 1）

FL009 What were the monthly wages, bonuses, and subsidies from this job, before you stopped working at this job? 您停止做这份工作前，平均每月总共挣多少钱（包括工资、奖金和补贴）？ _____ Yuan 元
[IWER: Mark 0 if there is no net income, and mark 999997 if running a deficit 访员注意：如果没有净收入请标注 0，如果是亏损请标注 999997] [Soft Check: Soft Check: Prompt for Verification if FL009 < 100]

FL010 [IWER: If R is unwilling to answer or does not remember, ask unfolding bracket questions here 访员注意：如果受访者不愿回答或者忘记了，在此处分级展开提问] 500 /1,000 /2,500 /5,000 /10,000 yuan 元

FL011 What was the value of other bonuses not paid with regular wages each year? 您从单位拿到的其他所有奖金（不和薪酬一起按期支付，比如年终奖等）每年一共有多少钱？ _____ Yuan 元

FL011_bracket [IWER: If R is unwilling to answer or does not remember, ask unfolding bracket questions here 访员注意：如果受访者不愿回答或者忘记了，在此处分级展开提问] 500 /1,000 /2,500 /5,000 /10,000 yuan 元

**PROCEDURE 程序:**
Ask FL012 if self-employed (FL001 = 2). 如果是自雇（FL001 = 2），问 FL012
Otherwise skip to FL013. 否则跳至FL013

FL012 Do you have employees? 您有雇员吗？
1. Self-employed with employees 有
2. Self-employed without employees 没有
3. Family business worker without pay 家里人一起做，不付工资

PROCEDURE 程序:

Ask FL013–FL017 only if employed (FL001 = 1). 对于问题FL013–FL017，只向受雇人群提问（FL001 = 1）

Otherwise skip to FL020. 否则跳至 FL020

FL013 Were you a regular worker, a temporary worker, or a casual worker? 您是正式员工、合同制员工、临时工，还是小时工？

1. Regular wage worker 正式员工
2. Contract worker 合同制员工
3. Temporary wage worker 临时工
4. Casual wage worker 小时工

FL014 Did you work for the government, institution, firm, NGO, individual farmer or a resident household? 您是为政府机构、事业单位、企业单位、个体户、非赢利机构（如社团、协会、学会等）、农户，还是居民户工作？

1. Government 政府部门
2. Institutions 事业单位 → Skip to FL017 跳至 FL017
3. NGO 非营利机构（如社团、协会、学会等）→ Skip to FL017 跳至 FL017
4. Firm 企业 Skip to → FL016 跳至FL016
5. Individual firm 个体户 → Skip to FL017 跳至 FL017
6. Individual farmer 农户 → Skip to FL017 跳至 FL017
7. Individual household 居民户 → Skip to FL017 跳至 FL017
8. Other, _____ (**FL014_1**) 其他，请注明 _____ (**FL014_1**) → Skip to FL017 跳至 FL017

PROCEDURE 程序:

If government employee (FL014= 1), ask FL015. 如果是政府雇员，问FL015

FL015 Were you a civil servant? 您是公务员吗？

1. Yes 是
2. No 否

PROCEDURE 程序:

If firm (FL014= 4), ask FL016. 如果在企业工作 (FL014= 4)，问FL016

Otherwise skip to FL017. 否则跳至FL017

FL016 What was the ownership type of the business? 您的企业的所有制类型是？

1. 100% State owned firm 国有企业
2. State-controlled firm 国有控股企业
3. 100% Collective-owned firm 集体所有制企业
4. Collective-controlled firm 集体控股企业
5. 100% Private firm 私营/个体
6. Private-controlled firm 私人控股企业

7. 100% foreign-owned 外商独资
8. Joint venture 中外合资
9. Other joint-ownership 其他联营企业
10. Other, _____ (**FL016_1**) 其他，请注明 _____ (**FL016_1**)

FL017 What sort of work did you do? 您自己具体从事什么工作？_____ (**FL017_1**)（例 1：餐厅面点厨师；例 2：生产线绕线工人）是否有相关的职业资格证书？_____ (**FL017_2**)（例 1：有高级中式烹调师证；例 2：没有职业资格证书）
[IWER: Ask the specific work 访员注意：请详细填写具体工作内容。注意：回答中请不要用逗号]

FL020 Why did you leave that employer? 您为什么离开这个工作单位？或者您为什么不继续您的生意或务农了，或者您为什么不再为家庭经营活动帮工了呢？
[IWER: Do not probe but check all that apply 访员注意：不用深究，请选择全部适用项]
1. Business closed 单位破产／倒闭了 (**FL020s1**)
2. Quit 辞职 (**FL020s2**)
3. I was laid off 下岗 (**FL020s3**)
4. I was fired 被辞退 (**FL020s4**)
5. I went to school 我上学了 (**FL020s5**)
6. I went abroad 我出国了 (**FL020s6**)
7. I stopped working for health reasons 因为健康原因中断工作 (**FL020s7**)
8. I stopped working for family reasons 因为家庭原因中断工作 (**FL020s8**)
9. I was transferred to another job 工作调动 (**FL020s9**)
10. I was sent down to the countryside to do manual labor 上山下乡 (**FL020s10**)
11. I started working off-farm locally 我在本地从事非农工作了 (**FL020s11**)
12. I went to work away from home 我外出打工了 (**FL020s12**)
13. Better job in local area 本地更好的新工作 (**FL020s13**)
14. Better job in another location 外地更好的新工作 (**FL020s14**)
15. I retired 我退休了 (**FL020s15**)
16. Other, _____ (**FL020_1**) 其他，请注明 _____ (**FL020_1**) (**FL020s16**)

FL021 Did you receive any payments other than the legal retirement allowance upon leaving your last job? (For example, condolence payment，workers compensation, etc.) 上一份工作结束时，除去退休工资，您是否还得到了其他补偿（如买断工龄，伤残津贴等）？
1. Yes 是
2. No 否

[访员注意：“买断工龄”指改革开放初期我国一些国有企业在改革过程中安置富余人员的一种办法，即参照员工在企业的工作年限、工资水平、工作岗位等条件，结合企业的实际情况，经企业与员工双方协商，报有关部门批准，由企业一次性支付给员工一定数额的货币，从而解除企业和富余员工之间的劳动关系，把员工推向社会的一种形式]

PROCEDURE 程序:
If FL021 = 1, ask FL022. 如果 FL021= 1，问 FL022

FL022 How much was the compensation and for how many years of work? _____ Yuan 元 _____ years 您得到了多少补偿? _____ (**FL022_1**) 元；是按照多少工龄算的？_____ 0...120 (**FL022_2**) 年

FL022_bracket [IWER: If R is unwilling to answer or does not remember, ask unfolding bracket questions here 访员注意：如果受访者不愿回答或者忘记了，在此处分级展开提问] 1000 /2000 /5000 /10,000 /20,000 yuan 元

FM RETIREMENT 退休与退职

FB011 BRANCHPOINT:

If old R had completed retirement procedure (including early retirement) or internal retirement (ZF15= 1), skip FB011 and FB012 如果老受访者已经办理了退休（包括提前退休）或者内退（ZF15= 1），跳过FB011 和FB012

NEWIW: Ask FB011

FB011 Have you completed retirement procedures （including early retirement）or internal retirement（Note: Retirement from government departments, enterprises and institutions, not including retirement in the sense of getting agricultural insurance）？ 您是否办理了退休手续（包括提前退休）或内退（注意：退休指从机关、事业单位、企业的退休，不包括获取农保的退休）？

1. Yes 是
2. No 否

FB012 Have you completed receding position procedures？您是否办理了退职手续？

1. Yes 是
2. No 否

[访员注意："退职"指职工因病残完全丧失劳动能力，但在年龄、工龄或个人缴费年限方面又不具备退休条件的，经医院证明并经劳动鉴定委员会确认、组织批准后退出生产或工作岗位，并按国家有关规定给予一定的物质帮助和被补偿，进行休养。或者是已到退休年龄，但工龄不够]

PROCEDURE 程序:

FB011 = 2 and FB012 = 2, skip to FN002_w3 跳至 FN002_w3

PROCEDURE 程序:

If R has processed retirement [FB011 = 1 or FB012 = 1 or ZF15 = 1 or ZF16 = 1], ask FM001 -FM059 . 如果受访者已经办了退休手续 [FB011 = 1 或ZF15 = 1] 或者退职手续 [FB012 = 1 或 ZF16 = 1]，提示受访者，您刚才告诉我们您已经办理了退休/退职手续，下面是关于您的退休/退职手续的一些问题，并提问FM001 -FM059

FM001_1 BRANCHPOINT：

如果老受访者上一轮调查时已经办理了退职（ZF16= 1），依次检查ZF25_1 ZF25_2 ZF25_3，如果ZF25_1= 1，则询问FM004，如果ZF25_2= 1，则询问FM005，如果ZF25_3= 1，则询问FM007。之后请跳至FM001_2 BRANCHPOINT

如果老受访者上一轮调查时已经办理了 [正式/提前] 退休（ZF25_4= 1），依次检查ZF25_1 ZF25_5 ZF25_6，如果ZF25_1= 1，则询问FM004，如果ZF25_5= 1，则询问FM014，如果ZF25_6= 1，则询问FM016-FM017。之后请跳至FM001_2 BRANCHPOINT

如果老受访者上一轮调查时已经办理了内退（ZF25_8= 1），依次检查ZF25_1 ZF25_9 ZF25_10 ZF25_11，如果ZF25_1= 1，则询问FM004，如果ZF25_9= 1，则询问FM025，如果ZF25_10= 1，则询问FM027，如果ZF25_11= 1，则询问FM028-FM029。之后请跳至FM001_2 BRANCHPOINT

FM001_2 BRANCHPOINT：

If old R had completed receding position procedure prior to the last IW (ZF16= 1), or had completed regular retirement in the last IW (ZF16= 1), skip to FM052 BRANCHPOINT. 如果老受访者上一轮调查时已经办理了退职，或上一轮调查时已经办理了正式退休，跳至FM052 BRANCHPOINT

If old R had completed internal retirement but not yet regular retirement prior to the last IW （ZF23= 1）, skip to FM037_w2 BRANCHPOINT. 如果老受访者上一轮调查时已经办理了内退但是还没有办理正式退休，跳至FM037_w2 BRANCHPOINT

If old R had not completed retirment procedure or receding position procedure prior to the last IW (ZF24= 1), continue with FM001 . 如果老受访者上一轮调查时还没有办理退休或退职手续，询问FM001

All new R continues with FM001 所有的新受访者，询问FM001

FM001 Which of the following is the work unit that processed your [preload: retirement /receding position] 给您办理 [退休/退职] 手续的单位是下面的哪一个？

[IWER: If R has no work unit (no answer to FD003 or FL027), please choose "(3) None of the above" 访员注意：如果此受访者没有工作单位，请选择"(3) 以上都不是"，并继续]

1. Current work unit 现在的工作单位 [for new R: preload FD003; for old R: ZF8 if FA006_w2_2a= 1 & FA006_w2_2a_3= 1, or FA006_w2_2a_1= 1 if FA006_w2_2a= 2 & FA006_w2_2a_3= 1, or FM003 if FA006_w2_2a_3= 2] → Skip to procedure before FM005 请跳至FM005 前的程序控制
2. Last work unit [preload FL005 or ZF25] FL005 中提到的上一份工作的单位 [下载FL005 or ZF25] → Skip to procedure before FM005 请跳至FM005 前的程序控制
3. None of the above 以上都不是

PROCEDURE 程序:

If answer to FM001is "(3) None of the above", then ask FM002-FM004 : 如果 FM001回答"(3) 以上都不是"，问FM002-FM004

FM002 What is the name of the employer that processed your [preload: retirement /receding position]? 给您办理 [退休/退职] 手续的单位的名称是什么？ _____

FM003 What was the type of your work unit at [preload: retirement /receding position]? 您办理 [退休/退职] 的单位是哪种类型的？

1. Government 政府部门
2. Institutions 事业单位
3. NGO 非营利机构（如社团、协会、学会等）
4. Firm 企业
5. Individual firm 个体户
6. Farmer 农户
7. Individual household 居民户
8. Other 其他

FM004 Where is this work unit located? 这个单位的地址是？

1. The same as permanent address 和目前常住地一样
2. Another village/neighborhood in permanent address's county/city/district 常住地所在县/市/区的其他 _____ (**FM004_1**) 乡/镇/街道/村/社区
3. Other 其它:_____ (**FM004_2**) province_city_county/city/district 省 _ 市 _ 县/市/区, _____ (**FM004_3**) 乡/镇/街道/村/社区
4. Abroad 国外

[访员注意：如果记不清详细的地址名称，请在相应输入框中填入“其它”]

PROCEDURE 程序:
If FB012= 1 (Receding), ask FM005-FM009 如果FB012= 1（受访者退职），询问 FM005-FM009

FM005 In what month and year did you recede from your position? 您是在哪年哪月办理的退职？ _____ (**FM005_1**) 1900...2015 year 年 _____ (**FM005_2**) 0...12 month 月
[IWER: Mark the year using four digits. Take down the month as its actual number. For example, write January as “1” not “01”, December as “12”. If do not remember month, fill ‘0’ 访员注意: 用 4 位数表示年，按照实际的月份填写月。例：1 月写作“1”，而不是“01”,12 月写作“12”。如果记不住月份，请填入‘0’]

FM006 What was the main reason you receded from your position? 您办退职的主要原因是？

1. Due to poor health, I couldn't continue my work any more, at the same time I wasn't eligible for retirement 身体原因不能继续工作，而又不符合退休条件
2. Years of eligible work are less than three, and time of stopping work due to diseases or injures not related to work are more than one year 连续工龄不满三年，因病或非因工负伤而停止工作的时间满一年
3. I'm recruit worker within 6 months, but I had serious chronic disease once and can't tstick to work any more 录用后在六个月以内，发现原来有严重慢性疾病，不能坚持工作的
4. I receded from my position voluntary 自愿退职
5. Reach retirement age, but not eligible working age. 到了退休年龄，但工龄不够
6. Other 其他

FM007 Your pre-receding total salary was _____ Yuan a month (including basic wage, bonus, et. al). 您退职前的总工资是每月多少钱? _____ 元/月, 包括基本工资和奖金等。

FM007_bracket [IWER: If R is unwilling to answer or does not remember, ask unfolding bracket questions here 访员注意: 如果受访者不愿回答或者忘记了, 在此处分级展开提问]
500 /1,000 /2,000 /3,500 /5,000 yuan 元

FM008 Did you receive any payments for leaving your job? 您是否得到了退职补偿?

1. Yes 是
2. No 否

PROCEDURE 程序:
If FM008 = 1, ask FM009. 如果 FM008 = 1, 问 FM009

FM009 How much was the compensation? _____ Yuan 元您得到了多少补偿? _____ 元;

FM009_bracket [IWER: If R is unwilling to answer or does not remember, ask unfolding bracket questions here 访员注意: 如果受访者不愿回答或者忘记了, 在此处分级展开提问]
500 /1,000 /2,000 /3,500 /5,000 yuan 元

PROCEDURE 程序:
If FB011 = 1 (Retirement), ask FM011-FM041. 如果 FB011= 1(受访者退休), 询问FM011-FM041
Otherwise skip to FM042, 否则跳至FM042

FM011 Was your retirement normal retirement; early retirement; or internal retirement initially, followed by regular retirement? 您办的是正常退休, 提前退休还是先内退然后办的正式退休或者将来再办正式退休?

1. Normal retirement 正常退休
2. Early retirement 提前退休
3. Internal retirement first, then regular retirement 先内退然后又正式退休
4. Internal retirement, but not yet regular retirement 内退但目前还没有办理正式退休

FM012 Did you retire as a worker or as a cadre? 您退休时的身份是干部还是工人?

1. Worker 工人
2. Cadre 干部

PROCEDURE 程序:
If FM011 = 1 or FM011 = 2 , ask FM014-FM017. 如果 FM011 = 1/2, 问 FM014-FM017

FM014 In what month and year did you take [preload: normal/early] retirement? 您办理[正式/提前] 退休手续是在哪年哪月? _____ 1900...2015 (**FM014_1**) year 年 _____ 0...12 (**FM014_2**) month 月
[IWER: Mark the year using four digits. Take down the month as its actual number.

For example, write January as “1” not “01”, December as “12”. If do not remember month, fill ‘0’ 访员注意: 用 4 位数表示年，按照实际的月份填写月。例：1 月写作“1”，而不是“01”,12 月写作“12”。如果记不住月份，请填入‘0’]
[Soft Check: Prompt for Verification/Correction if Age of Early Retirement is Young, e.g. Prompt if ((FM014_1 +FM014_2 /12)-(CV009_a+CV009_b/12) < 45 & CV004==2) | ((FM014_1 +FM014_1 /12)-(CV009_a+CV009_b/12) <50 & CV004== 1)]

PROCEDURE 程序:

Ask FM015 if FM011 = 2 (early retirement). 如果提前退休，问FM015

FM015 What was the main reason you processed early retirement? 您办提前退休的主要原因是？

1. I have 30 years job experience, which is enough for early retirement. 我的工龄达到了 30 年，可以提前退休。
2. My work unit belonged to the category of high-risk and hard manual labor and thus was eligible for offering early retirement 单位是高风险、高强度的工作，允许提前退休
3. My work unit was restructuring/bankrupt, so it offered early retirement 我的工作单位要破产、已经破产或者正在重组因此允许提前退休
4. Due to poor health 健康原因
5. Due to family reason 家庭原因
6. Other 其他

FM016 Your pre-retirement salary was _____ Yuan a month (including bonus and subsidy, et. al). 您退休前的工资，包括奖金和各种补贴每月多少钱？_____ 元/月。

FM017 [IWER: If R is unwilling to answer or does not remember, ask unfolding bracket questions here 访员注意：如果受访者不愿回答或者忘记了，在此处分级展开提问] 500 /1,000 /2,500 /5,000 /10,000 yuan 元

PROCEDURE 程序:

Ask FM025-FM029 if FM011 = 3or FM011 = 4 (internal retirement) 如果 FM011=3/4, 如果内退，问FM025-FM029
Otherwise skip to FM042, 否则跳至 FM042

FM025 In what month and year did you take internal retirement? 您是在哪年哪月办理的内退？_____ 1900...2015 (**FM025_1**) year 年 _____ 0...12 (**FM025_2**) month 月
[IWER: Mark the year using four digits. Take down the month as its actual number. For example, write January as “1” not “01”, December as “12”. If do not remember month, fill ‘0’ 访员注意: 用 4 位数表示年，按照实际的月份填写月。例：1 月写作“1”，而不是“01”,12 月写作“12”。如果记不住月份，请填入’0’]
[Soft Check: Prompt for Verification/Correction if Age of Retirement is Young, e.g. Prompt if (FM030_1 +FM030_2 /12)-(CV009_a+CV009_b/12) <45 & CV004==2) | ((FM030_1 + FM030_2 /12)-(CV009_a+CV009_b/12) <50 & CV004==1)]

FM026 What was the main reason you processed internal retirement? 您办理内退的主要原因是？

1. 5 years less than the legal retirement age 距法定退休年龄不足 5 年。
2. My work unit was restructuring/bankrupt 我的工作单位要破产、已经破产或者正在重组
3. Due to poor health 健康原因
4. Due to family reason 家庭原因
5. Other 其他

FM027 Your pre-internal retirement salary was _____ Yuan a month everything included. 您内退前的工资、奖金全部加起来是平均每月多少钱? _____ 元/月。

FM027_bracket [IWER: If R is unwilling to answer or does not remember, ask unfolding bracket questions here 访员注意：如果受访者不愿回答或者忘记了，在此处分级展开提问] 500 /1,000 /2,000 /3,500 /5,000 yuan 元

FM028 How much was the internal retirement wage (everything included) when you processed internal retirement? 您内退的时候，工资、奖金全部加起来每个月平均能拿到多少？_____ 元/月。

FM029 [IWER: If R is unwilling to answer or does not remember, ask unfolding bracket questions here 访员注意：如果受访者不愿回答或者忘记了，在此处分级展开提问] 500 /1,000 /2,000 /3,500 /5,000 yuan 元

PROCEDURE 程序:
Ask FM030–FM036 if FM011 = 3 如果 FM011= 3，问FM030–FM036

FM030 In what month and year did you process formal retirement? 您在哪年哪月办理的正式退休手续？ _____ 1900...2015 (**FM030_1**) year 年 _____ 0...12 (**FM030_2**) month 月
[IWER: Mark the year using four digits. Take down the month as its actual number. For example, write January as "1" not "01", December as "12". If do not remember month, fill '0' 访员注意: 用 4 位数表示年，按照实际的月份填写月。例：1 月写作"1"，而不是"01" ,12 月写作"12"。如果记不住月份，请填入'0']
[Soft Check: Prompt for Verification/Correction if Age of Retirement is Young, e.g. Prompt if ((FM030_1 +FM030_2 /12)-(CV009_a+CV009_b/12) <45 & CV004==2) | ((FM030_1 +FM030_2 /12)-(CV009_a+CV009_b/12) <50 & CV004==1)]

FM036 How many years of eligible work did you have at the time of formal retirement? 您正式退休时计算了多少年工龄/社会保险缴费年限或视同缴费年限？ _____ 0.00...100.00 Years 年

PROCEDURE 程序:
Skip to FM042 跳至FM042

FM037_w2 BRANCHPOINT :

If R had completed internal retirement but not yet regular retirement prio to the last IW (ZF23= 1), then ask FM037_w2- FM040_w2. 如果上一轮调查时受访者已经办理了内退但是还没有办理正式退休，询问 FM037_w2至FM040_w2

FM037_w2 Did you proceed formal retirement? 您是否已经办理正式退休手续?

1. Yes 是
2. No 否 → Skip to FM037 跳至FM037

FM038_w2 In what month and year did you process formal retirement? 您在哪年哪月办理的正式退休手续? _____ 1900...2015 (**FM038_w2_1**) year 年 _____ 0...12 (**FM038_w2_2**) month 月

[IWER: Mark the year using four digits. Take down the month as its actual number. For example, write January as "1" not "01", December as "12". If do not remember month, fill '0' 访员注意: 用 4 位数表示年，按照实际的月份填写月。例: 1 月写作 "1"，而不是 "01" ,12 月写作 "12"。如果记不住月份，请填入' 0']

[Soft Check: Prompt for Verification/Correction if Age of Retirement is Young, e.g. Prompt if ((FM030_1 +FM030_2 /12)-(CV009_a+CV009_b/12) <45 & CV004==2) | ((FM030_1 +FM030_2 /12)-(CV009_a+CV009_b/12) <50 & CV004==1)]

FM040_w2 How many years of eligible work did you have at the time of formal retirement? 您正式退休时计算了多少年工龄/社会保险缴费年限或视同缴费年限? _____ 0.00...100.00 Years 年

PROCEDURE 程序:

Ask FM037-FM040 if FM011 = 4 or FM037_w2 = 2. 如果FM011 = 4 或者 FM037_w2 = 2, 问FM037-FM040

Otherwise Skip to FM042. 否则跳至FM042

FM037 In what month and year are you going to process formal retirement? 您将会在哪年哪月办理正式退休手续? _____ 1900...2015 (**FM037_1**) year 年 _____ 0...12 (**FM030_2**) month 月

[IWER: Mark the year using four digits. Take down the month as its actual number. For example, write January as "1" not "01", December as "12". If do not remember month, fill '0' 访员注意: 用 4 位数表示年，按照实际的月份填写月。例: 1 月写作 "1"，而不是 "01" ,12 月写作 "12"。如果记不住月份，请填入' 0']

[Soft Check: Verify if Age of Respondent will be outside the legal retirement range, e.g., Prompt for verification if ((FM037_1 + FM037_2/12) - (CV009_a + CV009_b/12) < 50 | (FM037_1 + FM037_2/12) - (CV009_a + CV009_b/12) > 55) & CV004 == 2) | (FM037_1 + FM037_2 /12) - (CV009_a + CV009_b/12) < 55 | (FM037_1 + FM037_2/12) - (CV009_a + CV009_b/12) > 60) & CV004 ==1)]

FM040 How many years of eligible work will you have at the time of retirement? 您正式退休时预计将计算多少年工龄/社会保险缴费年限或视同缴费年限? _____ 0.00...100.00 Years 年

FM041 How many years of eligible work do you currently have? 您现在已经有多少年工龄了/社会保险缴费年限或视同缴费年限? _____ 0.00...100.00 Years 年 [FM040 > FM041]

**PROCEDURE 程序:**
Skip (FM042-FM051) if FM037_w2 = 2. 如果FM037_w2 = 2，跳过FM042-FM051

FM042 Did you have a spouse when you processed [preload: normal retirement / early retirement / internal retirement / receding position]? 您办理手续时是否有配偶? [正式退休/提前退休/内退/退职 (注：从FM011处加载。如果 FM037_w2 = 1，则此处加载“正式退休”。下同)]

1. Yes 是
2. No 否

FM043 How was your health at the time of your [preload: normal retirement / early retirement / internal retirement / receding position], excellent, very good, good, fair or poor? 您 [正式退休/提前退休/内退/退职] 时您的身体状况如何? 是极好、很好、好、一般还是不好?

1. Excellent 极好
2. Very good 很好
3. Good 好
4. Fair 一般
5. Poor 不好

**PROCEDURE 程序:**
Skip FM044-FM046 if FM042 = 2. 如果 FM042 = 2，跳过 FM044-FM046

FM044 Had your spouse already processed retirement when you processed [Preload: normal retirement / early retirement / internal retirement / receding position]? 您办理 [正式退休/提前退休/内退/退职] 时配偶是否已经办理了退休手续?

1. Yes 是
2. No 否

FM045 What kind of economic activities was your spouse engaged in at the time of your [preload: retirement / receding position]? 您 [退休/退职] 时配偶在从事哪种经济活动?

1. Employed by another person or company and received a wage 受雇于某公司或个人，领取工资
2. Ran own business 从事个体或者私营经济活动
3. Non-employed and looking for a job 没有工作但是在找工作
4. Non-employed and not looking for a job，or only doing household work 没有工作也没有找工作，或仅仅在家作家务
5. Farming 务农

FM046 How was your spouse's health at the time of your [preload: normal retirement / early retirement / internal retirement / receding position], excellent, very good, good, fair or poor? 您 [正式退休/提前退休/内退/退职] 时您配偶的身体状况如何? 是极好、很好、好、一般还是不好?

1. Excellent 极好
2. Very good 很好
3. Good 好
4. Fair 一般
5. Poor 不好

FM047 Was your father alive at the time of your [preload: normal retirement / early retirement / internal retirement / receding position]? 您 [正式退休/提前退休/内退/退职] 时，您父亲还健在吗？

1. Yes 是
2. No 否 → Skip to FM049 跳至FM049

FM048 How about the health of your father at the time of your [preload: normal retirement / early retirement / internal retirement / receding position], excellent, very good, good, fair or poor? 您 [正式退休/提前退休/内退/退职] 时您父亲的身体状况如何？是极好、很好、好、一般还是不好?

1. Excellent 极好
2. Very good 很好
3. Good 好
4. Fair 一般
5. Poor 不好

FM049 Did your mother alive when your [preload: normal retirement / early retirement / internal retirement / receding position]? 您 [正式退休/提前退休/内退/退职] 时，您母亲还健在吗？

1. Yes 是
2. No 否 → Skip to FM051 跳至FM051

FM050 How about the health of your mother at the time of your [preload: normal retirement / early retirement / internal retirement / receding position], excellent, very good, good, fair or poor? 您 [正式退休/提前退休/内退/退职] 时您母亲的身体状况如何？是极好、很好、好、一般还是不好?

1. Excellent 极好
2. Very good 很好
3. Good 好
4. Fair 一般
5. Poor 不好

FM051 How many grandchildren below age 6 did you have at the time of your [preload: normal retirement / early retirement / internal retirement / receding position]? 您 [正式退休/提前退休/内退/退职] 时有几个六岁以下的孙子女或者外孙子女？_____ 0...50 persons 人

[IWER: If none, fill ‘0’ 访员注意：如果没有请填“0”]

FM052 BRANCHPOINT:

For old R who had processed receding position procedure in the last IW (ZF16 = 1) or had processed internal but not regular retirement in the last IW (ZF23 = 1), or had processed regular retirement in the last IW (ZF26 = 1): if old R had not worked after processing retirement/receding position procedure until the last IW (ZF26 = 1) and is currently not working (XF1 = 2), ask FM052_w2; if old R had not worked after processing retirement/receding position procedure until the last IW (ZF27 = 1) and is currently working (XF1 = 1), ask FM053 上一轮时已经办理退职、或者已经办理内退但是还没有办理正式退休、或者已经办理正式退休手续的老受访者：如果老受访者办理退休/退职后到上一轮调查时没有工作过并且现在不工作，询问FM052_w2；如果老受访者办理退休/退职后到上一轮调查时没有工作过但是现在在工作，询问FM053。其他老受访者，询问FM052_w2

New R 新受访者：If R is currently not working (FA001 = 2 & FA002 = 2 & FA003 = 2), ask FM052. 如果受访者现在不在工作（FA001 = 2 & FA002 = 2 & FA003 = 2），问：FM052

If FA052 = 1 or If R is currently working, ask FM053. 如果受访者退休后工作过或者现在仍在工作（FA052 = 1 or FA001 = 1 or FA002 = 1 or FA003 = 1），跳至FM053

FM052 Did you work after you processed [preload: normal retirement / early retirement / internal retirement / receding position]? We consider any of the following activities to be work: agricultural work, earn a wage, run your own business and unpaid family business work, et. al. Work does not include doing your own housework or doing activities without pay, such as voluntary work. 在您办理了 [正式退休/提前退休/内退/退职] 之后，您是否工作过？务农、挣工资工作、从事个体、私营经济活动或不拿工资为家庭经营活动帮工都算是工作，但不包括家务劳动、义务的志愿劳动。

1. Yes 是 → Skip to FM053 跳至 FM053
2. No 否 → Skip to FM054 跳至FM054

FM052_w2 Did you work in the last two years? We consider any of the following activities to be work: agricultural work, earn a wage, run your own business and unpaid family business work, et.al. Work does not include doing your own householdwork or doing activities without pay, such as voluntary work. 过去两年您工作过吗？挣工资工作、从事个体、私营经济活动或不拿工资为家庭经营活动帮工都算是工作，但不包括家务劳动、义务的志愿劳动

1. Yes 是
2. No 否 → Skip to FM054 跳至FM054

FM053 After you processed [preload: normal retirement / early retirement / internal retirement / receding position] How long did you start to work again? 在您办理了 [正式退休/提前退休/内退/退职] 之后，您过了多长时间又开始工作的？_____ 0.00...100.00 Years 年 (allow for decimal points 允许小数点）

PROCEDURE 程序:

Skip to FN002_w3 跳至FN002_w3

FM054 Are you currently engaged in paid small pastime work? 您目前是否从事小的消遣性的工作但是也有收入?

1. Yes 是
2. No 否 → Skip to FN002_w3 跳至 FN002_w3

FM055 What kind of pastime job are you engaged in? 您从事哪种消遣性的工作? _____ [IWER: Ask the specific work 访员注意：请详细填写具体工作内容。注意：回答中请不要用逗号]

FM056 When did you start this job? 您从什么时候开始做这项工作的? _____ 1900...2015 (**FM056_1**) year 年 _____ 0...12 (**FM056_2**) month 月

[IWER: Mark the year using four digits. Take down the month as its actual number. For example, write January as "1" not "01", December as "12". If do not remember month, fill '0' 访员注意：用 4 位数表示年，按照实际的月份填写月。例：1 月写作"1"，而不是"01"，12 月写作"12"。如果记不住月份，请填入'0']

FM057 How many days per week do you usually work for your pastime job? 您一般每周做几天? An average of _____ days per week 平均每周 _____ 0...7 天

FM058 How many hours per week do you usually work at your pastime job? 您一般每周做几个小时? An average of _____ hours per week 平均每周 _____ 0.00...168.00 小时

FM059 What is your monthly income from the pastime work? 您每月能挣多少钱? _____ YUAN 元

[IWER: Mark 0 if there is no net income, and mark 999997 if running a deficit 访员注意：如果没有净收入填 0，如果每月亏损填 999997]

FM059_bracket [IWER: If R is unwilling to answer or does not remember, ask unfolding bracket questions here 访员注意：如果受访者不愿回答或者忘记了，在此处分级展开提问]

500 /1,000 /2,000 /3,500 /5,000 yuan 元

FN PENSION INSURANCE 养老保险

[Intro: Next we' ll ask you some questions about your pension insurance. It' s important to assess existing pension policy and revise it in the future 引语：下面我们将问一些有关您的养老保险的问题，这些问题对于评估现有的养老政策以及制定将来的养老政策有着重要意义] [F1:(1)"政府机关和事业单位退休金"指受访者从政府和事业单位领取的退休金。目前，我国大部分政府机构和事业单位，都还没有实行社会养老保险制度，仍实行退休养老制度。这一制度下，一般不需要个人在退休前缴纳养老保险金。（2）"企业职工基本养老保险"是指受访者参加社会基本养老保险制度，达到退休年龄并符合领取条件后，领取到社会养老保险金。（3）"企业补充养老保险"也叫企业年金，是指企业及其职工在依法参加基本养老保险的基础上，自愿建立的补充养老保险制度。（4）"商业养老保险"是指受访者直接向商业保险公司投保，定期缴纳保险费，从合同约定年龄开始持续、定期地领取养老金的人寿保险，能有效地满足客户的养老需要。（6）"城乡居民社会养老保险"指为推进城乡一体化，某些地区将城镇居民社会养老保险和新型农村社会养老保险统筹安排、合并实施，称为"城乡居民社会养

老保障制度”。（7）“城镇居民养老保险”指通过建立城镇居民养老保险个人账户，实行个人缴费和财政补贴相结合的筹资模式，为目前未享受定期养老待遇或其他相关定期养老待遇的城镇居民提供的养老保险。（8）“新型农村社会养老保险”（新农保）采取个人缴费、集体补助和政府补贴相结合，其中中央财政将对地方进行补助，并且会直接补贴到农民头上。（9）“高龄老人养老补助（补贴）”是指 60 岁以上老人享有政策性的优惠，如乘车免费、挂号费免费、参观等免费、部分社区活动免费，如注射流感疫苗等，百岁以上老人才享有月补助金。]

Part 1 政府机关、事业单位养老保险（退休金）及企业职工基本养老保险

FN002_w3 您是否参加了政府机关、事业单位或是企业职工基本养老保险？

1. Yes, I receive it 参加了，现在在领取
2. Yes, but I don't receive it 参加了，还未领取 → Skip to FN006_w2 跳至 FN006_w2
3. No 没有参加 → Skip to FN030_w2 跳至FN030_w2

FN002_w2 Are you currently receiving pension as follows (Check all that apply)? 您现在是否领取以下退休金/养老保险？(可多选)

1. 政府机关（公务员）退休金
2. 事业编制职工退休金
3. 企业职工基本养老保险

**PROCEDURE 程序**:
如果FN002_w2多选，则针对每一个选项，循环询问FN003_w2-FN005_w2_bracket

FN003_w2 In what month and year did you start to receive you pension benefits? 您从什么哪年哪月开始领取这一养老金 (退休金) 的？_____ 1900...2015 (**FN003_w2_1**) year 年 _____ 0...12 (**FN003_w2_2**) month 月

[IWER: Mark the year using four digits. Take down the month as its actual number. For example, write January as "1" not "01", December as "12". If do not remember month, fill '0' 访员注意：用 4 位数表示年，按照实际的月份填写月。例：1 月写作“1”，而不是“01”，12 月写作“12”。如果记不住月份，请填入’0’]

FN004_w2 How much were the benefits (including subsidy) when you retired? 您办理正式退休的时候，退休金/养老金 (包括各种补贴) 是多少？_____ Yuan per month 元/月。

FN004_w2_bracket [IWER: If R is unwilling to answer or does not remember, ask unfolding bracket questions here 访员注意：如果受访者不愿回答或者忘记了，在此处分级展开提问]
1000 /2,000 /3,000 /5,000 /10,000 yuan 元

FN005_w2 What is your monthly pension (including subsidy)? 您现在每月领多少退休金/养老金 (包括各种补贴)？_____ Yuan 元

[Soft Check: Verify if monthly benefits are low or high, e.g., prompt if FN005_w2 <200 yuan/month]

FN005_w2_bracket [IWER: If R is unwilling to answer or does not remember, ask unfolding bracket questions here 访员注意：如果受访者不愿回答或者忘记了，在此处分级展开提问]
1000 /2,000 /3,000 /5,000 /10,000 yuan 元

**PROCEDURE 程序**:
Skip to FN030_w2 跳至FN030_w2

FN006_w2 Are you currently enrolled in pension program of the government and institutions or basic pension of the firms? 您将来是否能够享受政府和事业单位的退休金待遇或是参加了企业职工的养老保险？

1. Yes, pension program of the government and institutions 是的，将来享受政府机关和事业单位的退休金待遇
2. Yes, basic pension insurance of the firms 是的，目前参加了企业职工基本养老保险
3. Yes, both of them. 是的，两个都在参加/能够享受。
4. No 否，不能享受 → Skip to FN022_w3 跳至 FN022_w3

**PROCEDURE 程序**:
如果FN006_w2 = 3，循环询问FN007_w2–FN014_w2_bracket

FN007_w2 From which of the following work units did you get the pension insurance you just told us? 将来给您提供的单位是下面的哪一个？
[Preload "pension program of government and institutions" if FN006_w2 = 1; or preload "Basic pension insurance of the firms" if FN006_w2 = 2] [IWER: Choose "(4) None of the above" if the respondent has no work unit 访员注意：如果受访者没有单位，请选择"(4) 都不是"并继续]

1. Current work unit 现在的工作单位 [for new R: preload FD003; for old R: ZF8 if FA006_w2_2a = 1 & FA006_w2_2a_3= 1, or FA006_w2_2a_1 if FA006_w2_2a= 2 & FA006_w2_3= 1, or FD003 if FA006_w2_2a_3= 2] → Skip to FN011_w2 跳至 FN011_w2
2. Last work unit [preload FL005 or ZF25] FL005中提到的上一份工作的单位 [下载FL005 or ZF25] → Skip to FN011_w2 跳至 FN011_w2
3. The work unit that processed retirement for the respondent [preload FM002] FM002 中提到的办理退休手续的单位 [下载FM002] → Skip to FN011_w2 跳至 FN011_w2
4. None of the above 都不是

**PROCEDURE 程序**:
If answer to FN007_w2 = 4 (None of the above), then ask FN008_w2 –FN010_w2. 如果FN007_w2 = 4 (回答"都不是")，请问 FN008_w2 -FN010_w2

FN008_w2 What is the name of the unit that provides you this pension insurance? 将来给您提供这份退休金/养老保险的单位的名称是什么？ _____

FN009_w2 What was the type of the unit that provides you the pension insurance? 这个给您提供养老保险的单位是哪种类型的？

1. Government 政府部门
2. Institutions 事业单位
3. NGO 非营利机构（如社团、协会、学会等）
4. Firm 企业
5. Individual 个体或者私营企业主
6. Other, _____ (**FN009_w2_1**) 其他，请注明 _____ (**FN009_w2_1**)

FN010_w2 Where is this work unit located? 这个给您提供养老保险的单位在哪?

1. The same as permanent address 和目前常住地一样
2. Another village/neighborhood in permanent address's county/city/district 常住地所在县/市/区的其他 _____ (**FN010_w2_1**) 乡/镇/街道, _____ (**FN010_w2_2**) village/neighborhood 村/社区
3. Other 其它: _____ (**FN010_w2_3**) province_city_county/city/district 省 _ 市 _ 县/市/区, _____ (**FN010_w2_4**) 乡/镇/街道, _____ (**FN010_w2_5**) village / neighborhood 村/社区
4. Abroad 国外

[IWER: 访员注意：如果记不清详细的地址名称，请在相应输入框中填入“其它”]

FN011_w2 In what month and year did you start to participate in the basic pension insurance of the firms through this work unit? 您是从哪年哪月开始在这个单位参加基本养老保险的? _____ 1900...2015 (**FN011_w2_1**) year 年 _____ 0...12 (**FN011_w2_2**) month 月

[IWER: Mark the year using four digits. Take down the month as its actual number. For example, write January as “1” not “01”, December as “12”. If do not remember month, fill ‘0’ 访员注意: 用 4 位数表示年，按照实际的月份填写月。例：1 月写作“1”，而不是“01”，12 月写作“12”。如果记不住月份，请填入’0’]

FN012_w2 Do you need to pay the premium by yourself or by work unit? 您参加的这份养老保险需要您个人或单位缴费吗?

1. Yes 需要
2. No 不需要 → Skip to FN017_w2 跳至 FN017_w2

[访员注意：如果是政府机关或是事业单位的退休金/养老保险，如果需要个人缴费就选 1 需要，如果不需要个人缴费就选 2 不需要，因为本轮的调查时，已经实行了政府机关、事业单位养老保险与企业职工基本养老保险并轨了，实行快的政府机关与事业单位已经需要个人缴费了，也有可能有些政府机关与事业单位还没实行，暂时还不需要个人缴费，所以请根据实际情况选择]

FN013_w2 How much is the premium you paid? 您个人现在缴纳的保险费是多少? _____ (**FN013_w2_1**) Yuan per month 元/月 Or 或 _____ 0.00...100.00 (**FN013_w2_2**) percent 工资的比例

FN014_w2 How much the premium your unit paid? 单位现在为您缴纳的保险费是多少? _____ (**FN014_w2_1**) Yuan per month 元/月 Or 或 _____ 0.00...100.00 (**FN014_w2_2**) 工资的比例

FN014_w2_bracket [IWER: If R is unwilling to answer or does not remember, ask unfolding bracket questions here 访员注意：如果受访者不愿回答或者忘记了，在此处分级展开提问] 500 /1,000 /2,000 /3,500 /5,000 yuan 元

FN017_w2 For how many years altogether have you been included in this program at local province? [Include years with other employers if the same plan.] 到现在为止您在本省/市可以用来计算养老金（退休金）的工龄/社会保险缴费年限或视同缴费年限一共有多少年？[不管雇主是否相同，只要能够累积计算工龄/社会保险缴费年限或视同缴费年限，都应算进去。] _____ 0.00...100.00 Years 年

FN017_w3 For how many years altogether have you been included in this program in other province? [Include years with other employers if the same plan.] 到现在为止您在其他省/市可以用来计算养老金（退休金）的工龄/社会保险缴费年限或视同缴费年限一共有多少年？[不管雇主是否相同，只要能够累积计算工龄/社会保险缴费年限或视同缴费年限，都应算进去。] _____ 0.00...100.00 Years 年

FN018_w2 For how many years altogether will you have been included in this program when you retire? [Include years with other employers if the same plan.] 您估计，到您退休时您可以用来计算养老金（退休金）的工龄/社会保险缴费年限或视同缴费年限一共有多少年？[不管雇主是否相同，只要能够累积计算工龄/社会保险缴费年限或视同缴费年限，都应算进去。] _____ 0.00...100.00 Years 年

FN019_w2 Will this be enough years to receive pension? 这工龄/社会保险缴费年限或视同缴费年限足够您从上述养老保险项目中领取养老金吗？

1. Yes 是 → Skip to FN021_w2 跳至 FN021_w2
2. No 否

FN020_w2 What do you plan to do? 那您计划怎么办？

1. I will pay the remaining premiums all in one payment at retirement to qualify for pension 退休的时候，我会一次性支付剩余年限的保险金，这样就能领取养老金了。
2. I will receive a one-time payment at retirement and not get pension 退休的时候，我会得到一次性的补偿，以后就没有养老金了。Skip to FN030_w2 跳至 FN030_w2
3. I will not receive pension 我将拿不到养老金。Skip to FN030_w2 跳至 FN030_w2

PROCEDURE 程序:

If R answered yes to pension [FN019_w2 = 1 or FN020_w2 = 1], ask FN021_w2 - FN021_w2_bracket, then skip to FN030_w2. 如果被访者退休的时候，能领养老金[FN019_w2= 1 or FN020_w2= 1]，请回答FN021_w2 - FN021_w2_bracket，然后跳至FN030_w2

FN021_w2 About how much do you expect your benefits to be? (as a percentage of your pay at retirement, or as an amount per month or year?) 您预期领多少钱的养老金？可以是退休前工资的百分比，也可以是每月多少钱。_____ （**FN021_w2_1**）Yuan per month 元/月 Or 或 _____ 0.00..100.00（**FN021_w2_2**）% of final pay 退休前工资

FN021_w2_bracket [IWER: If R is unwilling to answer or does not remember, ask unfolding bracket questions here 访员注意：如果受访者不愿回答或者忘记了，在此处分级展开提问] 500 /1,000 /2,000 /3,500 /5,000 yuan 元

FN022_w3 Are you ever enrolled in pension program of the government and institutions? 您刚刚说现在没有参加政府和事业单位的养老保险或者企业基本养老保险，请问您之前是否曾经参加过这类保险吗？

1. Yes, pension program of the government and institutions 是的，曾经参加过政府和事业单位的养老保险或者企业基本养老保险
2. No 否，没有参加过 Skip to FN030_w2 跳至 FN030_w2

FN023_w2 From which of the following work units did you get the pension insurance you just told us[preload “pension program of government and institutions” 您当时参加的单位是下面的哪一个？

[Preload “pension program of government and institutions” if FN022_w2 = 1; or preload “Basic pension insurance of the firms” if FN022_w2 = 2; 如果 FN022_w2 = 1, 下载“政府和事业单位养老保险”; 如果 FN022_w2 = 2, 下载“企业职工基本养老保险”]

[IWER: Choose “(4) None of the above” if the respondent has no work unit 访员注意：如果受访者曾经多次参加过上述两类保险，请其填写参保时间最长的那次时所在的单位；如果受访者没有单位，请选择“(4) 都不是”并继续]

1. Current work unit 现在的工作单位 [for new R: preload FD003; for old R: ZF8 if FA006_w2_2a = 1 & FA006_w2_3 = 1, or FA006_w2_2a_1 if FA006_w2_2a = 2 & FA006_w2_3 = 1, or FD003 if FA006_w2_2a_3 = 2] → Skip to FN027_w2 跳至FN027_w2
2. Last work unit [preload FL005 or ZF25] FL005 中提到的上一份工作的单位 [下载FL005 or ZF25] → Skip to FN027_w2 跳至FN027_w2
3. The work unit that processed retirement for the respondent [preload FM002] FM002 中提到的办理退休手续的单位 [下载FM002] → Skip to FN027_w2 跳至FN027_w2
4. None of the above 都不是

PROCEDURE 程序:

If answer to FN023_w2 = 4 (None of the above), then ask FN024_w2 -FN026_w2. 如果FN023_w2 = 4 (回答“都不是”), 请问 FN024_w2 -FN026_w2

FN024_w2 What is the name of the unit that provides you this pension insurance? 给您提供这份养老保险的单位的名称是什么? _____

FN025_w2 What was the type of the unit that provides you the pension insurance? 这个给您提供养老保险的单位是哪种类型的？

1. Government 政府部门
2. Institutions 事业单位
3. NGO 非营利机构（如社团、协会、学会等）
4. Firm 企业
5. Individual 个体或者私营企业主
6. Other, _____ (**FN025_w2_1**) 其他，请注明 _____ (**FN025_w2_1**)

FN026_w2 Where is this work unit located? 这个给您提供养老保险的单位在哪？

1. The same as permanent address 和目前常住地一样
2. Another village/neighborhood in permanent address's county/city/district 常住地所在县/市/区的其他 _____ (**FN026_w2_1**) 乡/镇/街道, _____ (**FN026_w2_2**) village/neighborhood 村/社区
3. Other 其它:_____ (**FN026_w2_3**) province_city_county/city/district 省_市_县/市/区, _____ (**FN026_w2_4**) 乡/镇/街道, _____ (**FN026_w2_5**) village/neighborhood 村/社区
4. Abroad 国外

[IWER: 访员注意：如果记不清详细的地址名称，请在相应输入框中填入"其它"]

FN027_w2 In what month and year did you start to participate in the basic pension insurance of the firms through this work unit? 您是从哪年哪月开始在这个单位参加养老保险的? _____ 1900...2015 (**FN027_w2_1**) year 年 _____ 0...12 (**FN027_w2_2**) month 月

[IWER: Mark the year using four digits. Take down the month as its actual number. For example, write January as "1" not "01", December as "12". If do not remember month, fill '0' 访员注意：用 4 位数表示年，按照实际的月份填写月。例：1 月写作"1"，而不是"01"，12 月写作"12"。如果记不住月份，请填入'0']

FN028_w2 In what month and year did you start to participate in the basic pension insurance of the firms through this work unit? 您是在哪年哪月停止参加这份养老保险的? _____ 1900...2015 (**FN028_w2_1**) year 年 _____ 0...12 (**FN028_w2_2**) month 月

[IWER: Mark the year using four digits. Take down the month as its actual number. For example, write January as "1" not "01", December as "12". If do not remember month, fill '0' 访员注意：用 4 位数表示年，按照实际的月份填写月。例：1 月写作"1"，而不是"01"，12 月写作"12"。如果记不住月份，请填入'0']

FN029_w2 What was the reason for you to stop participating in this pension? 您停止参加这份养老保险的原因是?（Multiple choice 可多选）

1. Business closed 单位倒闭了
2. I changed job/I was fired 换工作了/被辞退了
3. I moved to other city, pension can not be transferred 更换工作城市，无法转移保险
4. I thought it was not worth and exited 感觉不划算，自愿退保

Part 2 政府机关、事业单位和企业职工基本养老保险（年金）

FN030_w2 Did you participate in supplemental pension insurance of the government(institutions) firm or receive the pension? 您是否参加了政府机关、事业单位、企业补充养老保险，或者正在领取该补充养老保险金?

1. Yes, but I don' t receive it 参加了，还未领取
2. Yes, I receive it 参加了，现在在领取
3. No 没有参加 → Skip to FN069_w3 跳至 FN069_w3

FN031_w2 Where did you participate in the insurance? 您在哪儿参加的这份保险? _____ (**FN031_w2_1**) Province 省/自治区/直辖市 _____ (**FN031_w2_2**) city 市 _____ (**FN031_w2_3**) county 县

FN032_w2 What type of retirement pension plan is/was your employer’s supplement pension? 您的单位选择的补充养老金属于哪种类型？

1. Defined Benefit (DB) Retirement Pension 收益确定型退休金
2. Defined Contribution (DC) Retirement Pension 缴费确定型退休金

DB Retirement Pension Plan: A worker's retirement pension is determined in advance and the amount paid by the user shall change based on how well the savings are managed. 收益确定性退休金计划：预先决定员工的养老金，使用者所支付的金额根据储蓄管理情况而定。
DC Retirement Pension Plan: The amount paid by the user is determined in advance and the retirement pension paid to a worker shall change based on how well the savings are managed. 缴费确定性退休金计划：预先决定使用者支付的金额，员工领取的退休金根据储蓄管理情况而定。

**PROCEDURE 程序:**
Skip FN033_w2 - FN040_w2_bracket if FN030_w2 = 2 如果FN030_w2 = 2，跳过FN033_w2 - FN040_w2_bracket

FN033_w2 For how many years altogether have you been included in this plan? [Include years with other employers if the same plan.] 您参加该保障制度多少年了？[包括受雇不同雇主但享受同一计划的年度。] _____ 0.00...100.00 Years 年

FN034_w2 At what age do you expect to start receiving benefits from this plan? 您预期什么时候开始从这份保险中领取保险金？

1. At age 在_____ 0...120 (**FN034_w2_1**) 岁 or in 或在 _____ 0...120 (**FN034_w2_2**) years 年之后
2. I do not expect receiving these benefits because I have received cash settlements 领取不到保险金，因为已被买断 → Skip to FN040_w2 跳至FN040_w2
3. I do not expect receiving these benefits because I have lost benefits 领取不到保险金，因为保险已经中断。→ Skip to FN069_w3 跳至 FN069_w3
4. Other 其他 → Skip to FN069_w3 跳至 FN069_w3

FN035_w2 What is the combined monthly contribution from you and your employer? Of which, how much do you pay? 每个月您和雇主一共缴纳多少钱？_____ (**FN035_w2_1**) Yuan 元；其中，您个人支付部分是多少？_____ 0.00...100.00 (**FN035_w2_2**) %

FN035_w2_bracket [IWER: If R is unwilling to answer or does not remember, ask unfolding bracket questions here 访员注意：如果受访者不愿回答或者忘记了，在此处分级展开提问] 500 /1,000 /2,000 /3,500 /5,000 yuan 元

**PROCEDURE 程序:**
If FN032_w2 = 1 ask: 如果 FN032_w2 = 1，问：

FN036_w2 For a DB plan, do you know how much you are entitled to at (age in FN034_w2)? 如果是收益确定性退休金计划，[开始领退休金的年龄，preload FN034_w2] 岁时，您知道每月可以领取多少钱？_____ (**FN036_w2_1**) Yuan per month 元/月 Or 或 _____ 0.00...100.00 (**FN036_w2_2**) Or 或 _____ (**FN036_w2_3**) Yuan 元 (Lump sum amount 一次结清)

FN036_w2_bracket [IWER: If R is unwilling to answer or does not remember, ask unfolding bracket questions here 访员注意:如果受访者不愿回答或者忘记了,在此处分级展开提问] 500 /1,000 /2,000 /3,500 /5,000 yuan 元

PROCEDURE 程序:
Skip to FN069_w3 跳至 FN069_w3

PROCEDURE 程序:
If FN032_w2 = 2 ask: 如果FN032_w2 = 2，问:

FN037_w2 For a DC plan, have you ever checked your account balance? 如果是缴费确定型退休金计划，您有没有查看过账户余额?

1. Yes 有, _____ (**FN037_w2_1**) Yuan 元 in _____ (1900...2015 (**FN037_w2_2**) Year 年 _____ (0...12 (**FN037_w2_3**) Month 月
   [IWER: Mark the year using four digits. Take down the month as its actual number. For example, write January as "1" not "01", December as "12". If do not remember month, fill '0' 访员注意: 用 4 位数表示年，按照实际的月份填写月。例: 1 月写作 "1"，而不是 "01"，12 月写作 "12"。如果记不住月份，请填入' 0']
2. No 没有

FN038_w2 What is the earliest age at which you could leave this employer and start to receive pension benefits? 您最早可以在多大年纪时离开这家单位，并开始领取这项养老金?
_____ 45...120 Years old 岁

FN039_w2 By how much would your pension be reduced from full benefits if you left this job at (AGE IN FN038_w2)? 如果您 [preload FN038_w2] 岁时离开现在的工作，您可领取的养老金会比预期的全额养老金减少多少? By _____ 0.00...100.00 (**FN039_w2_1**) % or 或 _____ (**FN039_w2_2**) Yuan 元

FN039_w2_bracket [IWER: If R is unwilling to answer or does not remember, ask unfolding bracket questions here 访员注意:如果受访者不愿回答或者忘记了,在此处分级展开提问] 500 /1,000 /2,000 /3,500 /5,000 yuan 元

PROCEDURE 程序:
Skip to FN069_w3 跳至 FN069_w3

FN040_w2 How much cash settlements did you receive? 您买断时拿了多少钱? _____ Yuan 元

FN040_w2_bracket [IWER: If R is unwilling to answer or does not remember, ask unfolding bracket questions here 访员注意: 如果受访者不愿回答或者忘记了，在此处分级展开提问] 1000 /5,000 /10,000 /50,000 /100,000 yuan 元

PROCEDURE 程序:
Skip to FN069_w3 跳至 FN069_w3

FN041_w2 In what month and year did you start to receive pension benefits from supplement pension insurance of the firms? 您是从哪年哪月开始领企业补充养老保险金的? _____ 1900...2015 (**FN041_w2_1**) year 年 _____ 0...12 (**FN041_w2_2**) month 月 [访员注意:“企业补充养老保险”也叫企业年金,是指企业及其职工在依法参加基本养老保险的基础上,自愿建立的补充养老保险制度] [IWER: Mark the year using four digits. Take down the month as its actual number. For example, write January as “1” not “01”, December as “12”. If do not remember month, fill ‘0’ 访员注意:用 4 位数表示年,按照实际的月份填写月。例:1 月写作“1”,而不是“01”,12 月写作“12”。如果记不住月份,请填入’0’]

FN042_w2 What are your monthly benefits? 您每月领多少钱? _____ Yuan per month 元/月

FN042_w2_bracket [IWER: If R is unwilling to answer or does not remember, ask unfolding bracket questions here 访员注意:如果受访者不愿回答或者忘记了,在此处分级展开提问] 500 /1,000 /2,000 /3,500 /5,000 yuan 元

Part 3 新型农村养老保险、城乡居民养老保险及城镇居民养老保险

FN069_w3 您是否有资格参加以下三种基本养老保险:新农保、城居保和城乡居民养老保险(新型农村养老保险、城乡居民养老保险及城镇居民养老保险),也就是说你们村/社区开展了上述三种基本养老保险,并且您没有参加机关事业单位养老保险和企业职工养老保险。

1. 是的,我有参加上述三种保险的资格,并且没有参加机关事业单位养老保险和企业职工养老保险。
2. 是,我有资格,其他原因,请注明 _____ (**FN069_w3_1**)
3. 否,我没有上述三种保险的资格,因为我已经参加了机关事业单位养老保险和企业职工养老保险。
4. 否,我没有上述三种保险的资格,因为本村/社区没有实施。
5. 否,我没有资格,其他原因,请注明 _____ (**FN069_w3_2**)。

FN057_w3 What kind of pension did you participate in or receive? (multiple choice) 您是否参加了/正在领取以下养老保险?

1. 新农保
2. 城乡居民养老保险
3. 城镇居民养老保险
4. 以上都没有

PROCEDURE 程序:

If FN057_w3=4 and (FN069_w3=1 or FN069_w3=2), ask FN057_w3_0. 如果 FN057_w3=4 且 (FN069_w3=1 或 FN069_w3=2), 问 FN057_w3_0

If FN057_w3=4 and (FN069_w3=3 or FN069_w3=4 or FN069_w3=5), skip to FN079_w2_1. 如果 FN057_w3=4 且 (FN069_w3=3 或 FN069_w3=4 或 FN069_w3=5), 跳到 FN079_w2_1

If FN057_w3 is multiple choiced (FN057_w3=1 or FN057_w3=2 or FN057_w3=3), ask FN058_w2-FN075_w3_5 for every answer 如果 FN075_w3_5 多选 (=1 或 =2 或 =3 或多选), 则针对每个答案分别询问 FN058_w2-FN075_w3_5

FN057_w3_0 您刚才告诉我们您有资格，但是您没有参加的是以下哪种养老保险？
1. 新农保
2. 城乡居民养老保险
3. 城镇居民养老保险

PROCEDURE 程序:
If FN057_w3_0 is multiple choiced , ask FN057_w3_1-FN057_w3_6 for every answer 如果 FN057_w3_0 多选，则针对每个答案分别询问 FN057_w3_1-FN057_w3_6

FN057_w3_1 你们村、社区最初开展 [下载 FN057_w3_0 的答案] 的时候，您是否已经够领取的年龄，可以直接领取而不用自己再缴费了？［子女缴费不算］
1. 我当时已经够领取养老金的年龄，并且开始领取养老金 Skip to FN057_w3_2. 跳至 FN057_w3_2
2. 我当时已经够领取养老金的年龄，但没有开始领取养老金 Skip to FN057_w3_3. 跳至 FN057_w3_3
3. 我当时还不够领取养老金的年龄 Skip to FN057_w3_4. 跳至 FN057_w3_4

FN057_w3_2 你们村/社区最初开展 [下载 FN057_w3_0 的答案] 的时候，村里是否要求您的子女也参保，您才可以自己不用交钱，直接领取？
1. 是
2. 否

PROCEDURE 程序:
Skip to FN057_w3_5. 跳至 FN057_w3_5

FN057_w3_3 你们村/社区最初开展 [下载 FN057_w3_0 的答案] 的时候，没有领取养老金的原因是什么？
1. 因为当初村/社区要求子女缴费参加，我才可以参加，但我的子女没有缴费参加
2. 我没有缴费
3. 我不知道有这回事
4. 我不在户口所在地
5. 我不知道去哪里领
6. 我身体原因无法去领
7. 其他，请注明 _____ (**FN057_w3_3_1**)

PROCEDURE 程序:
Skip to FN057_w3_5. 跳至 FN057_w3_5

FN057_w3_4 您现在身份证上的年龄已经到领取 [下载 FN057_w3_0 的答案] 的年龄吗？
1. 是
2. 否 skip to FN057_w3_6. 跳至 FN057_w3_6

FN057_w3_5 您现在不领取 [下载 FN057_w3_0 的答案] 的原因?

1. 我没有缴费
2. 我不知道有这回事
3. 我不在户口所在地
4. 我不知道去哪里领
5. 我身体原因无法去领
6. 其他，请注明 _____ (**FN057_w3_5_1**)

**PROCEDURE 程序**:
Skip to FN079_w2_1. 跳至 FN079_w2_1

FN057_w3_6 您现在没有缴纳 [下载 FN057_w3_0 的答案] 的原因?

1. 我没有钱
2. 我对待遇水平不满意，缴费参加该保险是不划算的。
3. 待遇水平低，对我的生活没有什么意义
4. 申请办理或缴费不方便
5. 缴费设计不合理
6. 我没有本地户口
7. 我已经参加了其他社会养老保险项目，不能重复参加
8. 其他，请注明 _____ (**FN057_w3_6_1**)

**PROCEDURE 程序**:
Skip to FN079_w2_1. 跳至 FN079_w2_1

FN058_w2 For every answers of FN057_w3 , do you participate in or receive the pension? 对于 [下载 FN057_w3 的答案]，您现在是参加保险（正在缴费）、还是正在领取保险金?

1. Participate in 参加保险/正在缴费
2. Receive the pension 正在领取养老保险金

FN059_w2 Where did you participate in the pension? 您在哪儿参加的 [下载 FN057_w3 的答案]?

1. The same as permanent address 和目前常住地一样
2. Another village/neighborhood in permanent address's county/city/district 常住地所在县/市/区的其他 _____ (**FN059_w2_1**) 乡/镇/街道，_____ (**FN059_w2_2**) village/neighborhood 村/社区
3. Other 其它:_____ (**FN059_w2_3**) province_city_county/city/district 省 _ 市 _ 县/市/区，_____ (**FN059_w2_4**) 乡/镇/街道，_____ (**FN059_w2_5**) village/neighborhood 村/社区
4. Abroad 国外

[IWER: 访员注意：如果记不清详细的地址名称，请在相应输入框中填入“其它”]

FN061_w3 您是从哪年哪月开始参加 [下载FN057_w3 的答案] 的? _____ 1900...2015 (**FN061_w3_1**) year 年 _____ 0...12 (**FN061_w3_2**) month 月

PROCEDURE 程序:

Skip FN060_w2-FN066_w2_bracket if FN058_w2 = 2. 如果 FN058_w2 = 2，跳过 FN060_w2-FN066_w2_bracket

FN060_w2 Have you ever contributed to your [preload FN057_w3]? 您参加 [下载 FN057_w3 的答案] 缴过费吗？

[CAPI: (1) 如果是家人或亲戚替自己缴费，也算缴费 (2) If FN057_w3 = 1 or FN057_w3 = 2, prompt: If your residents' pension was transferred from other pension programs, your contribution to these other programs also counts. 如果 FN057_w3 = 1 or FN057_w3 = 2，提示：如果您的城乡居民养老保险或城镇居民养老保险是从其他保险转过来的，为其他养老保险缴的费用也算]

1. Yes 是，缴过
2. No 否，没有 → 跳到 FN063_w2 前的控制程序

FN072_w3 您在加入 [下载FN057_w3 的答案] 时，是不是需要补缴一笔钱？

1. 是
2. 否 Skip to FN072_w3_4 跳到 FN072_w3_4

[IWER: If R is unwilling to answer or does not remember, ask unfolding bracket questions here 访员注意：参加（新农保）补缴费用是指受访者预计在 60 岁时不够 15 年的缴费年限；或者此前一直没有参加，需要补缴之前的费用；或者希望未来领取更多的养老金而一次性或分多次缴纳的一笔费用]

FN072_w3_1 您总共需要补缴多少钱？

1. _____ (**FN072_w3_1_1**) 元
2. 或 _____ (**FN072_w3_1_1**) 万元

FN072_w3_2 您补缴多少钱？

1. _____ (**FN072_w3_2_1**) 元
2. 或 _____ (**FN072_w3_2_2**) 万元

FN074_w3 到目前为止，谁为您补缴了这笔费用？(多选题)

1 我自己
2-26 [加载子女姓名]
27 其他家人或亲戚
28 其他人，请注明 _____ (**FN074_w3_1**)

FN072_w3_4 除了您在上面所说补缴的费用，您现在是否还需要按年缴纳 [下载FN057_w3 的答案] 费用？

1. 是，现在还在按年缴纳，每年交 _____ (**FN072_w3_4_1**) 元，包括补贴 _____ (**FN072_w3_4_2**) 元，已经缴纳了 _____ (**FN072_w3_4_3**) 年，还需缴纳 _____ (**FN072_w3_4_4**) 年。
2. 否 Skip to FN063_w2 跳到 FN063_w2

FN072_w3_5 最近一年的缴纳费用是谁为您缴纳的？(多选题)

1 我自己
2-26 [加载子女姓名]
27 其他家人或亲戚
28 其他人，请注明 _____ (**FN072_w3_5_1**)

PROCEDURE 程序:

Ask FN063_w2 and FN064_w2 if the respondent is enrolled in residents' pension FN057_w3 = 2 or FN057_w3 = 3 . If FN057_w3 = 1, skip to FN065_w2 如果受访者参加了新农保 FN057_w3 = 1，跳到 FN065_w2。如果受访者参加了城乡居民养老保险 FN057_w3 = 2 或者城镇居民养老保险 FN057_w3 = 3，请回答FN063_w2和FN064_w2

FN063_w2 您参加的城乡居民养老保险、城镇居民养老保险是从企业职工养老保险转过来的吗？

1. Yes 是的
2. No 不是 → Skip FN064_w2 跳过 FN064_w2

[访员注意：(1)"农村养老保险"是指农村社会养老保险。1992 年民政部颁布了《县级农村社会养老保险基本方案》，确定以县为基本单位开展农村社会养老保险。(2)"企业基本养老保险"：是指受访者参加社会基本养老保险制度，达到退休年龄并符合领取条件后，领取到社会养老保险金。]

FN064_w2 您参加的城乡居民养老保险、城镇居民养老保险是从以下哪种养老保险转过来的？

1. 从企业职工基本养老保险转过来了 _____ (**FN064_w2_1**) 缴费年限
2. 从其他保险转过来的

FN065_w2 When do you expect to receive pension? 您预期在多大年龄享用该保险？At age 在 _____ 45...120 (**FN065_w2_1**) years old 岁 or in 或在 _____ 0.00...100.00 (**FN065_w2_2**) years 年之后

[Soft Check: Prompt or Verify if FN065_w2_1 <50 or FN065_w2_2 >60]

FN066_w2 About how much do you expect your benefits to be? (as an amount per month or year or a lump sum?) 您预期自己能领多少钱？_____ (**FN066_w2_1**) Yuan per month 元/月 Or 或 _____ (**FN066_w2_2**) Yuan 元 (Lump sum amount 一次结清)

[Soft Check: Prompt or Verify if These Benefits are Low or High, e.g., FN066_w2_1 <100 | FN066_w2_1 >5000 per month.]

FN066_w2_bracket [IWER: If R is unwilling to answer or does not remember, ask unfolding bracket questions here 访员注意：如果受访者不愿回答或者忘记了，在此处分级展开提问] 500 /1,000 /2,000 /3,500 /5,000 yuan 元

PROCEDURE 程序:

If FN058_w2 = 2 ask FN067_w2-FN075_w3_5

Otherwise skip to FN079_w2_1. 跳至 FN079_w2_1

FN067_w2 In what month and year did you start to receive your [preload FN057_w3]? 您是从哪年哪月开始领取 [下载 FN057_w3]? _____ 1900...2015 (**FN067_w2_1**) year 年 _____ 0...12 (**FN067_w2_2**) month 月
[IWER: Mark the year using four digits. Take down the month as its actual number. For example, write January as “1” not “01”, December as “12”. If do not remember month, fill ‘0’ 访员注意: 用 4 位数表示年, 按照实际的月份填写月。例: 1 月写作 “1”, 而不是 “01” ,12 月写作 “12”。如果记不住月份, 请填入’ 0’]

FN068_w2 How much do you receive now? (as an amount per month?) 您现在每月领多少钱? _____ Yuan per month 元/月

FN068_w2_bracket [IWER: If R is unwilling to answer or does not remember, ask unfolding bracket questions here 访员注意: 如果受访者不愿回答或者忘记了, 在此处分级展开提问] 500 /1,000 /2,000 /3,500 /5,000 yuan 元

FN068_w3 你们村/社区最初开展 FN057_w3 的时候, 您是否已经够领取 FN057_w3 的年龄, 可以直接领取而不用自己缴费了? [子女缴费不算]
1. 是
2. 否 FN075_w3 跳至 FN075_w3

FN068_w3_1 你们村/社区最初开展 FN057_w3 的时候, 村里/社区是否要求您的子女也参保, 您才可以自己不用交钱, 直接领取?
1. 是
2. 否 Skip to FN075_w3 跳至 FN075_w3

FN068_w3_2 当时您的哪些子女根据村/社区里的要求, 为了您能够领取 FN057_w3, 开始缴费?

1-25 [加载子女姓名]
26 没有人

FN075_w3 您在领取 FN057_w3 时, 是不是需要补缴一笔钱?
1. 是
2. 否 Skip to FN075_w3_4 跳至 FN075_w3_4

[IWER: If R is unwilling to answer or does not remember, ask unfolding bracket questions here 访员注意: 参加 (新农保) 补缴费用是指受访者预计在 60 岁时不够 15 年的缴费年限; 或者此前一直没有参加, 需要补缴之前的费用; 或者希望未来领取更多的养老金而一次性或分多次缴纳的一笔费用]

FN075_w3_1 您总共需要补缴多少钱?
1. _____ (**FN075_w3_1_1**) 元
2. 或 _____ (**FN075_w3_1_2**) 万元

FN075_w3_2 您补缴多少钱?
1. _____ (**FN075_w3_2_1**) 元

2. 或 _____ (**FN075_w3_2_2**) 万元

FN076_w3 谁为您补缴了这笔费用？(多选题)

1 我自己
2-26 [加载子女姓名]
27 其他家人或亲戚
28 其他人，请注明 _____ (**FN076_w3_1**)

FN075_w3_4 除了您在上面所说补缴的费用，您是否还曾经需要按年缴纳 FN057_w3 费用？

1. 是，曾经需要按年缴纳，缴纳了 _____ (**FN075_w3_4_1**) 年，总共缴纳了 _____ (**FN075_w3_4_2**) 钱。
2. 否 Skip to FN079_w2_1. 跳至 FN079_w2_1.

FN075_w3_5 谁为您缴纳了这笔费用？（多选题）

1 我自己
2-26 [加载子女姓名]
27 其他家人或亲戚
28 其他人，请注明 _____ (**FN075_w3_5_1**)

Part 4 征地养老保险（失地农民养老保险/被征地农民养老保险）

FN079_w2_1 Whether your land was acquired? 您家的土地是否被征用过？

1. Yes 是
2. No 否 → Skip to FN080_w2 请跳至FN080_w2

FN079_w2_2 When was your land acquired? 您家的土地是哪一年被征用的？_____ 1900...2015 Year 年

FN079_w2_3 Did you participate in land expropriation pension insurance, or did you receive the pension? 您是否参加了/正在领取征地养老保险？

1. Yes, but I don’t receive it 参加了保险，还未领取养老保险金
2. Yes, I receive it 正在领取征地养老保险金 → Skip to FN079_w2_10 请跳至FN079_w2_10
3. No 没有参加或领取征地养老保险 → Skip to FN080_w2 请跳至FN080_w2

FN079_w2_4 Do you need to pay the premium by yourself? 您的征地养老保险是否需要个人缴费？

1. Yes 是
2. No 否 → Skip to FN079_w2_7 请跳至 FN079_w2_7

FN079_w2_5 Who paid for this pension insurance? 谁给您的征地养老保险缴费？

1. Myself 我自己
2. My children 我的孩子
3. Other family member or relative 其他家人或亲戚

4. Others 其他人

FN079_w2_6 How much did you need to pay? 要缴纳多少钱? _____ (**FN079_w2_6_1**) Yuan per month 元/月
Or 或 _____ (**FN079_w2_6_2**) Yuan per year 元/年

FN079_w2_7 How much did government and community subsidize? 您的征地养老保险，政府和集体补助多少钱? Government paid 政府出资 _____ (**FN079_w2_7_1**) Yuan per month 元/月 Or 或 _____ (**FN079_w2_7_2**) Yuan per year 元/年 Community paid 集体出资 _____ (**FN079_w2_7_3**) Yuan per month 元/月 Or 或 _____ (**FN079_w2_7_4**) Yuan per year 元/年

FN079_w2_8 When do you expect to receive this pension? 您预期在什么时候享用该保险? At age 在 _____ 45...120 (**FN079_w2_8_1**) years old 岁 or in 或在 _____ 0.00...100.00 (**FN079_w2_8_2**) years 年之后

FN079_w2_9 About how much do you expect your benefits to be? (as an amount per month or year or a lump sum?) 您预期自己能领多少钱? _____ (**FN079_w2_9_1**) Yuan per month 元/月 Or 或 _____ (**FN079_w2_9_2**) Yuan 元 (Lump sum amount 一次结清)

PROCEDURE 程序:
If FN079_w2_3 = 2 ask FN079_w2_10-FFN079_w2_11_bracket
Otherwise skip to FN080_w2. 跳至 FN080_w2

FN079_w2_10 In what month and year did you start to receive this pension? 您是从哪年哪月开始领取征地养老保险金的? _____ 1990...2015 (**FN079_w2_10_1**) year 年 _____ 0...12 (**FN079_w2_10_2**) month 月
[IWER: Mark the year using four digits. Take down the month as its actual number. For example, write January as "1" not "01", December as "12". If do not remember month, fill '0' 访员注意: 用 4 位数表示年，按照实际的月份填写月。例: 1 月写作"1"，而不是"01"，12 月写作"12"。如果记不住月份，请填入'0']

FN079_w2_11 How much do you receive now? (as an amount per month?) 您现在每月领多少钱? _____ Yuan per month 元/月

FN079_w2_11_bracket [IWER: If R is unwilling to answer or does not remember, ask unfolding bracket questions here 访员注意：如果受访者不愿回答或者忘记了，在此处分级展开提问] 10 /50 /100 /500 /1,000 yuan 元/月

Part 5 高龄老人补贴

FN080_w2 Did you receive old age pension allowance? 您是否在领取高龄老人补贴?
1. Yes 是
2. No 否 → Skip to FN056_w2_1 请跳至 FN056_w2_1

FN081_w2 In what month and year did you start to receive the pension subsidy for the oldest old? 您是从哪年哪月开始领取高龄老人养老补贴的？_____ 1990...2015 (**FN081_w2_1**) year 年 _____ 0...12 (**FN081_w2_1**) month 月
[访员注意：“高龄老人养老补助（补贴）”指 60 岁以上老人享有政策性的优惠，如乘车免费、挂号费免费、参观等免费、部分社区活动免费，如注射流感疫苗等，百岁以上老人才享有月补助金]
[IWER: Mark the year using four digits. Take down the month as its actual number. For example, write January as “1” not “01”, December as “12”. If do not remember month, fill ‘0’ 访员注意：用 4 位数表示年，按照实际的月份填写月。例：1 月写作 “1”，而不是 “01”，12 月写作 “12”。如果记不住月份，请填入’0’]

FN082_w2 How much do you receive now? 您现在每月领多少钱？_____ Yuan per month 元/月

FN082_w2_bracket [IWER: If R is unwilling to answer or does not remember, ask unfolding bracket questions here 访员注意：如果受访者不愿回答或者忘记了，在此处分级展开提问] 500 /1,000 /2,000 /3,500 /5,000 yuan 元

Part 6 人寿保险

FN056_w2_1 Did you participate in life insurance? 你是否购买过人寿保险？
1. Yes 是
2. No 否 → Skip to FN043_w2 跳至 FN043_w2

FN056_w2_2 When did you start to participate in life insurance? 您什么时候购买人寿保险的？_____ 1900...2015 (**FN056_w2_2_1**) year 年 _____ 0...12 (**FN056_w2_2_2**) month 月

FN056_w2_3 What kind of life insurance did you participate in? 你购买的人寿保险是哪一种？
1. Term life insurance 定期寿险
2. Whole life insurance 终身寿险
3. Survivorship insurance 生存保险
4. Endowment insurance 生死两全保险
5. Other, please specify 其它（请填写具体名称 _____）(**FN056_w2_3_1**)

FN056_w2_4 Who paid for your life insurance? 谁给您的人寿保险缴费？
1. Myself 我自己
2. My children 我的孩子
3. Other family member or relative 其他家人或亲戚
4. Others 其他人

FN056_w2_5 How to pay the insurance premium? 保险费的缴纳方式是？
1. Monthly 月交
2. Quaterly 季交
3. Half a year 半年交

4. Annually 年交

FN056_w2_6 How much is the payment amount? 缴费金额是多少？
1. If pay it monthly 如果月交，_____ Yuan/Month 元/月 (**FN056_w2_6_1**)
2. If pay it quarterly 如果季交，_____ Yuan/Quarter 元/季 (**FN056_w2_6_2**)
3. If pay it every six months 如果半年交，_____ Yuan/Half a year 元/半年 (**FN056_w2_6_3**)
4. If pay it annually 如果年交，_____ Yuan/Year 元/年 (**FN056_w2_6_4**)

FN056_w2_7 Did you receive reimbursement from your life insurance? 你是否从人寿保险中领取过赔付金？
1. Yes 是
2. No 否 → Skip to FN056_w2_9 跳至 FN056_w2_9

FN056_w2_8 How much did you get? 领取过多少钱？_____ Yuan 元

FN056_w2_9 How much do you expect to receive? 你预计将来可能从人寿保险中领取多少钱？_____ (**FN056_w2_9_1**) Yuan/Month 元/月 Or 或 _____ (**FN056_w2_9_2**) Yuan 元(Lump sum amount 一次结清)

Part 7 商业养老保险（人寿保险除外）

FN043_w2 Did you participate in commercial pension insurance, did someone buy commercial pension insurance for you, or did you get commercial pension insurance? 您是否参加了/别人给买了商业养老保险，或者正在领取商业养老保险金？
1. Yes, but I don't receive it 参加了/别人帮忙买了，但是还未领取
2. Yes, I receive it 现在在领取商业养老保险金
3. No 没有参加或领取商业养老保险 → Skip to FN083_w2 跳至 FN083_w2

FN044_w2 Where did you participate in this insurance? 您在哪儿参加的这份保险？
1. The same as permanent address 和目前常住地一样
2. Another village/neighborhood in permanent address's county/city/district 常住地所在县/市/区的其他 _____ (**FN044_w2_1**) 乡/镇/街道，_____ (**FN044_w2_2**) village/neighborhood 村/社区
3. Other 其它:_____ (**FN044_w2_3**) province_city_county/city/district 省 _ 市 _ 县/市/区，_____ (**FN044_w2_4**) 乡/镇/街道，_____ (**FN044_w2_5**) village/neighborhood 村/社区
4. Abroad 国外 [访员注意：如果记不清详细的地址名称，请在相应输入框中填入"其它"]

FN045_w2 Who paid for the commercial pension insurance? 谁给您买的商业养老保险？
1. Myself 我自己
2. My employer 我的雇主
3. My family or relative 我的家人或亲戚
4. Others 其他人

[访员注意：“商业养老保险”是指受访者直接向商业保险公司投保，定期缴纳保险费，从合同约定年龄开始持续、定期地领取养老金的人寿保险，能有效地满足客户的养老需要]

PROCEDURE 程序:
Skip FN046_w2-FN054_w2_bracket if FN043_w2 = 2 如果FN043_w2 = 2，跳过FN046_w2-FN054_w2_bracket

FN046_w2 When did you start paying for the commercial pension? 您什么时候开始购买这商业养老保险的？_____ 1900...2015 (**FN046_w2_1**) year 年 _____ 0...12 (**FN046_w2_2**) month 月
[访员注意：“商业养老保险”是指受访者直接向商业保险公司投保，定期缴纳保险费，从合同约定年龄开始持续、定期地领取养老金的人寿保险，能有效地满足客户的养老需要] [IWER: Mark the year using four digits. Take down the month as its actual number. For example, write January as “1” not “01”, December as “12”. If do not remember month, fill ‘0’ 访员注意：用 4 位数表示年，按照实际的月份填写月。例：1 月写作“1”，而不是“01”，12 月写作“12”。如果记不住月份，请填入’0’]

FN047_w2 How do you contribute to the commercial pension? 以什么样的形式交纳保险费?
1. Annual payment 按年
2. Lump sum amount 一次性缴费 → Skip to FN050_w2 跳至 FN050_w2

FN048_w2 You contribute _____ yuan/ year to the commercial insurance ? 您每年需要交纳多少保险费？_____ Yuan/ year 元/年

FN049_w2 How many years do you need to pay? 您需要缴几年保险费? _____ Years 年
[Soft Check: Prompt for verification if greater than a legal maximum]

PROCEDURE 程序:
Skip FN050_w2-FN050_w2_bracket if FN047_w2 = 1 如果FN047_w2 = 1，跳过FN050_w2-FN050_w2_bracket

FN050_w2 How much premium do you need to pay in total? 您一共需要交纳多少保险费？_____ Yuan 元

FN050_w2_bracket [IWER: If R is unwilling to answer or does not remember, ask unfolding bracket questions here 访员注意：如果受访者不愿回答或者忘记了，在此处分级展开提问]
1,000 /2,000 /5,000 /10,000 yuan 元

FN051_w2 How do you receive the pension? 保险费的领取方式？
1. Lump sum amount 一次性领取 → Skip FN052_w2-FN053_w2_bracket 请跳过FN052_w2-FN053_w2_bracket
2. Yearly 年领
3. Monthly 月领

PROCEDURE 程序:
If FN051_w2 = 2 ask FN052_w2 and FN052_w2_bracket

FN052_w2 How much do you expect to receive _____ yuan/ year after your retirement? 您预期退休后每年能领取多少保险费? _____ Yuan/year 元/年
[Soft Check: Prompt for verification if low or high, e.g. FN052_w2 < 1200 per year or FN052_w2 > 60000 per year]

FN052_w2_bracket [IWER: If R is unwilling to answer or does not remember, ask unfolding bracket questions here 访员注意:如果受访者不愿回答或者忘记了,在此处分级展开提问] 500 /1,000 /2,000 /3,500 /5,000 yuan 元

PROCEDURE 程序:
If FN051_w2 = 3 ask FN053_w2 and FN053_w2_bracket

FN053_w2 How much do you expect to receive _____ yuan/month in the future? 您预期以后每月能领取多少养老金? _____ Yuan/ month 元/月
[Soft Check: Prompt for verification if low or high, e.g. FN053_w2 < 100 per month or FN053_w2 >5000 per year]

FN053_w2_bracket [IWER: If R is unwilling to answer or does not remember, ask unfolding bracket questions here 访员注意:如果受访者不愿回答或者忘记了,在此处分级展开提问] 500 /1,000 /2,000 /3,500 /5,000 yuan 元

FN054_w2 How much do you expect to receive _____ yuan in total? 您预期领取的养老金总额能达到多少? _____ Yuan 元

FN054_w2_bracket [IWER: If R is unwilling to answer or does not remember, ask unfolding bracket questions here 访员注意:如果受访者不愿回答或者忘记了,在此处分级展开提问] 1,000 /5,000 /10,000 /50,000 /100,000 yuan 元

PROCEDURE 程序:
Skip to FN083_w2. 跳至 FN083_w2

FN055_w2 In what month and year did you start to receive commercial pension benefits? 您是从哪年哪月开始领商业养老金的? _____ 1900...2015 (**FN055_w2_1**) year 年 _____ 0...12 (**FN055_w2_2**) month 月
[IWER: Mark the year using four digits. Take down the month as its actual number. For example, write January as "1" not "01", December as "12". If do not remember month, fill '0' 访员注意: 用 4 位数表示年,按照实际的月份填写月。例: 1 月写作"1",而不是"01",12 月写作"12"。如果记不住月份,请填入'0']

FN056_w2 What is your monthly benefit? 您每月领多少钱? _____ Yuan per month 元/月

FN056_w2_bracket [IWER: If R is unwilling to answer or does not remember, ask unfolding bracket questions here 访员注意:如果受访者不愿回答或者忘记了,在此处分级展开提问] 500 /1,000 /2,000 /3,500 /5,000 yuan 元

Part 8 其他养老保险

FN083_w2 You just told us you are receiving benefits from other pension program, what is the name of this pension program? 除了上述几种养老保险之外，您是否还参加了其他养老保险项目/正在领取其他养老保险金？

1. Yes 是，正在参加其他养老保险项目/正在缴费，目前还未领取养老金
2. Yes 是，参加了其他养老保险项目，现在在领取养老金
3. No 否，没有参加其他养老保险项目 → Skip to FN097_w2 请跳至 FN097_w2

FN084_w2 What is the name of the program? 这个项目的名称是 _____

[访员注意：如果受访者回答正在参加/领取多份其他养老保险，请其选取自己认为最主要的一份回答]

FN085_w2 where did you participate in the pension program? 您在哪儿参加的这份保险项目？

1. The same as permanent address 和目前常住地一样
2. Another village/neighborhood in permanent address's county/city/district 常住地所在县/市/区的其他 _____ (**FN085_w2_1**) 乡/镇/街道，_____ (**FN085_w2_2**) village/neighborhood 村/社区
3. Other 其它:_____ (**FN085_w2_3**) province_city_county/city/district 省 _ 市 _ 县/市/区，_____ (**FN085_w2_4**) 乡/镇/街道，_____ (**FN085_w2_5**) village/neighborhood 村/社区
4. Abroad 国外

[访员注意：如果记不清详细的地址名称，请在相应输入框中填入“其它”]

PROCEDURE 程序:

Skip FN086_w2-FN094_w2_2_bracket if FN083_w2 = 2. 如果FN083_w2 = 2，跳过FN086_w2-FN094_w2_2_bracket

FN086_w2 When did you start to participate in this pension? 您什么时候开始参加这份养老保险的？ _____ 1990...2015(**FN086_w2_1**)year 年 _____ 0...12 (**FN086_w2_2**)month 月

[IWER: Mark the year using four digits. Take down the month as its actual number. For example, write January as “1” not “01”, December as “12”. If do not remember month, fill ‘0’ 访员注意：用 4 位数表示年，按照实际的月份填写月。例：1 月写作“1”，而不是“01”，12 月写作“12”。如果记不住月份，请填入‘0’]

FN087_w2 Did you need to pay the premium? 您参加的这份养老保险需要缴费吗？

1. Yes 需要
2. No 不需要 → Skip to FN093_w2 请跳至 FN093_w2

FN088_w2 Who paid for the this pension insurance? 谁给您的这份养老保险缴费？

1. Myself 我自己
2. My employer 我的雇主
3. My family or relative 我的家人或亲戚

4. Others 其他人

FN089_w2 How do you contribute to the commercial pension? 以什么样的形式交纳保险费?
1. Annual/Monthly payment 按年/按月
2. Lump sum amount 一次性缴费 → Skip to FN092_w2 请跳至 FN092_w2

FN090_w2 You contribute _____ Yuan/ year to the commercial insurance ? 按年算的话，您每年需要交纳多少保险费? _____ Yuan/ year 元/年

FN091_w2 How many years do you need to pay? 您需要缴几年保险费? _____ Years 年
[Soft Check: Prompt for verification if greater than a legal maximum]

PROCEDURE 程序:
Skip FN092_w2-FN092_w2_bracket if FN089_w2 = 1. 如果 FN089_w2 = 1，跳过 FN092_w2-FN092_w2_bracket

FN092_w2 How much premium do you need to pay in total? 您一共需要交纳多少保险费? _____ Yuan 元

FN092_w2_bracket [IWER: If R is unwilling to answer or does not remember, ask unfolding bracket questions here 访员注意：如果受访者不愿回答或者忘记了，在此处分级展开提问]
2,000 /5,000 /10,000 /50,000/100,000 yuan 元

FN093_w2 When do you expect to receive pension 您预期在多大年龄享用该保险?At age 在_____ (**FN093_w2_1**) 45..120 岁 or in 或在 _____ (**FN093_w2_2**) years 年之后。

FN094_w2 About how much do you expect your benefits to be? (as an amount per month or year or a lump sum?) 您预期自已能领多少钱? _____ (**FN094_w2_1**) Yuan per month 元/月 Or 或 _____ (**FN094_w2_1**) Yuan 元（Lump sum amount 一次结清）

PROCEDURE 程序:
If FN083_w2 = 2 ask FN095_w2 - FN096_w2_bracket . 如果 FN083_w2 = 2 询问FN095_w2 - FN096_w2_bracket
Otherwise skip to FN097_w2. 跳至 FN097_w2

FN095_w2 In what month and year did you start to receive this pension benefits? 您是从哪年哪月开始领取这份养老金（退休金）的? _____ 1990...2015 (**FN095_w2_1**) year 年 _____ 0...12 (**FN095_w2_2**) month 月
[IWER: Mark the year using four digits. Take down the month as its actual number. For example, write January as "1" not "01", December as "12". If do not remember month, fill '0' 访员注意：用 4 位数表示年，按照实际的月份填写月。例：1 月写作"1"，而不是"01"，12 月写作"12"。如果记不住月份，请填入' 0']

FN096_w2 How much do you receive now? 您现在每月领多少钱? _____ Yuan per month 元/月

FN096_w2_bracket [IWER: If R is unwilling to answer or does not remember, ask unfolding bracket questions here 访员注意：如果受访者不愿回答或者忘记了，在此处分级展开提问] 500 /1,000 /2,000 /3,500 /5,000 yuan 元

[Ask all R 所有人回答]

FN097_w2 Who do you think you can rely on financially for old-age support? 如果您将来老了干不动工作了，您认为生活来源主要将是什么？

1. Children 子女 → Skip to FN098_w2 请跳至 FN098_w2
2. Savings 储蓄结束本部分
3. Pension or retirement salary 养老金或退休金结束本部分
4. Commercial pension insurance 商业养老保险结束本部分
5. Other 其他结束本部分

[访员注意："商业养老保险"是指受访者直接向商业保险公司投保，定期缴纳保险费，从合同约定年龄开始持续、定期地领取养老金的人寿保险，能有效地满足客户的养老需要]

FN098_w2 Which child(ren)? 是哪个/哪些子女？(choose all that apply 可多选) [加载子女列表，让受访者选择]

G&H INCOME, EXPENDITURES AND ASSETS 收入、支出与资产

G2 HOUSEHOLD INCOME AND EXPENDITURES 家户收入与支出

[IWER: Part 1_1 is asked of the main respondent and spouse respectively. Other parts in this section is asked of the family respondent. Do not allow a proxy respondent to answer the entire section. Part 1_1 由主要受访者及其配偶分别回答，其余部分由家庭受访者回答。本部分不允许完全请别人代答。]

PART 1 Household Wage Income and Individual-based transfers 家户工资收入和个人获得的转移收入

Part 1_1: Main Respondent and Spouse's Wage Income and Individual-based Transfers 主要受访者及其配偶的工资收入和个人获得的转移收入

[IWER: Please conduct Part 1_1 when the main respondent and spouse are at home. Don't allow a proxy to complete the part. 访员注意：当主要受访者及其配偶在场时，提问 Part 1_1 问卷。这部分不允许请人完全代填。]

GA001 Did you receive any wage and bonus income in the past year? 过去一年，您有没有领工资（包括奖金、各种补贴，不包括退休工资）？

1. Yes 有
2. No 没有 → Skip to GA003 跳至 GA003

GA002 How much did you receive last year? 一共领了多少钱？_____ Yuan 元

Wage income: yuan [soft check >240,000]

GA002_bracket [IWER: If R is unwilling to answer or does not remember, ask unfolding bracket questions here. 访员注意：如果受访者不愿回答或者忘记了，在此处分级展开提问]

10,000 /30,000 /50,000 /100,000 /200,000 Yuan 元

GA002_W2_1 Does the above mentioned wage exclude any insurance, income tax, public housing funds and other fees? 上面提到的工资有没有扣除各类保险、所得税、住房公积金或其他杂费？

1. Yes 有
2. No 没有

GA002_W2_2 What is the total amount of your insurance, income tax, public housing funds and other fees? 您被扣除的/上交的个人所得税、各类保险、住房公积金或其他杂费一共是多少元？

1. _______ (**GA002_w2_2a**) Yuan/Year 元/年

2. _____ (**GA002_w2_2b**) Yuan/Month 元/月
3. About 或相当于工资的 ___ (**GA002_w2_2c**) % of wage
4. No 没有

[IWER: If R is unwilling to answer or does not remember, ask unfolding bracket questions here. 访员注意：如果受访者不愿回答或者忘记了，在此处分级展开提问:] 个人所得税、各类保险、住房公积金或其他杂费一共是多少元/月？300 /500 /1,000 /2,000 /3,000 Yuan/Month 元/月。

Among it, 其中:

1. Income tax 个人所得税是
   1. _______ **GA002_w2_2_1a**)Yuan/Year 元/年
   2. _____ (**GA002_w2_2_1b**)Yuan/Month 元/月
   3. About 或相当于工资的 ___ (**GA002_w2_2_1c**) % of wage
   4. No 没有
2. Various insurance (pension insurance, health insurance, unemployment insurance, worker's injury insurance, maternity insurance) 各类保险（包括养老保险、医疗保险、失业保险、工伤保险、生育保险等）是
   1. _______ (**GA002_w2_2_2a**)Yuan/Year 元/年
   2. _____ (**GA002_w2_2_2b**)Yuan/Month 元/月
   3. About 或相当于工资的 ___ (**GA002_w2_2_2c**) % of wage
   4. No 没有
3. Public housing fund 住房公积金是
   1. _______ (**GA002_w2_2_3a**))Yuan/Year 元/年
   2. _____ (**GA002_w2_2_3b**)Yuan/Month 元/月
   3. About 或相当于工资的 ___ (**GA002_w2_2_3c**) % of wage
   4. No 没有

GA003 Did you receive any of the following types of individual income in the past year? (check all that apply) 过去一年，您有没有领到下列收入？(可多选)

2 Unemployment compensation 失业补助
3 Pension subsidy for the oldest old/pension voucher 高龄老人养老补助/养老券
4 Workers' compensation from Industrial Accident Compensation Insurance includes wage-replacement benefits, disability benefits, and survivors' benefits 工伤保险金包括误工补贴、伤残补助、丧葬费等
5 Elderly family planning subsidies 独生子女老年补助
6 Medical aid 医疗救助
7 Other government subsidies 政府给个人的其他补助
8 Social assistance 社会捐助
9 Other income sources 其他收入
10 None of the above 以上均没有 → Skip GA004 跳过 GA004

GA004 How much did you receive last year? 过去一年一共领了多少钱？____ Yuan 元

PROCEDURE 程序:

If GA003 = 3, ask GA004_W3 and GA004_W3_1. 如果受访者领取了高龄老人养老补助/养老券，询问 GA004_W3和GA004_W3_1。

GA004_W3 What type of the pension subsidy? 领取高龄老人养老补助/养老券的形式是？(可多选)

1. Cash 现金 → Skip to Part 1_2 跳至 Part 1_2
2. Card 卡
3. Voucher 券

GA004_W3_1 How do you use the card/voucher? 您怎样使用养老卡/券的？(可多选)

1. Life care 生活照料
2. Domestic service 家政服务
3. Rehabilitation service 康复服务
4. Buy food 养老餐桌
5. Buy medicine 购买药品
6. Buy life items 购买生活物品
7. Other, please specify 其他，请注明 ____(**GA004_W3_1_1**)

Part 1_2: Other Household Member's Wage Income and Individual-based transfers 其他家户成员的工资收入和个人获得的转移收入

[IWER reminder: make sure others are not present. 访员注意：请确保没有其他人在场。]

[Intro: We'd like to ask you some questions about the income and assets of OTHER members of your household. 下面我们想问问您家其他家户成员的收入情况。我们将会对您的回答严格保密，并且仅用于学术研究。]

PROCEDURE 程序:

The names of other household members are preloaded from the cover screen information. For each member (excluding main respondent and spouse)ask GA005 to GA008_bracket. 其他家户成员名单要从过滤问卷获得。对于每一个家户成员（不包括主要受访者及其配偶），分别询问GA005至GA008_bracket。

GA005 Did [preload household member name] receive any wage and bonus income in the past year? 过去一年，[preload household member name] 有没有领工资（包括奖金，不包括退休工资)？

1. Yes 有
2. No 没有 → Skip to GA007 跳至 GA007

GA006 After tax and employee social insurance , how much did he/she receive in the past year year? 扣除税和缴纳的职工社会保险，过去一年他/她一共领了多少钱？______ (**GA006_1**) Yuan 元 or 或每个月领多少钱？_____ (**GA006_2**) Yuan/Month 元

Wage income: yuan [soft check >200,000] or yuan/month [soft check>15,000]

GA006_bracket [IWER: If R is unwilling to answer or does not remember, ask unfolding bracket questions here. 访员注意：如果受访者不愿回答或者忘记了，在此处分级展开提问] 10,000 /30,000 /50,000 /100,000 /200,000 Yuan 元

GA006_w2 How much did he/she pay the tax and employee social insurance in the past year year? 过去一年，[preload household member name] 缴纳的税和职工社会保险一共有多少钱? ______ (**GA006_w2_1**) Yuan 元 or 或每个月缴多少钱? _____ (**GA006_w2_2**) Yuan/Month 元/月。

GA007 Did [preload household member name] receive any of the following types of individual income in the past year? (check all that apply) 过去一年，[preload household member name] 有没有领到下列收入？(可多选)

1. Pensions (including wages from government institutions and firms, supplemental pension of the firms,and income from such programs as rural pension insurance, Urban residents' pension and commercial pension insurance, new rural social pension insurance and pension subsidy for the oldest old) 退休金或养老金（包括政府机关和事业单位退休金，企业职工基本养老保险，企业补充养老保险，农村、城乡、城镇居民养老保险，商业养老保险，人寿保险，征地养老保险，高龄老人养老补助等）
2. unemployment compensation 失业补助
3. pension subsidy 无保障老人生活补贴
4. Workers' compensation from Industrial Accident Compensation Insurance includes wage-replacement benefits, disability benefits, and survivors' benefits 工伤保险金包括误工补贴、伤残补助、丧葬费等
5. elderly family planning subsidies 独生子女老年补助
6. medical aid 医疗救助
7. other government subsidies 政府发放给个人的其他补助
8. social assistance 社会捐助
9. other income sources 其他收入
10. None of the above 以上均没有 → Skip GA008 跳过 GA008

[F1：“医疗救助”在部分区县试点，救助标准是由各区县根据自身经济情况制定，导致救助标准有高有低。主要三种，一是提供社会医疗救助金，给救助对象以经济补偿；二是给医疗机构一定的经济补贴，使后者直接减免救助对象的部分医疗费；三是由社会医疗救助机构举办专门医疗机构，免费为救助对象提供医疗服务。]

GA008 How much did he/she receive last year? 过去一年一共领了多少钱? ____ (**GA008_b**) Yuan 元 [soft check >10000] or 或每个月领多少钱? ____ (**GA008_c**) Yuan/Month 元/月 [soft check >3000]

PROCEDURE 程序:

If GA007 = 1 and GA008 = DK, ask GA008_bracket. 如果领取了退休金或养老金，但是没回答多少钱，提问 GA008_bracket

GA008_bracket [IWER: If R is unwilling to answer or does not remember, ask unfolding bracket questions here. 访员注意：如果受访者不愿回答或者忘记了，在此处分级展开提问：]
10,000 /30,000 /50,000 /100,000 /200,000 Yuan 元

PROCEDURE 程序:
Skip to next person. 请询问下一个人。

PART 2 HOUSEHOLD AGRICULTURAL INCOME AND EXPENDITURE 家户农业收入与支出

[Intro: Next we will ask some questions about your household agricultural income and expenditure. 下面，我们将问一些有关您家农业收入与支出的问题。]

GB001 Did your household engage in agricultural work (including cropping, forestry, livestock, and fish) last year? 过去一年，除了您和您的配偶，您家其他家庭成员有没有从事种地、管理果树、采集农林产品、养鱼、打鱼、养牲畜等农业活动，或者去市场销售自家生产的农产品？

1. Yes 有
2. No 没有 → Skip to GC001 跳至 GC001

GB002 [IWER: The names of other household members not including respondent and spouse are preloaded from the cover screen information: 访员注意：其他家户成员不包括主要受访者及其配偶，其他家户成员的名单从过滤问卷获得] [preload other household member name] Who engaged in agricultural work in the past year? 过去一年，从事这些农业活动的家户成员有哪些？(可多选)

Crops and forestry products 农林产品

GB003 Did your household engage in cropping or forestry last year? 您家过去一年是否从事了种植业或林业（包括花木、蔬菜和各类农作物的种植）？

1. Yes 是
2. No 否 → Skip to GB007 跳至 GB007

GB004 When was the most recent harvest? 最近的一次收成是在什么时候？ ____2009...2015 (**GB004_1**) Year 年 ____0...12 (**GB004_2**) Month 月
[IWER: Mark the year using four digits. Take down the month as its actual number. For example, write January as "1" not "01", December as "12". If do not remember month, fill "0". 访员注意：用 4 位数表示年，按照实际的月份填写月。例：1 月写作“1”，而不是“01”，12 月写作“12”。如果记不住月份，请填入“0”。]

GB005 What is the total value of all crops and forestry products produced in the past year? 过去一年，您家生产的农产品和林产品加起来一共值多少钱？ ____ (**GB005_1**) Yuan 元 [soft check: 75000 yuan] Among it, what is the value of the crops and forestry products that is home consumed? 其中，自家消费的占多少？ ____ (**GB005_2**)Yuan 元或 __ (**GB005_3**) %

GB005_bracket [IWER: If R is unwilling to answer or does not remember, ask unfolding bracket questions here. 访员注意：如果受访者不愿回答或者忘记了，在此处分级展开提问:] 加起来一共值多少钱？1,000 /3,000 /5,000 /7,000 /10,000 Yuan 元

GB005_w2_bracket [IWER: If R is unwilling to answer or does not remember, ask unfolding bracket questions here. 访员注意：如果受访者不愿回答或者忘记了，在此处分级展开提问:] 自家消费的占多少？1,000 /3,000 /5,000 /7,000 /10,000 Yuan 元

GB006 What was the total cost of producing crops (including vegetables and Chinese herbs) and forestry products in the past year? (including Seeds (including home-used seeds), Fertilizer, Organic fertilizer, Pesticide, Plastic sheets, Hiring labor (including with machine or animals), Land rents, Rents (excluding land rents), Irrigation, Fuel, Transportation, Processing, Marketing (including packaging, management fee)) 过去一年，您家为了农业生产（包括种植粮食作物、蔬菜和中药材等）和林业生产（包括种植蘑菇、木耳等林下作物、种植茶叶），总共投入了多少钱？[包括种子（含自家留种的价值）、化肥、农家肥、农药、塑料薄膜、雇工费（包括其使用的机器和役畜）、土地租金、除地租以外的其他租金（例如：租用收割机）、灌溉费、燃料、运输费、加工费、市场费用（包括包装费，管理费等）] ____ Yuan 元 [soft check: 50,000 yuan]

GB006_bracket [IWER: If R is unwilling to answer or does not remember, ask unfolding bracket questions here. 访员注意：如果受访者不愿回答或者忘记了，在此处分级展开提问:] 300 /600 /1,000 /2000 /5,000 Yuan 元

Livestock and fisheries 牲畜和水产品

GB007 Did your household grow any livestock or aquatic life last year? 您家过去一年养过牲畜（家禽、家畜如鸡、鸭、牛、猪、羊等都算）或者水产品吗？

1. Yes 是
2. No 否 → Skip to GC001 跳至 GC001

GB008 What is the current value of all livestock (including chicken, duck, cattle, pig, sheep, etc.) and aquatic life? 现在，您家所有的家禽、家畜（包括鸡、鸭、牛、猪、羊等），水产品加起来一共值多少钱？ ____ Yuan 元 [soft check: 100,000 yuan]

GB008_bracket [IWER: If R is unwilling to answer or does not remember, ask unfolding bracket questions here. 访员注意：如果受访者不愿回答或者忘记了，在此处分级展开提问:] 500 /1,500 /3,000 /4,500 /9,000 Yuan 元

GB009 What was the value of all livestock and aquatic life at this time last year? 去年这个时候，您家拥有的牲畜及水产品一共值多少钱？ ____Yuan 元 [soft check: 100,000 yuan]

GB009_bracket [IWER: If R is unwilling to answer or does not remember, ask unfolding bracket questions here. 访员注意：如果受访者不愿回答或者忘记了，在此处分级展开提问:] 500 /1,500 /2,500 /4,000 /8,000 Yuan 元

GB010 How much did you spend purchasing new livestock and aquatic life in the past year? 过去一年，你们家买牲畜及水产品一共花了多少钱？____ Yuan 元 [soft check: 50,000]

GB011 What was the value of all livestock and aquatic life that were sold or consumed in the past year? 过去一年，你们家卖出去的和自家消费的牲畜及水产品加起来一共值多少钱?____ (**GB011_1**) Yuan 元 [soft check: 100,000 yuan] Among it, what is the amount or percent consumed at your home? 其中，自家消费的占多少？____ (**GB011_2**) Yuan 元或 ___ (**GB011_3**) %

GB011_bracket [IWER: If R is unwilling to answer or does not remember, ask unfolding bracket questions here. 访员注意：如果受访者不愿回答或者忘记了，在此处分级展开提问:] 加起来一共值多少钱？200 /900 /1,500 /2,500 /5,000 Yuan 元

GB011_w2_bracket [IWER: If R is unwilling to answer or does not remember, ask unfolding bracket questions here. 访员注意：如果受访者不愿回答或者忘记了，在此处分级展开提问:] 其中，自家消费的占多少？200 /900 /1,500 /2,500 /5,000 Yuan 元

GB012 What was the value of all livestock products produced (including the self consumption value) in the past year, including milk, wool (including cashmere, sheep or goat skin), and eggs? 过去一年，你们家养的牲畜生产出来的副产品（比如鸡蛋、牛奶、羊毛、羊绒、羊皮），加起来一共值多少钱（包括所有卖出去的和自家消费的）？____ (**GB012_1**) Yuan 元 [soft check: 50,000 yuan] Among it, what is the amount or percent consumed at your home? 其中，自家消费的占多少？____ (**GB012_2**) Yuan 元或 ___ (**GB012_3**) % [soft check: 50,000 yuan]

GB012_bracket [IWER: If R is unwilling to answer or does not remember, ask unfolding bracket questions here. 访员注意：如果受访者不愿回答或者忘记了，在此处分级展开提问:] 一共值多少钱（包括所有卖出去的和自家消费的）？100 /200 /300 /500 /1,000 Yuan 元

GB012_w2_bracket [IWER: If R is unwilling to answer or does not remember, ask unfolding bracket questions here. 访员注意：如果受访者不愿回答或者忘记了，在此处分级展开提问:] 其中，自家消费的占多少？100 /200 /300 /500 /1,000 Yuan 元

GB013 What was the cost of producing livestock and aquatic life in the past year, including the value of all feed, medicine, pasture fees, animal pens, wages, etc. 过去一年，养这些牲畜及水产品一共花了多少钱，包括喂养费、医药费、放牧费、畜舍栅栏费、雇工费等？____ Yuan 元

[soft check: 50,000 yuan] [soft check: "reported raising livestock but no evidence of such activity" if GB008 = 0 and GB009 = 0 如果受访者饲养过牲畜或水产品，但 GB008 = 0 且 GB009 = 0，则进行检查]

PART 3 Self-employed Activities 个体经营或开办私营企业

GC001 Did your household members engage in any self-employed activities last year? 过去一年，您家是否有家户成员从事某些个体经营或开办私营企业？

1. Yes 是
2. No 否 → Skip to GD001 跳至 GD001

GC002 How many types of activities did your household members participate in the past year? ____ Activities. 过去一年，您家家户成员从事几项个体经营活动或开办几家私营企业？

PROCEDURE 程序:
For each self-employed activity in GC002, ask GC003 - GC005. 针对每一项个体经营活动或私营企业，询问 GC003 - GC005

GC003 Who engaged in this self-employment businesse in the past year? 过去一年，从事这项个体经营或私营企业的家户成员有哪些？
[IWER: All the names of household members are preloaded from the cover screen information 访员注意：所有家户成员名单（包括主要受访者及其配偶）要从过滤问卷获得] (可多选)

GC004 Which types of activities? 这项活动属于什么行业？

1. Services (cooking, sewing, private clinic etc.) 服务业（烹饪、缝纫、私人诊所等）
2. Transportation 交通运输
3. Construction 建筑业
4. Mining 采掘
5. Processing production 加工生产业
6. Business 商业
7. Others, please specify 其他，请注明 ____(**GC004_1**)

GC005 Not including fixed capital costs, what is your best estimate of the net income earned from this activity by your household members last year? [If the activity was conducted jointly with non-household members, report only the net income earned by household members. Remember to consider the following types of costs: energy, housing or equipment rental, raw materials, transportation, marketing, wages, taxes or fees.] 不包括固定资本成本，您能否精确估计一下您家过去一年从这项活动净赚多少钱？如果此项经营活动有非家户成员参与，只计算家户成员的净收入。别忘了考虑下列各种成本：能源、住房和设备租用费、原材料、交通费、营销、工资、税收和杂费。____ Yuan 元
[soft check: 500,000 yuan]

GC005_bracket [IWER: If R is unwilling to answer or does not remember, ask unfolding bracket questions here. 访员注意：如果受访者不愿回答或者忘记了，在此处分级展开提问:]
5,000 /10,000 /50,000 /100,000 /200,000 Yuan 元

PART 4 HOUSEHOLD PUBLIC TRANSFER INCOME 家户政府转移支付收入

[We ask the public transfers received by the households (with household as the unit). Public transfers have characteristic of welfare, such as Wubaohu Subsidy and Tekunhu Subsidy gived by government. 这部分询问家户从政府所得的各种转移支付收入。政府转移支付具有福利支出的性质，如政府给五保户和特困户的补助金等。这里询问的是以家户为单位获得的转移支付。]

GD001 How much Dibao assistance did your household receive last year? (if not applicable, fill in 0 yuan). 您家过去一年得到低保了吗？得到多少？（如果没有，请填 0） ____ Yuan 元

GD002 Did your household receive any of the following government subsidies in the past year? (check all that apply) 您家过去一年有没有收到下列政府补助？（可多选）
[soft checks for each category: 20,000 yuan 对每项进行检查：20,000 元。]

1. Reforestation 退耕还林: how much? 有多少？ ____ (**GD002_1**) Yuan 元
2. Agricultural subsidies 农业补助: how much? 有多少？ ____ (**GD002_2**) Yuan 元
3. Wubaohu (targets low-income, blind, disabled, aged persons, and young persons that have no means to support themselves. 五保户补助金（用来补助那些无法自力更生的低收入者、盲人、身体残疾者、老人和小孩: how much? 有多少？ ____ (**GD002_3**) Yuan 元
4. Tekunhu 特困户补助: how much? 有多少？ ____ (**GD002_4**) Yuan 元
5. Work injury subsidies to the immediate family members 工伤人员供养直系亲属抚恤金 how much? 有多少？ ____ (**GD002_5**) Yuan 元
6. Emergency or disaster relief (jiujikuan, jiuzaikuan) last year? 突发事件或重大灾难之后的补助（即救济金、赈灾款）（包括实物形式）: how much? 加上实物救助的价值一共有多少？ ____ (**GD002_6**) Yuan 元
7. Other, please specify 其他补助，请注明 ____(**GD002_other**): how much? 有多少？ ____ (**GD002_7**) Yuan 元
8. None 没有收到任何政府补助 → Skip to GD003 跳至 GD003

GD003 Did your household receive any income from the following sources in the past year? (check all that apply) 您家过去一年有没有收到下列哪种捐助或补偿？（可多选）

1. Donations from the society (including cash, and items like food, clothing, etc.) 社会捐助（包括现金，食品、衣服等）: how much? 加上实物的价值一共有多少？ ____ (**GD003_1**) Yuan 元 [soft check: 20,000 yuan]
2. Compensation for land seizure last? 征地补偿金: how much? 有多少？____ (**GD003_2**) Yuan 元 [soft check: 100,000 yuan]
3. Compensation to pulling down your house or apartment last year? 住房拆迁补偿: how much? 有多少？ ____(**GD003_3**) Yuan 元 [soft check: 100,000 yuan]
4. None 没有收到任何捐助或补偿

PART 5 HOUSEHOLD LIVING EXPENDITURE 家户生活支出

[访员注意：如果过去一年中，主要受访者与配偶一起居住，则本部分只需要生成一份，询问家庭受访者；如果主要受访者和配偶中的一方因打工等原因长期在外居住（一个月及以上，非分居、分家等情

况)，本部分需要生成两份问卷，分别归于两个受访者的个人问卷中。如果家庭成员分别跟随两个人生活，需要将受访者与跟他们一起生活的家庭成员加载到一个列表中。]

GE001 We wish to know your family food expenditure for the last week. Are you the primary person who purchases food for the household? 我们想知道您家最近一周的食品支出，您负责为家里购买食品吗？

1. Yes 是 → skip to GE004 跳至 GE004
2. No 否

GE002 Who is the primary person purchasing food for the household? 谁负责为您家购买食品？

[CAPI: Preload all the HHmember list 列出所有家户成员的名单]

1. HHmember list 家户成员列表
2. Other children 其他子女（列出所有不在家户成员名单中的子女并从中选取）
3. Nanny 保姆
4. Neighbor 邻居
5. Other, please specify 其他

[IWER: If possible, the primary person who purchases food for the household should answer the questions about expenditures FE003-FE009] [访员注意：如果可能的话，由负责为该家庭购买食品的人来回答生活支出问题]

GE004 In the past week, how many people usually ate meals together in your household (not including guests)? 最近一周，您家里一般有几口人吃饭（不包括客人）？ __ Persons 人 [soft check: 10]

GE005 Last week how many meals did you provide to guests? 最近一周，您家的客人在您家吃了几顿饭（按人次计算）？ ____ Meals 人次？ [soft check: 100]

[Intro: The next questions are about your household living expenditure, including your household members ‘(preloaded names of household members) living expenditure. If one attends school/work outside and comes home almost every week, GE006 –GE008 includes his/her expenditureon food and meals outside. If one attends school/work outside but not come home every week, GE006 –GE008 excludes his/her expenditureon food and meals outside.] [引语：下面问题是关于您家的生活支出的。这些支出包括所有家户成员的生活支出。如果有人在外上学/上班，但周末回家，他们在外上学/上班发生的开支算在内；如果有人在外打工等，周末不回家，他们在外的生活开支不算在内。]

GE006 In the past week, how much did your household spend on food (excluding eating out expenditure, alcohol, cigarettes, cigars and tobacco expenditure)? 最近一周，您家花了多少钱购买食品，不包括外出就餐、购买香烟、酒水等？ ____ Yuan 元 [soft check: 6000 yuan]

GE006_W2 Does your household produce agricultural products yourself (including plants, meat, eggs, aquatic lives, oil, vegetables and fruits, cigaretts and wine, drinks and milk products, produced food, seasonings, etc.)? 您家自己生产农产品吗（包括粮食作物、肉类、蛋类、水产品、油、蔬菜水果、烟酒、饮料及乳制品、加工食品、调料等）？

1. Yes 生产
2. No 不生产 → skip to GE007 跳至 GE007

GE006_W2_1 In the past week, what was the market value of the food that members of the household consumed that you grew yourselves? 最近一周，您家里人消费的自家生产的农产品在市场上卖的话值多少钱？ ____ Yuan 元

GE007 Among it, how much did your household spend on eating out? 最近一周，您家花了多少钱外出就餐等？ ____ Yuan 元 [soft check: 3000 yuan]

GE008 Among it, how much did your household spend on alcohol, Cigarettes, cigars and tobacco? 最近一周，您家花了多少钱购买香烟、酒水等？ ____ Yuan 元 [soft check: 3000 yuan]

GE009 Please tell me the expenditure last month for your household for the following items. 下面我们想了解您家过去一个月在以下各项消费中的支出。

[soft check for each category: 5000 yuan] [IWER: fill in 0 if no corresponding expenditure; fill in -9999 if the respondent cannot recall the expenditure. 访员注意：没有相应项支出用 0 元表示，记不清则请受访者估计一个数字，如果实在无法估计，该支出用-9999 表示]

1. Communication fees (including post, internet usage, telephone and cell phone usage) 邮电、通讯支出（包括电话、手机、上网、邮寄等） ____ (**GE009_1**) Yuan 元
2. Utilities: Water and electricity 水费、电费 ____ (**GE009_2**) Yuan 元
3. Fuels (including gas, coal, etc.) 燃料费（包括煤炭、煤制品、柴草、木炭、天然气、液化气等） ____ (**GE009_3**) Yuan 元
4. Fees for Matron, housekeepers and servants 保姆、小时工、佣人等的支出 __ (**GE009_4**) Yuan 元
5. Local Transportation 在当地的交通费 ____ (**GE009_5**) Yuan 元
6. Household items and personal toiletries that are used daily plus beauty treatments (e.g., detergent, soap, toothpaste, toothbrush, cosmetics, beauty salon, etc.) 日用品（包括洗漱用品、家居用品、厨卫用品、装饰用品等） ____ (**GE009_6**) Yuan 元
7. Entertainment (including fees to buy books, newspapers, VCCs, DVDs, going to cinema and bars) 文化娱乐支出（包括书报杂志、光盘、影剧票、歌舞厅和网吧等） ____ (**GE009_7**) Yuan 元

GE010 In the last year how much did your household spend on the following items? 下面我们想了解您家过去一年在以下各项消费中的支出。

[IWER: fill in 0 if no corresponding expenditure; fill in -9999 if the respondent cannot recall the expenditure. 访员注意：没有相应项支出用 0 元表示，记不清则请受访者估计一个数字，如果实在无法估计，该支出用-9999 表示。] [soft check: >=100,000 yuan]

1. Clothing and bedding 衣着消费 ____ (**GE010_1**) Yuan 元
2. Long distance traveling expenses 家庭的旅游支出 ____ (**GE010_2**) Yuan 元
3. Heating(centrally heated) 家庭的取暖费支出 (指集中供暖)____ (**GE010_3**) Yuan 元
4. Furniture, consumption of durable goods and electronics, includes refrigerator, washing machine, TV, computers and expensive instruments like pianio. 家具、耐用消费品及电器的支出（包括电冰箱、洗衣机、电视、电脑和高档乐器如钢琴等）____ (**GE010_4**) Yuan 元
5. Education and training(including tuition, training fees, etc.) 教育和培训支出（包括学杂费、培训费等）____ (**GE010_5**) Yuan 元
6. Medical expenditure 医疗支出（包括直接或间接。注：间接医疗支出，指因为医疗而产生的交通费、营养费、家人陪护花费等。）____ (**GE010_6**) Yuan 元
7. Fitness expenditures 保健费用（包括健身锻炼及产品器械、保健品等）____ (**GE010_7**) Yuan 元
8. Beauty (including make-ups, facials, massages, etc.) 美容支出（包括化妆品、美容护理、按摩等）____ (**GE010_8**) Yuan 元
9. Automobiles 购买汽车 ____ (**GE010_9**) Yuan 元
10. Purchase, Maintenance and repair (of transportation vehicles, appliances, communication products, etc.) 各种交通工具（如自行车、电动自行车等，不包括汽车）、通讯工具（如电话、手机等）的购买、维修及配件费用 ____ (**GE010_10**) Yuan 元
11. Property management fees (including parking fee) 物业费(包括车位费)____ (**GE010_11**) Yuan 元
12. Taxes and fees turned over to the government 上交给政府相关部门的税费和杂费（不包括所得税）____ (**GE010_12**) Yuan 元
13. Donations to the society (including cash, and items like food, clothing, etc.) 社会捐助支出（包括现金，食品、衣服等）____ (**GE010_13**) Yuan 元

GE011 How often did the respondent receive assistance in answering section Household income and expenditure? 受访者填写该部分问卷时是否求助？

[IWER: If it is answered by a proxy, please record the respondent's reaction. 访员注意：如果是协助回答，请记录受访者的反应。]

1. Never 从未
2. A few times 偶尔几次
3. Most or all of the time 大多数时间

HA HOUSEHOLD ASSETS 家户资产

[IWER: This section is asked of the family respondent. Do not allow a proxy respondent to answer the entire section. 访员注意：这部分要问家庭受访者，不允许完全请别人代答。]

PART 1 Current Residence 第一部分：现有住宅

The following questions pertain to your current residence. 下面是关于您现住宅的问题。

HA000_W2_0 访员填写：受访者当前居住住宅类型是？
1. 一般居民住宅
2. 养老院/敬老院 → skip to HA026_W2 Branchpoint 跳至 HA026_W2 Branchpoint
3. 医院 → skip to HA026_W2 Branchpoint 跳至 HA026_W2 Branchpoint
4. 干休所 → skip to HA026_W2 Branchpoint 跳至 HA026_W2 Branchpoint
5. 其他机构 → skip to HA026_W2 Branchpoint 跳至 HA026_W2 Branchpoint

HA000_W2_1 BRANCHPOINT :
IF THIS IS A NEW INTERVIEWED HOUSEHOLD, GO TO HA001 如果是新的受访户，请询问HA001.
IF THIS IS A REINTERVIEWED HOUSEHOLD, GO TO HA000_W2_1 如果是老受访户，请询问HA000_W2_1.

HA000_W2_1 这所房子是上次访问时 [ZIWTime]，您居住的房屋 [加载上一轮调查时现有住宅的特征] 吗？
1. Yes 是 → skip to HA001_W2 跳至 HA001_W2
2. No 否

HA001 When did your household start to live at your current residence? 您家何时开始住进现在的房子？ ____1900...2015 Year 年
[IWER: Mark the year using four digits. 访员注意：用 4 位数表示年。]

HA001_W2 What is the construction area of the house? 这房子的建筑面积是多大？ ____ m^2 平方米 [soft check<10 or >500]

HA002 Do you pay rent for your current residence? 您家现在住的房子要付租金吗？
1. Yes 要
2. No 不要 → skip to HA005 跳至 HA005

HA003 How much rent do you pay each month? 如果要付租金，一个月付多少钱？_____ Yuan/Month 元/月 [soft check: <100, >10,000 yuan]

HA006 How much of the rent was paid by a housing subsidy from the employer of a household member? which household member? 您家户成员的单位提供多少住房补贴来支付租金？_____ (**HA006_1**)yuan 元 [soft check >10,000 yuan] ,哪几个家户成员？____ (**HA006_2**) [preloaded list 从前面提取家户成员名单]

HA004 Did you pay less than the market rental value? 您家支付的租金是否低于市场价？
1. Yes 是
2. No 否 → skip to HA007 跳至 HA007

HA005 If you rented the same housing unit from the market, what is the rent per month you would have to pay? 如果从市场上租同样的房子，每月的租金会是多少？ ____ Yuan/Month 元/月 [soft check: <100, >10,000 yuan]

HA007 Who owns your current residence? 您家现在的房子归谁所有?

1. 完全归家户成员所有
2. 部分归家户成员所有
3. 完全不归家户成员所有 → skip to HA010 跳至 HA010

HA007_W2_1 [CAPI: Ask this question only when the earlier records show that ONE of the respondent (main respond OR the spouse passed away in the last two years 只有从前面问卷中,发现主要受访者与其配偶中,有一人去世的情况,才问此问] Has the owndership status changes following the death of [preload the name of the deceased main respondent or spouse] 在过去两年内，有没有因为 [加载去世的主要受访者或配偶的名字] 的去世，而导致现在这所房子的所有权状况发生改变?

1. Yes 有
2. No 没有 → skip to HA008 跳至 HA008

HA007_W2_2 在去世前，[加载去世的主要受访者或配偶的名字] 拥有该房产的比重是 __ %.

HA007_W2_3 [加载去世的主要受访者或配偶的名字] 拥有的那部分房产，是如何处置的? 继承的比重分别是?

[注意，这里问的是去世受访者拥有的产权部分的继承比重分配。比如去世受访者拥有 60 他将自己产权的 5050 的继承情况，并未询问家庭成员目前拥有的产权情况。]

[Hard check: 以下各项总和不能超过 100]

1. 由配偶继承，___ **(HA007_w2_3_1[1])**%
2. 子女、女婿、儿媳，___ **(HA007_w2_3_1[2])**%
3. 兄弟姐妹，___ **(HA007_w2_3_1[3])**%
4. 其他亲戚，___ **(HA007_w2_3_1[4])**%
5. 孙子/女，___ **(HA007_w2_3_1[5])**%
6. 朋友，___ **(HA007_w2_3_1[6])**%
7. 慈善机构，___ **(HA007_w2_3_1[7])**%
8. 其他 ___ **(HA007_w2_3_1[8])**%

PROCEDURE 程序:
如果继承人包含了子女（HA007_W2_3 = 2），提问 HA007_W2_4 和 HA007_W2_5

HA007_W2_4 是哪些子女? [Load 子女名称让 proxy 选择，可多选]。

HA007_W2_5 [加载 HA007_W2_4 中的子女姓名，逐个询问] 继承的产权，占 [加载去世的主要受访者或配偶的名字] 生前拥有产权比重的 ___ %?

PROCEDURE 程序:
如果继承人包含了孙子女（HA007_W2_3 = 5），提问 HA007_W2_6 和 HA007_W2_7

HA007_W2_6 孙子女是哪些子女的孩子? [Load 子女名称让 proxy 选择，可多选]。

HA007_W2_7 [加载 HA007_W2_6 中的子女姓名，逐个询问] 的孩子继承的产权，占 [加载去世的主要受访者或配偶的名字] 生前拥有产权比重的 __ %?

HA008 Which household member(s) own the house? (preloaded names of household members) 哪一个/些家户成员拥有这处房子？ ____ [从前面提取家户成员名单]

HA009 What share of the house is owned by [preloaded names of household members]?（加载 HA008 的答案）拥有产权的比重是？ ____ 0.00...100.00 % [hard check: range 0-100]

PROCEDURE 程序:
Skip to HA011. 跳至 HA011

HA010 Which non-household members own all or part of your current residence? (circle all that apply) 您家现在的房子是哪些非家户成员的？（可多选）

1. Working unit of household member, 家户成员单位的，which household member? ____ (**HA010_1**) 哪一个家户成员的单位 [preloaded list] [从前面提取家户成员名单]
2. Government indemnificatory housing 政府保障性住房
3. Child(non- household member) of main respondent or spouse 主要受访者（或其配偶）的非家户成员的孩子, which child? 哪个孩子？ ____ (**HA010_2**) [preloaded list] [从前面提取孩子的名单]
4. Parent(non- household member) of main respondent or spouse 主要受访者（或其配偶）的非家户成员的父母 ____ (**HA010_3**) [preload list] [从前面提取父母的名单]
5. Nonresident other relatives 没在一起居住的其他亲戚
6. Friends 朋友
7. Other 其他人

PROCEDURE 程序:
If HA007 = 3, skip HA011 and HA012. 如果您家现在的房子完全不归家户成员所有，跳过HA011和HA012。

HA011 What is the present market value of your house? Or, what is the present market value of a similar housing unit within its neighborhood? 这所房子当前的市场价是多少？或者说，这房子附近类似的房子当前的市场价是多少？
Total price 总价格 ____ (**HA011_1**) 10000 Yuan 万元 [soft check < 10, > 500]
Or unit price 或单位价格 ____ (**HA011_2**) 1000 Yuan/m^2 千元/平方米
[IWER: Skip to HA013 if R answered HA011. If not, ask unfolding brackets. 访员注意：如果答卷人回答了HA011，跳至 HA013，否则，提问分级展开问题。]

HA012 [IWER: If R is unwilling to answer or does not remember, ask unfolding bracket questions here. 访员注意：如果受访者不愿回答或者忘记了，在此处分级展开提问]
20,000/50,000/100,000/200,000/500,000 Yuan 元

HA026_W2 BRANCHPOINT:
新受访者，跳至 HA027。
老受访者，如果 HA000_W2_1 = 1，跳至 PART 2.

[我们的数据显示，您家现在居住的住宅与两年前不同。我们想询问一下上次访问时您家所居住住宅的处置情况（HA026_W2_1[25]-HA026_W2_14_bracket[25]）
其他房产（HA026_W2_1-HA026_W2_14_bracket）为一个循环]

HA026_W2_1 您上次访问时回答的 [下载上次访问时的相关住宅信息] 住宅是怎么处置的？

1. 现在自己住着 → 结束本循环
2. Empty 空着 → 结束本循环
3. Sold 出售
4. Rented out 出租 → 结束本循环
5. Being torn down 被拆迁 → skip to HA026_W2_6 跳至 HA026_W2_6
6. My child(ren) stay in it 子女住着 → skip to HA026_W2_9 跳至 HA026_W2_9
7. My parent(s) stay in it 父母住着 → skip to HA026_W2_9 跳至 HA026_W2_9
8. Others (relatives, friends) stay in it 给其他人 (亲戚, 朋友) 居住 → skip to HA026_W2_9 跳至 HA026_W2_9
9. Give to others as gift 赠予他人 → skip to HA026_W2_12 跳至 HA026_W2_12
10. Last residence is rented, no longer rent it 上次住宅是租来的，现已不再租用 → 结束本循环
11. Other, please specify 其它，请注明 ____ (**HA026_W2_1a**) → 结束本循环
12. Last record is wrong, the mentioned residence is not ours 上次记录有误，提及的住宅不是我家的 → 结束本循环

HA026_W2_2 When did you sell that house? ____ Year __ Month 您何时出售的该住宅？____ (**HA026_W2_2_1**) 年 __ (**HA026_W2_2_2**) 月

HA026_W2_3 What is the net income you got from selling the house, excluding relative fees? 扣除相关费用，您出售该住宅后获得的净收入是多少？Total price 总价格 ____ 10,000 Yuan 万元 [soft check < 0.1, > 500]

HA026_W2_4 [IWER: If R is unwilling to answer or does not remember, ask unfolding bracket questions here. 访员注意：如果受访者不愿回答或者忘记了，在此处分级展开提问] 20,000/50,000/100,000/200,000/500,000 yuan 元

HA026_W2_5 How did you distribute the income from selling this house? (Can be multiple choice, ask the respondent to fill in corresponding percentages.) 您如何处理卖房所得的收入？[下列选项可以多选，有下列处置方式的请受访者回答相应比例]

1. Deposits in bank account 存入银行账户，占售房总收入的 ___ (**HA026_W2_5a**) % of total income
2. Everyday living expenses 用于日常生活支出，占售房总收入的 ___ (**HA026_W2_5b**) % of total income
3. Buying new houses 用于购买新房，占售房总收入的 ___ (**HA026_W2_5c**) % of total income
4. Medical expenditure 用于医疗支出，占售房总收入的 ___ (**HA026_W2_5d**) % of total income
5. Other important expenses 用于其他重大支出，占售房总收入的 ___ (**HA026_W2_5e**) % of total income
6. Trasfer to Children 赠予儿女，占售房总收入的 ___ (**HA026_W2_5f**) % of total income

7. Transfer to parents 赠予父母，占售房总收入的 ___ (**HA026_W2_5g**) % of total income
8. Transfer to relatives 赠予亲戚，占总收入的 ___ (**HA026_W2_5h**) % of total income
9. Other, please specify 其他，请注明 ___ (**HA026_W2_5i**)，占总收入的 ___ (**HA026_W2_5j**) % of total income

[CAPI: 结束本循环]

HA026_W2_6 Did you receive any compensation for tearing down the house? 这处房屋被拆迁，您家是否获得拆迁补偿款？
1. Yes 是
2. No 否 → 结束本循环

HA026_W2_7 When did you receive the compensation for tearing down the house? 您家何时获得了拆迁补偿款？ ____Year 年 (**HA026_W2_7_1**) __ Month 月 (**HA026_W2_7_2**)

HA026_W2_8 What is the total amount of the compensation? 您家获得的拆迁补偿款的总额是多少？ ____ 10,000 Yuan 万元

[CAPI: 结束本循环]

HA026_W2_9 Have those who live in your house pay you any cash, or provide you in-kind payment? 住您房屋的人，有没有给您现金或者实物的补偿？
1. Yes 是
2. No 否 → 结束本循环

HA026_W2_10 How did those who live in your house pay you the cash or the in-kind payment? 住您房屋的人是如何给您现金或者实物的补偿的？
1. One lump-sum payment 一次性补偿
2. Monthly payment 每月支付
3. Irregular payment over the year 不定期补偿

HA026_W2_11 What is the total amount did those who live in your house pay you in the past year? 过去一年，住您房屋的人给您的补偿是 ____ (**HA026_W2_11a**) Yuan 元？Among it, cash is 其中，现金 ____ (**HA026_W2_11b**) Yuan 元，in-kind payment is 实物值 ____ (**HA026_W2_11c**) Yuan 元？

[CAPI: 结束本循环]

HA026_W2_12 Have those who got your house as gifts pay you any cash, or provide you in-kind payment? 获得您赠予房产的人，有没有给您现金或者实物的补偿？
1. Yes 是
2. No 否 → 结束本循环

HA026_W2_13 How did those who got your house as gifts pay you the cash or the in-kind payment? 获得您赠予房产的人是如何给您现金或者实物的补偿的？

1. One lump-sum payment 一次性补偿
2. Monthly payment 每月支付
3. Irregular payment over the year 不定期补偿

HA026_W2_14 What is the total amount did those who got your house as a gift pay you in the past year? 过去一年，获得您赠予房产的人，给您的补偿是 ____ (**HA026_W2_14a**) Yuan 元？Among it, cash is 其中，现金 ____ (**HA026_W2_14b**) Yuan 元，in-kind payment is 实物值 ____ (**HA026_W2_14c**) Yuan 元？

[CAPI: 循环结束]

PROCEDURE 程序:
老受访者，请跳过 HA027 - HA028。

HA027 Excluding the house in which you live, do you or members of your household own any other residential properties? 除了您家现在的住房以外，您和/或您的配偶是否拥有其他房产（是否拥有以您和/或您的配偶是否拥有产权为准）？

1. Yes 是
2. No 否 → skip to HA052 跳至 HA052

HA028 How many other housing units do you or members of your household currently own? 您和/或您的配偶还有几处其他的房产？ __ 0...10 处

PART 2 Other Residences 第二部分：其他房产

[For every other housing unit owned by main respondents and his/her spouse, ask the following questions, 对主要受访者和配偶拥有的其他的每处房产，询问以下的问题：]

HA029_W2 BRANCHPOINT:
IF THE HOUSEHOLD IS A NEW INTERVIEW HOUSEHOLD, THEN SKIP TO HA029. 如果是新家户，跳至 HA029
IF THE HOUSEHOLD IS A RE-INTERVIEW HOUSEHOLD, SKIP TO HA029_W2. 如果是老家户，回答 HA029_W2

HA029_W2 In the past two years since [preload the year of last wave] Year [preload the month of last wave] month, have your household obtained (purchased/built/inherited/received as gift/got new house due to tearing down) any new houses? 自从上次访问 [ZIWTime] 以来，您家有没有得到（购买/新建/继承/受赠/拆迁换房等）新的房产（前面回答过的现有住宅除外）？

1. Yes, obtained 是，得到了 __ 0...10 (**HA029_W2_1**) houses 处
2. No 否 → skip to HA051_W2_1 Branchpoint 跳至 HA051_W2_1 Branchpoint

[CAPI：HA029-HA051 为一个循环]

HA030 Who owns this residence? 这所房子归谁所有？

1. Owned completely by you and your spouse. 完全归我和配偶所有
2. Owned partly by you and your spouse. 部分归我和配偶所有

HA030_W2_1 [CAPI: Ask this question only when the earlier records show that one of the respondent (main respond or the spouse passed away in the last two years 只有从前面问卷中，发现主要受访者与其配偶中，有一人去世的情况，才问此问。] Has the owndership status changes following the death of [preload the name of the deceased main respondent or spouse]? 在过去两年内，有没有因为 [加载去世的主要受访者或配偶的名字] 的去世，而导致现在这所房子的所有权状况发生改变？

1. Yes 有
2. No 没有 → skip to HA031 跳至HA031

HA030_W2_2 在去世前，[加载去世的主要受访者或配偶的名字] 拥有该房产的比重是 __ %.

HA030_W2_3 [加载去世的主要受访者或配偶的名字] 拥有的那部分房产，是如何处置的？继承的比重分别是？ [注意，这里问的是去世受访者拥有的产权部分的继承比重分配。比如去世受访者拥有 60% 的产权，他将自己产权的 50% 给了配偶，另外 50% 平均分配给两个儿女。那么这里要填的比重分别是配偶 50%，子女 50%。]

[Hard check: 以下各项总和不能超过 100]

1. 由配偶继承，___ (**HA030_w2_3_1[1]**)%
2. 子女、女婿、儿媳，___ (**HA030_w2_3_1[2]**)%
3. 兄弟姐妹，___ (**HA030_w2_3_1[3]**)%
4. 其他亲戚，___ (**HA030_w2_3_1[4]**)%
5. 孙子/女，___ (**HA030_w2_3_1[5]**)%
6. 朋友，___ (**HA030_w2_3_1[6]**)%
7. 慈善机构，___ (**HA030_w2_3_1[7]**)%
8. 其他，___ (**HA030_w2_3_1[8]**)%

PROCEDURE 程序:
如果继承人选包含了子女（HA030_W2_3 =2），询问 HA030_W2_4 和 HA030_W2_5

HA030_W2_4 是哪些子女？[Load 子女名称让 proxy 选择，可多选]

HA030_W2_5 [加载 HA030_W2_4 中的子女姓名，逐个询问] 继承的产权，占 [加载去世的主要受访者或配偶的名字] 生前拥有产权比重的 __ %？

PROCEDURE 程序:
如果继承人选包含了孙子女（HA030_W2_3 =5），询问 HA030_W2_6 和 HA030_W2_7

HA030_W2_6 孙子女是哪些子女的孩子？[Load 子女名称让 proxy 选择，可多选]。

HA030_W2_7 [加载 HA030_W2_6 中的子女姓名，逐个询问] 的孩子继承的产权，占 [加载去世的主要受访者或配偶的名字] 生前拥有产权比重的 __ %？

HA031 Who owns the house? [preloaded names of main respondents and his/her spouse] 您和您的配偶谁拥有这处房子？____ [加载受访者和配偶姓名]

HA032 What share of the house is owned by [preloaded names of main respondents and his/her spouse]? [加载 HA031 的回答] 拥有产权的比重是？_____ %0.00...100.00% [hard check: range 0-100]

HA034 What is the present market value of your house? Or, what is the present market value of a similar housing unit within its neighborhood? 当前的市场价是多少？或者说，这房子附近类似的房子当前的市场价是多少？Total price 总价格 ____ (**HA034_1**) 10,000 Yuan 万元 [soft check <10, >500] or unit price 或单位价格 ____ (**HA034_2**) 1,000Yuan/m^2 千元/平方米 [soft check<1, >25]
[CAPI: Skip to HA036 if R answered HA034. If not, ask unfolding brackets. 如果答卷人回答了HA034，跳至 HA036，否则，提问分级展开问题]

HA035 [IWER: If R is unwilling to answer or does not remember, ask unfolding bracket questions here. 访员注意：如果受访者不愿回答或者忘记了，在此处分级展开提问]
20,000/50,000/100,000/200,000/500,000 Yuan 元

HA039 How was this housing unit obtained? 房子的来源是什么？
1. Purchased from market 是从市场买来的
2. Purchased from working unit of respondent or spouse 从受访者或其配偶的单位买来的，谁的单位 ____ (**HA039_1**)？[preloaded list] [从前面提取受访者或其配偶的名单]
3. Purchased by child of main respondent or spouse, which one? 由主要受访者（或其配偶）的孩子购得，哪一个 ____ (**HA039_2**) [preload list] [提取孩子名单]
4. Purchased by parents of main respondent or spouse, of who (main respondent or spouse) ? 由主要受访者（或其配偶）的父母购得，谁的（主要受访者还是其配偶的）父母 ____ (**HA039_3**)
5. Purchased from Other relatives 从其他亲戚那里买的
6. Self-built 自建 → Skip to HA041 请跳至 HA041
7. Inherited, bequeathed, or given 继承（如祖业）或受赠与 → Skip to HA041 请跳至 HA041
8. Received home as compensation for demolition of old home, 拆迁换房 → Skip to HA041 请跳至HA041
9. Other 其他 → Skip to HA045 请跳至 HA045

HA040 Was it purchased at market price, subsidized by working unit, or as purchased as economical housing? 这房子是以市场价买、单位优惠价格买的、或者是经济适用房？
1. Market price 以市场价购买
2. Subsidized by working unit 单位优惠价格买的
3. Economic housing 经济适用房
4. Other 其他

HA041 When did you purchase/build/inherit it? 这所房子是什么时候买/建/换/继承/受赠的?

____ 1900...2015 Year 年

[IWER: Mark the year using four digits. 访员注意: 用 4 位数表示年。]

HA042 Can you sell the house freely if you want? 如果想卖的话，您家是不是可以自由卖房?

1. Yes 是的
2. No, restricted by work unit 不是，受到单位的限制

PROCEDURE 程序:

If HA039 = 7 , then skip to HA045. 如果 HA039 = 7 ，请跳至 HA045。

HA043 How much of your own money did you spend on the house (including loan-financed)? 买/建/换这所房子你家花了多少钱? ____ 10,000Yuan 万元 [soft check<10, >500]

[IWER: Skip to HA045 if R answered HA043. If not, ask unfolding brackets. 如果答卷人回答了 HA043，跳至 HA045，否则，提问分级展开问题。]

HA044 [IWER: If R is unwilling to answer or does not remember, ask unfolding bracket questions here. 访员注意：如果受访者不愿回答或者忘记了，在此处分级展开提问]

10,000/20,000/50,000/100,000/200,000 Yuan 元

PROCEDURE 程序:

If HA040 = 1, then skip to HA051. 如果 HA040 = 1 ，请跳至HA051。

If HA039 = 6, then skip to HA051. 如果 HA039 = 6 ，请跳至HA051。

HA045 What would you have to pay if you had paid a market-set price for the same housing? 同样房子当时的市场价格是多少? Total price 总价格 ____ (**HA045_1**) 10,000 Yuan 万元 [soft check >500] or unit price 或单位价格 ____ (**HA045_2**) 1,000 Yuan/m^2 千元/平方米 [soft check >30]

HA051 What is the construction area of the house? 这房子的建筑面积是多大? ____m^2 平方米 [soft check<10 or >500]

[Skip to next house until the last house 跳至下一处房产，直至循环结束]

HA051_W2_1 BRANCHPOINT:

If the household is a new interview household, skip to HA052 如果该家户是新家户，则跳至HA052

[CAPI：下载上一轮其他房产的信息，对于每一处，询问 HA026_W2_1-HA026_W2_14_bracket 的循环 (循环序号从 1 开始)。循环结束之后，跳至HA052。]

[现在我们想了解一下上次访问时您提到的其他房产的情况。]

HA026_W2_1 您上次访问时回答的 [下载上次访问时的相关住宅信息] 住宅是怎么处置的?

1. 现在自己住着 → 结束本循环

2. Empty 空着 → 结束本循环
3. Sold 出售
4. Rented out 出租 → 结束本循环
5. Being torn down 被拆迁 → skip to HA026_W2_6 跳至 HA026_W2_6
6. My child(ren) stay in it 子女住着 → skip to HA026_W2_9 跳至 HA026_W2_9
7. My parent(s) stay in it 父母住着 → skip to HA026_W2_9 跳至 HA026_W2_9
8. Others (relatives, friends) stay in it 给其他人 (亲戚, 朋友) 居住 → skip to HA026_W2_9 跳至 HA026_W2_9
9. Give to others as gift 赠予他人 → skip to HA026_W2_12 跳至 HA026_W2_12
10. Last residence is rented, no longer rent it 上次住宅是租来的，现已不再租用 → 结束本循环
11. Other, please specify 其它，请注明 ____ (**HA026_W2_1a**) → 结束本循环
12. Last record is wrong, the mentioned residence is not ours 上次记录有误，提及的住宅不是我家的 → 结束本循环
13. The house talked just now 刚才问的现在住的房子 → 结束本循环

HA052 What is the monthly rental income for all room or houses owned by main respondent or spouse, that you are currently leasing? 主要受访者（或其配偶）拥有的房屋，正在出租的，每月一共能收取多少房租？

1. ____ (**HA052_1**) Yuan/Month 元/月 [soft check >20,000]
2. Not applicable 没有出租

HA054_W3 Do you or other household members take out a bank loan to finance the purchase of your and your spouse's houses now? 您家是否正在用银行按揭贷款来购买您的这些房子？（访员注意：针对受访者和其配偶拥有的所有房产提问，已还清贷款不算）

1. Yes 是
2. No 否 skip to HA054 跳至 HA054

HA055_W3 What is the outstanding amount of the loans? 连本金带利息，还有多少贷款没还清？ ____10,000Yuan 万元

HA056_W3 How much is the unpaid interest? 其中应付利息是 ____10,000 Yuan 万元

HA057_W3 What is the monthly mortgage payment? 每月要还多少贷款？ ____10,000 Yuan 万元 [soft check >20,000]

PART 3 Land 第三部分：土地

[The following questions pertain to your land. 下面是关于土地的问题]

HA054 Does your and your spouse have any collective distributing or rent cultivated land, forest land, pasture and/or pond? (Choose all that apply) 您和您的配偶是否有集体分配的耕地、林地、牧场或水塘，或者从别人那里租用了耕地、林地、牧场或水塘？（可多选）

1. Cultivated land 耕地
2. Forest land 林地
3. Pasture 牧场
4. Pond 水塘
5. None 没有 → Skip to HA064 请跳至HA064

PROCEDURE 程序:
According to all options choosed in HA054 , ask HA055 -HA063 in loop. 针对 HA054 的所有选择，循环询问 HA055 -HA063 题。

HA055 How many mu of [preload answer fromHA054] do you and your spouse have? 您和您的配偶从集体分配到的 [加载 HA054 的答案] 有多少亩？[访员注意：这里只记录受访者和其配偶分配到的，其他家户成员分配到的要剥离出去] ____ Mu 亩 [soft check > 50]

HA056 How many mu of them are irrigable? 其中可灌溉面积是多少亩？____Mu 亩 [hard check cannot be > HA055]

HA057 What is the rent per mu per year you would get if you rent out all your [preload answer from HA054]? 如果出租的话，每亩 [加载 HA054 的答案] 每年的租金会是多少？____ Yuan per mu per year 元每年每亩 [soft check < 10, > 4000]

HA058 Did you rent out any of your [preload answer from HA054] in the past year? 过去一年您和您的配偶是否将 [加载 HA054 的答案] 出租给了其他人？
1. Yes 是
2. No 否 → Skip to HA061 请跳至 HA061

HA059 How much [preload answer from HA054] did you rent out the past year? 过去一年您和您的配偶出租了多少亩 [加载 HA054 的答案]？ ____ Mu 亩 [hard check, cannot be > HA055]

HA060 How much rental income did you earn in the past year? 过去一年您和您的配偶出租 [加载 HA054 的答案] 收到的租金是多少？ ____ Yuan 元

HA061 Did you rent in any [preload answer from HA054] from others (including the collective) in the past year? 过去一年您和您的配偶是否从别人（包括集体）那租用了 [加载 HA054 的答案]？
1. Yes 是
2. No 否 → Skip toHA064 请跳至 HA064

HA062 How much did you rent in the past year? 过去一年您和您的配偶租用了多少亩 [加载 HA054 的答案]？ ____ Mu 亩 [soft check > 100]

HA063 How much rent did you pay in the past year? 过去一年您和您的配偶租用 [加载 HA054 的答案] 付出的租金是多少？ ____ Yuan 元 [soft check >20,000]

HA064 How much rental income did you earn for any other household assets other than housing or land? (trees, use of fixed capital, durables, or livestock)? 除了出租房屋或者土地，过去一年您和您的配偶从出租其他家庭资产（如树木、固定资本的使用、耐用品或者牲畜）收取了多少租金？

1. ____ (**HA064_1**) Yuan 元
2. Not applicable 没有出租

PART 4 Equipments, Consumption durables, and Valuables. 家用设备、耐用消费品和其他贵重物品

HA065 Do you and your spouse own the following assets? (Choose all that apply) 您和您的配偶有下列物品吗？（可多选）

For all categories, add [soft check <100 or >30,000] unless other check is written

[For each asset owned by main respondent and his/her spouse] what is the asset's current value? (Yuan) 针对受访者和其配偶有的每一项物品提问：当前值多少？（元）

1. Automobile 汽车 [soft check <3000, >500,000] ____ (**HA065_1[1]**)
2. Electric Bicycle 电动自行车 ____ (**HA065_1[2]**)
3. Motorcycle 摩托车 ____ (**HA065_1[3]**)
4. Refrigerator 电冰箱、冰柜 ____ (**HA065_1[4]**)
5. Washing machine 洗衣机 ____ (**HA065_1[5]**)
6. TV 电视机 ____ (**HA065_1[6]**)
7. Computer 家用电脑 ____ (**HA065_1[7]**)
8. Stereo system 组合音响 ____ (**HA065_1[8]**)
9. Video camera 摄像机 ____ (**HA065_1[9]**)
10. Camera 照相机 ____ (**HA065_1[10]**)
11. Air conditioner 空调 ____ (**HA065_1[11]**)
12. Mobile phone 手机 ____ (**HA065_1[12]**)
13. Furniture 值钱家具 ____ (**HA065_1[13]**)
14. Music instrument 高档乐器 ____ (**HA065_1[14]**)
15. Valuable decorations, ornaments 昂贵的装饰、物品 ____ (**HA065_1[15]**)
16. Treasures and precious metal (such as gold) 珠宝和贵重金属（如黄金等）__________ (**HA065_1[16]**)
17. Antiques, valuable paintings and calligraphic work, and other artistic work 古董、字画及其他艺术品 ____ (**HA065_1[17]**)
18. None 没有以上物品

HA066 Do you and your spouse own the following fixed capital assets? How much are the assets worth? (check all that apply)) 您和您的配偶有下列固定资产吗？现在值多少钱？（可多选）

1. Tractor, current value____ Yuan 拖拉机，现在值 ____ (**HA066_1_1**) 元 [soft check $< 1000, > 30,000$]

2. Thresher, current value____ Yuan 脱粒机 (包括打稻机) ，现在值 ____ (**HA066_1_2**) 元 [soft check < 100 or $> 10,000$]
3. Tractor tools, current value____ Yuan 机引农具，现在值 ____ (**HA066_1_3**) 元 [soft check < 100 or $> 10,000$]
4. Water pump, current value____ Yuan 抽水机 (包括水泵)，现在值 ____ (**HA066_1_4**) 元 [soft check < 100 or $> 10,000$]
5. Processing equipment, current value____ Yuan 加工机械, 现在值 ____ (**HA066_1_5**) 元 [soft check < 100 or $> 10,000$]
6. None 没有以上固定资产

HA067 What is the current value of other fixed capital assets used in household production or self-employed activities?) 您和您的配偶用于家庭生产、个体经营或开办私营企业的其他固定资产现在值多少钱？ ____ Yuan 元

[IWER: Be sure to ask about fixed capital assets used in all self-employment activities, do not count assets already reported above. 访员注意：务必要询问用于所有家庭生产、个体经营或开办私营企业活动的固定资产，不包括前面问过了的固定资产。]

HA068 Do you and your spouse have any other durable or fixed assets worth 500 yuan or more?) 您和您的配偶有其他 500 元及以上的其他耐用品或固定资产吗？

1. Yes 有 How much are the assets worth? 现在值多少？ ____ (**HA068_1**) Yuan 元 [hard check > 500] [soft check $> 50,000$]
2. No 没有

HA076 How often did the respondent receive assistance in answering section Household assets? 受访者填写该部分问卷时是否求助？

[IWER: If it is answered by a proxy, please record the respondent’s reaction. 访员注意：如果是协助回答，请记录受访者的反应。]

1. Never 从未
2. A few times 偶尔几次
3. Most or all of the time 大多数时间

HB INDIVIDUAL ASSETS 个人资产

[IWER: Please conduct sections HB and HC when the main respondent and his/her spouse are at home. Don’t allow a proxy to complete the entire sections. 访员注意：当主要受访者和其配偶在场时，才提问 HB 和 HC 部分问卷。这部分不允许请人完全代填。]

PART 1 Financial Assets 第一部分：金融资产

[The following questions pertain to your financial asset. 下面是关于金融资产的问题。]

[IWER reminder: make sure others are not present, IWER read following instructions: the following questions pertain to your financial asset, the answers to these questions will be

kept strictly confidential and will be used for research purposes only. 访员注意：请确保没有其他人在场。请调查员念“下面是关于您的金融资产问题。我们将会对您的回答严格保密，并且仅用于学术研究。”]

HC001 How much cash is held by you and your spouse at home? 您和您爱人现在在家里有多少现金（随身携带以及放在家里的）? _____ Yuan 元 [soft check $> 50,000$ or < 100]
[IWER: Skip to HC005 if R answered HC001 and HC001 $\neq 0$. If not, ask unfolding brackets. 访员注意：如果受访者回答了HC001且回答不为 0，跳至 HC005，否则，提问分级展开问题]

HC002 [IWER: If R is unwilling to answer or does not remember, ask unfolding bracket questions here. 访员注意:如果受访者不愿回答或者忘记了,在此处分级展开提问] 500 /1,000 /2,000 /5,000 /10,000 Yuan 元

[CAPI: prompt for HC004 - HC019: IWER: for deposit, bonds, stocks, and funds, only include assets legally in his/her name. 对 HC004 - HC019 提示：访员注意：对于存款、政府债券、股票和基金，只计算法定意义上在受访者名下的财产]

HC005 What is the total amount of deposits you are currently holding in financial institutions（eg: bank）? 您现在在金融机构 (如银行等) 存了多少钱? ______ Yuan 元 [soft check $> 500,000$ or < 100]

PROCEDURE 程序:
Skip to HC007 if R answered HC005. If not, ask unfolding brackets. 访员注意：如果受访者回答了 HC005，跳至 HC007，否则，提问分级展开问题。]

HC006 [IWER: If R is unwilling to answer or does not remember, ask unfolding bracket questions here. 访员注意：如果受访者不愿回答或者忘记了，在此处分级展开提问]
2,000/10,000/50,000/100,000/500,000 Yuan 元

HC007 Do you have any government bonds (e.g. Treasury bills) in your name? 您有（记在您名下的）债券（如国库券，企业债券）吗?
1. Yes 有
2. No 没有 → Skip to HC010 请跳至HC010

HC008 What is the total face value of government bonds that you are currently holding? 您现有的所有债券 (如国库券，企业债券等) 总共面值多少?_____ Yuan 元 [soft check $> 50,000$]

PROCEDURE 程序:
Skip to HC0010 if R answered HC008. If not, ask unfolding brackets HC009. 访员注意：如果受访者回答了 HC008，跳至 HC0010，否则，提问分级展开问题HC009。

HC009 [IWER: If R is unwilling to answer or does not remember, ask unfolding bracket questions here. 访员注意：如果受访者不愿回答或者忘记了，在此处分级展开提问] 10,000 /50,000 /100,000 /200,000 /500,000 Yuan 元

HC010 Have you held any stocks in the past year in your name, excluding the equity or stock of your work unit? 过去一年，您持有过（记在您名下的）股票吗？

1. Yes 有
2. No 没有 → Skip to HC015 请跳至 HC015

HC013 What is the present market value of all the stocks you are currently holding? 您现在持有的股票当前价值多少？ ______ Yuan 元 [soft check $> 200,000$]

PROCEDURE 程序:

Skip to HC015 if R answered HC013. If not, ask unfolding brackets. 访员注意：如果受访者回答了 HC013，跳至HC015，否则，提问分级展开问题。

HC014 [IWER: If R is unwilling to answer or does not remember, ask unfolding bracket questions here. 访员注意：如果受访者不愿回答或者忘记了，在此处分级展开提问] 10,000 /50,000 /100,000 /200,000 /500,000 Yuan 元

HC015 Have you held any funds in your name in the past year? 过去一年，您持有过（记在您名下的）基金吗？

1. Yes 有
2. No 没有 → Go to PROGRAM before HC020 请跳至 HC020 前面的程序控制

HC018 What is the present market value of all the mutual funds you are currently holding? 您现在持有的基金当前价值多少？ ______ Yuan 元 [soft check $> 200,000$]
[IWER: Skip to HC020 if R answered HC018. If not, ask unfolding brackets. 访员注意：如果受访者回答了 HC018，跳至HC020，否则，提问分级展开问题。]

HC019 [IWER: If R is unwilling to answer or does not remember, ask unfolding bracket questions here. 访员注意：如果受访者不愿回答或者忘记了，在此处分级展开提问] 10,000 /50,000 /100,000 /200,000 /500,000 Yuan 元

PROCEDURE 程序:

if HC005 $= 0$ and HC007 $= 2$ and HC010 $= 2$ and HC015 $= 2$, then skip HC020. 如果HC005 $= 0$，且HC007，HC010，HC015 答案都为 2，那么请跳过 HC020。

HC020 What percentage of the deposits, bonds, stocks, and funds held in your name is fully controlled by you and not your spouse? (%) 您名下的存款、政府债券、股票和基金有百分之多少是完全由你支配的？ ____ 0...100 % [hard check $\geq 0, \leq 100$]

HC021 Do you have any other deposits, bonds, stocks, or funds that belong to you but which are held in a person's name other than you or your spouse? 您是否还有其他存款、政府债券、股票和基金没有记在您或您爱人的名下？

1. Yes 是
2. No 否 → Skip to HC027 请跳至 HC027

HC022 What is the value of such assets? 这些资产的总价值是多少? ______ yuan 元 [soft check $> 200,000$]

HC027 Do you have public housing funding? 您有住房公积金吗?

1. Yes 有
2. No 没有 → Skip to HC030 请跳至HC030

HC028 What is the total amount of money in your public housing fund? 您现在住房公积金账户上一共有多少钱? ______ Yuan 元 [soft check $> 100,000$]

PROCEDURE 程序:

Skip to HC030 if R answered HC028. If not, ask unfolding brackets HC029. 访员注意:如果受访者回答了 HC028, 跳至 HC030, 否则, 提问分级展开问题HC029。

HC029 [IWER: If R is unwilling to answer or does not remember, ask unfolding bracket questions here. 访员注意:如果受访者不愿回答或者忘记了, 在此处分级展开提问]
5,000/10,000/50,000/100,000/200,000 Yuan 元

HC030 Do you have Jizikuan that your work unit or other work units have collected from you and are still holding (Jizikuan is fund individuals provided to the work unit for the purpose of investment, building apartments, etc.)? 您有没有交给自己单位或其他单位的集资款还未返还?

1. Yes 有
2. No 没有 → Skip to HC033 请跳至 HC033

HC031 What is the amount of your jizikuan? 您的集资款总金额是多少? ______Y uan 元 [soft check $> 200,000$]

PROCEDURE 程序:

Skip to HC033 if R answered HC031. If not, ask unfolding brackets HC032. 访员注意:如果受访者回答了 HC031, 跳至 HC033, 否则, 提问分级展开问题 HC032。

HC032 [IWER: If R is unwilling to answer or does not remember, ask unfolding bracket questions here. 访员注意:如果受访者不愿回答或者忘记了, 在此处分级展开提问]
5,000/10,000/50,000/100,000/200,000 Yuan 元

HC033 Do you have any unpaid salary that your work unit still owes you? 目前, 有单位拖欠您的工资吗?

1. Yes 有
2. No 没有 → Skip to HC036 请跳至 HC036

HC034 What is the amount of your unpaid salary? 一共拖欠您多少工资? ______ Yuan 元 [soft check $> 100,000$]

PROCEDURE 程序:

Skip to HC036 if R answered HC034. If not, ask unfolding brackets HC035. 访员注意:如果受访者回答了 HC034,跳至 HC036,否则,提问分级展开问题 HC035。

HC035 [IWER: If R is unwilling to answer or does not remember, ask unfolding bracket questions here. 访员注意:如果受访者不愿回答或者忘记了,在此处分级展开提问] 5,000/10,000 /50,000 /100,000 /200,000 Yuan 元

HC036 Have you participated in any private lending during the past year? 您在过去一年里有没有参与过民间借贷(如标会等)?

1. Yes 有
2. No 没有 → Skip to HC039_W3 请跳至 HC039_W3

HC036_W3 What is the total amount of funds that you are still obligated to private lending during the past year? 过去一年您通过民间借贷获得了多少钱? ______ Yuan 元 [soft check $> 100,000$]

PROCEDURE 程序:

Skip to HC037 if R answered HC036_W3. If not, ask unfolding brackets HC036_W3_1. 访员注意:如果受访者回答了 HC036_W3,跳至 HC037,否则,提问分级展开问题 HC036_W3_1。

HC036_W3_1 [IWER: If R is unwilling to answer or does not remember, ask unfolding bracket questions here. 访员注意:如果受访者不愿回答或者忘记了,在此处分级展开提问] 5,000/10,000 /50,000 /100,000 /200,000 Yuan 元

HC037 What is the total amount of funds that you are still obligated to pay to private lending? 您目前还欠民间借贷多少钱? ______ Yuan 元

PROCEDURE 程序:

Skip to HC039_W3 if R answered HC037. If not, ask unfolding brackets HC038_W3. 访员注意:如果受访者回答了 HC037,跳至 HC039_W3,否则,提问分级展开问题 HC038_W3。

HC038_W3 [IWER: If R is unwilling to answer or does not remember, ask unfolding bracket questions here. 访员注意:如果受访者不愿回答或者忘记了,在此处分级展开提问] 5,000/10,000/50,000/100,000/200,000 Yuan 元

HC039_W3 Have you lent to other families or individuals and not been repaid by them? 除了前面提到的,现在还有没有人或单位欠您的钱没还的?

1. Yes 有
2. No 没有 → Skip HC040_W3 and HC041_W3 请跳过 HC040_W3 和HC041_W3

HC040_W3 What is the total amount of the loans? 他们一共还欠你多少钱? ______ Yuan 元 [soft check $> 500,000$]

PROCEDURE 程序:
Skip HC041_W3 if R answered HC040_W3. If not, ask unfolding brackets HC041_W3. 访员注意：如果受访者回答了 HC040_W3，跳过HC041_W3，否则，提问分级展开问题 HC041_W3。

HC041_W3 [IWER: If R is unwilling to answer or does not remember, ask unfolding bracket questions here. 访员注意：如果受访者不愿回答或者忘记了，在此处分级展开提问]
5,000/10,000/50,000/100,000/200,000 Yuan 元

PART 2 DEBTS 第二部分：债务

[The following questions pertain to your debt. 下面是关于您债务的问题。]

HD001 What is the total amount of loan that you haven't repaid yet (not including loans for house)? 您尚未还清的贷款总额（不包括买房、建房、装修的贷款）是多少？ ______ Yuan 元 [soft check $> 500,000$]

PROCEDURE 程序:
Skip to HD003 if R answered HD001. If not, ask unfolding brackets HD002. 访员注意：如果受访者回答了HD001，跳至 HD003，否则，提问分级展开问题 HD002。

HD002 [IWER: If R is unwilling to answer or does not remember, ask unfolding bracket questions here. 访员注意：如果受访者不愿回答或者忘记了，在此处分级展开提问]
5,000/10,000/50,000/100,000/500,000 Yuan 元

HD003 What is the amount of your credit card balance? 您有信用卡吗？有的话现在您的信用卡所欠金额是多少？ _____ Yuan 元 [soft check $> 50,000$]（如果没有，请填 0）

PROCEDURE 程序:
Skip to HD004_W3 if R answered HD003 . If not, ask unfolding brackets HD004. 访员注意：如果受访者回答了 HD003，跳至 HD004_W3，否则，提问分级展开问题 HD004。

HD004 [IWER: If R is unwilling to answer or does not remember, ask unfolding bracket questions here. 访员注意：如果受访者不愿回答或者忘记了，在此处分级展开提问]
500/1,000/5,000/10,000/50,000 Yuan 元

HD004_W3 您还欠其他家庭、个人或单位的钱总数是多少？(不包括前面提到的贷款及信用卡欠款等) _____ Yuan 元 [soft check $> 50,000$]（如果没有，请填 0）

PROCEDURE 程序:
Skip HD004_W3_1, if R answered HD004_W3. If not, ask unfolding brackets HD004_W3_1. 访员注意：如果受访者回答了 HD004_W3，跳过HD004_W3_1，否则，提问分级展开问题 HD004_W3_1。

HD004_W3_1 [IWER: If R is unwilling to answer or does not remember, ask unfolding bracket questions here. 访员注意：如果受访者不愿回答或者忘记了，在此处分级展开提问]
500/1,000/5,000/10,000/50,000 Yuan 元

HD005_W3 您有否遭遇诈骗的经历？

1. Yes 有
2. No 没有 → Skip HD005_W3_1 请跳过 HD005_W3_1

HD005_W3_1 有的话，损失多少？ ______ Yuan 元

HD012 How often did the respondent receive assistance in answering section H ASSETS?
受访者填写该部分问卷时是否求助？
[IWER: If it is answered by a proxy, please record the respondent’s reaction. 访员注意：如果是协助回答，请记录受访者的反应。]

1. Never 从未
2. A few times 偶尔几次
3. Most or all of the time 大多数时间

This page intentionally left blank

I HOUSING CHARACTERISTICS 住房情况

PROCEDURE 程序:
Only main respondent answer I002 - I026. 仅主要受访者回答 I002 - I026

I002 What is the total housing land area? 宅基地的面积有多大?（宅基地是指农村集体经济组织为保障农户生活需要而拨给农户建造房屋及小庭院使用的土地） _____ m^2 平方米 [soft check < 10, > 1000]
[访员注意：如果没有此项，请填写"0"]

I003 Is your residence used for business as well? 这个住房兼作生产经营用房吗?
1. Yes 是
2. No 否

I004 What type of structure is this building? 这个住房是什么建筑结构?
1. Concrete and steel/Bricks and wood 钢筋混凝土或砖木结构
2. Adobe 土坯房/土房
3. Wood/Thatched 木草屋/茅草屋
4. Cave dwelling 窑洞
5. Mongolian yurt/Woolen felt/Tent 蒙古包/毡房/帐篷
6. Stone 石头房
7. Other, please specify 其它结构，请注明 _____ (**I004_1**)

I005 When was this house built? 这个房子是什么时候建成的? _____ Year 年 (**I005_1**)
If R is unclear about year, please choose among following items. 如果不清楚具体年份，从以下选项中选择:
1. 0 – 5 years 0 – 5 年
2. 5 – 10 years 5 – 10 年
3. 10 – 20 years 10 – 20 年
4. 20 – 30 years 20 – 30 年
5. 30 – 40 years 30 – 40 年
6. More than 40 years 40 年以上

I006 Is the building one story or multi-level building? 住房所在建筑是平房还是楼房?
1. One-story building 平房 → skip to I007 请跳至I007
2. Common multi-story building 一般楼房 → skip to I008 请跳至I008
3. Self-contained multi-story building 独门独户楼房 → skip to I009 请跳至I009

I007 Is the story independent or compound? 是独立的平房，还是大杂院?
1. Independent story 独立的平房
2. Compound 大杂院

I008 Which story is this building on? 该住房在第几层? _____ Storey 层

PROCEDURE 程序:
If I008 > 1, ask I009. 如果 I008 > 1，询问I009

I009 Does it has elevator? 有电梯吗?
1. Yes 是
2. No 否

I010 Are there any handicapped facilities (e.g., non-stair ramp)? 住宅是否有无障碍通道?（例如：没有台阶的斜坡等）
1. Yes 是
2. No 否

PROCEDURE 程序:
If I010 = 2, ask I011. 如果 I010 = 2，询问I011

I011 How many steps had to be climbed to get to the main entrance of the household's flat? 从外面回来要爬多少个阶梯才能到家门口?
[IWER: Do not count steps if an elevator is available 访员注意：可以坐电梯而不用爬的阶梯忽略不计]
1. 0 0 个
2. 1 to 5 1 – 5 个阶梯
3. 6 to 15 6 – 15 个阶梯
4. 16 to 25 16 – 25 个阶梯
5. More than 25 25 个阶梯以上

I012 How many bedrooms, living rooms, bathrooms, and kitchens are there in your residence? 您家现在的房屋有 _____ (**I012_1**) bedrooms 室 _____ (**I012_2**) living rooms 厅 _____ (**I012_3**) toilets 卫生间(厕所) _____ (**I012_4**) kitchens 厨房 _____ (**I012_5**) balcony 阳台 [soft check: > 20]

PROCEDURE 程序:
Ask I013 if no toilets in the answer to I012. 如果I012答案中没有卫生间，则提问I013

I013 How far is the nearest toilet to your house? 离您家最近的厕所多远? _____ (**I013**) Meters 米 [soft check: > 500]

I014 What is the type of toilet? 厕所是什么样的? 是蹲坑式还是坐式?
1. Toilet without a seat 蹲坑式
2. Toilet with a seat 坐式 → skip to I016 请跳至I016

I015 Is the toilet flushable? 厕所能冲水吗?
1. Yes 是
2. No 否

I016 Does your residence have electricity? 您家的住房是否有电？
1. Yes 是
2. No 否

I017 Does your residence have running water? 是否有自来水？
1. Yes 是
2. No 否

I018 Is there in-house shower or bath facility? What type? 住房内有无洗澡设施？是什么样的？
1. Hot water provided 统一供热水
2. Water heater installed by the household 家庭自装热水器
3. No 无

I019 Does your residence have coal gas or natural gas supply? 是否有管道煤气或天然气？
1. Yes 是
2. No 否

I020 Does your residence have heating? 是否带供暖设施（不包括土暖气和可制暖的空调）？
1. Yes 是
2. No 否 → skip to I022 请跳至I022

I021 What is the main heating energy source? 供暖所用的主要能源是什么？
1. Solar 太阳能
2. Coal 煤炭、蜂窝煤
3. Natural gas 管道天然气或煤气
4. Liquefied Petroleum Gas 液化石油气
5. Electric 电
6. Crop residue/Wood burning 秸秆、柴火
7. Other, please specify 其他，请注明 _____ (**I021_1**)

I022 What is the main source of cooking fuel? 做饭用的主要燃料是什么？
1. Coal 煤炭、蜂窝煤
2. Natural gas 管道天然气或煤气
3. Marsh gas 沼气
4. Liquefied Petroleum Gas 液化石油气
5. Electric 电
6. Crop residue/Wood burning 秸秆、柴火
7. Other, please specify 其他，请注明 _____ (**I022_1**)

I023 Does your residence have a telephone connection? 您家装电话了吗？
1. Yes 是
2. No 否

I024 Does your residence have broad-band internet connection? 您家可以宽带上网吗？

1. Yes 是
2. No 否

I027_W3 Does your residence have an air cleaner in your home? 您家里是否有空气净化器？
1. Yes 是
2. No 否

I025 [Interviewer records it 访员自己记录] How clear and tidy is in this household? 这户人家的室内整洁度如何？
1. Excellent 非常整洁
2. Very clear 很整洁
3. Clear 整洁
4. Fair 一般
5. Poor 不整洁
6. Not applicable 不适用

I026 [Interviewer records it 访员自己记录] How is the temperature in this household? 这户人家的室内温度如何？
1. Very hot 很热
2. Hot 比较热
3. Bearable 还可以
4. Cold 比较冷
5. Very cold 很冷
6. Not applicable 不适用
